# Towards Greener Synthesis of Substituted 3-Aminophthalates Starting from 2*H*-Pyran-2-ones via Diels–Alder Reaction of Acetylenedicarboxylates

**DOI:** 10.3390/molecules30112271

**Published:** 2025-05-22

**Authors:** Dominik Fendre, Miha Lukšič, Krištof Kranjc

**Affiliations:** Faculty of Chemistry and Chemical Technology, University of Ljubljana, Večna pot 113, SI-1000 Ljubljana, Slovenia; df9313@student.uni-lj.si (D.F.); miha.luksic@fkkt.uni-lj.si (M.L.)

**Keywords:** heterocycles, 2*H*-pyran-2-ones, [4+2] cycloaddition, phthalic esters, anilines

## Abstract

The aim of this work was to prepare a large set of variously substituted 3-aminophthalates starting from substituted 3-acylamino-2*H*-pyran-2-ones acting as dienes in Diels–Alder reactions with dialkyl acetylenedicarboxylates having the role of dienophiles. These thermally allowed [4+2] cycloadditions were taking place with normal electron demand due to rather electron-deficient dienophiles and relatively electron-rich dienes; however, they still required quite harsh reaction conditions: heating in closed vessels at 190 °C for up to 17 h was sufficient in most cases (albeit for a few reactions the time needed was up to 58 h) to achieve conversions above 95%. Such conditions, unfortunately, necessitated the use of a larger excess of dienophiles (as undesired polymerization takes place concomitantly); nevertheless, the straightforward isolation procedures enabled access to the target compounds in moderate to high yields (average yield 56%). All products were characterized by standard analytical and spectroscopic methods. With the goal of changing the reaction conditions to be more environmentally friendly, we investigated the effect of various solvents (water, *n*-butanol, butyl acetate, xylene, *para*-cymene, *n*-nonane, etc.) and the temperature applied (130–190 °C) on the conversion. We found that higher temperatures are necessary in most cases (except for the most reactive 2*H*-pyran-2-ones) regardless of the solvent used. Relative reactivity was determined for both sets of reactants and the experimentally obtained data show good agreement with the computational results.

## 1. Introduction

2*H*-Pyran-2-ones [1,2] represent a versatile class of compounds, being key structural fragments in many natural compounds as well as in their synthetic analogues. 3-Acylamino-2*H*-pyran-2-ones have already demonstrated various reactivity patterns, the application as dienes in [4+2] cycloadditions [3] being one of their most prominent roles [4]. It was already shown that 2*H*-pyran-2-ones can react with a variety of alkenes acting as dienophiles, yielding oxabicyclo [2.2.2]octenes that usually undergo spontaneous elimination of CO_2_, producing cyclohexadiene intermediates [5,6]; however, sometimes under suitable conditions even these CO_2_-bridged systems can be isolated and characterized [7]. Cyclohexadiene systems can either be oxidized (aromatized) towards aniline derivatives or can act as novel dienes reacting with another molecule of dienophiles, producing bicyclo[2.2.2]octenes [8]. Reactions with alkynes, on the other hand, provide less diversity, as oxabicyclo[2.2.2]octadienes obtained in the first step always undergo CO_2_ elimination, yielding aniline derivatives, formally identical to those obtained with the cycloaddition of corresponding alkenes with subsequent aromatization [9,10,11,12,13,14]. Some such alkyne cycloadditions have already featured in detailed computational studies [15,16], thus supplementing experimental results. Density functional theory (DFT) calculations have revealed that strain, substituent effects, and distortion energies play crucial roles in determining reaction feasibility, particularly in intramolecular variants [17].

When anilines are the desired final products, two possibilities thus arise: (i) either application of alkynes as dienophiles or (ii) application of alkenes, followed by an aromatization step [9,10]. Thus, to enable access to the 3-aminophthalates, both possibilities are viable; however, their weak and strong points are different. Approach (ii) is less favourable because an additional aromatization step is required, necessitating the application of a suitable oxidant (dehydrogenation agent); on the other hand, the availability of variously substituted fumaric and maleic acid derivatives is larger than the corresponding acetylenedicarboxylates, making approach (i) less attractive. However, 2*H*-pyran-2-ones are generally poorly reactive dienes due to their (at least partial) delocalization of the π electrons, thus necessitating rather harsh reaction conditions. For many cases in the literature, refluxing in solvents with boiling points above 200 °C was needed, thus complicating the isolation procedures and decreasing the yields. Therefore, we wanted to use environmentally less problematic solvents with lower boiling points (so that straightforward removal via rotatory evaporation would be possible, thus avoiding the need for chromatographic separations). To reach the necessary reaction temperatures, closed reaction vessels were used.

Substituted dialkyl 3-acylaminophtalates have alternatively been synthesized by a number of different approaches: electrocyclization of water-based 2,3-diarylmaleate salts with a subsequent photorearrangement reaction [18], Diels–Alder reaction of dimethyl acetylenedicarboxylate and 2-aryl-4*H*-furo[3,2-*b*]pyrrole with subsequent rearrangement [19], gold-catalyzed three-component reaction [20], Diels–Alder reaction of anellated 2-aminothiophenes and dimethyl acetylenedicarboxylate [21], Diels–Alder reaction of dimethyl acetylenedicarboxylate and substituted 5,5-dimethylcyclohexa-1,3-diene with 2-methylprop-1-ene as a leaving group [22], and the most widely investigated approach: Diels–Alder cycloaddition of dimethyl acetylenedicarboxylate and substituted 3-acylamino-2*H*-pyran-2-ones with CO_2_ as a leaving group [23,24,25,26]. Conditions of these reactions varied from reflux in high-boiling-point solvents (decalin, b.p. cca. 190 °C) to microwave-assisted heating in aqueous media; on the other hand, research on these reactions in sealed high-pressure tubes is rather scarce. The advantage of the use of closed vessels is that the reaction mixture can achieve a significantly higher temperature than the boiling point of the solvent at atmospheric pressure, so a solvent with a lower boiling point can be used instead, enabling the use of a larger palette of solvents, including ones that are available from renewable sources and/or are of lesser environmental concern.

The desired dialkyl 3-acylaminophthalates represent parts of many important natural products, such as biologically active and pharmaceutically relevant compounds, including pesticides [27]. Additionally, *N*,*N*-disubstituted 3-aminophtalates have been shown to express potent inhibitory activity against metallo-β-lactamase, and substituted 3,6-diaminophtalates have shown IMP-1 inhibitory activity [28].

## 2. Results and Discussion

**Scheme 1:**
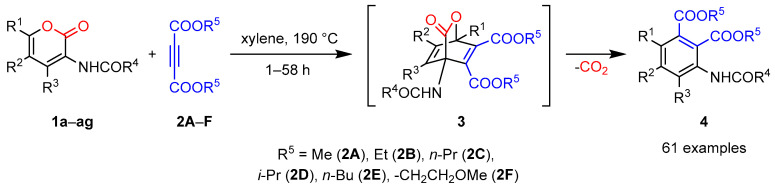
General reaction scheme of cycloadditions between 2*H*-pyran-2-ones (**1**) and acetylenedicarboxylates (**2**).

To investigate the cycloaddition between variously substituted (and fused) 3-acylamino-2*H*-pyran-2-ones **1** [29,30,31,32,33,34,35,36,37,38,39] and suitable dienophiles such as dialkyl acetylenedicarboxylates **2** [40,41,42,43], we initially started by screening various solvents for the preparation of the desired dialkyl 3-acylaminophthalates **4** (Scheme 1). Among the 3-acylamino-2*H*-pyran-2-ones **1**, we selected the one presumed to be the most reactive in normal electron demand (NED) Diels–Alder reactions, i.e., one of the most electron-rich derivatives, i.e., 5-(4-methoxyphenyl)-6-methyl derivative **1l**. In all cases, 0.5 mmol of **1l** was mixed with 1.5 mmol of dimethyl acetylenedicarboxylate (**2A**) in 2 mL of solvent and heated in a closed thick-walled glass tube. Due to various boiling points, we arranged the solvents into two groups and selected the reaction temperature accordingly: 170 °C (for *n*-nonane, xylene, anisole, DMF and *para*-cymene) and 130 °C (for xylene, *n*-BuOH, *n*-BuOAc, water and dimethyl carbonate). The conversions at 130 °C (regardless of the solvent used), determined on the basis of the H-4 signal in ^1^H NMR spectra of crude reaction mixtures after 60 min, were not sufficient, being only 31–37% (except for *n*-BuOH, where it was 43%). As 170 °C is not attainable with these solvents (due to safety reasons), we opted for the second group and again determined the conversions. The best result after 60 min at 170 °C was obtained with *n*-nonane (90% conversion); in xylene, anisole and *para*-cymene, the conversion was 77–81%, whereas in DMF it was only 62%. Thus, it can be concluded that the reaction temperature required for adequate conversions cannot be substantially decreased (even for such electron-rich dienes as **1l**) in comparison with the literature data [24,26]. Also, it was evident that the effect of the solvent on the conversion is not substantial.

According to these preliminary results, we have chosen xylene as the most suitable solvent: (i) it is possible to reach temperatures of up to 190 °C and its removal is relatively easy and energy-efficient; (ii) xylene is much cheaper in comparison with *n*-nonane (more than 10× for small quantities); and (iii) it does not represent substantial environmental risks. Additionally, it is important to stress that this is a move towards a more acceptable solvent as we avoided the use of tetralin, which was previously employed [24,26].

The following conditions were then applied for the synthesis of a set of dialkyl 3-acylaminophthalates **4** (Table 1, Table 2 and Table 3): 1.0 mmol of **1** was mixed with 2.0 mmol of dienophiles **2** in 4 mL of xylene and heated at 190 °C in a closed thick-walled glass tube. The reaction times were selected according to the substitution patterns of **1** (electron-rich versus electron-poor) and conversions were checked by ^1^H NMR spectra of crude reaction mixtures. In all cases (except for the preparation of **4Av**, **4Ax**, **4Az** and **4Aaf**, as well as **4Fa** and **4Fc**), reaction times could be reached that enabled conversions above 95%; however, in the cases of the synthesis of **4Ax** and **4Az** (from 2*H*-pyran-2-one derivatives **1x** and **1z**), even after a rather long reaction time (42 and 40 h, respectively) at 190 °C, only conversion slightly below 70% was achieved; therefore, it was necessary to modify the approach: for the preparation of **4Ax,** initially 3 eq. of **2A** was used and, after 26 h of heating, another 3 eq. was added and heating was continued for 16 h (reaching conversion of 70%). For **4Az,** initially, 3 eq. of **2A** was also used and, after 24 h of heating, another 3 eq. was added, with heating prolonged for 16 h (reaching conversion of 60%). The reason for the very low reactivity of **1x** and **1z** is their electronic push–pull characteristic that is the consequence of a rather strong electron-donating group at position 5 or 6, respectively, and an electron-withdrawing group at the other position, thus strongly delocalizing the electrons away from the 1,3-diene system of the 2*H*-pyran-2-one ring and thus strongly decreasing its diene character. It also turned out that the addition of acetylene **2** in two separate portions is beneficial, as this decreases the amount of its polymerization. For the preparation of **4Av,** conversion of 90% was reached after 21 h, whereas for **4Aaf** after 15 h 90% conversion was achieved; however, due to the complex reaction mixture formed, the isolated yield of **4Aaf** was rather low (20%). It is important to stress that (as in other cases) additional reaction time causes a larger degree of polymerization of **2** without increasing the conversion towards product **4**. Similarly, the preparation of **4Fa** (from **1b**) and **4Fc** (from **1n**) required longer reaction times (28 h) and only 85% conversions were reached (the reason being analogous as in the cases mentioned above).

Thus, a large excess of dienophiles **2** needed in all cases is the consequence of their tendency toward polymerization, which is even more pronounced when poorly reactive 2*H*-pyran-2-ones **1** are used that require longer reaction times. This drawback can be at least partially offset by adding **2** in two portions (i.e., synthesis of **4Ax** and **4Az**). To unequivocally prove the problem of polymerization, we subjected acetylene **2A** (3 mmol) in xylene (2 mL) to the same reaction conditions as applied for the synthesis of adducts **4** at 190 °C. After 2 h, 17.5 h and 25 h aliquots were sampled and analyzed by ^1^H NMR: after 2 h, there was still a substantial amount of **2A** unpolymerized, whereas after 17.5 h it was evident that the majority of **2A** had already polymerized. These results suggest that the application of solvent-free conditions for these cycloadditions would not be suitable as the solvent substantially decreases the propensity for polymerization.

A combination of ^1^H and ^13^C NMR alongside mass spectroscopy was sufficient to determine the structures of the final products **4**. For example, the ^1^H NMR spectrum of product **4Aa** shows two singlets in the aliphatic range (i.e., with chemical shifts δ 2.33 and 3.91) with integrals 3H and 6H, respectively, clearly corresponding to the 6-methyl group and both methyl substituents of the carboxylate groups. The characteristic singlet at δ 11.28 corresponds to the NH group of the amide; both doublets at δ 8.87 and 7.44 (each integrated for 1H) are 4-H and 5-H on the central aromatic ring. The remaining signals (with total integral of 5H) belong to the phenyl group. The ^13^C NMR signal of **4Aa** at δ 19.1 corresponds to the 6-methyl group; signals at δ 52.4 and 53.0 belong to carbons of the methyl substituents (in the carboxylate groups); all three carbonyl carbons are clearly identifiable with signals at appropriate chemical shifts (δ 165.5, 168.1, and 169.2). The other 14 signals are in the typical range of aromatic carbons and correspond to the central aromatic ring and phenyl substituent. Furthermore, the elemental composition of the product was confirmed by high-resolution mass spectroscopy. An analogous procedure was used to determine the structures of all other products **4** and, since there are no regio- or stereoselectivity issues, the structures provided are the only possibilities.

According to the reaction pathway, the only other possible product would be the intermediate CO_2_-bridged system **3;** however, these products would be clearly distinguishable from products **4** on the basis of their ^13^C NMR spectra (where an additional signal with chemical shift around δ 165 would be observed for the lactone carbonyl carbon) and mass spectra (where the measured mass would be higher for 44). Neither of these was observed in any case of the products obtained; therefore, we can confirm that all products isolated are of the aromatic type with structure **4**.

To obtain additional insight into the reactivity, we decided to investigate the effects of various substituents on the conversions of these—presumably—NED Diels–Alder reactions. We conducted pairwise comparisons of 2*H*-pyran-2-ones (**1**) by reacting 0.5 mmol of each with dienophile **2A** (2.0 mmol) in closed thick-walled glass vessels under otherwise identical conditions (at 170 °C, with 4 mL of xylene as the solvent). After 90 min, aliquots were sampled and analyzed by ^1^H NMR to establish the conversions for each sample of **1** being reacted (all conversions provided in the next paragraphs were obtained in this way) providing the following reactivity ranking (in order of decreasing reactivity):

(Most reactive) **1aa** > **1u** > **1l**, **1m**, **1ad** > **1k** > **1a**, **1ab** > **1o**, **1p**, **1q**, **1r**, **1s**, **1t** >

**1b**, **1c**, **1d**, **1g**, **1h**, **1j**, **1n**, **1v**, **1w**, **1ae** > **1x**, **1z** (least reactive)

Electron-rich 2*H*-pyran-2-ones **1l** and **1d**, both having a strong electron-donating substituent (*para*-methoxyphenyl) yet differing in its position, would be expected to be the most reactive; indeed, both performed well, but the position of the electron donor also had a strong influence on the reactivity: when this group is on C-5 (i.e., **1l**) the reactivity is markedly higher, but when the same electron donor is on C-6 (i.e., **1d**), the reactivity is strongly decreased (conversion for **1l:** 80%; for **1d:** only 15%). This can, however, be rationalized by the fact that groups on position 6 increase the delocalization of the electrons in the 1,3-diene system of 2*H*-pyran-2-one (**1**) more than the same groups on position 5.

Surprisingly, the highest conversions (above 90%) were achieved with **1aa** and **1u**, even though their substituents are only alkyl groups, which are commonly classified as poor electron donors. The most reactive among all 2*H*-pyran-2-ones was **1aa,** having a five-membered fused ring (conversion 95%); on the other hand, **1ab,** having a fused six-membered ring, was far less reactive (conversion 55%).

Comparison of conversions between 6-methyl- and 6-phenyl-2*H*-pyran-2-ones (**1a** and **1b**) (45% vs. 15%) suggests that a phenyl group acts only as an electron-withdrawing group and thus decreases the reactivity. On the other hand, when the 6-phenyl group in **1b** was exchanged with a stronger electron donor, such as the *para*-methoxyphenyl group (in **1d**), which was expected to significantly increase the reactivity, the reactivity of both compounds was nearly the same (15% conversion in both cases). With 6-phenyl group compound **1b** being so poorly reactive, an interesting comparison can be made with a fused tricyclic **1ad** (80%), displaying an unexpectedly high conversion (approaching 6-ethyl-5-methyl **1u** and surpassing **1a**). Electron-withdrawing groups, such as acetyl and ester groups, should decrease the reactivity; however, conversion of 5-acetyl derivative **1p** (35%) and 5-methoxycabonyl-substituted **1n** (20%) surpassed the reactivity of *para*-methoxyphenyl derivative **1d**.

Comparison between 5-methoxyphenyl-substituted 2*H*-pyran-2-one **1m** and 5-dimethoxyphenyl analogue **1l** showed that an additional electron-donating methoxy group on the phenyl substituent does not increase the reactivity (in both cases, conversions were 80%); however, conversion was slightly lower (nearly 70%) for 5-phenyl-substituted 2*H*-pyran-2-one **1k**, but still higher than with 6-methyl derivative **1a** (conversion: 45%).

The effects of the 3-acylamino group were also investigated, comparing different electron-donating or -withdrawing groups on the 3-amino group in **1**; it was determined that reactivity is not influenced much by various benzoyl moieties, with a slightly lesser reactivity observed with the *para*-nitrobenzoyl group in **1q** (28% conversion) in comparison with unsubstituted benzoyl group in **1p** or *para*-methoxybenzoyl in **1r** (both 35% conversion). No difference in reactivity was detected between 3-acetylamino derivative **1t** and 3-benzoylamino **1p** (both having 35% conversion).

When, instead of a phenyl group in the C-6 position (i.e., **1b**), other aromatic groups were present, a nearly negligible difference in reactivity was observed; for example, *para*-tolyl (in **1c**), furan-2-yl (in **1h**), thiophen-2-yl (in **1i**), naphtalen-2-yl (in **1j**) and 4-fluorophenyl (in **1w**) all exhibited conversion of around 15%. If the 6-phenyl derivative **1b** was additionally substituted on the C-4 position with a methyl group (i.e., **1ae**), no change in reactivity was detected; however, if **1b** was additionally substituted with a 5-phenyl group (i.e., **1v**), the conversion was somewhat decreased (from 15% to 10%).

To obtain deeper insight into the reactivity and make a comparison with literature data [9,10,11,15,25,26], eight reactions of 3-acylamino-2*H*-pyran-2-ones (**1a**,**b**,**d**,**l**,**p**,**u**,**x**,**aa**) with dimethyl acetylenedicarboxylate (**2A**) were selected to be investigated theoretically. Density functional theory (ωB97X-D4/def2-TZVP) was used to optimize the nuclear geometries of reactants (**1** + **2A**), bicyclic intermediate products **3** and final products (**4A** + CO_2_), as well as of transition state geometries (TS1 for reactions **1** + **2A** → **3** and TS2 for subsequent carbon dioxide elimination reactions **3** → **4A** + CO_2_). The method of Nudged Elastic Band with transition state optimization (NEB-TS) was used to identify the transition state structures. The solvent (xylene) was treated implicitly using the conductor-like polarizable continuum model (CPCM). DLPNO-CCSD(T)/def2-TZVPP theory was used to calculate the electronic energies of the optimized structures. Standard Gibbs free energies of reactants, products and transition states were corrected accordingly. Optimized structures and their energies are given in the Supporting Information, which also contains schematic representations of the reaction coordinates for all cases, as well as imaginary frequencies of transition states (TS1 and TS2) and their graphical representations. The activation standard Gibbs free energies for the selected eight reactions are given in Table 4. The activation barrier for the formation of oxabicyclo[2.2.2]octa-5,7-dien-3-ones **3** from 2*H*-pyran-2-ones **1** and **2A**, ${∆G}_{TS1}^{\#}$, is significantly higher than the free energy barrier for CO_2_ elimination from the intermediate products **3** and subsequent formation of product **4A**, ${∆G}_{TS2}^{\#}$. The differences, ${∆G}_{TS2}^{\#}-{∆G}_{TS1}^{\#}$, range from approximately 17 up to 90 kJ/mol for cases **4Aaa** and **4Ax**, respectively. This is in agreement with experimental observations, where no intermediate products **3** were found in the reaction mixtures. Comparing the activation standard Gibbs free energies of the rate-determining step, ${∆G}_{TS1}^{\#}$, we determine that the reactivity order is the same as the one observed experimentally (with the exception of **1b**). A lower value of ${∆G}_{TS1}^{\#}$ implies better reactivity of the 2*H*-pyran-2-ones **1**. The results of the calculations imply the following ranking (from the most to the least reactive): **1aa** > **1u** > **1b**, **1l** > **1a** > **1p** > **1d** > **1x** (c.f. experimental ranking above).

**Figure 1:**
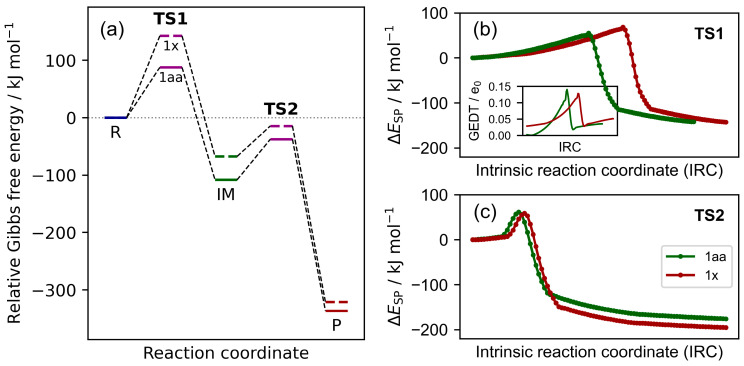
(**a**) Schematic representation of the reaction coordinate for reaction **1** + **2A** → **4A** + CO_2_ for dienes **1aa** and **1x**. R stands for reactants (**1** + **2A**), IM for bicyclic intermediate products (**3**), and P for product (**4A** + CO_2_). (**b**) Intrinsic reaction coordinate (IRC) paths for R → IM in case of dienes **1aa** (green) and **1x** (red). Inset shows changes in the global electron density transfer (GEDT) along IRC. (**c**) IRC path for IM → P. *E*_SP_ on panels (**b**,**c**) designates the single-point energy.

Figure 1a shows the relative standard Gibbs free energies of the stationary points for reactions involving dienes **1aa** (most reactive) and **1x** (least reactive) and the dienophile **2A** (reactants, R) leading to the products **4Aaa** and **4Ax** and CO_2_ (products, P) via bicyclic intermediate (IM) **3**. If we compare these two cases, we can see that the product **4Aaa** is slightly more thermodynamically stable than the product **4Ax** (${∆}_{r}G\left(1aa\to 4Aaa\right)=-336.7$ kJ/mol, ${∆}_{r}G\left(1x\to 4Ax\right)=-321.3$ kJ/mol). Reaction diagrams for other cases are given in the Supporting Information.

For these two cases, the intrinsic reaction coordinate (IRC) profiles are shown in Figure 1b,c for steps R → IM via transition state TS1 and IM → P via transition state TS2, respectively. A uniform energy profile without intermediate states was observed in all cases (TS1 and TS2), indicating a concerted reaction mechanism. The bond formation was slightly asynchronous due to the asymmetry of the diene. The inset in Figure 1b shows the changes in the absolute values of the global electron density transfer (GEDT) [44] calculated from the Hirshfeld charges (the part belonging to dienophile **2A** was selected as the framework). GEDT reaches a maximum at TS1 (for all other cases studied, GEDT values for TS1 are given in the Supporting Information). Our GEDT values are characteristic for moderately polar Diels–Alder reactions [45]. In all cases, the electron density flux is from the diene to the dienophile (**2A**), as expected for experimentally studied reactions with normal electron demand. In the case of our reactions, no zwitterionic intermediates were detected, indicated also by values of GEDT lower than 0.15*e*_0_ [46]. The value of the GEDT at TS1 for the case involving diene **1aa** is somewhat larger than in case **1x**. A higher value of the GEDT implies grater TS stabilization and therefore a lower activation barrier (faster reaction). This is consistent with the activation standard Gibbs free energies for the cycloaddition reactions of these two cases (Table 4).

To investigate the reactivity of acetylenes (**2**), the electron-rich 2*H*-pyran-2-one **1l** (0.5 mmol) was reacted with acetylenes **2A**–**F** (1.0 mmol) in 2 mL of xylene at 170 °C in a thick-walled glass tube. After 90 min, aliquots were sampled and conversions were determined with ^1^H NMR on the basis of H-4 signals. Surprisingly, the largest conversions were obtained with acetylenes **2F** and **2C** (82% and 80% conversion, respectively), followed by **2D** (65%) and **2E** (55%), whereas **2A** and **2B** were the least reactive (45% and 32%, respectively). The trend observed does not correlate with the presumed steric hindrance, nor with the electronic effects of the substituents (NED Diels–Alder reaction should be accelerated when electron-deficient dienophiles are reacted). However, this reactivity order of acetylenes (**2**) is not automatically reflected in the reaction conditions necessary for the preparation of the final products (**4**), as higher reactivity of the acetylenes (**2**) generally causes larger degree of their undesired polymerization, thus decreasing the overall yields of **4**.

**Scheme 2:**
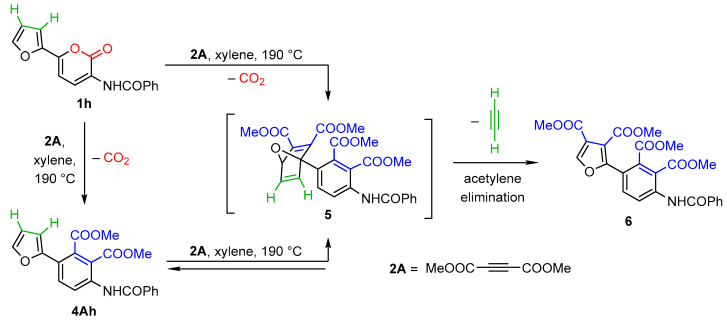
Cycloaddition of 6-furyl-substituted 2*H*-pyran-2-one **1h**.

When investigating the synthesis of furyl-substituted **4Ah** (Scheme 2), additional signals were present in ^1^H NMR spectra of crude reaction mixtures, implying the presence of possible side product(s): when 2*H*-pyran-2-one **1h** was heated at 190 °C in xylene with 10 eq. of **2A** after 2 h ^1^H NMR showed the presence of the expected product **4Ah**, with some unreacted starting **1h** as well as an additional adduct (approximate ratio of **4Ah**:**1h**:**6** = 3:1:1). Upon prolonged heating (23 h), starting **1h** was not detected any more; the ratio between **4Ah** and the additional adduct **6** was found to be 0.7:1, remaining unchanged even with the addition of another 2 eq. of **2A** and further heating for 3 h. The additional product **6** was separated from **4Ah** and found to be the tetraester double cycloadduct **6**. Intermediate **5** could not be detected as it immediately reacts either towards **6** via an irreversible retro-Diels–Alder elimination of gaseous acetylene (thus re-establishing the aromaticity) or with an analogous (though reversible) elimination of **2A** yielding **4Ah**. Adduct **6** is thus the predominant product when long reaction times are used (thermodynamic product), whereas **4Ah** could be prepared when the reaction time was shortened to 15 h (with 3 eq. of **2A**), albeit some **6** was also detected in the crude reaction mixture (**4Ah**:**6** = 3:2).

To investigate the possible effects of the increased pressure (due to the evolution of CO_2_ during the aromatization step) that might decrease the reaction progress (due to hindering the elimination of CO_2_), we compared conversions of **1l** (0.5 mmol) with **2A** (1 mmol) in 2 mL of xylene in a closed versus open vessel (both vessels were immersed in the same oil bath at 140 °C for 2 h). We found out that the conversion in the closed vessel was even slightly larger (50%) than the one in the open vessel (43%), thus showing that the pressure build-up does not cause any detectable changes in the conversion.

To obtain a rough estimate regarding the improvement reached by changing the reaction conditions from those previously applied [26], Sheldon’s E-factor [47,48] for the synthesis of **4Bl** according to our improved procedure was estimated to be around 4.18, whereas for the procedure described initially for the same compound it was around 5.19. Among all phthalates (**4**), the most favourable E-factor was found to be 3.75 for the synthesis of the product **4Cc**, therefore clearly showing that our improved procedure represents a step (albeit small) towards greener synthesis of products **4**.

## 3. Materials and Methods

### 3.1. General

Melting points were determined using an automatic OptiMelt MPA100 (Stanford Research System, Sunnyvale, CA, USA) instrument and were uncorrected. NMR spectra were recorded with a Bruker (Zürich, Switzerland) Avance III 500 spectrometer at 29 °C using TMS as the internal standard at 500 MHz for ^1^H NMR and 126 MHz for ^13^C NMR. Chemical shifts are provided as ppm values on the δ scale; the coupling constants (*J*) are given in Hertz. ^13^C NMR spectra are referenced against the central line of the solvent signal (CDCl_3_ at 77.0 ppm). IR spectra of compounds as powders were recorded on a Bruker (Zürich, Switzerland) Alpha Platinum ATR FT-IR spectrophotometer. Mass spectra were recorded using an Agilent (Santa Clara, CA, USA) 6624 Accurate Mass TOF LC/MS spectrometer via ESI ionization. Reagents and solvents were used as received from commercial suppliers with a purity of 98% or more. The xylene used was a commercially available mixture of all three isomers. Commercially available thick-walled ACE glass tubes closed by a Teflon screw-plug were used.

Starting 2*H*-pyran-2-ones (**1**) were prepared according to the published procedures [29,30,31,32,33,34,35,36,37,38,39]; briefly, **1a**–**p** and **1u**–**ag** were synthesized in a two-step one-pot procedure by heating appropriate ketones with 2 eq. of *N*,*N*-dimethylformamide dimethyl acetal (or *N*,*N*-dimethylacetamide dimethyl acetal in the case of **1ae** and **1af**) for 4 h, followed by a reaction with hippuric acid in acetic anhydride (4 h at 90 °C). Treating 5-acetyl-3-amino-6-methyl-2*H*-pyran-2-one [prepared from **1p** via amide hydrolysis by heating in concentrated H_2_SO_4_] with the corresponding acyl or aryl chloride in CH_2_Cl_2_ with the addition of pyridine at room temperature yielded 2*H*-pyran-2-ones **1q**–**t**. Dialkyl acetylenedicaboxylates **2C**–**F** were prepared according to the published procedure [40] with modified extraction.

### 3.2. Synthesis of Diels–Alder Adducts 4 and 6

2*H*-Pyran-2-one **1** (2 mmol), acetylene **2** (4 or 6 mmol, see Table 1, Table 2 and Table 3) and xylene (4 mL) were heated in a closed thick-walled glass vial at 190 °C. After the reactions were complete (for times, see Table 1, Table 2 and Table 3), the mixture was cooled to room temperature. Those products that precipitated upon cooling in an ice bath were collected by vacuum filtration (isolation I1 for **4Ac**, **4Af**, **4Ag**, **4Ak**–**n**, **4Aq**, **4Ar**, **4Aad**, **4Bh**, **4Bj**, **4Da**). In other cases, volatile components were removed with a rotary evaporator in vacuo. The oily residue thus obtained was treated with either an ice-cold MeOH/petroleum ether mixture (isolation I2, for **4Aa**, **4Ab**, **4Ai**, **4Aj**, **4Ap**, **4Av**, **4Aw**, **4Ay**, **4Aaa**–**ac**, **4Aae**, **4Bb**, **4Bc**, **4Be**–**g**, **4Bi**, **4Bl**, **4Cd**–**f**) or ice-cold MeOH (isolation I3, for **4Ad**, **4Ae**, **4Ao**), triggering the precipitation of crystalline products. In all other cases, oily residues were loaded on a chromatography column (isolation I4) packed with silica gel and eluted with various mobile phases: (i) petroleum ether/EtOAc 3:1 for **4Ah**, **4As** (with gradient to 1:1), **4At**, **4Au**, **4Az**, **4Aaf**, **4Ba**, **4Bd**, **4Bk**, **4Bm**–**o**, **4Ca**–**c**, **4Cg**, **4Db** and **4Ea**; (ii) petroleum ether/EtOAc 3:2 for **4Ax**; and (iii) petroleum ether/EtOAc 1:1 for **4Fa**–**c** and **6**. Some of the products obtained were purified by an additional chromatography column (silica gel, mobile phase petroleum ether/EtOAc 1:1 for **4At** and **4Az**; petroleum ether/EtOAc 3:1 for **4Ax** and **4Bk**; petroleum ether/EtOAc 10:1 for **4Cb** and **4Ea**; petroleum ether/EtOAc 4:1 for **4Cg**). Regardless of the isolation procedure, some of the products were additionally purified by recrystallization from EtOH (**4Ab**, **4Ac**, **4Ae**, **4Af**, **4Ah**–**j**, **4Am**, **4An**, **4Ap**, **4Av**, **4Aw**, **4Ay**, **4Aaa**–**ae**, **4Bb**–**j**, **4Bl**), petroleum ether/Et_2_O (**4As**), Et_2_O (**4Ax**, **4Az**, **4Fb**), petroleum ether (**4Bk**, **4Fc**) or petroleum ether/EtOAc (**6**).

Dimethyl 3-benzamido-6-methylphthalate (**4Aa**) [24]: pale yellow needles (MeOH); mp 118.3–119.3 °C (mp lit. [24] 110–113 °C (Et_2_O)); IR ν_max_ 3256 (N–H), 2956 (H–C-sp^3^), 1729 (COO), 1683 (NHCO), 1669, 1569, 1519, 1443, 1396 cm^−1^; ^1^H NMR (CDCl_3_, 500 MHz) δ 11.28 (1H, s, NH), 8.78 (1H, d, *J* = 8.7 Hz, H-4 or H-5), 7.99 (2H, m, Ph), 7.57 (1H, m, Ph), 7.52 (2H, m, Ph), 7.44 (1H, d, *J* = 8.7 Hz, H-4 or H-5), 3.91 (6H, m, 2 × COOCH_3_), 2.33 (3H, s, CH_3_); ^13^C NMR (CDCl_3_, 126 MHz) δ 169.2 (COO), 168.1 (COO), 165.5 (NHCO), 138.5, 135.7, 135.0, 134.6, 132.1, 130.4, 128.9, 127.3, 122.2, 114.7 (10 × C_Ar_), 53.0 (COOCH_3_), 52.4 (COOCH_3_), 19.1 (CH_3_); HREIMS *m*/*z* 328.1181 (calcd for C_18_H_18_NO_5_ (M+H)^+^, 328.1179).

Dimethyl 4-benzamido-[1,1′-biphenyl]-2,3-dicarboxylate (**4Ab**) [23]: off-white crystal flakes (EtOH); mp 155.8–157.7 °C (mp lit. [23] 153–155 °C (MeOH)); IR ν_max_ 3323 (N–H), 2947 (H–C-sp^3^), 1714 (COO), 1694, 1679 (NHCO), 1526, 1491 cm^−1^; ^1^H NMR (CDCl_3_, 500 MHz) δ 11.28 (1H, s, NH), 8.92 (1H, d, *J* = 8.7 Hz, H-5 or H-6), 8.02 (2H, m), 7.56 (4H, m), 7.39 (3H, m), 7.33 (2H, m) (2×Ph and H-5 or H-6), 3.91 (3H, s, COOCH_3_), 3.59 (3H, s, COOCH_3_); ^13^C NMR (CDCl_3_, 126 MHz) δ 168.9 (COO), 168.1 (COO), 165.6 (NHCO), 139.4, 139.2, 135.5, 135.3, 134.8, 134.4, 132.3, 128.9, 128.4, 128.3, 127.7, 127.4, 122.2, 115.2 (14 × C_Ar_), 53.1 (CH_3_), 52.2 (CH_3_); HREIMS *m*/*z* 390.1329 (calcd for C_23_H_20_NO_5_ (M+H)^+^, 390.1336).

Dimethyl 4-benzamido-4′-methyl-[1,1′-biphenyl]-2,3-dicarboxylate (**4Ac**) [20]: pale yellow crystals (EtOH); mp 154.4–157.7 °C; IR ν_max_ 3346 (N–H), 2955 (H–C-sp^3^), 1736 (COO), 1681 (NHCO), 1438 cm^−1^; ^1^H NMR (CDCl_3_, 500 MHz) δ 11.27 (1H, s, NH), 8.90 (1H, d, *J* = 8.7 Hz, H-5 or H-6), 8.02 (2H, m, Ph), 7.55 (4H, m, H-5 or H-6, Ph), 7.21 (4H, m, C_6_H_4_), 3.91 (3H, s, COOCH_3_), 3.62 (3H, s, COOCH_3_), 2.39 (3H, s, CH_3_); ^13^C NMR (CDCl_3_, 126 MHz) δ 169.0 (COO), 168.1 (COO), 165.6 (NHCO), 139.2, 137.5, 136.3, 135.5, 135.3, 134.7, 134.4, 132.2, 129.0, 128.9, 128.3, 127.3, 122.2, 115.1 (14 × C_Ar_), 53.1 (COOCH_3_), 52.2 (COOCH_3_), 21.2 (CH_3_); HREIMS *m*/*z* 404.1482 (calcd for C_24_H_22_NO_5_ (M+H)^+^, 404.1492).

Dimethyl 4-benzamido-4′-methoxy-[1,1′-biphenyl]-2,3-dicarboxylate (**4Ad**) [20]: pale brown powder (MeOH); mp 145.7–148.1 °C; IR ν_max_ 3309 (N–H), 3002, 2954 (H–C-sp^3^), 1751 (COO), 1676 (NHCO), 1585, 1507, 1488, 1457, 1436 cm^–1^; ^1^H NMR (CDCl_3_, 500 MHz) δ 11.28 (1H, s, NH), 8.89 (1H, d, *J* = 8.8 Hz, H-5 or H-6), 8.02 (2H, m, Ph), 7.56 (4H, m, H-5 or H-6 and Ph), 7.26 and 6.93 (2H each, AA’XX’, *J* = 8.7 Hz, C_6_H_4_OCH_3_), 3.91 (3H, s, COOCH_3_), 3.85 (3H, s, COOCH_3_), 3.63 (3H, s, OCH_3_); ^13^C NMR (CDCl_3_, 126 MHz) δ 169.1 (COO), 168.1 (COO), 165.6 (NHCO), 159.2, 139.1, 135.4, 135.2, 134.7, 134.5, 132.2, 131.6, 129.6, 128.9, 127.4, 122.2, 115.1, 113.8 (14 × C_Ar_), 55.3 (OCH_3_), 53.1 (COOCH_3_), 52.3 (COOCH_3_); HREIMS *m*/*z* 420.1437 (calcd for C_24_H_22_NO_6_ (M+H)^+^, 420.1442).

Dimethyl 4-benzamido-4′-nitro-[1,1′-biphenyl]-2,3-dicarboxylate (**4Ae**): pale orange powder (EtOH); mp 199.9–201.9 °C; IR ν_max_ 3352 (N–H), 2944 (H–C-sp^3^), 1730 (COO), 1706, 1673 (NHCO), 1597, 1578, 1517, 1489, 1435, 1410 cm^–1^; ^1^H NMR (CDCl_3_, 500 MHz) δ 11.32 (1H, s, NH), 8.99 (1H, d, *J* = 8.7 Hz, H-5 or H-6), 8.28 and 7.51 (2H each, AA’XX’, *J* = 8.7 Hz, C_6_H_4_NO_2_), 8.02 (2H, m, Ph), 7.61 (1H, m, Ph), 7.56 (3H, m, H-5 or H-6 and Ph), 3.93 (3H, s, COOCH_3_), 3.64 (3H, s, COOCH_3_); ^13^C NMR (CDCl_3_, 126 MHz) δ 168.3 (COO), 167.7 (COO), 165.7 (NHCO), 147.4, 145.9, 140.5, 134.8, 134.7, 134.2, 132.9, 132.5, 129.5, 129.0, 127.4, 123.6, 122.5, 115.5 (14 × C_Ar_), 53.3 (CH_3_), 52.5 (CH_3_); HREIMS *m*/*z* 435.1184 (calcd for C_23_H_19_N_2_O_7_ (M+H)^+^, 435.1187).

Dimethyl 4-benzamido-3′-chloro-[1,1′-biphenyl]-2,3-dicarboxylate (**4Af**): pale yellow powder (EtOH); mp 132.8–135.5 °C; IR ν_max_ 3361 (N–H), 2943 (H–C-sp^3^), 1746 (COO), 1691 (NHCO), 1519, 1434 cm^−1^; ^1^H NMR (CDCl_3_, 500 MHz) δ 11.31 (1H, s, NH), 8.94 (1H, d, *J* = 8.7 Hz, H-5 or H-6), 8.02 (2H, m, Ph), 7.62–7.52 (4H, m, H-5 or H-6, Ph), 7.34 (3H, m, C_6_H_4_Cl), 7.22 (1H, m, C_6_H_4_Cl), 3.92 (3H, s, COOCH_3_), 3.64 (3H, s, COOCH_3_); ^13^C NMR (CDCl_3_, 126 MHz) δ 168.6 (COO), 167.9 (COO), 165.6 (NHCO), 141.0, 139.9, 135.0, 134.8, 134.3, 134.2, 133.9, 132.3, 129.6, 129.0, 128.6, 127.9, 127.4, 126.7, 122.3, 115.2 (16 × C_Ar_), 53.2 (CH_3_), 52.3 (CH_3_); HREIMS *m*/*z* 424.0936 (calcd for C_23_H_19_NO_5_ (M+H)^+^, 424.0946).

Dimethyl 4-benzamido-[1,1′:4′,1′’-terphenyl]-2,3-dicarboxylate (**4Ag**): white powder (xylene); mp 194.4–197.5 °C; IR ν_max_ 3347 (N–H), 3028, 2948 (H–C-sp^3^), 1702 (COO), 1672 (NHCO), 1593, 1559, 1516, 1486, 1442, 1391 cm^–1^; ^1^H NMR (CDCl_3_, 500 MHz) δ 11.30 (1H, s, NH), 8.94 (1H, d, *J* = 8.7 Hz, H-5 or H-6), 8.02 (2H, m), 7.59 (8H, m), 7.46 (2H, m), 7.38 (3H, m) (2 × Ph, C_6_H_4_, H-5 or H-6), 3.92 (3H, s, COOCH_3_), 3.64 (3H, s, COOCH_3_); ^13^C NMR (CDCl_3_, 126 MHz) δ 169.0 (COO), 168.1 (COO), 165.6 (NHCO), 140.49, 140.46, 139.5, 138.2, 135.3, 135.1, 134.8, 134.4, 132.3, 128.94, 128.88, 128.87, 127.5, 127.4, 127.08, 127.01, 122.3, 115.3 (18 × C_Ar_), 53.2 (CH_3_), 52.3 (CH_3_); HREIMS *m*/*z* 466.1655 (calcd for C_29_H_24_NO_5_ (M+H)^+^, 466.1649).

Dimethyl 3-benzamido-6-(furan-2-yl)phthalate (**4Ah**): isolated by column chromatography (petroleum ether/EtOAc = 3:1), pale yellow powder (EtOH); mp 173.0–174.8 °C; IR ν_max_ 3358 (N–H), 3118, 2956 (H–C-sp^3^), 1726 (COO), 1675 (NHCO), 1590, 1522, 1493 cm^−1^; ^1^H NMR (CDCl_3_, 500 MHz) δ 11.41 (1H, s, NH), 8.95 (1H, d, *J* = 9.0 Hz, H-4 or H-5), 8.00 (2H, m, Ph), 7.88 (1H, d, *J* = 9.0 Hz, H-4 or H-5), 7.59 (1H, m, Ph), 7.54 (2H, m, Ph), 7.49 (1H, m, C_4_H_3_O), 6.57 (1H, m, C_4_H_3_O), 6.48 (1H, m, C_4_H_3_O), 3.94 (3H, s, COOCH_3_), 3.88 (3H, s, COOCH_3_); ^13^C NMR (CDCl_3_, 126 MHz) δ 169.1 (COO), 167.8 (COO), 165.6 (NHCO), 150.7, 142.9, 139.9, 134.4, 132.5, 132.31, 132.28, 128.9, 127.4, 124.0, 122.2, 115.0, 111.8, 108.2 (10 × C_Ar_, 4 × C_Fur_), 53.2 (CH_3_), 52.7 (CH_3_); HREIMS *m*/*z* 380.1128 (calcd for C_21_H_18_NO_6_ (M+H)^+^, 380.1129).

Dimethyl 3-benzamido-6-(thiophen-2-yl)phthalate (**4Ai**): pale brown powder (EtOH); mp 140.3–143.4 °C; IR ν_max_ 3311 (N–H), 2942 (H–C-sp^3^), 1711 (COO), 1679 (NHCO), 1580, 1519, 1437 cm^–1^; ^1^H NMR (CDCl_3_, 500 MHz) δ 11.44 (1H, s, NH), 8.93 (1H, d, *J* = 8.8 Hz, H-4 or H-5), 8.02 (2H, m, Ph), 7.68 (1H, d, *J* = 8.8 Hz, H-4 or H-5), 7.59 (1H, m, Ph), 7.54 (2H, m, Ph), 7.36 (1H, m, C_4_H_3_S), 7.06 (2H, m, C_4_H_3_S), 3.93 (3H, s, COOCH_3_), 3.73 (3H, s, COOCH_3_); ^13^C NMR (CDCl_3_, 126 MHz) δ 168.8 (COO), 167.8 (COO), 165.6 (NHCO), 140.2, 139.9, 136.0, 135.2, 134.4, 132.3, 128.9, 127.8, 127.4, 127.0, 126.5, 122.0, 114.7 (9 × C_Ar_, 4 × C_Thioph_), 53.2 (CH_3_), 52.5 (CH_3_) (one aromatic signal is hidden); HREIMS *m*/*z* 396.0892 (calcd for C_21_H_18_NO_5_ (M+H)^+^, 396.0900).

Dimethyl 3-benzamido-6-(naphthalen-2-yl)phthalate (**4Aj**): off-white powder (EtOH); mp 171.0–174.1 °C; IR ν_max_ 3337 (N–H), 2949 (H–C-sp^3^), 1707 (COO), 1671 (NHCO), 1578, 1523, 1492, 1435, 1400 cm^−1^; ^1^H NMR (CDCl_3_, 500 MHz) δ 11.30 (1H, s, NH), 8.95 (1H, d, *J* = 8.7 Hz, H-4 or H-5), 8.04 (2H, m, Ph), 7.86 (3H, m), 7.81 (1H, m) (H-aromatic), 7.67 (1H, d, *J* = 8.7 Hz, H-4 or H-5), 7.54 (5H, m), 7.46 (1H, m) (H-aromatic), 3.92 (3H, s, COOCH_3_), 3.55 (3H, s, COOCH_3_); ^13^C NMR (CDCl_3_, 126 MHz) δ 169.0 (COO), 168.1 (COO), 165.6 (NHCO), 139.5, 136.7, 135.5, 135.4, 135.0, 134.4, 133.2, 132.6, 132.3, 129.0, 128.2, 128.0, 127.7, 127.5, 127.4, 126.5, 126.3, 122.3, 115.3 (19 × C_Ar_), 53.1 (CH_3_), 52.3 (CH_3_) (one aromatic signal is hidden); HREIMS *m*/*z* 440.1503 (calcd for C_27_H_22_NO_5_ (M+H)^+^, 440.1492).

Dimethyl 5-benzamido-2-methyl-[1,1′-biphenyl]-3,4-dicarboxylate (**4Ak**): white crystals (xylene); mp 170.0–173.4 °C; IR ν_max_ 3329 (N–H), 2952 (H–C-sp^3^), 1707 (COO), 1681 (NHCO), 1576, 1519, 1492, 1437, 1404 cm^–1^; ^1^H NMR (CDCl_3_, 500 MHz) δ 11.46 (1H, s, NH), 8.84 (1H, s, H-6), 8.00 (2H, m, Ph), 7.53 (3H, m, Ph), 7.42 (3H, m, Ph), 7.34 (2H, m, Ph), 3.95 (3H, s, COOCH_3_), 3.94 (3H, s, COOCH_3_), 2.18 (3H, s, CH_3_); ^13^C NMR (CDCl_3_, 126 MHz) δ 169.7 (COO), 168.0 (COO), 165.5 (NHCO), 148.7, 140.2, 138.5, 136.4, 134.6, 132.1, 129.0, 128.9, 128.3, 127.83, 127.80, 127.3, 123.4, 113.2 (14 × C_Ar_), 53.1 (COOCH_3_), 52.5 (COOCH_3_), 17.2 (CH_3_); HREIMS *m*/*z* 404.1490 (calcd for C_24_H_22_NO_5_ (M+H)^+^, 404.1492).

Dimethyl 5-benzamido-4′-methoxy-2-methyl-[1,1′-biphenyl]-3,4-dicarboxylate (**4Al**) [24]: white crystals (EtOH); mp 171.4–173.2 °C (mp lit. [24] 162.5–165.5 °C (MeOH)); IR ν_max_ 3264 (N–H), 2949 (H–C-sp^3^), 1714 (COO), 1694 (NHCO), 1673, 1580, 1508, 1441 cm^−1^; ^1^H NMR (CDCl_3_, 500 MHz) δ 11.46 (1H, s, NH), 8.83 (1H, s, H-6), 8.00 (2H, m, Ph), 7.56 (1H, m, Ph), 7.52 (2H, m, Ph), 7.28 and 6.96 (2H each, AA’XX’, *J* = 8.7 Hz, C_6_H_4_OCH_3_), 3.94 (3H, s, COOCH_3_), 3.93 (3H, s, COOCH_3_), 3.86 (3H, s, OCH_3_), 2.19 (3H, s, CH_3_); ^13^C NMR (CDCl_3_, 126 MHz) δ 169.8 (COO), 168.0 (COO), 165.5 (NHCO), 159.3, 148.4, 138.5, 136.4, 134.6, 132.6, 132.1, 130.3, 128.9, 127.9, 127.3, 123.5, 113.7, 112.8 (14 × C_Ar_), 55.4 (OCH_3_), 53.0 (COOCH_3_), 52.5 (COOCH_3_), 17.3 (CH_3_); HREIMS *m*/*z* 434.1579 (calcd for C_25_H_24_NO_6_ (M+H)^+^, 434.1598).

Dimethyl 5-benzamido-3′,4′-dimethoxy-2-methyl-[1,1′-biphenyl]-3,4-dicarboxylate (**4Am**) [23]: white powder (EtOH) (mp lit. [23] 181–183 °C (MeOH)); mp 188.4–190.4 °C; IR ν_max_ 3308 (N–H), 2959 (H–C-sp^3^), 1731 (COO), 1671 (NHCO), 1571, 1508, 1489, 3308, 2959, 1731, 1671, 1571, 1508, 1489, cm^−1^; ^1^H NMR (CDCl_3_, 500 MHz) δ 11.47 (1H, s, NH), 8.85 (1H, s, H-6), 8.00 (2H, m, Ph), 7.53 (3H, s, Ph), 6.92 (2H, m, C_6_H_3_), 6.85 (1H, m, C_6_H_3_), 3.95 (3H, s), 3.94 (3H, s), 3.93 (3H, s), 3.90 (3H, s) (2 × OCH_3_ and 2 × COOCH_3_), 2.20 (3H, s, CH_3_); ^13^C NMR (CDCl_3_, 126 MHz) δ 169.7 (COO), 167.9 (COO), 165.5 (NHCO), 148.7, 148.6, 148.5, 138.4, 136.4, 134.6, 132.9, 132.1, 128.9, 127.9, 127.3, 123.3, 121.5, 112.9, 112.2, 111.0 (16 × C_Ar_), 56.0 (OCH_3_), 55.9 (OCH_3_), 53.0 (COOCH_3_), 52.5 (COOCH_3_), 17.3 (CH_3_); HREIMS *m*/*z* 464.1700 (calcd for C_26_H_26_NO_7_ (M+H)^+^, 464.1704).

Trimethyl 6-benzamido-3-methylbenzene-1,2,4-tricarboxylate (**4An**): pale yellow powder (EtOH); mp 164.6–167.9 °C; IR ν_max_ 3323 (N–H), 2948 (H–C-sp^3^), 1742 (COO), 1673 (NHCO), 1578, 1436, 1396 cm^–1^; ^1^H NMR (CDCl_3_, 500 MHz) δ 11.26 (1H, s, NH), 9.29 (1H, s, H-5), 8.00 (2H, m, Ph), 7.58 (1H, m, Ph), 7.53 (2H, m, Ph), 3.94 (6H, s, 2 × COOCH_3_), 3.93 (3H, s, COOCH_3_), 2.45 (3H, s, CH_3_); ^13^C NMR (CDCl_3_, 126 MHz) δ 168.8 (COO), 167.4 (COO), 167.0 (COO), 165.5 (NHCO), 138.2, 137.1, 136.1, 134.2, 132.3, 130.6, 128.9, 127.3, 123.3, 116.8 (10 × C_Ar_), 53.3 (COOCH_3_), 52.6 (COOCH_3_), 52.6 (COOCH_3_), 17.0 (CH_3_); HREIMS *m*/*z* 386.1225 (calcd for C_20_H_20_NO_7_ (M+H)^+^, 386.1234).

Dimethyl 6-benzamido-4-benzoyl-3-methylphthalate (**4Ao**) [25]: white powder (MeOH); mp 160.4–162.4 °C (mp lit. [25] 160–161 °C (EtOH)); IR ν_max_ 3359 (N–H), 2948 (H–C-sp^3^), 1708 (COO), 1692 (NHCO), 1666, 1598, 1577, 1516, 1492, 1436, 1399 cm^−1^; ^1^H NMR (CDCl_3_, 500 MHz) δ 11.39 (1H, s, NH), 8.88 (1H, s, H-5), 7.96 (2H, m, Ph), 7.86 (2H, m Ph), 7.62 (1H, m, Ph), 7.57 (1H, m, Ph), 7.50 (4H, m, Ph), 3.97 (3H, s, COOCH_3_), 3.94 (3H, s, COOCH_3_), 2.19 (3H, s, CH_3_); ^13^C NMR (CDCl_3_, 126 MHz) δ 196.6 (COPh), 168.8 (COO), 167.6 (COO), 165.5 (NHCO), 145.1, 138.4, 136.8, 136.1, 134.2, 134.2, 132.3, 130.2, 128.9, 128.9, 127.7, 127.3, 120.6, 115.4 (14 × C_Ar_), 53.3 (COOCH_3_), 52.6 (COOCH_3_), 16.4 (CH_3_); HREIMS *m*/*z* 432.1433 (calcd for C_25_H_22_NO_6_ (M+H)^+^, 432.1442).

Dimethyl 4-acetyl-6-benzamido-3-methylphthalate (**4Ap**) [23,25]: yellow powder (EtOH); mp 150.2–152.0 °C (mp lit. [23,25] 149–150 °C (EtOH)); IR ν_max_ 3335 (N–H), 2951 (H–C-sp^3^), 1738 (COO), 1679 (NHCO), 1579, 1432 cm^−1^; ^1^H NMR (CDCl_3_, 500 MHz) δ 11.40 (1H, s, NH), 9.20 (1H, s, H-5), 7.99 (2H, m, Ph), 7.59 (1H, m, Ph), 7.54 (2H, m, Ph), 3.94 (3H, s, COOCH_3_), 3.93 (3H, s, COOCH_3_), 2.64 (3H, s, COCH_3_), 2.33 (3H, s, CH_3_); ^13^C NMR (CDCl_3_, 126 MHz) δ 202.0 (COCH_3_), 168.9 (COO), 167.5 (COO), 165.7 (NHCO), 144.2, 138.7, 137.4, 134.2, 132.4, 129.0, 128.2, 127.3, 121.0, 115.8 (10 × C_Ar_), 53.3 (COOCH_3_), 52.6 (COOCH_3_), 30.4 (COCH_3_), 16.6 (CH_3_); HREIMS *m*/*z* 370.1284 (calcd for C_20_H_20_NO_6_ (M+H)^+^, 370.1285).

Dimethyl 4-acetyl-3-methyl-6-(4-nitrobenzamido)phthalate (**4Aq**) [23]: pale brown powder (xylene); mp 205.8–208.5 °C (mp lit. [23] 209–211 °C (MeOH)); IR ν_max_ 3262 (N–H), 2947 (H–C-sp^3^), 1740 (COO), 1695 (NHCO), 1603, 1579, 1518, 1487, 1434, 1397 cm^−1^; ^1^H NMR (CDCl_3_, 500 MHz) δ 11.70 (1H, s, NH), 9.15 (1H, s, H-5), 8.40 and 8.17 (2H each, AA’XX’, *J* = 8.9 Hz, C_6_H_4_NO_2_), 3.96 (3H, s, COOCH_3_), 3.94 (3H, s, COOCH_3_), 2.64 (3H, s, COCH_3_), 2.34 (3H, s, CH_3_); ^13^C NMR (CDCl_3_, 126 MHz) δ 201.8 (COCH_3_), 168.7 (COO), 167.6 (COO), 163.5 (NHCO), 150.0, 144.6, 139.7, 138.2, 137.7, 128.9, 128.5, 124.2, 120.7, 115.6 (10 × C_Ar_), 53.5 (COOCH_3_), 52.7 (COOCH_3_), 30.4 (COCH_3_), 16.6 (CH_3_); HREIMS *m*/*z* 415.1138 (calcd for C_20_H_19_N_2_O_8_ (M+H)^+^, 415.1136).

Dimethyl 4-acetyl-6-(4-methoxybenzamido)-3-methylphthalate (**4Ar**) [23]: yellow crystals (xylene); mp 139.3–141.2 °C (mp lit. [23] 139–141 °C (MeOH)); IR ν_max_ 3325 (N–H), 2953 (H–C-sp^3^), 2838, 1727 (COO), 1674 (NHCO), 1581, 1530, 1504, 1436, 1397 cm^−1^; ^1^H NMR (CDCl_3_, 500 MHz) δ 11.32 (1H, s, NH), 9.19 (1H, s, H-5), 7.96 and 7.02 (2H each, AA’XX’, *J* = 8.8 Hz, C_6_H_4_OCH_3_), 3.94 (3H, s, COOCH_3_), 3.92 (3H, s, COOCH_3_), 3.88 (3H, s, OCH_3_), 2.63 (3H, s, COCH_3_), 2.33 (3H, s, CH_3_); ^13^C NMR (CDCl_3_, 126 MHz) δ 202.0 (COCH_3_), 168.9 (COO), 167.5 (COO), 165.3 (NHCO), 162.9, 144.1, 138.9, 137.4, 129.2, 127.9, 126.4, 121.0, 115.5, 114.2 (10 × C_Ar_), 55.5 (OCH_3_), 53.3 (COOCH_3_), 52.5 (COOCH_3_), 30.3 (COCH_3_), 16.6 (CH_3_); HREIMS *m*/*z* 400.1388 (calcd for C_21_H_22_NO_7_ (M+H)^+^, 400.1391).

Dimethyl 4-acetyl-3-methyl-6-(3,4,5-trimethoxybenzamido)phthalate (**4As**): isolated by column chromatography (petroleum ether/EtOAc = 3:1 to 1:1), white powder (petroleum ether/Et_2_O); mp 87.9–92.7 °C; IR ν_max_ 3325 (N–H), 2951 (H–C-sp^3^), 2839, 1732 (COO), 1692 (NHCO), 1671, 1580, 1497, 1435, 1415, 1396 cm^−1^; ^1^H NMR (CDCl_3_, 500 MHz) δ 11.43 (1H, s, NH), 9.18 (1H, s, H-5), 7.26 (2H, s, C_6_*H*_2_(OCH_3_)_3_), 3.98 (6H, s, 2 × OCH_3_), 3.947 (3H, s), 3.945 (3H, s), 3.93 (3H, s) (2 × COOCH_3_ and OCH_3_), 2.64 (3H, s, COCH_3_), 2.34 (3H, s, CH_3_); ^13^C NMR (CDCl_3_, 126 MHz) δ 201.9 (COCH_3_), 168.8 (COO), 167.5 (COO), 165.3 (NHCO), 153.4, 144.2, 141.6, 138.7, 137.5, 129.4, 128.2, 120.7, 115.6, 104.6 (10 × C_Ar_), 61.0 (OCH_3_), 56.3 (OCH_3_), 53.3 (COOCH_3_), 52.6 (COOCH_3_), 30.3 (COCH_3_), 16.6 (CH_3_); HREIMS *m*/*z* 460.1601 (calcd for C_23_H_26_NO_9_ (M+H)^+^, 460.1602).

Dimethyl 6-acetamido-4-acetyl-3-methylphthalate (**4At**): isolated by two rounds of column chromatography (first column was petroleum ether/EtOAc = 3:1, second column was petroleum ether/EtOAc = 1:1), yellow waxy solid (petroleum ether/EtOAc); IR ν_max_ 3328 (N–H), 2955 (H–C-sp^3^), 1726 (COO), 1688 (NHCO), 1571, 1505, 1430, 1396 cm^−1^; ^1^H NMR (CDCl_3_, 500 MHz) δ 10.27 (1H, s, NHCOCH_3_), 8.92 (1H, s, H-5), 3.908 (3H, s, COOCH_3_), 3.906 (3H, s, COOCH_3_), 2.58 (3H, s, COCH_3_), 2.30 (3H, s, NHCOCH_3_), 2.23 (3H, s, CH_3_); ^13^C NMR (CDCl_3_, 126 MHz) δ 202.0 (COCH_3_), 169.1 (COO), 168.8 (COO), 167.0 (NHCO), 144.0, 138.0, 137.1, 128.0, 121.0, 115.9 (6 × C_Ar_), 53.1 (COOCH_3_), 52.5 (COOCH_3_), 30.3 (COCH_3_), 25.4 (COCH_3_), 16.5 (CH_3_); HREIMS *m*/*z* 308.1128 (calcd for C_15_H_18_NO_6_ (M+H)^+^, 308.1129).

Dimethyl 6-benzamido-3-ethyl-4-methylphthalate (**4Au**): isolated by column chromatography (petroleum ether/EtOAc = 3:1), white powder (petroleum ether/EtOAc); mp 95.4–99.9 °C; IR ν_max_ 3290 (N–H), 2943 (H–C-sp^3^), 1731 (COO), 1668 (NHCO), 1577, 1507, 1441, 1404 cm^–1^; ^1^H NMR (CDCl_3_, 500 MHz) δ 11.44 (1H, s, NH), 8.74 (1H, s, H-5), 8.00 (2H, m, Ph), 7.53 (3H, m, Ph), 3.91 (3H, s, COOCH_3_), 3.90 (3H, s, COOCH_3_), 2.58 (2H, q, *J* = 7.5 Hz, CH_2_CH_3_), 2.44 (3H, s, CH_3_), 1,16 (3H, t, *J* = 7.5 Hz, CH_2_CH_3_); ^13^C NMR (CDCl_3_, 126 MHz) δ 169.8 (COO), 168.0 (COO), 165.5 (NHCO), 143.9, 138.7, 135.3, 134.9, 134.7, 132.1, 128.9, 127.3, 123.8, 112.2 (10 × C_Ar_), 52.9 (COOCH_3_), 52.3 (COOCH_3_), 23.8 (CH_2_CH_3_), 20.2 (CH_2_CH_3_), 14.5 (CH_3_); HREIMS *m*/*z* 356.1487 (calcd for C_20_H_22_NO_5_ (M+H)^+^, 356.1492).

Dimethyl 5′-benzamido-[1,1′:2′,1′’-terphenyl]-3′,4′-dicarboxylate (**4Av**): off-white powder (EtOH); mp 122.3–125.7 °C; IR ν_max_ 3308 (N–H), 2949 (H–C-sp^3^), 1736 (COO), 1675 (NHCO), 1565, 1485, 1438 cm^−1^; ^1^H NMR (CDCl_3_, 500 MHz) δ 11.51 (1H, s, NH), 9.00 (1H, s, H-6), 8.04 (2H, m), 7.59 (1H, m) 7.54 (2H, m), 7.19 (3H, m), 7.16 (3H, m), 7.12 (2H, m), 7.07 (2H, m) (3 × Ph), 3.92 (3H, s, COOCH_3_), 3.48 (3H, s, COOCH_3_); ^13^C NMR (CDCl_3_, 126 MHz) δ 168.9 (COO), 168.0 (COO), 165.6 (NHCO), 147.4, 139.8, 139.7, 137.5, 136.8, 134.5, 133.8, 132.2, 130.3, 129.7, 128.9, 127.8, 127.6, 127.4, 127.3, 127.2, 123.7, 113.2 (18 × C_Ar_), 53.1 (COOCH_3_), 52.1 (COOCH_3_); HREIMS *m*/*z* 466.1635 (calcd for C_29_H_24_NO_5_ (M+H)^+^, 466.1649).

Dimethyl 4-benzamido-4′-fluoro-6-methyl-[1,1′-biphenyl]-2,3-dicarboxylate (**4Aw**): white powder (EtOH); mp 172.7–175.7 °C; IR ν_max_ 3255 (N–H), 2958 (H–C-sp^3^), 1718 (COO), 1678 (NHCO), 1579, 1489, 1432 cm^–1^; ^1^H NMR (CDCl_3_, 500 MHz) δ 11.58 (1H, s, NH), 8.86 (1H, s, H-5), 8.04 (2H, m, Ph), 7.59 (1H, m, Ph), 7.54 (2H, m, Ph), 7.15 (2H, m, C_6_H_4_F), 7.09 (2H, m, C_6_H_4_F), 3.88 (3H, s, COOCH_3_), 3.48 (3H, s, COOCH_3_), 2.16 (3H, s, CH_3_); ^13^C NMR (CDCl_3_, 126 MHz) δ 168.8 (COO), 167.9 (COO), 165.7 (NHCO), 162.2 (d, *J* = 247.0 Hz, C–4′), 144.1, 140.1, 136.3, 134.5, 134.0 (5 × C_Ar_), 133.5 (d, *J* = 3.5 Hz, C–1′), 132.2 (C_Ar_), 131.2 (d, *J* = 8.1 Hz, C–2′), 128.9, 127.4, 123.0 (3 × C_Ar_), 115.2 (d, *J* = 21.4 Hz, C–3′), 111.6 (C_Ar_), 52.9 (COOCH_3_), 52.0 (COOCH_3_), 21.6 (CH_3_); ^19^F NMR (CDCl_3_, 471 MHz) δ –114.3; HREIMS *m*/*z* 422.1390 (calcd for C_24_H_21_FNO_5_ (M+H)^+^, 422.1398).

6-Ethyl 2,3-dimethyl 4-benzamido-4′-methoxy-[1,1′-biphenyl]-2,3,6-tricarboxylate (**4Ax**): isolated by rounds of two column chromatography (first column was petroleum ether/EtOAc = 3:2, second column was petroleum ether/EtOAc = 3:1), white powder (Et_2_O); mp 165.7–169.2 °C; IR ν_max_ 3332 (N–H), 2955 (H–C-sp^3^), 1713 (COO), 1678 (NHCO), 1579, 1510, 1492, 1435 cm^−1^; ^1^H NMR (CDCl_3_, 500 MHz) δ 11.43 (1H, s, NH), 9.25 (1H, s, H-5), 8.02 (2H, m, Ph), 7.56 (3H, m, Ph), 7.15 and 6.89 (2H each, AA’XX’, *J* = 8.5 Hz, C_6_H_4_OCH_3_), 4.09 (2H, q, *J* = 7.1 Hz, COOCH_2_CH_3_), 3.92 (3H, s, COOCH_3_), 3.83 (3H, s, COOCH_3_), 3.52 (3H, s, OCH_3_), 1.07 (3H, t, *J* = 7.1 Hz, COOCH_2_CH_3_); ^13^C NMR (CDCl_3_, 126 MHz) δ 168.1 (COO), 167.5 (COO), 166.8 (COO), 165.6 (NHCO), 159.2, 139.6, 138.2, 137.4, 134.3, 133.8, 132.4, 130.4, 129.1, 129.0, 127.4, 121.9, 115.6, 113.1 (14 × C_Ar_), 61.6 (OCH_3_), 55.2 (CH_2_CH_3_), 53.3 (COOCH_3_), 52.2 (COOCH_3_), 13.9 (CH_2_CH_3_); HREIMS *m*/*z* 492.1645 (calcd for C_27_H_26_NO_8_ (M+H)^+^, 492.1653).

Trimethyl 6-benzamido-3-(2-methoxy-2-oxoethyl)benzene-1,2,4-tricarboxylate (**4Ay**) [25]: off-white powder (EtOH); mp 148.6–150.1 °C (mp lit. [25] 143.5–145.8 °C (MeOH)); IR ν_max_ 3320 (N–H), 2954 (H–C-sp^3^), 1735 (COO), 1683 (NHCO), 1580, 1524, 1428, 1402 cm^−1^; ^1^H NMR (CDCl_3_, 500 MHz) δ 11.20 (1H, s, NH), 9.47 (1H, s, H-5), 7.99 (2H, m, Ph), 7.60 (1H, m, Ph), 7.54 (2H, m, Ph), 4.06 (2H, s, CH_2_), 3.94 (3H, s, COOCH_3_), 3.93 (3H, s, COOCH_3_), 3.91 (3H, s, COOCH_3_), 3.69 (3H, s, COOCH_3_); ^13^C NMR (CDCl_3_, 126 MHz) δ 170.8 (COO), 168.2 (COO), 167.3 (COO), 166.5 (COO), 165.5 (NHCO), 139.3, 137.2, 135.4, 134.0, 132.5, 129.0, 127.6, 127.3, 124.4, 117.9 (10 × C_Ar_), 53.4 (COOCH_3_), 52.8 (COOCH_3_), 52.8 (COOCH_3_), 52.2 (COOCH_3_), 35.6 (CH_2_); HREIMS *m*/*z* 444.1287 (calcd for C_22_H_22_NO_9_ (M+H)^+^, 444.1289).

Trimethyl 5-benzamido-4′-methoxy-[1,1′-biphenyl]-2,3,4-tricarboxylate (**4Az**): pale yellow powder (EtOH); mp 154.3–157.5 °C; IR ν_max_ 3321 (N–H), 2949 (H–C-sp^3^), 1721 (COO), 1681 (NHCO), 1569, 1506, 1488, 1432 cm^−1^; ^1^H NMR (CDCl_3_, 500 MHz) δ 11.43 (1H, s, NH), 9.29 (1H, s, H-6), 8.02 (2H, m, Ph), 7.56 (3H, m, Ph), 7.14 and 6.89 (2H each, AA’XX’, *J* = 8.7 Hz, C_6_H_4_OCH_3_), 3.92 (3H, s, COOCH_3_), 3.84 (3H, s, COOCH_3_), 3.67 (3H, s), 3.52 (3H, s) (OCH_3_ and COOCH_3_); ^13^C NMR (CDCl_3_, 126 MHz) δ 168.1 (COO), 167.4 (COO), 167.0 (COO), 165.6 (NHCO), 159.2, 139.6, 137.5, 137.4, 134.2, 134.1, 132.4, 130.3, 129.0, 128.9, 127.4, 122.1, 115.9, 113.1 (14 × C_Ar_), 55.2 (OCH_3_), 53.3 (COOCH_3_), 52.5 (COOCH_3_), 52.2 (COOCH_3_); HREIMS *m*/*z* 478.1484 (calcd for C_26_H_24_NO_8_ (M+H)^+^, 478.1496).

Dimethyl 6-benzamido-2,3-dihydro-1*H*-indene-4,5-dicarboxylate (**4Aaa**): white powder (EtOH); mp 107.7–108.9 °C; IR ν_max_ 3250 (N–H), 2948 (H–C-sp^3^), 1737 (COO), 1667 (NHCO), 1591, 1523, 1434 cm^–1^; ^1^H NMR (CDCl_3_, 500 MHz) δ 11.20 (1H, s, NH), 8.73 (1H, s, H-7), 8.00 (2H, m, Ph), 7.53 (3H, m, Ph), 3.895 (3H, s, COOCH_3_), 3.889 (3H, s, COOCH_3_), 3.01 (2H, t, *J* = 7.5 Hz), 2.94 (2H, t, *J* = 7.5 Hz), 2.12 (2H, deg. dt, *J* = 7.5 Hz) (3 × CH_2_); ^13^C NMR (CDCl_3_, 126 MHz) δ 169.0 (COO), 168.6 (COO), 165.5 (NHCO), 151.6, 139.0, 138.0, 134.6, 132.1, 130.8, 128.9, 127.3, 118.7, 113.5 (10 × C_Ar_), 52.8 (COOCH_3_), 52.4 (COOCH_3_), 33.6, 31.5, 25.0 (3 × CH_2_); HREIMS *m*/*z* 354.1337 (calcd for C_20_H_20_NO_5_ (M+H)^+^, 354.1336).

Dimethyl 3-benzamido-5,6,7,8-tetrahydronaphthalene-1,2-dicarboxylate (**4Aab**): off-white crystals (EtOH); mp 131.8–133.6 °C; IR ν_max_ 3250 (N–H), 2945 (H–C-sp^3^), 1727 (COO), 1671 (NHCO), 1578, 1509, 1411 cm^−1^; ^1^H NMR (CDCl_3_, 500 MHz) δ 11.44 (1H, s, NH), 8.66 (1H, s, H-4), 8.00 (2H, m, Ph), 7.53 (3H, m, Ph), 3.90 (6H, s, 2 × COOCH_3_), 2.88 (2H, m), 2.67 (2H, m), 1.79 (4H, m) (4 × CH_2_); ^13^C NMR (CDCl_3_, 126 MHz) δ 169.5 (COO), 168.1 (COO), 165.5 (NHCO), 145.0, 138.1, 135.7, 134.7, 132.0, 129.5, 128.8, 127.3, 122.4, 111.9 (10 × C_Ar_), 52.9 (COOCH_3_), 52.3 (COOCH_3_), 30.5, 26.2, 22.7, 22.2 (4 × CH_2_); HREIMS *m*/*z* 368.1491 (calcd for C_21_H_22_NO_5_ (M+H)^+^, 368.1492).

Dimethyl 3-benzamido-5,6,7,8,9,10-hexahydrobenzo [8]annulene-1,2-dicarboxylate (**4Aac**) [23]: white crystals (EtOH); mp 130.3–133.1 °C (mp lit. [23] 128–129 °C (MeOH)); IR ν_max_ 3261 (N–H), 2926 (H–C-sp^3^), 1709 (COO), 1670 (NHCO), 1581, 1518, 1442 cm^−1^; ^1^H NMR (CDCl_3_, 500 MHz) δ 11.51 (1H, s, NH), 8.75 (1H, s, H-4), 8.00 (2H, m, Ph), 7.54 (3H, m, Ph), 3.91 (3H, s, COOCH_3_), 3.90 (3H, s, COOCH_3_), 2.86 (2H, m), 2.73 (2H, m), 1.77 (2H, m), 1.71 (2H, m) (4 × CH_2_), 1.38 (4H, m, 2 × CH_2_); ^13^C NMR (CDCl_3_, 126 MHz) δ 169.9 (COO), 168.1 (COO), 165.5 (NHCO), 149.8, 139.1, 135.3, 134.8, 133.3, 132.0, 128.9, 127.3, 122.6, 112.3 (10 × C_Ar_), 52.9 (COOCH_3_), 52.3 (COOCH_3_), 33.4, 31.9, 31.1, 28.4, 26.2, 25.8 (6 × CH_2_); HREIMS *m*/*z* 396.1802 (calcd for C_23_H_26_NO_5_ (M+H)^+^, 396.1805).

Dimethyl 2-benzamido-9*H*-fluorene-3,4-dicarboxylate (**4Aad**): pale yellow powder (EtOH); mp 189.5–192.5 °C; IR ν_max_ 3321 (N–H), 2949 (H–C-sp^3^), 1736, 1716 (COO), 1684 (NHCO), 1580, 1514, 1490, 1433, 1403 cm^−1^; ^1^H NMR (CDCl_3_, 500 MHz) δ 11.73 (1H, s, NH), 9.17 (1H, s, H-1), 8.04 (2H, m, Ph), 7.56 (5H, m), 7.35 (2H, m) (Ph, H-5, H-6, H-7, H-8), 4.06 (3H, s, COOCH_3_), 4.02 (2H, s, CH_2_), 3.98 (3H, s, COOCH_3_); ^13^C NMR (CDCl_3_, 126 MHz) δ 169.1 (COO), 168.3 (COO), 165.7 (NHCO), 150.6, 143.5, 139.7, 138.6, 134.6, 133.4, 132.2, 128.9, 127.6, 127.4, 127.2, 125.1, 121.5, 118.6, 112.7 (15 × C_Ar_), 53.1 (COOCH_3_), 52.7 (COOCH_3_), 37.4 (CH_2_) (one aromatic signal is hidden); HREIMS *m*/*z* 402.1327 (calcd for C_24_H_20_NO_5_ (M+H)^+^, 402.1336).

Dimethyl 4-benzamido-5-methyl-[1,1′-biphenyl]-2,3-dicarboxylate (**4Aae**): white powder (EtOH); mp 177.0–180.7 °C; IR ν_max_ 3274 (N–H), 2944 (H–C-sp^3^), 1740, 1720 (COO), 1707 (NHCO), 1637, 1513, 1484, 1432 cm^−1^; ^1^H NMR (CDCl_3_, 500 MHz) δ 9.20 (1H, s, NH), 7.97 (2H, m, Ph), 7.58 (1H, m, Ph), 7.51 (2H, m, Ph), 7.43–7.35 (4H, m, H-6 and Ph), 7.32 (2H, m, Ph), 3.80 (3H, s, COOCH_3_), 3.56 (3H, s, COOCH_3_), 2.39 (3H, s, CH_3_); ^13^C NMR (CDCl_3_, 126 MHz) δ 168.7 (COO), 167.7 (COO), 165.4 (NHCO), 139.5, 138.7, 138.3, 135.7, 134.2, 133.8, 132.2, 131.2, 128.9, 128.3, 128.3, 127.8, 127.5 (13 × C_Ar_), 53.0 (COOCH_3_), 52.3 (COOCH_3_), 19.4 (CH_3_) (one aromatic signal is hidden); HREIMS *m*/*z* 404.1485 (calcd for C_24_H_22_NO_5_ (M+H)^+^, 404.1492).

Dimethyl 3-benzamido-4-methyl-6-(thiophen-2-yl)phthalate (**4Aaf**): isolated by column chromatography (petroleum ether/EtOAc = 3:1), white crystals (petroleum ether/EtOAc); mp 193.5–196.2 °C; IR ν_max_ 3235 (N–H), 3105, 2946 (H–C-sp^3^), 1715 (COO), 1643 (NHCO), 1602, 1508, 1486, 1430 cm^−1^; ^1^H NMR (CDCl_3_, 500 MHz) δ 9.28 (1H, s, NH), 7.97 (2H, m, Ph), 7.59 (1H, m, Ph), 7.52 (3H, m, Ph, H-5), 7.36 (1H, m, C_4_H_3_S), 7.06 (2H, m, C_4_H_3_S), 3.82 (3H, s, COOCH_3_), 3.69 (3H, s, COOCH_3_), 2.38 (3H, s, CH_3_); ^13^C NMR (CDCl_3_, 126 MHz) δ 168.5 (COO), 167.3 (COO), 165.3 (NHCO), 140.1, 138.1, 136.2, 135.0, 133.7, 132.2, 131.7, 130.8, 128.9, 127.5, 127.4, 126.8, 126.5, 125.5 (10 × C_Ar_, 4 × C_Thioph_), 53.0 (COOCH_3_), 52.5 (COOCH_3_), 19.4 (CH_3_); HREIMS *m*/*z* 410.1052 (calcd for C_22_H_20_NO_5_S (M+H)^+^, 410.1057).

Diethyl 3-benzamido-6-methylphthalate (**4Ba**) [24]: isolated by column chromatography (petroleum ether/EtOAc = 3:1), yellow waxy solid (petroleum ether/EtOAc) (mp lit. [24] 54–57 °C (petroleum ether:AcOEt)); IR ν_max_ 3320 (N–H), 2981 (H–C-sp^3^), 1728 (COO), 1676 (NHCO), 1519, 1492 cm^−1^; ^1^H NMR (CDCl_3_, 500 MHz) δ 11.34 (1H, s, NH), 8.77 (1H, d, *J* = 8.7 Hz, H-4 or H-5), 8.00 (2H, m, Ph), 7.53 (3H, m, Ph), 7.42 (1H, d, *J* = 8.7 Hz, H-4 or H-5), 4.38 (4H, q, *J* = 7.2 Hz, 2 × COOCH_2_CH_3_), 2.34 (3H, s, CH_3_), 1.40 (3H, t, *J* = 7.2 Hz, COOCH_2_CH_3_), 1.37 (3H, t, *J* = 7.2 Hz, COOCH_2_CH_3_); ^13^C NMR (CDCl_3_, 126 MHz) δ 168.7 (COO), 167.8 (COO), 165.5 (NHCO), 138.4, 135.5, 135.3, 134.6, 132.0, 130.2, 128.8, 127.3, 122.1, 114.9 (10 × C_Ar_), 62.3 (CH_2_CH_3_), 61.4 (CH_2_CH_3_), 19.1 (CH_3_), 14.2 (CH_2_CH_3_), 13.8 (CH_2_CH_3_); HREIMS *m*/*z* 356.1492 (calcd for C_20_H_22_NO_5_ (M+H)^+^, 356.1492).

Diethyl 4-benzamido-[1,1′-biphenyl]-2,3-dicarboxylate (**4Bb**): off-white powder (EtOH); mp 112.2–113.4 °C; IR ν_max_ 3255 (N–H), 2978 (H–C-sp^3^), 1731 (COO), 1690 (NHCO), 1665, 1518, 1490 cm^−1^; ^1^H NMR (CDCl_3_, 500 MHz) δ 11.34 (1H, s, NH), 8.90 (1H, d, *J* = 8.7 Hz, H-5 or H-6), 8.02 (2H, m, Ph), 7.55 (4H, m, H-5 or H-6, Ph), 7.38 (5H, m, Ph), 4.38 (2H, q, *J* = 7.2 Hz, COOCH_2_CH_3_), 4.02 (2H, q, *J* = 7.2 Hz, COOCH_2_CH_3_), 1.34 (3H, t, *J* = 7.2 Hz, COOCH_2_CH_3_), 0.93 (3H, t, *J* = 7.2 Hz, COOCH_2_CH_3_); ^13^C NMR (CDCl_3_, 126 MHz) δ 168.4 (COO), 167.8 (COO), 165.6 (NHCO), 139.41, 139.36, 135.5, 135.1, 135.0, 134.5, 132.2, 128.9, 128.6, 128.2, 127.7, 127.4, 122.0, 115.3 (14 × C_Ar_), 62.5 (CH_2_CH_3_), 61.3 (CH_2_CH_3_), 13.7 (CH_2_CH_3_), 13.5 (CH_2_CH_3_); HREIMS *m*/*z* 418.1639 (calcd for C_25_H_24_NO_5_ (M+H)^+^, 418.1649).

Diethyl 4-benzamido-4′-methyl-[1,1′-biphenyl]-2,3-dicarboxylate (**4Bc**): yellow-brown powder (EtOH); mp 125.8–128.7 °C; IR ν_max_ 3335 (N–H), 2985 (H–C-sp^3^), 1709 (COO), 1681 (NHCO), 1584, 1508, 1489, 1364 cm^–1^; ^1^H NMR (CDCl_3_, 500 MHz) δ 11.32 (1H, s, NH), 8.88 (1H, d, *J* = 8.7 Hz, H-5 or H-6), 8.02 (2H, m, Ph), 7.55 (4H, m, Ph and H-5 or H-6), 7.21 (4H, m, C_6_H_4_), 4.38 (2H, q, *J* = 7.1 Hz, CH_2_), 4.04 (2H, q, *J* = 7.1 Hz, CH_2_), 2.39 (3H, s, CH_3_), 1.34 (3H, t, *J* = 7.1 Hz, CH_3_), 0.98 (3H, t, *J* = 7.1 Hz, CH_3_); ^13^C NMR (CDCl_3_, 126 MHz) δ 168.5 (COO), 167.8 (COO), 165.6 (NHCO), 139.2, 137.4, 136.5, 135.6, 135.1, 135.0, 134.5, 132.2, 128.9, 128.9, 128.5, 127.4, 122.0, 115.3 (14 × C_Ar_), 62.5 (CH_2_CH_3_), 61.3 (CH_2_CH_3_), 21.2 (CH_3_), 13.7 (CH_2_CH_3_), 13.6 (CH_2_CH_3_); HREIMS *m*/*z* 432.1789 (calcd for C_26_H_26_NO_5_ (M+H)^+^, 432.1805).

Diethyl 3-benzamido-6-(furan-2-yl)phthalate (**4Bd**): isolated by column chromatography (petroleum ether/EtOAc = 3:1), pale yellow powder (EtOH); mp 128.6–130.6 °C; IR ν_max_ 3255 (N–H), 2984 (H–C-sp^3^), 1727 (COO), 1674 (NHCO), 1575, 1523, 1484 cm^−1^; ^1^H NMR (CDCl_3_, 500 MHz) δ 11.41 (1H, s, NH), 8.92 (1H, d, *J* = 8.9 Hz, H-4 or H-5), 8.00 (2H, m, Ph), 7.84 (1H, d, *J* = 8.9 Hz, H-4 or H-5), 7.58 (1H, m, Ph), 7.53 (2H, m, Ph), 7.49 (1H, m, C_4_H_3_O), 6.56 (1H, m, C_4_H_3_O), 6.48 (1H, m, C_4_H_3_O), 4.41 (2H, q, *J* = 7.2 Hz, COOCH_2_CH_3_), 4.32 (2H, q, *J* = 7.2 Hz, COOCH_2_CH_3_), 1.38 (3H, t, *J* = 7.2 Hz, COOCH_2_CH_3_), 1.28 (3H, t, *J* = 7.2 Hz, COOCH_2_CH_3_); ^13^C NMR (CDCl_3_, 126 MHz) δ 168.5 (COO), 167.6 (COO), 165.6 (NHCO), 150.9, 142.8, 139.8, 134.5, 132.8, 132.6, 132.2, 128.9, 127.4, 124.1, 122.1, 115.4, 111.7, 108.3 (10 × C_Ar_, 4 × C_Fur_), 62.6 (CH_2_CH_3_), 61.7 (CH_2_CH_3_), 14.0 (CH_2_CH_3_), 13.8 (CH_2_CH_3_); HREIMS *m*/*z* 408.1437 (calcd for C_23_H_22_NO_6_ (M+H)^+^, 408.1442).

Diethyl 3-benzamido-6-(thiophen-2-yl)phthalate (**4Be**): orange-brown crystals (EtOH); mp 122.8–124.8 °C; IR ν_max_ 3313 (N–H), 3114, 2979 (H–C-sp^3^), 1727 (COO), 1673 (NHCO), 1581, 1535, 1513, 1490, 1365 cm^−1^; ^1^H NMR (CDCl_3_, 500 MHz) δ 11.49 (1H, s, NH), 8.92 (1H, d, *J* = 8.8 Hz, H-4 or H-5), 8.02 (2H, m, Ph), 7.66 (1H, d, *J* = 8.8 Hz, H-4 or H-5), 7.58 (1H, m, Ph), 7.53 (2H, m, Ph), 7.36 (1H, m), 7.05 (2H, m) (C_4_H_3_S), 4.40 (2H, q, *J* = 7.2 Hz, COOC*H*_2_CH_3_), 4.14 (2H, q, *J* = 7.2 Hz, COOCH_2_CH_3_), 1.36 (3H, t, *J* = 7.2 Hz, COOCH_2_CH_3_), 1.09 (3H, t, *J* = 7.2 Hz, COOCH_2_CH_3_); ^13^C NMR (CDCl_3_, 126 MHz) δ 168.2 (COO), 167.5 (COO), 165.6 (NHCO), 140.2, 139.9, 135.9, 135.7, 134.4, 132.3, 128.9, 127.8, 127.4, 127.22, 127.15, 126.3, 121.8, 114.9 (10 × C_Ar_, 4 × C_Thioph_), 62.6 (CH_2_CH_3_), 61.5 (CH_2_CH_3_), 13.73 (CH_2_CH_3_), 13.68 (CH_2_CH_3_); HREIMS *m/z* 424.1203 (calcd for C_23_H_22_NO_5_S (M+H)^+^, 424.1213).

Diethyl 5-benzamido-4′-methoxy-2-methyl-[1,1′-biphenyl]-3,4-dicarboxylate (**4Bf**) [24]: pale yellow powder (EtOH); mp 109.6–112.0 °C (mp lit. [24] 116.0–116.8 °C (MeOH)); IR ν_max_ 3340 (N–H), 2977 (H–C-sp^3^), 1720 (COO), 1701, 1668 (NHCO), 1508, 1365 cm^−1^; ^1^H NMR (CDCl_3_, 500 MHz) δ 11.52 (1H, s, NH), 8.82 (1H, s, H-6), 8.00 (2H, m, Ph), 7.53 (3H, m, Ph), 7.28 and 6.96 (2H each, AA’XX’, *J* = 8.7 Hz, C_6_H_4_OCH_3_), 4.41 (2H, q, *J* = 7.2 Hz, COOCH_2_CH_3_), 4.40 (2H, q, *J* = 7.1 Hz, COOCH_2_CH_3_), 3.86 (3H, s, OCH_3_), 2.21 (3H, s, CH_3_), 1.42 (3H, t, *J* = 7.0 Hz, COOCH_2_CH_3_), 1.39 (3H, t, *J* = 7.0 Hz, COOCH_2_CH_3_); ^13^C NMR (CDCl_3_, 126 MHz) δ 169.2 (COO), 167.7 (COO), 165.5 (NHCO), 159.2, 148.2, 138.4, 136.5, 134.7, 132.7, 132.0, 130.3, 128.8, 127.8, 127.3, 123.3, 113.7, 113.0 (14 × C_Ar_), 62.3 (CH_2_CH_3_), 61.4 (CH_2_CH_3_), 55.4 (OCH_3_), 17.2 (CH_3_), 14.2 (CH_2_CH_3_), 13.9 (CH_2_CH_3_); HREIMS *m*/*z* 462.1899 (calcd for C_27_H_28_NO_6_ (M+H)^+^, 462.1911).

Diethyl 5-benzamido-3′,4′-dimethoxy-2-methyl-[1,1′-biphenyl]-3,4-dicarboxylate (**4Bg**) [24]: pale yellow powder (EtOH); mp 114.2–116.4 °C (mp lit. [24] 109.0–111.5 °C (Et_2_O)); IR ν_max_ 3330 (N–H), 2982 (H–C-sp^3^), 1725 (COO), 1676 (NHCO), 1508, 1366 cm^−1^; ^1^H NMR (CDCl_3_, 500 MHz) δ 11.52 (1H, s, NH), 8.82 (1H, s, H-6), 8.00 (2H, m, Ph), 7.53 (3H, m, Ph), 6.92 (2H, m, C_6_H_3_(OCH_3_)_2_), 6.84 (1H, m, C_6_H_3_(OCH_3_)_2_), 4.42 (2H, q, *J* = 7.1 Hz, COOCH_2_CH_3_), 4.40 (2H, q, *J* = 7.1 Hz, COOCH_2_CH_3_), 3.94 (3H, s, OCH_3_), 3.90 (3H, s, OCH_3_), 2.21 (3H, s, CH_3_), 1.41 (3H, t, *J* = 7.1 Hz, COOCH_2_CH_3_), 1.40 (3H, t, *J* = 7.1 Hz, COOCH_2_CH_3_); ^13^C NMR (CDCl_3_, 126 MHz) δ 169.2 (COO), 167.7 (COO), 165.5 (NHCO), 148.7, 148.6, 148.3, 138.4, 136.5, 134.7, 133.0, 132.1, 128.9, 127.8, 127.3, 123.3, 121.5, 113.1, 112.2, 111.0 (16 × C_Ar_), 62.3 (CH_2_CH_3_), 61.4 (CH_2_CH_3_), 56.00 (OCH_3_), 55.98 (OCH_3_), 17.3 (CH_3_), 14.2 (CH_2_CH_3_), 13.9 (CH_2_CH_3_); HREIMS *m*/*z* 492.2004 (calcd for C_28_H_30_NO_7_ (M+H)^+^, 492.2017).

1,2-Diethyl 4-methyl 6-benzamido-3-methylbenzene-1,2,4-tricarboxylate (**4Bh**): off-white crystals (EtOH); mp 134.6–137.9 °C; IR ν_max_ 3309 (N–H), 2981 (H–C-sp^3^), 1728 (COO), 1679 (NHCO), 1577, 1519, 1436 cm^−1^; ^1^H NMR (CDCl_3_, 500 MHz) δ 11.32 (1H, s, NH), 9.28 (1H, s, H-5), 8.00 (2H, m, Ph), 7.54 (3H, m, Ph), 4.40 (2H, q, *J* = 7.3 Hz, COOCH_2_CH_3_), 4.39 (2H, q, *J* = 7.1 Hz, COOCH_2_CH_3_), 3.94 (3H, s, COOCH_3_), 2.47 (3H, s, CH_3_), 1.40 (3H, t, *J* = 7.3 Hz, COOCH_2_CH_3_), 1.38 (3H, t, *J* = 7.1 Hz, COOCH_2_CH_3_); ^13^C NMR (CDCl_3_, 126 MHz) δ 168.3 (COO), 167.1 (COO), 167.1 (COO), 165.5 (NHCO), 138.1, 137.3, 136.0, 134.3, 132.3, 130.5, 128.9, 127.3, 123.2, 117.0 (10 × C_Ar_), 62.8 (CH_2_CH_3_), 61.6 (CH_2_CH_3_), 52.6 (COOCH_3_), 16.9 (CH_3_), 14.1 (CH_2_CH_3_), 13.8 (CH_2_CH_3_); HREIMS *m*/*z* 414.1537 (calcd for C_22_H_24_NO_7_ (M+H)^+^, 414.1547).

Diethyl 4-acetyl-6-benzamido-3-methylphthalate (**4Bi**) [25]: canary-yellow powder (EtOH); mp 91.9–93.9 °C (mp lit. [25] 97–99 °C (Et_2_O)); IR ν_max_ 3259 (N–H), 2982 (H–C-sp^3^), 1731 (COO), 1683 (NHCO), 1578, 1517, 1491 cm^−1^; ^1^H NMR (CDCl_3_, 500 MHz) δ 11.46 (1H, s, NH), 9.19 (1H, s, H-5), 8.00 (2H, m, Ph), 7.55 (3H, m, Ph), 4.41 (2H, q, *J* = 7.3 Hz, COOCH_2_CH_3_), 4.38 (2H, q, *J* = 7.2 Hz, COOCH_2_CH_3_), 2.64 (3H, s, COCH_3_), 2.35 (3H, s, CH_3_), 1.41 (3H, t, *J* = 6.9 Hz, COOCH_2_CH_3_), 1.38 (3H, t, *J* = 6.8 Hz, COOCH_2_CH_3_); ^13^C NMR (CDCl_3_, 126 MHz) δ 202.1 (COCH_3_), 168.4 (COO), 167.1 (COO), 165.7 (NHCO), 144.1, 138.6, 137.6, 134.3, 132.3, 129.0, 128.1, 127.3, 120.9, 116.0 (10 × C_Ar_), 62.8 (CH_2_CH_3_), 61.6 (CH_2_CH_3_), 30.4 (COCH_3_), 16.5 (CH_3_), 14.1 (CH_2_CH_3_), 13.8 (CH_2_CH_3_); HREIMS *m*/*z* 398.1585 (calcd for C_22_H_24_NO_6_ (M+H)^+^, 398.1585).

Diethyl 4-acetyl-3-methyl-6-(4-nitrobenzamido)phthalate (**4Bj**): off-white powder (EtOH); mp 166.1–167.6 °C; IR ν_max_ 3332 (N–H), 3108, 2983 (H–C-sp^3^), 1727 (COO), 1699 (NHCO), 1676, 1582, 1520, 1489 cm^–1^; ^1^H NMR (CDCl_3_, 500 MHz) δ 11.77 (1H, s, NH), 9.13 (1H, s, H-5), 8.38 and 8.16 (2H each, AA’XX’, *J* = 8.5 Hz, C_6_H_4_NO_2_), 4.43 (2H, q, *J* = 7.2 Hz, COOCH_2_CH_3_), 4.41 (2H, q, *J* = 7.2 Hz, COOCH_2_CH_3_), 2.64 (3H, s, COCH_3_), 2.35 (3H, s, CH_3_), 1.413 (3H, t, *J* = 7.2 Hz, COOCH_2_CH_3_), 1.406 (3H, t, *J* = 7.2 Hz, COOCH_2_CH_3_); ^13^C NMR (CDCl_3_, 126 MHz) δ 201.9 (COCH_3_), 168.1 (COO), 167.3 (COO), 163.5 (NHCO), 150.0, 144.5, 139.7, 138.2, 137.9, 128.8, 128.5, 124.1, 120.5, 115.7 (10 × C_Ar_), 63.0 (CH_2_CH_3_), 61.7 (CH_2_CH_3_), 30.4 (COCH_3_), 16.5 (CH_3_), 14.1 (CH_2_CH_3_), 13.8 (CH_2_CH_3_); HREIMS *m*/*z* 460.1704 (calcd for C_22_H_26_N_3_O_8_ (M+NH_4_)^+^, 460.1714).

Diethyl 6-benzamido-3-ethyl-4-methylphthalate (**4Bk**): isolated by two rounds of column chromatography (petroleum ether/EtOAc = 3:1), white powder (petroleum ether); mp 105.0–107.3 °C; IR ν_max_ 3307 (N–H), 2973 (H–C-sp^3^), 1734 (COO), 1671 (NHCO), 1580, 1519, 1492, 1468, 1409 cm^−1^; ^1^H NMR (CDCl_3_, 500 MHz) δ 11.51 (1H, s, NH), 8.73 (1H, s, H-5), 8.00 (2H, m, Ph), 7.53 (3H, m, Ph), 4.38 (2H, q, *J* = 7.2 Hz, COOCH_2_CH_3_), 4.37 (2H, q, *J* = 7.2 Hz, COOCH_2_CH_3_), 2.60 (2H, q, *J* = 7.4 Hz, CH_2_CH_3_), 2.44 (3H, s, CH_3_), 1.40 (6H, t, *J* = 7.2 Hz, COOCH_2_CH_3_), 1.37 (3H, t, *J* = 7.4 Hz, CH_2_CH_3_); ^13^C NMR (CDCl_3_, 126 MHz) δ 169.2 (COO), 167.8 (COO), 165.5 (NHCO), 143.7, 138.7, 135.5, 134.8, 134.8, 132.0, 128.8, 127.3, 123.7, 112.3 (10 × C_Ar_), 62.2 (CH_2_CH_3_), 61.3 (CH_2_CH_3_), 23.7, 20.2, 14.5, 14.2, 13.9 (4 × CH_3_, CH_2_); HREIMS *m*/*z* 384.1802 (calcd for C_22_H_26_NO_5_ (M+H)^+^, 384.1805).

1,2-Diethyl 4-methyl 6-benzamido-3-(2-methoxy-2-oxoethyl)benzene-1,2,4-tricarboxylate (**4Bl**) [25]: white crystals (EtOH); mp 119.4–121.2 °C (mp lit. [25] 113.1–114.6 °C (MeOH/H_2_O)); IR ν_max_ 3257 (N–H), 2992 (H–C-sp^3^), 2953, 1730 (COO), 1681 (NHCO), 1579, 1521, 1491 cm^−1^; ^1^H NMR (CDCl_3_, 500 MHz) δ 11.28 (1H, s, NH), 9.47 (1H, s, H-5), 8.00 (2H, m, Ph), 7.59 (1H, m, Ph), 7.53 (2H, m, Ph), 4.40 (2H, q, *J* = 7.2 Hz, COOCH_2_CH_3_), 4.37 (2H, q, *J* = 7.2 Hz, COOCH_2_CH_3_), 4.07 (2H, s, CH_2_COOCH_3_), 3.93 (3H, s, COOCH_3_), 3.69 (3H, s, COOCH_3_), 1.38 (3H, t, *J* = 7.2 Hz, COOCH_2_CH_3_), 1.37 (3H, t, *J* = 7.2 Hz, COOCH_2_CH_3_); ^13^C NMR (CDCl_3_, 126 MHz) δ 170.8, 167.7, 167.0, 166.5, 165.5 (5 × CO), 139.3, 137.5, 135.2, 134.1, 132.4, 129.0, 127.5, 127.3, 124.2, 118.0 (10 × C_Ar_), 62.9 (CH_2_CH_3_), 61.9 (CH_2_CH_3_), 52.8 (COOCH_3_), 52.1 (COOCH_3_), 35.6 (CH_2_), 14.0 (CH_2_CH_3_), 13.8 (CH_2_CH_3_); HREIMS *m/z* 472.1592 (calcd for C_24_H_26_NO_9_ (M+H)^+^, 472.1602).

Diethyl 6-benzamido-2,3-dihydro-1*H*-indene-4,5-dicarboxylate (**4Bm**): isolated by column chromatography (petroleum ether/EtOAc = 3:1), white waxy solid (petroleum ether/EtOAc); IR ν_max_ 3255 (N–H), 2957 (H–C-sp^3^), 1721 (COO), 1641, 1516, 1488 cm^−1^; ^1^H NMR (CDCl_3_, 500 MHz) δ 11.29 (1H, s, NH), 8.73 (1H, s, H-7), 8.00 (2H, m, Ph), 7.53 (3H, m, Ph), 4.36 (2H, q, *J* = 7.2 Hz, COOCH_2_CH_3_), 4.35 (2H, q, *J* = 7.2 Hz, COOCH_2_CH_3_), 3.01 (2H, t, *J* = 7.5 Hz, CH_2_), 2.95 (2H, t, *J* = 7.5 Hz, CH_2_), 2.12 (2H, deg. tt, *J* = 7.5 Hz, CH_2_), 1.39 (3H, t, *J* = 7.2 Hz, COOCH_2_CH_3_), 1.35 (3H, t, *J* = 7.2 Hz, COOCH_2_CH_3_); ^13^C NMR (CDCl_3_, 126 MHz) δ 168.5 (COO), 168.2 (COO), 165.5 (NHCO), 151.3, 139.1, 137.7, 134.7, 132.0, 131.2, 128.8, 127.3, 118.5, 113.5 (10 × C_Ar_), 62.1 (CH_2_CH_3_), 61.3 (CH_2_CH_3_), 33.6, 31.5, 25.0 (3 × CH_2_), 14.3 (CH_2_CH_3_), 13.8 (CH_2_CH_3_); HREIMS *m*/*z* 382.1642 (calcd for C_22_H_24_NO_5_ (M+H)^+^, 382.1649).

Diethyl 3-benzamido-5,6,7,8-tetrahydronaphthalene-1,2-dicarboxylate (**4Bn**): isolated by column chromatography (petroleum ether/EtOAc = 3:1), white waxy solid (petroleum ether/EtOAc); IR ν_max_ 3252 (N–H), 2941 (H–C-sp^3^), 1723 (COO), 1644, 1517 1488 cm^−1^; ^1^H NMR (CDCl_3_, 500 MHz) δ 11.51 (1H, s, NH), 8.65 (1H, s, H-4), 8.00 (2H, m, Ph), 7.53 (3H, m, Ph), 4.37 (2H, q, *J* = 7.2 Hz, COOCH_2_CH_3_), 4.36 (2H, q, *J* = 7.2 Hz, COOCH_2_CH_3_), 2.88 (2H, t, *J* = 5.4 Hz), 2.69 (2H, t, *J* = 5.4 Hz), 1.80 (4H, m) (4 × CH_2_), 1.39 (3H, t, *J* = 7.2 Hz, COOCH_2_CH_3_), 1.37 (3H, t, *J* = 7.2 Hz, COOCH_2_CH_3_); ^13^C NMR (CDCl_3_, 126 MHz) δ 169.0 (COO), 167.8 (COO), 165.5 (NHCO), 144.8, 138.1, 135.9, 134.8, 132.0, 129.3, 128.8, 127.3, 122.3, 112.1 (10 × C_Ar_), 62.1 (CH_2_CH_3_), 61.2 (CH_2_CH_3_), 30.5, 26.1, 22.7, 22.2 (4 × CH_2_), 14.2 (CH_2_CH_3_), 13.9 (CH_2_CH_3_); HREIMS *m*/*z* 396.1797 (calcd for C_23_H_26_NO_5_ (M+H)^+^, 396.1805).

Diethyl 3-benzamido-6,7,8,9-tetrahydro-5*H*-benzo [7]annulene-1,2-dicarboxylate (**4Bo**): isolated by column chromatography (petroleum ether/EtOAc = 3:1), off-white waxy solid (petroleum ether/EtOAc); IR ν_max_ 3258 (N–H), 2970 (H–C-sp^3^), 2926, 2853, 1715 (COO), 1671 (NHCO), 1585, 1525, 1492 cm^–1^; ^1^H NMR (CDCl_3_, 500 MHz) δ 11.59 (1H, s, NH), 8.73 (1H, s, H-4), 8.00 (2H, m Ph), 7.53 (3H, m, Ph), 4.37 (4H, q, *J* = 7.2 Hz, 2 × COOCH_2_CH_3_), 2.91 (2H, m), 2.70 (2H, m), 1.83 (2H, m), 1.67 (4H, m), (2 × CH_2_), 1.39 (3H, t, *J* = 7.2 Hz, COOCH_2_CH_3_), 1.37 (3H, t, *J* = 7.2 Hz, COOCH_2_CH_3_); ^13^C NMR (CDCl_3_, 126 MHz) δ 169.4 (COO), 167.8 (COO), 165.5 (NHCO), 151.2, 139.1, 135.4, 135.0, 134.8, 132.0, 128.8, 127.3, 122.1, 111.5 (10 × C_Ar_), 62.1 (CH_2_CH_3_), 61.2 (CH_2_CH_3_), 36.8, 31.9, 31.5, 27.7, 27.5 (5 × CH_2_), 14.2 (CH_2_CH_3_), 13.9 (CH_2_CH_3_); HREIMS *m/z* 410.1956 (calcd for C_24_H_28_NO_5_ (M+H)^+^, 410.1962).

Dipropyl 3-benzamido-6-(thiophen-2-yl)phthalate (**4Ca**): isolated by column chromatography (petroleum ether/EtOAc = 3:1), off-white powder (petroleum ether/EtOAc); mp 122.5–124.1 °C; IR ν_max_ 3363 (N–H), 2964 (H–C-sp^3^), 2878, 1717 (COO), 1688 (NHCO), 1511, 1489 cm^−1^; ^1^H NMR (CDCl_3_, 500 MHz) δ 11.48 (1H, s, NH), 8.92 (1H, d, *J* = 8.8 Hz, H-4 or H-5), 8.02 (2H, m, Ph), 7.66 (1H, d, *J* = 8.8 Hz, H-4 or H-5), 7.55 (3H, m Ph), 7.35 (1H, m C_4_H_3_S), 7.05 (2H, m, C_4_H_3_S), 4.29 (2H, t, *J* = 6.9 Hz, COOCH_2_CH_2_CH_3_), 4.01 (2H, t, *J* = 6.6 Hz, COOCH_2_CH_2_CH_3_), 1.74 (2H, deg. hept, *J* = 6.9 Hz, COOCH_2_CH_2_CH_3_), 1.48 (2H, deg. hept, *J* = 6.9 Hz, COOCH_2_CH_2_CH_3_), 0.98 (3H, t, *J* = 7.4 Hz, COOCH_2_CH_2_CH_3_), 0.77 (3H, t, *J* = 7.4 Hz, COOCH_2_CH_2_CH_3_); ^13^C NMR (CDCl_3_, 126 MHz) δ 168.4 (COO), 167.7 (COO), 165.6 (NHCO), 140.2, 140.0, 135.8, 135.6, 134.5, 132.2, 128.9, 127.8, 127.4, 127.2, 127.1, 126.3, 121.9, 115.1 (10 × C_Ar_, 4 × C_Thioph_), 68.3, 67.4 (2 × CH_2_CH_2_CH_3_), 21.7, 21.5 (2 × CH_2_CH_2_CH_3_), 10.4, 10.3 (2 × CH_2_CH_2_CH_3_); HREIMS *m/z* 452.1522 (calcd for C_25_H_26_NO_5_S (M+H)^+^, 452.1526).

Dipropyl 5-benzamido-4′-methoxy-2-methyl-[1,1′-biphenyl]-3,4-dicarboxylate (**4Cb**): isolated by three rounds of column chromatography (first column was petroleum ether/EtOAc = 3:1, second and third columns were petroleum ether/EtOAc = 10:1), yellow waxy solid (petroleum ether/EtOAc); IR ν_max_ 3305 (N–H), 2966 (H–C-sp^3^), 1730 (COO), 1676 (NHCO), 1605, 1579, 1506, 1490, 1462, 1403 cm^−1^; ^1^H NMR (CDCl_3_, 500 MHz) δ 11.50 (1H, s, NH), 8.81 (1H, s, H-6), 8.00 (2H, m, Ph), 7.56 (1H, m, Ph), 7.51 (2H, m, Ph), 7.28 and 6.96 (2H each, AA’XX’, *J* = 8.7 Hz, C_6_H_4_OCH_3_), 4.30 (2H, t, *J* = 7.4 Hz, COOCH_2_CH_2_CH_3_), 4.27 (2H, t, *J* = 7.2 Hz, COOCH_2_CH_2_CH_3_), 3.86 (3H, s, OCH_3_), 2.20 (3H, s, CH_3_), 1.79 (4H, m, 2 × COOCH_2_CH_2_CH_3_), 1.02 (6H, q, *J* = 7.6 Hz, 2 × COOCH_2_CH_2_CH_3_); ^13^C NMR (CDCl_3_, 126 MHz) δ 169.3 (COO), 167.8 (COO), 165.5 (NHCO), 159.2, 148.1, 138.4, 136.5, 134.7, 132.7, 132.0, 130.3, 128.8, 127.9, 127.3, 123.4, 113.7, 113.2 (14 × C_Ar_), 68.0, 67.2 (2 × CH_2_CH_2_CH_3_), 55.4 (OCH_3_), 21.9, 21.8 (2 × CH_2_CH_2_CH_3_), 17.3 (CH_3_), 10.7, 10.4 (2 × CH_2_CH_2_CH_3_); HREIMS *m*/*z* 490.2221 (calcd for C_29_H_32_NO_6_ (M+H)^+^, 490.2224).

Dipropyl 5-benzamido-3′,4′-dimethoxy-2-methyl-[1,1′-biphenyl]-3,4-dicarboxylate (**4Cc**): isolated by column chromatography (petroleum ether/EtOAc = 3:1), yellow waxy solid (petroleum ether/EtOAc); IR ν_max_ 3348 (N–H), 2963 (H–C-sp^3^), 2878, 1728 (COO), 1675 (NHCO), 1602, 1514, 1491 cm^−1^; ^1^H NMR (CDCl_3_, 500 MHz) δ 11.50 (1H, s, NH), 8.83 (1H, s, H-6), 8.00 (2H, m, Ph), 7.53 (3H, m, Ph), 6.89 (3H, m, C_6_H_3_(OCH_3_)_2_), 4.31 (2H, t *J* = 7.6 Hz, COOCH_2_CH_2_CH_3_), 4.28 (2H, t *J* = 7.6 Hz, COOCH_2_CH_2_CH_3_), 3.94 (3H, s, OCH_3_), 3.90 (3H, s, OCH_3_), 2.21 (3H, s, CH_3_), 1.79 (4H, deg. tq, *J* = 7.0 Hz, 2 × COOCH_2_CH_2_CH_3_), 1.03 (3H, t, *J* = 7.4 Hz, COOCH_2_CH_2_CH_3_), 1.01 (3H, t, *J* = 7.4 Hz, COOCH_2_CH_2_CH_3_); ^13^C NMR (CDCl_3_, 126 MHz) δ 169.3 (COO), 167.8 (COO), 165.5 (NHCO), 148.7, 148.6, 148.3, 138.3, 136.5, 134.7, 133.0, 132.1, 128.8, 127.9, 127.3, 123.3, 121.5, 113.3, 112.3, 111.0 (16 × C_Ar_), 68.0, 67.2 (2 × CH_2_CH_2_CH_3_), 56.00, 55.98 (2 × OCH_3_), 21.9, 21.8 (2 × CH_2_CH_2_CH_3_), 17.3 (CH_3_), 10.7, 10.4 (2 × CH_2_CH_2_CH_3_); HREIMS *m*/*z* 520.2322 (calcd for C_30_H_34_NO_7_ (M+H)^+^, 520.2330).

4-Methyl 1,2-dipropyl 6-benzamido-3-methylbenzene-1,2,4-tricarboxylate (**4Cd**): white powder (petroleum ether/MeOH); mp 118.2–120.7 °C; IR ν_max_ 3316 (N–H), 2973 (H–C-sp^3^), 1729 (COO), 1678 (NHCO), 1578, 1522, 1492, 1436, 1406 cm^−1^; ^1^H NMR (CDCl_3_, 500 MHz) δ 11.29 (1H, s, NH), 9.27 (1H, s, H-5), 8.00 (2H, m, Ph), 7.58 (1H, m, Ph), 7.53 (2H, m, Ph), 4.30 (2H, t, *J* = 7.1 Hz, COOCH_2_CH_2_CH_3_), 4.27 (2H, t, *J* = 7.0 Hz, COOCH_2_CH_2_CH_3_), 3.94 (3H, s, COOCH_3_), 2.47 (3H, s, CH_3_), 1.77 (4H, m, 2 × COOCH_2_CH_2_CH_3_), 1.02 (3H, t, *J* = 7.4 Hz, COOCH_2_CH_2_CH_3_), 0.99 (3H, t, *J* = 7.5 Hz, COOCH_2_CH_2_CH_3_); ^13^C NMR (CDCl_3_, 126 MHz) δ 168.4 (COO), 167.3 (COO), 167.1 (COO), 165.5 (NHCO), 138.1, 137.3, 135.9, 134.3, 132.3, 130.5, 128.9, 127.3, 123.2, 117.2 (10 × C_Ar_), 68.4, 67.4 (2 × CH_2_CH_2_CH_3_), 52.6 (COOCH_3_), 21.8, 21.7 (2 × CH_2_CH_2_CH_3_), 17.0 (CH_3_), 10.6, 10.4 (2 × CH_2_CH_2_CH_3_); HREIMS *m*/*z* 442.1849 (calcd for C_24_H_28_NO_7_ (M+H)^+^, 442.1860).

Dipropyl 4-acetyl-3-methyl-6-(4-nitrobenzamido)phthalate (**4Ce**): pale brown powder (petroleum ether/MeOH); mp 103.7–105.2 °C; IR ν_max_ 3338 (N–H), 2975 (H–C-sp^3^), 1728 (COO), 1701, 1677 (NHCO), 1601, 1580, 1520, 1490 cm^–1^; ^1^H NMR (CDCl_3_, 500 MHz) δ 11.77 (1H, s, NH), 9.13 (1H, s, H-5), 8.39 (2H, d, *J* = 8.4 Hz, C_6_H_4_NO_2_), 8.17 (2H, d, *J* = 8.4 Hz, C_6_H_4_NO_2_), 4.32 (2H, t, *J* = 6.9 Hz, COOCH_2_CH_2_CH_3_), 4.28 (2H, t, *J* = 6.9 Hz, COOCH_2_CH_2_CH_3_), 2.64 (3H, s, COCH_3_), 2.35 (3H, s, CH_3_), 1.78 (4H, m, 2 × COOCH_2_CH_2_CH_3_), 1.03 (3H, t, *J* = 7.2 Hz, COOCH_2_CH_2_CH_3_), 1.00 (3H, t, *J* = 7.3 Hz, COOCH_2_CH_2_CH_3_); ^13^C NMR (CDCl_3_, 126 MHz) δ 201.9 (COCH_3_), 168.2 (COO), 167.4 (COO), 163.5 (NHCO), 150.0, 144.4, 139.8, 138.2, 137.9, 128.8, 128.5, 124.1, 120.6, 115.9 (10 × C_Ar_), 68.7, 67.5 (2 × CH_2_CH_2_CH_3_), 30.4 (COCH_3_), 21.8, 21.7 (2 × CH_2_CH_2_CH_3_), 16.6 (CH_3_), 10.6, 10.3 (2 × CH_2_CH_2_CH_3_); HREIMS *m/z* 488.2025 (calcd for C_24_H_30_N_3_O_8_ (M+NH_4_)^+^, 488.2027).

4-Methyl 1,2-dipropyl 6-benzamido-3-(2-methoxy-2-oxoethyl)benzene-1,2,4-tricarboxylate (**4Cf**): pale brown powder (xylene); mp 130.9–134.4 °C; IR ν_max_ 3328 (N–H), 2953 (H–C-sp^3^), 1731, 1720 (COO), 1685 (NHCO), 1576, 1523, 1492 1434 cm^−1^; ^1^H NMR (CDCl_3_, 500 MHz) δ 11.25 (1H, s, NH), 9.46 (1H, s, H-5), 8.00 (2H, m, Ph), 7.59 (1H, m, Ph), 7.54 (2H, m, Ph), 4.29 (2H, t, *J* = 6.9 Hz, COOCH_2_CH_2_CH_3_), 4.24 (2H, t, *J* = 6.9 Hz, COOCH_2_CH_2_CH_3_), 4.07 (2H, s, CH_2_COOCH_3_), 3.93 (3H, s, COOCH_3_), 3.68 (3H, s, COOCH_3_), 1.75 (4H, m, 2 × COOCH_2_CH_2_CH_3_), 0.987 (3H, t, *J* = 7.4 Hz, COOCH_2_CH_2_CH_3_), 0.985 (3H, t, *J* = 7.4 Hz, COOCH_2_CH_2_CH_3_); ^13^C NMR (CDCl_3_, 126 MHz) δ 170.8, 167.8, 167.1, 166.5, 165.5 (5 × CO), 139.2, 137.5, 135.2, 134.1, 132.4, 129.0, 127.5, 127.3, 124.3, 118.2 (10 × C_Ar_), 68.6, 67.7 (2 × CH_2_CH_2_CH_3_), 52.8, 52.1 (2 × COOCH_3_), 35.6 (CH_2_), 21.8, 21.7 (2 × CH_2_CH_2_CH_3_), 10.5, 10.4 (2 × CH_2_CH_2_CH_3_); HREIMS *m*/*z* 500.1917 (calcd for C_26_H_30_NO_9_ (M+H)^+^, 500.1915).

Dipropyl 6-benzamido-2,3-dihydro-1*H*-indene-4,5-dicarboxylate (**4Cg**): isolated by two rounds of column chromatography (first column was petroleum ether/EtOAc = 3:1, second column was petroleum ether/EtOAc = 4:1), orange waxy solid (petroleum ether/EtOAc); IR ν_max_ 3330 (N–H), 2962 (H–C-sp^3^), 1723 (COO), 1676 (NHCO), 1589, 1525, 1491, 1454, 1433 cm^−1^; ^1^H NMR (CDCl_3_, 500 MHz) δ 11.26 (1H, s, NH), 8.73 (1H, s, H-7), 8.00 (2H, m, Ph), 7.56 (1H, m, Ph), 7.52 (2H, m, Ph), 4.25 (4H, t, *J* = 6.8 Hz, 2 × COOCH_2_CH_2_CH_3_), 3.01 (2H, t, *J* = 7.5 Hz), 2.95 (2H, t, *J* = 7.5 Hz), 2.12 (2H, deg. dt, *J* = 7.5 Hz) (3 × CH_2_), 1.75 (4H, m, 2 × COOCH_2_CH_2_CH_3_), 1.02 (3H, t, *J* = 7.4 Hz, COOCH_2_CH_2_CH_3_), 0.98 (3H, t, *J* = 7.4 Hz, COOCH_2_CH_2_CH_3_); ^13^C NMR (CDCl_3_, 126 MHz) δ 168.5 (COO), 168.3 (COO), 165.4 (NHCO), 151.2, 139.0, 137.8, 134.6, 132.0, 131.2, 128.8, 127.3, 118.6, 113.8 (10 × C_Ar_), 67.8, 67.1 (2 × CH_2_CH_2_CH_3_), 33.6, 31.6, 25.0 (3 × CH_2_), 22.0, 21.7 (2 × CH_2_CH_2_CH_3_), 10.6, 10.4 (2 × CH_2_CH_2_CH_3_); HREIMS *m/z* 410.1959 (calcd for C_24_H_28_NO_5_ (M+H)^+^, 410.1962).

Diisopropyl 5-benzamido-4′-methoxy-2-methyl-[1,1′-biphenyl]-3,4-dicarboxylate (**4Da**): off-white powder (petroleum ether/MeOH); mp 132.2–135.3 °C; IR ν_max_ 3351 (N–H), 2979 (H–C-sp^3^), 1721 (COO), 1694, 1672 (NHCO), 1605, 1581, 1525, 1509, 1492, 1464, 1401 cm^–1^; ^1^H NMR (CDCl_3_, 500 MHz) δ 11.47 (1H, s, NH), 8.78 (1H, s, H-6), 7.99 (2H, m, Ph), 7.53 (3H, m, Ph), 7.27 and 6.95 (2H each, AA’XX’, *J* = 8.7 Hz, C_6_H_4_OCH_3_), 5.33 (1H, hept, *J* = 6.1 Hz, COOCH(CH_3_)_2_), 5.26 (1H, hept, *J* = 6.1 Hz, COOCH(CH_3_)_2_), 3.86 (3H, s, OCH_3_), 2.21 (3H, s, CH_3_), 1.42 (6H, d, *J* = 6.3 Hz, COOCH(CH_3_)_2_), 1.39 (6H, d, *J* = 6.3 Hz, COOCH(CH_3_)_2_); ^13^C NMR (CDCl_3_, 126 MHz) δ 168.6 (COO), 167.3 (COO), 165.4 (NHCO), 159.2, 147.9, 138.2, 136.7, 134.8, 132.8, 132.0, 130.3, 128.8, 127.7, 127.3, 123.3, 113.7, 113.5 (14 × C_Ar_), 70.6, 69.4 (2 × CH(CH_3_)_2_), 55.4 (OCH_3_), 21.8, 21.6 (2 × CH(CH_3_)_2_), 17.1 (CH_3_); HREIMS *m/z* 490.2226 (calcd for C_29_H_32_NO_6_ (M+H)^+^, 490.2224).

Diisopropyl 4-acetyl-6-benzamido-3-methylphthalate (**4Db**): isolated by column chromatography (petroleum ether/EtOAc = 3:1), pale yellow powder (petroleum ether); mp 117.3–118.9 °C; IR ν_max_ 3350 (N–H), 2981 (H–C-sp^3^), 1724 (COO), 1702, 1674 (NHCO), 1604, 1580, 1528, 1494, 1439, 1403 cm^–1^; ^1^H NMR (CDCl_3_, 500 MHz) δ 11.40 (1H, s, NH), 9.15 (1H, s, H-5), 7.99 (2H, m, Ph), 7.59 (1H, m, Ph), 7.53 (2H, m, Ph), 5.32 (1H, hept, *J* = 6.3 Hz, COOCH(CH_3_)_2_), 5.24 (1H, hept, *J* = 6.2 Hz, COOCH(CH_3_)_2_), 2.63 (3H, s, COCH_3_), 2.36 (3H, s, CH_3_), 1.41 (6H, d, *J* = 6.3 Hz, COOCH(CH_3_)_2_), 1.38 (6H, d, *J* = 6.3 Hz, COOCH(CH_3_)_2_); ^13^C NMR (CDCl_3_, 126 MHz) δ 202.2 (COCH_3_), 167.8 (COO), 166.7 (COO), 165.7 (NHCO), 143.9, 138.3, 137.7, 134.4, 132.3, 128.9, 128.1, 127.3, 120.8, 116.6 (10 × C_Ar_), 71.2, 69.7 (2 × CH(CH_3_)_2_), 30.4 (COCH_3_), 21.8, 21.5 (2 × CH(CH_3_)_2_), 16.4 (CH_3_); HREIMS *m/z* 426.1912 (calcd for C_24_H_28_NO_6_ (M+H)^+^, 426.1911).

Dibutyl 5-benzamido-4′-methoxy-2-methyl-[1,1′-biphenyl]-3,4-dicarboxylate (**4Ea**): isolated by two rounds of column chromatography (first column was petroleum ether/EtOAc = 3:1, second column was petroleum ether/EtOAc = 10:1), orange oil (petroleum ether/EtOAc); IR ν_max_ 3297 (N–H), 2959 (H–C-sp^3^), 2873, 1730 (COO), 1676 (NHCO), 1579, 1507, 1491, 1462, 1403 cm^–1^; ^1^H NMR (CDCl_3_, 500 MHz) δ 11.49 (1H, s, NH), 8.81 (1H, s, H-6), 8.00 (2H, m, Ph), 7.53 (3H, m, Ph), 7.28 and 6.96 (2H each, AA’XX’, *J* = 8.6 Hz, C_6_H_4_OCH_3_), 4.34 (2H, t, *J* = 6.9 Hz, COOCH_2_CH_2_CH_2_CH_3_), 4.31 (2H, t, *J* = 6.7 Hz, COOCH_2_CH_2_CH_2_CH_3_), 3.86 (3H, s, OCH_3_), 2.20 (3H, s, CH_3_), 1.74 (4H, m, 2 × COOCH_2_CH_2_CH_2_CH_3_), 1.45 (4H, m, 2 × COOCH_2_CH_2_CH_2_CH_3_), 0.973 (3H, t, *J* = 7.4 Hz, COOCH_2_CH_2_CH_2_CH_3_), 0.965 (3H, t, *J* = 7.4 Hz, COOCH_2_CH_2_CH_2_CH_3_); ^13^C NMR (CDCl_3_, 126 MHz) δ 169.3 (COO), 167.8 (COO), 165.5 (NHCO), 159.2, 148.1, 138.3, 136.6, 134.7, 132.7, 132.0, 130.3, 128.8, 127.8, 127.3, 123.4, 113.7, 113.2 (14 × C_Ar_), 66.3, 65.5 (2 × CH_2_CH_2_CH_2_CH_3_), 55.4 (OCH_3_), 30.6, 30.4 (2 × CH_2_CH_2_CH_2_CH_3_), 19.4, 19.1 (2 × CH_2_CH_2_CH_2_CH_3_), 17.3 (CH_3_), 13.8, 13.7 (2 × CH_2_CH_2_CH_2_CH_3_); HREIMS *m*/*z* 518.2530 (calcd for C_31_H_36_NO_6_ (M+H)^+^, 518.2537).

Bis(2-methoxyethyl) 4-benzamido-[1,1′-biphenyl]-2,3-dicarboxylate (**4Fa**): isolated by column chromatography (petroleum ether/EtOAc = 1:1), yellow oil (petroleum ether/EtOAc); IR ν_max_ 3334 (N–H), 2284 (H–C-sp^3^), 1725 (COO), 1681 (NHCO), 1584, 1515, 1488, 1447, 1396 cm^−1^; ^1^H NMR (CDCl_3_, 500 MHz) δ 11.19 (1H, s, NH), 8.90 (1H, d, *J* = 8.8 Hz, H-5 or H-6), 8.02 (2H, m), 7.55 (4H, m), 7.38 (5H, m) (2 × Ph and H-5 or H-6), 4.47 (2H, m, COOCH_2_CH_2_OCH_3_), 4.15 (2H, m, COOCH_2_CH_2_OCH_3_), 3.63 (2H, m, COOCH_2_CH_2_OCH_3_), 3.31 (3H, s, COOCH_2_CH_2_OCH_3_), 3.26 (2H, m, COOCH_2_CH_2_OCH_3_), 3.23 (3H, s, COOCH_2_CH_2_OCH_3_); ^13^C NMR (CDCl_3_, 126 MHz) δ 168.2 (COO), 167.6 (COO), 165.6 (NHCO), 139.4, 139.3, 135.6, 135.1, 134.7, 134.5, 132.2, 128.9, 128.7, 128.2, 127.7, 127.4, 122.2, 115.4 (14 × C_Ar_), 69.74, 69.70, 65.4, 64.4 (2 × OCH_2_CH_2_O), 58.9, 58.8 (2 × OCH_3_); HREIMS *m*/*z* 478.1855 (calcd for C_27_H_28_NO_7_ (M+H)^+^, 478.1860).

Bis(2-methoxyethyl) 5-benzamido-4′-methoxy-2-methyl-[1,1′-biphenyl]-3,4-dicarboxylate (**4Fb**): isolated by column chromatography (petroleum ether/EtOAc = 1:1), white powder (Et_2_O); mp 86.9–88.8 °C; IR ν_max_ 3362 (N–H), 2886 (H–C-sp^3^), 1722 (COO), 1671 (NHCO), 1603, 1579, 1525, 1508, 1493, 1454, 1403 cm^−1^; ^1^H NMR (CDCl_3_, 500 MHz) δ 11.34 (1H, s, NH), 8.80 (1H, s, H-6), 7.99 (2H, m, Ph), 7.56 (1H, m, Ph), 7.51 (2H, m, Ph), 7.27 and 6.96 (2H each, AA’XX’, *J* = 8.7 Hz, C_6_H_4_OCH_3_), 4.50 (4H, m, 2 × COOCH_2_CH_2_OCH_3_), 3.86 (3H, s, OCH_3_), 3.71 (2H, m, COOCH_2_CH_2_OCH_3_), 3.68 (2H, m, COOCH_2_CH_2_OCH_3_), 3.39 (3H, s, COOCH_2_CH_2_OCH_3_), 3.37 (3H, s, COOCH_2_CH_2_OCH_3_), 2.22 (3H, s, CH_3_); ^13^C NMR (CDCl_3_, 126 MHz) δ 169.1 (COO), 167.5 (COO), 165.5 (NHCO), 159.2, 148.3, 138.3, 136.3, 134.7, 132.7, 132.0, 130.3, 128.8, 128.2, 127.3, 123.5, 113.7, 113.1 (14 × C_Ar_), 70.2, 69.9, 65.3, 64.6 (2 × OCH_2_CH_2_O), 59.0, 58.9 (2 × OCH_3_), 55.4 (ArOCH_3_), 17.2 (CH_3_); HREIMS *m*/*z* 522.2115 (calcd for C_29_H_32_NO_8_ (M+H)^+^, 522.2122).

1,2-Bis(2-methoxyethyl) 4-methyl 6-benzamido-3-methylbenzene-1,2,4-tricarboxylate (**4Fc**): isolated by column chromatography (petroleum ether/EtOAc = 1:1), off-white powder (petroleum ether); mp 84.8–86.3 °C; IR ν_max_ 3259 (N–H), 2883 (H–C-sp^3^), 1737, 1722 (COO), 1677 (NHCO), 1601, 1579, 1521, 1492, 1435, 1400 cm^−1^; ^1^H NMR (CDCl_3_, 500 MHz) δ 11.11 (1H, s, NH), 9.25 (1H, s, H-5), 7.99 (2H, m, Ph), 7.58 (1H, m, Ph), 7.52 (2H, m, Ph), 4.50 (4H, m, 2 × COOCH_2_CH_2_OCH_3_), 3.94 (3H, s, COOCH_3_), 3.70 (2H, m, COOCH_2_CH_2_OCH_3_), 3.66 (2H, m, COOCH_2_CH_2_OCH_3_), 3.39 (3H, s, COOCH_2_CH_2_OCH_3_), 3.34 (3H, s, COOCH_2_CH_2_OCH_3_), 2.49 (3H, s, CH_3_); ^13^C NMR (CDCl_3_, 126 MHz) δ 168.1, 167.1, 167.0, 165.6 (3 × COO, NHCO), 137.9, 137.0, 136.0, 134.3, 132.3, 130.9, 128.9, 127.4, 123.4, 117.3 (10 × C_Ar_), 70.1, 69.7, 65.7, 64.8 (2 × OCH_2_CH_2_O), 59.0, 58.9 (2 × OCH_3_), 52.6 (COOCH_3_), 16.9 (CH_3_); HREIMS *m/z* 474.1748 (calcd for C_24_H_28_NO_9_ (M+H)^+^, 474.1759).

Dimethyl 2-(4-benzamido-2,3-bis(methoxycarbonyl)phenyl)furan-3,4-dicarboxylate (**6**): isolated by column chromatography (petroleum ether/EtOAc = 1:1), light yellow powder (CDCl_3_); mp 166.0–169.4 °C; IR ν_max_ 3323 (N–H), 3151, 2949 (H–C-sp^3^), 1730 (COO), 1710, 1677 (NHCO), 1616, 1586, 1549, 1516 cm^–1^; ^1^H NMR (CDCl_3_, 500 MHz) δ 11.21 (1H, s, NH), 8.97 (1H, d, *J* = 8.9 Hz, H-5′ or H-6′), 8.00 (2H, m, Ph), 7.95 (1H, s, H-5), 7.77 (1H, s, *J* = 8.9 Hz, H-5′ or H-6′), 7.60 (1H, m, Ph), 7.54 (3H, m, Ph), 3.91 (3H s), 3.87 (3H, s), 3.78 (3H, s), 3.76 (3H, s) (4 × COOCH_3_); ^13^C NMR (CDCl_3_, 126 MHz) δ 167.7, 167.6, 165.6, 162.9, 162.0 (4 × COO, NHCO), 153.7, 147.1, 141.2, 135.5, 135.3, 134.1, 132.5, 129.0, 127.4, 122.2, 122.0, 119.6, 116.1, 115.8 (10 × C_Ar_, 4 × C_Fur_), 53.3, 52.9, 52.4, 52.1 (4 × COOCH_3_); HREIMS *m*/*z* 496.1232 (calcd for C_25_H_22_NO_10_ (M+H)^+^, 496.1238).

### 3.3. Theoretical Calculations

Quantum–chemical calculations were carried out using ORCA 6.0 software [49]. We used the ωB97X-D4 range-separated hybrid functional [50] with DFT-D4 dispersion correction for van der Waals interactions [51] and the def2-TZVP basis set [52] along with the def2/J auxiliary basis set [53] for the RIJCOSX approximation for geometry optimization. The ωB97X functional is accurate for modelling systems with π stacking, van der Waals interactions, and dispersion effects. Due to its good cost/accuracy ratio, it is a recommended choice for the determination of geometry optimized structures and frequencies [54]. It has also been evaluated in Diels–Alder reactions [55]. The self-consistent field (SCF) energy convergence threshold was set to $1\cdot 10^{-8}$ Ha. Solvation effects were accounted for using a conductor-like polarizable continuum model (CPCM) [56] with a dielectric constant and refractive index of xylene of 2.399 and 1.4995, respectively. Thermodynamic functions were calculated at 298.15 K and 1.00 atm assuming ideal gas behaviour and treating all vibrations as harmonic. The standard Gibbs free energy was corrected for electronic energy; a domain-based local-pair natural orbital coupled cluster theory with single, double, and perturbative triple excitations (DLPNO-CCSD(T)) [57] with the def2-TZVPP [52] basis set and def2-TZVPP/C auxiliary basis set [58] was used to determine the electronic energies of the optimized structures. A method of Nudged Elastic Band with transition state optimization (TS-NEB) [59] was used to find the transition state structures. Vibrational frequencies were calculated for all nuclear geometry optimizations in order to check whether they were stable minima or transition states. The ground-state molecular geometries were characterized by the absence of imaginary (negative) frequencies, while the transition states were characterized by a single imaginary frequency. For all transition states, the values of the imaginary frequency are given in the Supporting Information. The number of intermediate images and images free to move was set to 8. Images were calculated using the limited-memory BFGS (L-BFGS) optimizer. The maximum allowed step size was set to 0.10 Bohr and a maximum force of $2\cdot 10^{-3}$ Ha/Bohr was set as the convergence parameter for climbing image. The highest-energy image from the NEB calculation was used to optimize the transition state. An intrinsic reaction coordinate (IRC) path was calculated using Morokuma et al.’s method implemented in ORCA. The global electron density transfer (GEDT) [44,46] at the transition structure was calculated from the sum of the Hirshfeld charges of the atoms belonging to the dienophile part. The absolute values and the electron density flux are given in the figures in the Supporting Information.

## 4. Conclusions

We have shown that the cycloadditions between various 3-acylamino-2*H*-pyran-2-one derivatives **1a**–**ag** and dialkyl acetylenedicarboxylates **2** acting as dienophiles yield substituted dialkyl 3-acylaminophthalic esters **4** (61 examples, 43 novel compounds, yields 3–87%, average yield 56%, median yield 60%). The reactions take place in two steps: initial [4+2] cycloaddition yields the oxabicyclo[2.2.2]octadiene intermediates **3** (that are not isolated, corroborated by the results of the computational study as well), and the second step being an irreversible elimination of carbon dioxide via retro-hetero-Diels–Alder reaction, providing **4**. However, the reaction conditions to achieve appropriate conversions (above 95% in most cases) are rather harsh: heating at 190 °C in xylene for prolonged reaction times (up to 58 h, average time 9.4 h, median time 8 h). However, in some cases, even this was insufficient (for **4Ax** and **4Az,** 42 and 40 h, respectively, with only 60–70% conversion; for **4Fa** and **4Fc**, 28 h with only 85% conversion).

To elucidate the reactivity of various 2*H*-pyran-2-ones **1**, the reactions (at 170 °C) were stopped after 90 min: conversions above 90% were reached only by two 2*H*-pyran-2-one derivatives (**1u** and **1aa**); slightly lower conversions (75–85%) were achieved by **1l**, **1m** and **1ad**. These observations are somewhat unexpected, as those 2*H*-pyran-2-ones **1** that were presumably the most electron-rich (such as **1l** having a 4-methoxyphenyl substituent) were not the most reactive. Additionally, the position of the electron-donating group on the 2*H*-pyran-2-one ring strongly influenced their reactivity: in the case of **1d** having the same electron-donating group as **1l**, the change in its position from C-4 (in **1l**) to C-5 (in **1d**) decreased the conversion from 75–85% to only 15–25%, making **1d** even less reactive than electron-deficient examples, such as **1p** (having a 4-acetyl substituent), where the conversions were 25–35%. However, these reactivity differences, observed experimentally, were also confirmed by computational calculations and explained by the electron delocalization that disturbs the 1,3-diene character of these 2*H*-pyran-2-ones **1**. This effect is most important in 6-substituted 2*H*-pyran-2-ones (for example, extremely low reactivity of **1d**). The perturbing effects are even more pronounced in those 2*H*-pyran-2-ones **1** that contain substituents on positions 5 and 6, where one is electron-donating and the other electron-withdrawing, thus exerting electronic push–pull effects (such as **1x**).

A special case was observed with furyl-substituted 2*H*-pyran-2-one **1h**, where cycloaddition of dimethyl acetylenedicarboxylate (**2A**) produced two different products: not only the 2*H*-pyran-2-one system was reacting as a diene (thus yielding **4Ah**), but also the furan ring reacted with acetylenedicarboxylate **2A**; however, intermediate **5** extruded a molecule of acetylene (via an irreversible retro-Diels–Alder reaction) thus yielding **6** as the other final product. We were able to fine-tune the necessary reaction conditions to be able to prepare and isolate either of the two products (**4Ah** and **6**), albeit in low yields due to the complications with isolation and purification. It is of interest to note that the reactivity of the furan ring in **1h** with diethyl acetylenedicarboxylate (**2B**) was not sufficient to be able to isolate product type **6**; additionally, the cycloadducts on the thiophene ring (in **1i** and **1af**) with any of the acetylenes (**2A**–**C**) could also not be detected.

## Figures and Tables

**Table 1:** Cycloadditions of dimethyl acetylenedicarboxylate (**2A**) in xylene at 190 °C.

Run	Starting 2*H*-Pyran-2-one					*t* [h] ^a^	Product	Isolation ^b^	Yield [%]
	R^1^	R^2^	R^3^	R^4^	1				
1	Me	H	H	Ph	**1a** [29]	6 ^c^	**4Aa** [24]	I2	66
2	Ph	H	H	Ph	**1b** [29]	8 ^c,d^	**4Ab** [23]	I2	57
3	4-Me-C_6_H_4_	H	H	Ph	**1c**	8 ^c,d^	**4Ac** [20]	I1	62
4	4-MeO-C_6_H_4_	H	H	Ph	**1d**	12 ^c,d^	**4Ad** [20]	I3	69
5	4-NO_2_-C_6_H_4_	H	H	Ph	**1e**	22 ^c,d^	**4Ae**	I3	60 ^e^
6	3-Cl-C_6_H_4_	H	H	Ph	**1f**	16 ^c,d^	**4Af**	I1	70
7	4-Ph-C_6_H_4_	H	H	Ph	**1g**	10 ^c,d^	**4Ag**	I1	81
8	Furan-2-yl	H	H	Ph	**1h** [29]	15 ^c,d^	**4Ah**	I4	27
9	Furan-2-yl	H	H	Ph	**1h** [29]	25 ^c,f^	**6**	I4	4
10	Thiophen-2-yl	H	H	Ph	**1i** [29]	12 ^c,d^	**4Ai**	I3	53
11	Naphthalen-2-yl	H	H	Ph	**1j** [30]	10 ^c,d^	**4Aj**	I2	64 ^e^
12	Me	Ph	H	Ph	**1k** [31]	3 ^c^	**4Ak**	I1	79
13	Me	4-MeO-C_6_H_4_	H	Ph	**1l** [32]	2 ^c^	**4Al** [24]	I1	87
14	Me	3,4-(OMe)_2_-C_6_H_3_	H	Ph	**1m** [32]	2 ^c^	**4Am** [23]	I1	87
15	Me	COOMe	H	Ph	**1n**	8 ^c,d^	**4An**	I1	79
16	Me	COPh	H	Ph	**1o** [33]	7 ^c^	**4Ao** [25]	I3	75
17	Me	COMe	H	Ph	**1p** [34]	7 ^c^	**4Ap** [23,25]	I2	73
18	Me	COMe	H	4-NO_2_-C_6_H_4_	**1q**	8 ^c^	**4Aq** [23]	I1	62
19	Me	COMe	H	4-MeO-C_6_H_4_	**1r**	7 ^c^	**4Ar** [23]	I1	72
20	Me	COMe	H	3,4,5-(OMe)_3_-C_6_H_2_	**1s**	7 ^c^	**4As**	I4	76 ^e^
21	Me	COMe	H	Me	**1t** [35]	7 ^c^	**4At**	I4	62
22	Et	Me	H	Ph	**1u**	1 ^c^	**4Au**	I4	63
23	Ph	Ph	H	Ph	**1v**	21 ^d,g^	**4Av**	I2	55
24	4-F-C_6_H_4_	Me	H	Ph	**1w**	8 ^c,d^	**4Aw**	I2	79
25	4-MeO-C_6_H_4_	COOEt	H	Ph	**1x**	42 ^h^	**4Ax**	I4	6
26	CH_2_COOMe	COOMe	H	Ph	**1y** [36]	8 ^c,d^	**4Ay** [25]	I2	59
27	COOMe	4-MeO-C_6_H_4_	H	Ph	**1z**	40 ^i^	**4Az**	I4	3
28	-(CH_2_)_3_-		H	Ph	**1aa** [32]	1 ^c^	**4Aaa**	I2	64
29	-(CH_2_)_4_-		H	Ph	**1ab** [37]	5 ^c^	**4Aab**	I2	78
30	-(CH_2_)_6_-		H	Ph	**1ac** [32]	4 ^c^	**4Aac** [23]	I2	69
31	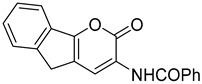				**1ad** [38]	2 ^c^	**4Aad**	I1	58 ^e^
32	Ph	H	Me	Ph	**1ae** [39]	10 ^c,d^	**4Aae**	I2	58
33	Thiophen-2-yl	H	Me	Ph	**1af** [39]	15 ^d,g,j^	**4Aaf**	I4	20

^a^ General reaction conditions: 1 eq. of **1** and 2 eq. of **2A** (except where stated otherwise). ^b^ For isolations, see experimental section. ^c^ Conversion (>95%) estimated by ^1^H NMR of crude reaction mixture. ^d^ With 3 eq. of **2A**. ^e^ Yield after recrystallization. ^f^ With 10 eq. of **2A**. ^g^ Conversion approx. 90% estimated by ^1^H NMR of crude reaction mixture. ^h^ With 3 eq. of **2A**, after 26 h addition of another 3 eq. of **2A** (approx. conv. 70%). ^i^ With 3 eq. of **2A**, after 24 h addition of another 3 eq. of **2A** (approx. conv. 60%). ^j^ Highly complex product mixture.

**Table 2:** Cycloadditions of diethyl acetylenedicarboxylate (**2B**) in xylene at 190 °C.

Run	Starting 2*H*-Pyran-2-one					*t* [h] ^a^	Product	Isolation ^b^	Yield [%]
	R^1^	R^2^	R^3^	R^4^	1				
1	Me	H	H	Ph	**1a** [29]	12 ^c^	**4Ba** [24]	I4	47
2	Ph	H	H	Ph	**1b** [29]	17 ^c,d^	**4Bb**	I2	62
3	4-Me-C_6_H_4_	H	H	Ph	**1c**	15 ^c,d^	**4Bc**	I2	59
4	Furan-2-yl	H	H	Ph	**1h** [29]	17 ^c,d^	**4Bd**	I4	13 ^e^
5	Thiophen-2-yl	H	H	Ph	**1i** [29]	17 ^c,d^	**4Be**	I2	55
6	Me	4-MeO-C_6_H_4_	H	Ph	**1l** [32]	2 ^c^	**4Bf** [24]	I2	71
7	Me	3,4-(MeO)_2_-C_6_H_3_	H	Ph	**1m** [32]	2 ^c^	**4Bg** [24]	I2	82
8	Me	COOMe	H	Ph	**1n**	12 ^c,d^	**4Bh**	I1	66
9	Me	COMe	H	Ph	**1p** [34]	12 ^c^	**4Bi** [25]	I2	46 ^e^
10	Me	COMe	H	4-NO_2_-C_6_H_4_	**1q**	12 ^c^	**4Bj**	I1	65
11	Et	Me	H	Ph	**1u**	1^c^	**4Bk**	I4	35 ^e^
12	CH_2_COOMe	COOMe	H	Ph	**1z**	15 ^c,d^	**4Bl** [25,26]	I2	59
13	-(CH_2_)_3_-		H	Ph	**1ab** [37]	1.5 ^c^	**4Bm**	I4	53
14	-(CH_2_)_4_-		H	Ph	**1ac** [32]	11 ^c^	**4Bn**	I4	63
15	-(CH_2_)_5_-		H	Ph	**1ag** [32]	6 ^c^	**4Bo**	I4	49

^a^ General reaction conditions: 1 eq. of **1** and 2 eq. of **2B** (except where stated otherwise). ^b^ For isolations, see experimental section. ^c^ Conversion (>95%) estimated by ^1^H NMR of crude reaction mixture. ^d^ With 3 eq. of **2B**. ^e^ Yield after recrystallization.

**Table 3:** Cycloadditions of acetylenedicarboxylates **2C**–**F** in xylene at 190 °C.

Run	Starting 2*H*-Pyran-2-one					Dienophile		*t* [h] ^a^	Product	Isolation ^b^	Yield [%]
	R^1^	R^2^	R^3^	R^4^	1	R^5^	2				
1	Thiophen-2-yl	H	H	Ph	**1i** [29]	*n*-Pr	**2C** [40,41]	58 ^c,d^	**4Ca**	I4	31
2	Me	4-MeO-C_6_H_4_	H	Ph	**1l** [32]	*n*-Pr	**2C** [40,41]	3 ^c^	**4Cb**	I4	48
3	Me	3,4-(MeO)_2_-C_6_H_3_	H	Ph	**1m** [32]	*n*-Pr	**2C** [40,41]	3 ^c^	**4Cc**	I4	80
4	Me	COOMe	H	Ph	**1n**	*n*-Pr	**2C** [40,41]	12 ^c,d^	**4Cd**	I2	51
5	Me	COMe	H	4-NO_2_-C_6_H_4_	**1q**	*n*-Pr	**2C** [40,41]	7 ^c^	**4Ce**	I2	37
6	CH_2_COOMe	COOMe	H	Ph	**1z**	*n*-Pr	**2C** [40,41]	16.5 ^c,d^	**4Cf**	I2	33
7	-(CH_2_)_3_-		H	Ph	**1ab** [37]	*n*-Pr	**2C** [40,41]	1.5 ^c^	**4Cg**	I4	72
8	Me	4-MeO-C_6_H_4_	H	Ph	**1l** [32]	*i*-Pr	**2D** [40,42]	3 ^c^	**4Da**	I1	61
9	Me	COMe	H	Ph	**1p** [34]	*i*-Pr	**2D** [40,42]	9 ^c^	**4Db**	I4	33 ^e^
10	Me	4-MeO-C_6_H_4_	H	Ph	**1l** [32]	*n*-Bu	**2E** [40,43]	3 ^c^	**4Ea**	I4	27
11	Ph	H	H	Ph	**1b** [29]	CH_2_CH_2_OMe	**2F**	28 ^d,f^	**4Fa**	I4	45
12	Me	4-MeO-C_6_H_4_	H	Ph	**1l** [32]	CH_2_CH_2_OMe	**2F**	6 ^c^	**4Fb**	I4	32 ^e^
13	Me	COOMe	H	Ph	**1n**	CH_2_CH_2_OMe	**2F**	28 ^d,f^	**4Fc**	I4	43 ^e^

^a^ General reaction conditions: 1 eq. of **1** and 2 eq. of **2** (except where stated otherwise). ^b^ For isolations, see experimental section. ^c^ Conversion (>95%) estimated by ^1^H NMR of crude reaction mixture. ^d^ With 3 eq. of **2**. ^e^ Yield after recrystallization. ^f^ Conversion approx. 85% estimated by ^1^H NMR of crude reaction mixture.

**Table 4:** Activation standard Gibbs free energies for the cycloaddition reactions **1** + **2A** → **3** (${∆G}_{TS1}^{\#}$) and subsequent CO_2_ elimination reactions **3** → **4A** + CO_2_ (${∆G}_{TS2}^{\#}$).

Starting 2*H*-Pyran-2-one 1					Product	${∆G}_{TS1}^{\#}$ [kJ mol^–1^] ^a^	${∆G}_{TS2}^{\#}$ [kJ mol^–1^] ^a^
R^1^	R^2^	R^3^	R^4^				
-(CH_2_)_3_-		H	Ph	**1aa**	**4A** **aa**	87.7	70.7
Et	Me	H	Ph	**1u**	**4A** **u**	90.8	73.9
Ph	H	H	Ph	**1b**	**4A** **b**	96.6	62.3
Me	4-MeO-C_6_H_4_	H	Ph	**1l**	**4A** **l**	97.5	66.8
Me	H	H	Ph	**1a**	**4A** **a**	101.3	67.6
Me	COMe	H	Ph	**1p**	**4A** **p**	107.2	68.0
4-MeO-C_6_H_4_	H	H	Ph	**1d**	**4A** **d**	112.7	61.4
4-MeO-C_6_H_4_	COOEt	H	Ph	**1x**	**4A** **x**	142.6	53.0

^a^ ωB97X-D4 density functional with def2-TZVP basis set was used for geometry optimization; DLPNO-CCSD(T) method was used to correct the electronic energies; and NEB-TS was used to find the transition state geometries. Xylene was taken into account using the CPCM.

## Data Availability

Upon request, authors can provide original spectroscopic and analytic data.

## Supplementary Materials

The following supporting information can be downloaded at https://www.mdpi.com/article/10.3390/molecules30112271/s1, ^1^H and ^13^C NMR spectra of all products **4** and **6** and for theoretically examined cases coordinates of the optimized structures from the DFT calculations; imaginary frequencies and 3D representations of transition states; GEDT and direction of electron density flux and reaction coordinate diagrams.

## References

[1] Ram V.J., Goel A., Pratap R. (2022). Isolated Pyranones: Multifaceted Building Blocks for Molecular Diversity.

[2] Goel A., Ram V.J. (2009). Natural and synthetic 2*H*-pyran-2-ones and their versatility in organic synthesis. Tetrahedron.

[3] Nájera C., Sansano J.M., Yus M. (2021). Diels–Alder reactions of 1-amino-1,3-dienes and related systems. Tetrahedron.

[4] Kranjc K. (2022). 3-Acylamino-2*H*-pyran-2-ones as dienes in Diels–Alder reactions. Targets Heterocycl. Syst..

[5] Juranovič A., Kranjc K., Perdih F., Polanc S., Kočevar M. (2011). Comparison of the reaction pathways and intermediate products of a microwave-assisted and high-pressure-promoted cycloaddition of vinyl-moiety-containing dienophiles on 2*H*-pyran-2-ones. Tetrahedron.

[6] Afarinkia K., Abdullah M.H., Scowen I.J. (2010). A synthesis of carbasugar-sugar pseudodisaccharides via cycloaddition-cycloreversion reaction of 2*H*-pyran-2-ones. Org. Lett..

[7] Kranjc K., Juranovič A., Kočevar M., Perdih F. (2020). Supramolecular diversity of oxabicyclo[2.2.2]octenes formed between substituted 2*H*-pyran-2-ones and vinyl-moiety-containing dienophiles. Symmetry.

[8] Herlah B., Hoivik A., Jamšek L., Valjavec K., Yamamoto N., Hoshino T., Kranjc K., Perdih A. (2022). Design, synthesis and evaluation of fused bicyclo[2.2.2]octene as a potential core scaffold for the non-covalent inhibitors of SARS-CoV-2 3CL^pro^ main protease. Pharmaceuticals.

[9] Juranovič A., Kranjc K., Polanc S., Kočevar M. (2014). Cycloaddition of styrenes as phenylacetylene substitutes on 2*H*-pyran-2-ones and the consequent metal-free dehydrogenation: Case study of Boscalid derivatives. Synthesis.

[10] Suljagić J., Juranovič A., Krivec M., Kranjc K., Kočevar M. (2017). Chloranil as an efficient oxidant of the cyclohexadiene intermediates formed by a cycloaddition of substituted 2*H*-pyran-2-ones and styrenes en route to the Boscalid derivatives. J. Heterocycl. Chem..

[11] Okura K., Tamura R., Shigehara K., Masai E., Nakamura M., Otsuka Y., Katayama Y., Nakao Y. (2014). Synthesis of polysubstituted benzenes from 2-pyrone-4,6-dicarboxylic acid. Chem. Lett..

[12] Khatri A.I., Samant S.D. (2015). Facile, diversity-oriented, normal-electron-demand Diels–Alder reactions of 6-amino-2*H*-pyran-2-ones with diethyl acetylenedicarboxylate, 1,4-naphthoquinone, and *N*-phenylmaleimide. Synthesis.

[13] Hossaini Z., Rostami-Charati F., Sheikholeslami-Farahani F., Ghasemian M. (2015). Synthesis of functionalized benzene using Diels–Alder reaction of activated acetylenes with synthesized phosphoryl-2-oxo-2*H*-pyran. Z. Naturforsch..

[14] Komiyama T., Takaguchi Y., Tsuboi S. (2007). Synthesis of 4-arylthio-3-hydroxyphthalate by the Diels–Alder reaction of 4-arylthio-3-hydroxy-2-pyrone. Synth. Commun..

[15] Štefane B., Perdih A., Pevec A., Šolmajer T., Kočevar M. (2010). The participation of 2*H*-pyran-2-ones in [4+2] cycloadditions: An experimental and computational study. Eur. J. Org. Chem..

[16] Goldstein E., Kallel A., Beauchamp P.S. (1987). Theoretical study of the mechanism of the cycloaddition of acetylene to α-pyrone: Asynchronism and substituent effects. J. Mol. Struct. Theochem.

[17] Bickelhaupt F.M., Houk K.N. (2017). Analyzing reaction rates with the distortion/interaction activation strain model. Angew. Chem. Int. Ed..

[18] Bolotova I.A., Ustyuzhanin A.O., Sergeeva E.S., Faizdrakhmanova A.A., Hai Y., Stepanov A.V., Ushakov I.A., Lyssenko K.A., You L., Lvov A.G. (2023). 2,3-Diarylmaleate salts as a versatile class of diarylethenes with a full spectrum of photoactivity in water. Chem. Sci..

[19] Kraľovičová E., Krutošíková A., Kováč J., Dandárová M. (1986). Reactions of 2-aryl-4*H*-furo[3,2-*b*]pyrrole derivatives. Collect. Czech. Chem. Commun..

[20] Ueda H., Yamamoto R., Yamaguchi M., Tokuyama H. (2021). Synthesis of substituted anilines via a gold-catalyzed three-component reaction. Org. Biomol. Chem..

[21] Sopbué Fondjo E., Döpp D., Henkel G. (2006). Reactions of some anellated 2-aminothiophenes with electron poor acetylenes. Tetrahedron.

[22] Weaver M.G., Bai W.-J., Jackson S.K., Pettus T.R.R. (2014). Diels–Alder construction of regiodifferentiated *meta*-amino phenols and derivatives. Org. Lett..

[23] Krivec M., Perdih F., Košmrlj J., Kočevar M. (2016). Regioselective hydrolysis and transesterification of dimethyl 3-benzamidophthalates assisted by a neighboring amide group. J. Org. Chem..

[24] Kranjc K., Kočevar M. (2005). Diels–Alder reaction of highly substituted 2*H*-pyran-2-ones with alkynes: Reactivity and regioselectivity. N. J. Chem..

[25] Kranjc K., Kočevar M. (2006). Intensification of a reaction by the addition of a minor amount of solvent: Diels–Alder reaction of 2*H*-pyran-2-ones with alkynes. Collect. Czech. Chem. Commun..

[26] Kranjc K., Štefane B., Polanc S., Kočevar M. (2004). Synthesis of highly substituted aniline and *o*-phenylenediamine derivatives containing various substitution patterns. J. Org. Chem..

[27] Elbe H.-L., Dutzmann S., Stenzel K. (1998). Preparation of aminophthalic acid derivatives as pesticides. PCT Int. Appl. WO 9747589 A1, 1997. Chem. Abstr..

[28] Hiraiwa Y., Saito J., Watanabe T., Yamada M., Morinaka A., Fukushima T., Kudo T. (2014). X-ray crystallographic analysis of IMP-1 metallo-β-lactamase complexed with a 3-aminophthalic acid derivative, structure-based drug design, and synthesis of 3,6-disubstituted phthalic acid derivative inhibitors. Bioorg. Med. Chem. Lett..

[29] Kepe V., Kočevar M., Polanc S. (1996). One-pot synthesis of some 2*H*-pyran-2-one derivatives. J. Heterocycl. Chem..

[30] Al-Omran F., Al-Awadhi N., Khalik M.M.A., Kual K., El-Khair A.A., Elnagdi M.H. (1997). 1-Substituted 3-dimethylaminoprop-2-en-1-ones as building blocks in heterocyclic synthesis: Routes to 6-aryl- and 6-heteroaryl-2*H*-pyran-2-ones and 6- and 4-arylpyridin-2(1*H*)-ones. J. Chem. Res. Synopses.

[31] Hussein A.M., Abdel Hafiz I.S., Ishak E.A., Elnagdi M.H., Atalla A.A. (2008). Studies on enaminones: Synthesis and chemical reactivity of 4-dimethylamino-3-phenylbut-3-en-2-one. Org. Chem. Ind. J..

[32] Požgan F., Kranjc K., Kepe V., Polanc S., Kočevar M. (2007). Synthesis of 2*H*-pyran-2-ones and fused pyran-2-ones as useful building blocks. Arkivoc.

[33] Svete J., Čadež Z., Stanovnik B., Tišler M. (1990). Methyl 2-(benzoylamino)-3-(dimethylamino)propenoate in the synthesis of heterocyclic systems. The synthesis of substituted 3-benzoylamino-2*H*-pyran-2-ones. Synthesis.

[34] Kepe V., Kočevar M., Polanc S., Verček B., Tišler M. (1990). A simple and general one-pot synthesis of some 2*H*-pyran-2-ones and fused pyran-2-ones. Tetrahedron.

[35] Požgan F., Krejan M., Polanc S., Kočevar M. (2006). 5-Acyl-2*H*-pyran-2-ones in the Schmidt reaction: Migration of the pyran-2-one ring. Heterocycles.

[36] Kepe V., Kočevar M., Petrič A., Polanc S., Verček B. (1992). 4-(Ethoxymethylene)-2-phenyl-5(4*H*)-oxazolone as a synthon for the synthesis of some 2*H*-pyran-2-ones. Heterocycles.

[37] Behringer H., Falkenberg K. (1963). Syntheses with vinylogous acid halides. VI. Ring isomerization in the condensation of ethoxyalkylidene-2-phenyl-5-oxazolones with active methylene compounds. Chem. Ber..

[38] Ornik B., Stanovnik B., Tišler M. (1992). Methyl 2-benzoylamino-3-dimethylaminopropenoate in the synthesis of heterocyclic systems. A simple synthesis of amino derivatives of isomeric naphthopyranones and naphthodipyranones. J. Heterocycl. Chem..

[39] Kepe V., Polanc S., Kočevar M. (1998). A simple preparation of some 4-methyl-2*H*-pyran-2-ones. Heterocycles.

[40] Huntress E.H., Lesslie T.E., Bornstein J. (1952). Dimethyl acetylenedicarboxylate. Org. Synth..

[41] Servalli M., Gyr L., Sakamoto J., Schlueter A.D. (2015). Propeller-Shaped D3h-Symmetric Macrocycles with Three 1,8-Diazaanthracene Blades as Building Blocks for Photochemically Induced Growth Reactions. Eur. J. Org. Chem..

[42] Maturi M.M., Bach T. (2014). Enantioselective Catalysis of the Intramolecular [2+2] Photocycloaddition between 2-Pyridones and Acetylenedicarboxylates. Angew. Chem..

[43] Nisrine S., Coralie T., Blanco L., Deloisy S. (2011). Preparation of unsymmetrical dialkyl acetylenedicarboxylates and related esters by enzymatic transesterification. Tetrahedron Lett..

[44] Domingo L.R. (2014). A new C–C bond formation model based on the quantum chemical topology of electron density. RSC Adv..

[45] Domingo L.R., Sáez J.A. (2009). Understanding the mechanism of polar Diels-Alder reactions. Org. Biomol. Chem..

[46] Domingo L.R., Ríos-Gutiérrez M., Pérez P. (2017). How does the global electron density transfer diminish activation energies in polar cycloaddition reactions? A Molecular Electron Density Theory study. Tetrahedron.

[47] Sheldon R.A. (1992). Organic synthesis: Past, present and future. Chem. Ind..

[48] Sheldon R.A. (2023). The E factor at 30: A passion for pollution prevention. Green Chem..

[49] Neese F. (2022). Software update: The ORCA program system, version 5.0. WIRES Comput. Molec. Sci..

[50] Chai J.-D., Head-Gordon M. (2008). Long-range corrected hybrid density functionals with damped atom–atom dispersion corrections. Phys. Chem. Chem. Phys..

[51] Caldeweyher E., Ehlert S., Hansen A., Neugebauer H., Spicher S., Bannwarth C., Grimme S. (2019). A generally applicable atomic-charge dependent London dispersion correction. J. Chem. Phys..

[52] Weigend F., Ahlrichs R. (2005). Balanced basis sets of split valence, triple zeta valence and quadruple zeta valence quality for H to Rn: Design and assessment of accuracy. Phys. Chem. Chem. Phys..

[53] Weigend F. (2006). Accurate Coulomb-fitting basis sets for H to Rn. Phys. Chem. Chem. Phys..

[54] Bursch M., Mewes J.-M., Hansen A., Grimme S. (2022). Best-Practice DFT Protocols for Basic Molecular Computational Chemistry. Angew. Chem. Int. Ed..

[55] Mezei P.D., Csonka G.I., Kállay M. (2015). Accurate Diels–Alder Reaction Energies from Efficient Density Functional Calculations. J. Chem. Theory Comput..

[56] Barone V., Cossi M. (1998). Quantum calculation of molecular energies and energy gradients in solution by a conductor solvent model. J. Phys. Chem. A.

[57] Riplinger C., Pinski P., Becker U., Valeev E.F., Neese F. (2016). Sparse maps–A systematic infrastructure for reduced-scaling electronic structure methods. II. Linear scaling domain based pair natural orbital coupled cluster theory. J. Chem. Phys..

[58] Hellweg A., Hättig C., Höfener S., Klopper W. (2007). Optimized accurate auxiliary basis sets for RI-MP2 and RI-CC2 calculations for the atoms Rb to Rn. Theor. Chem. Acc..

[59] Ásgeirsson V., Birgisson B.O., Bjornsson R., Becker U., Neese F., Riplinger C., Jónsson H. (2021). Nudged elastic band method for molecular reactions using energy-weighted springs combined with eigenvector following. J. Chem. Theory Comput..

