# Supplementary material for: Towards Greener Synthesis of Substituted 3-Aminophthalates Starting from 2H-Pyran-2-ones via Diels–Alder Reaction of Acetylenedicarboxylates
Source: Molecules. 2025 May 22;30(11):2271. doi: 10.3390/molecules30112271 (PMC12156201; doi:10.3390/molecules30112271)
Supplement: Supplementary file 1 [file molecules-30-02271-s001.zip › molecules-3634831-supplementary.pdf]

Towards greener synthesis of substituted 3-aminophthalates starting from 2*H*-pyran-2-ones *via* Diels–Alder reaction of acetylenedicarboxylates

Dominik Fendre [1], Miha Lukšič [1] and Krištof Kranjc [1,*]

[1] Faculty of Chemistry and Chemical Technology, University of Ljubljana, Večna pot 113, SI-1000 Ljubljana, Slovenia
* Correspondence: kristof.kranjc@fkkt.uni-lj.si; Tel.: +386 (0) 1 4798563

Supplementary material

^{1}H and ^{13}C NMR spectra of products **4** S2

Quantum-chemical calculation results S64

4Aa

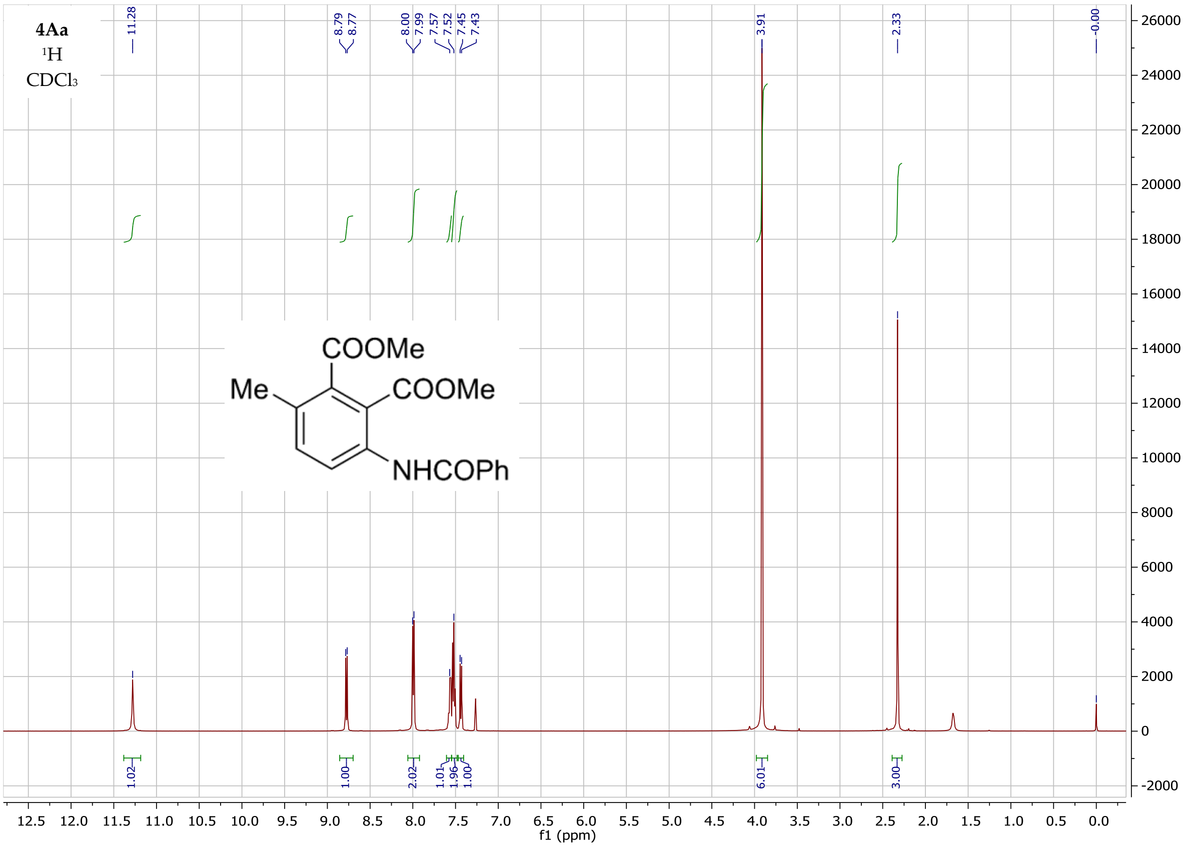
4Aa
^{1}H
CDCl3
11.28
8.79
8.77
8.00
7.99
7.57
7.52
7.45
7.43
3.91
2.33
-0.00
1.02
1.00
2.02
1.01
1.96
1.00
6.01
3.00
COOMe
Me
COOMe
NHCOPh
f1 (ppm)

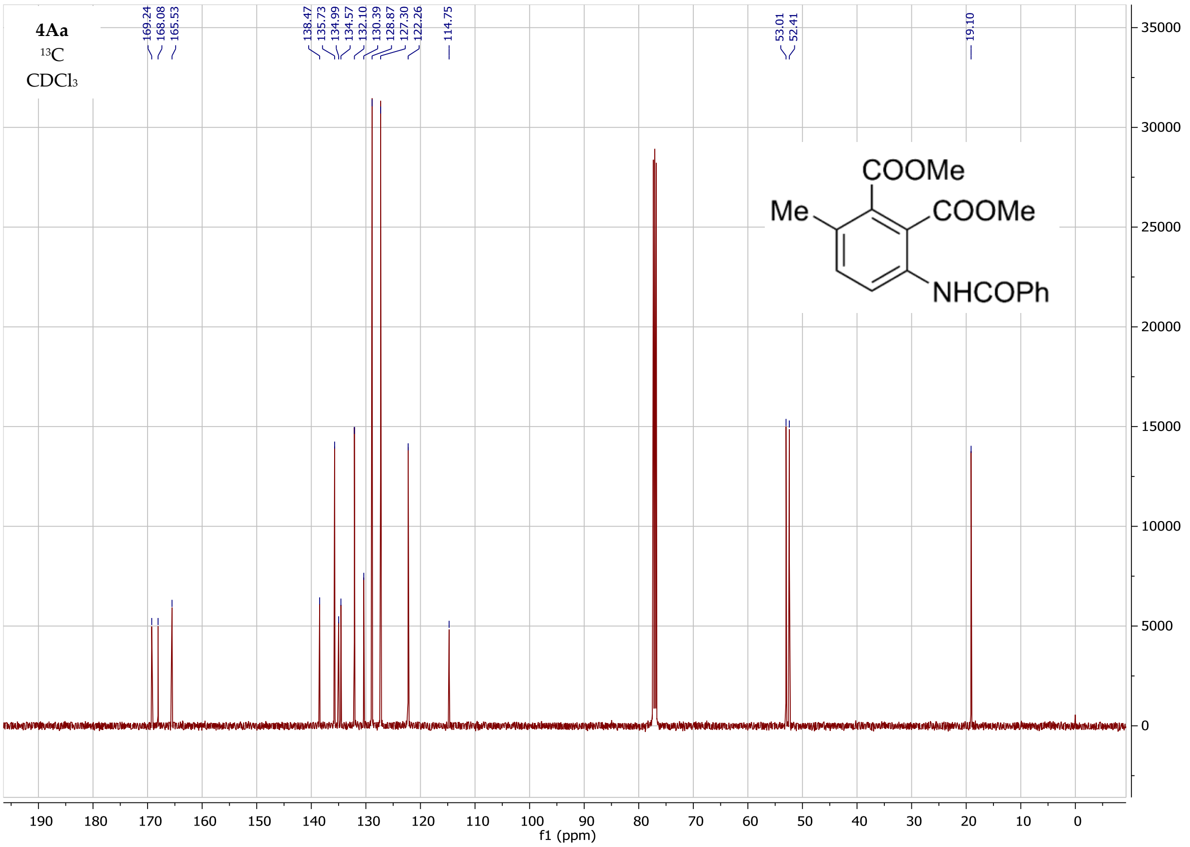
4Aa
^{13}C
CDCl3
169.24
168.08
165.53
138.47
135.73
134.99
134.57
132.10
130.39
128.87
127.30
122.26
114.75
53.01
52.41
19.10
COOMe
Me
COOMe
NHCOPh
f1 (ppm)

4Ab

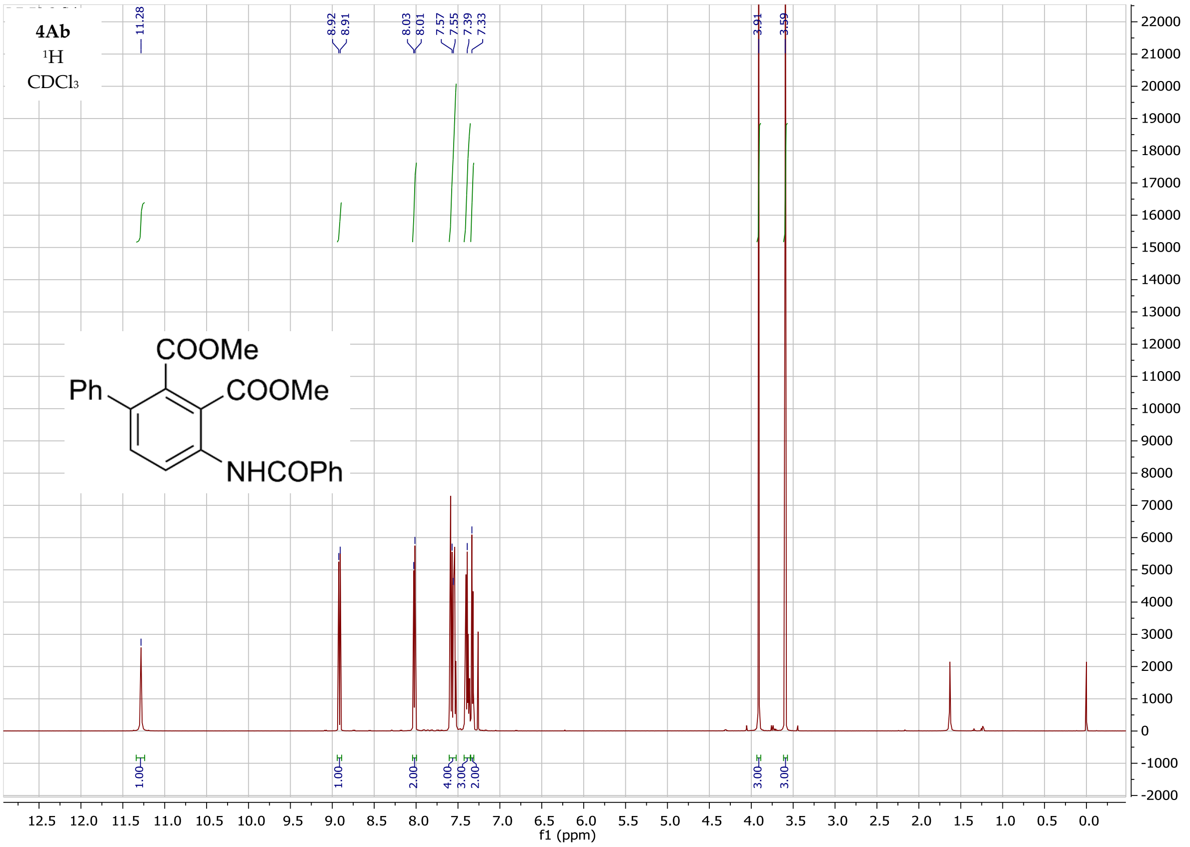

4Ab
1H
CDCl3
11.28
8.92
8.91
8.03
8.01
7.57
7.55
7.39
7.33
3.91
3.59
COOMe
Ph
COOMe
NHCOPh
1.00
1.00
2.00
4.00
3.00
2.00
3.00
3.00
f1 (ppm)

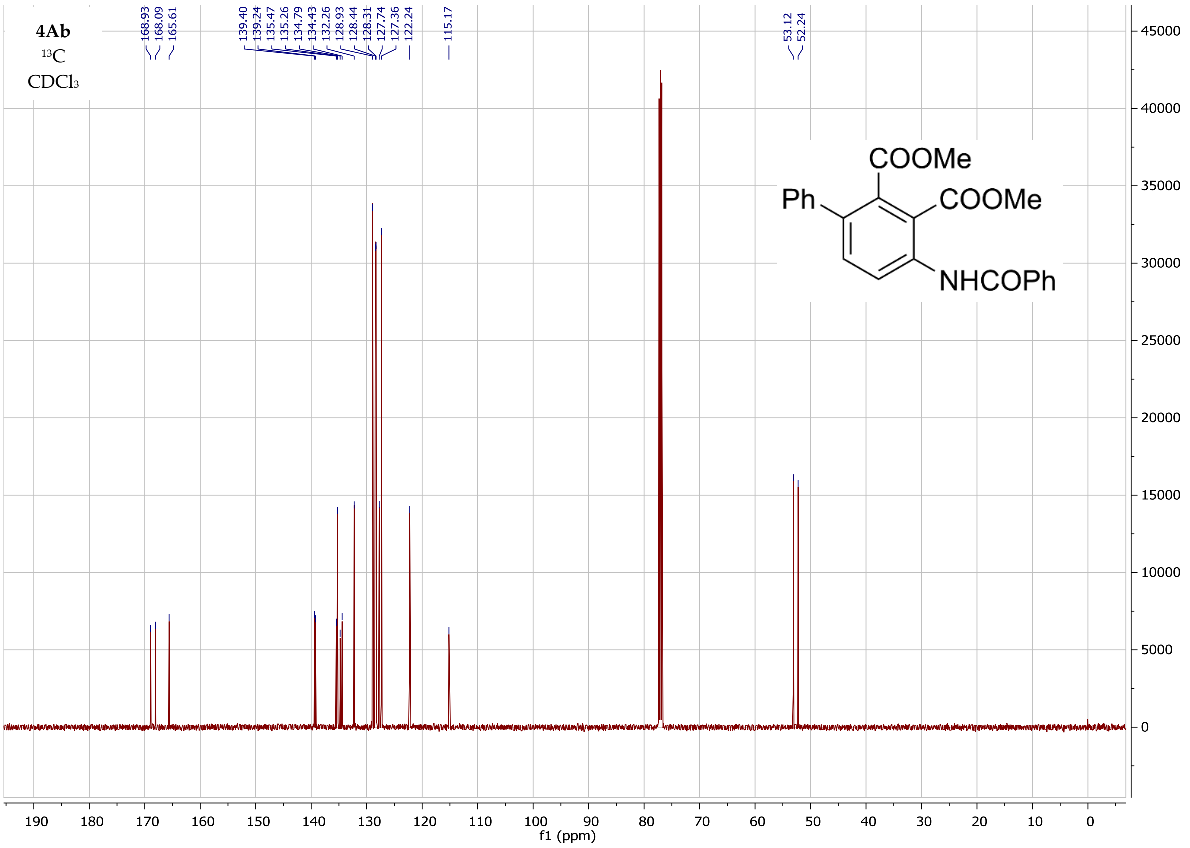

4Ab
13C
CDCl3
168.93
168.09
165.61
139.40
139.24
135.47
135.26
134.79
134.43
132.26
128.93
128.44
128.31
127.74
127.36
122.24
115.17
53.12
52.24
COOMe
Ph
COOMe
NHCOPh
f1 (ppm)

4Ac

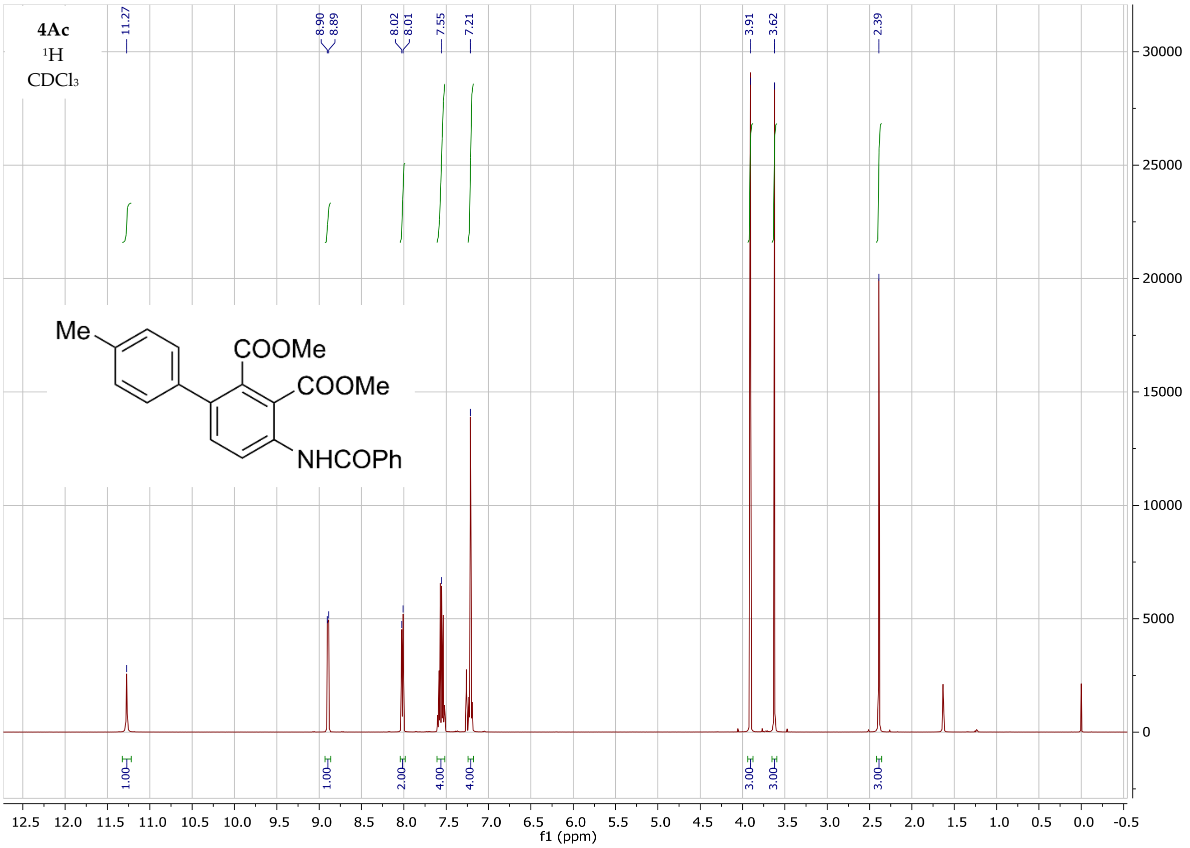

4Ac
1H
CDCl3
11.27
8.90
8.89
8.02
8.01
7.55
7.21
3.91
3.62
2.39
1.00
1.00
2.00
4.00
4.00
3.00
3.00
3.00
Me
COOMe
COOMe
NHCOPh
f1 (ppm)

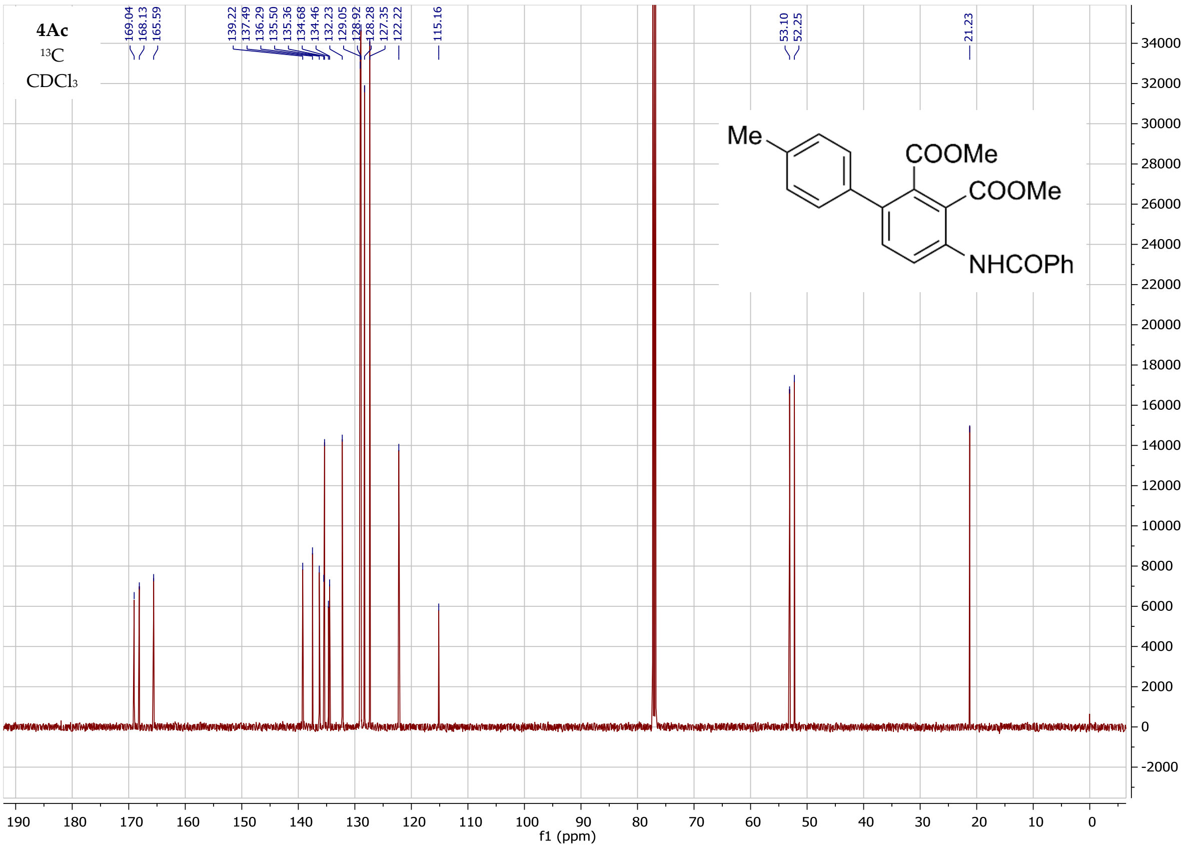

4Ac
13C
CDCl3
169.04
168.13
165.59
139.22
137.49
136.29
135.50
135.36
134.68
134.46
132.23
129.05
128.92
128.28
127.35
122.22
115.16
53.10
52.25
21.23
Me
COOMe
COOMe
NHCOPh
f1 (ppm)

4Ad

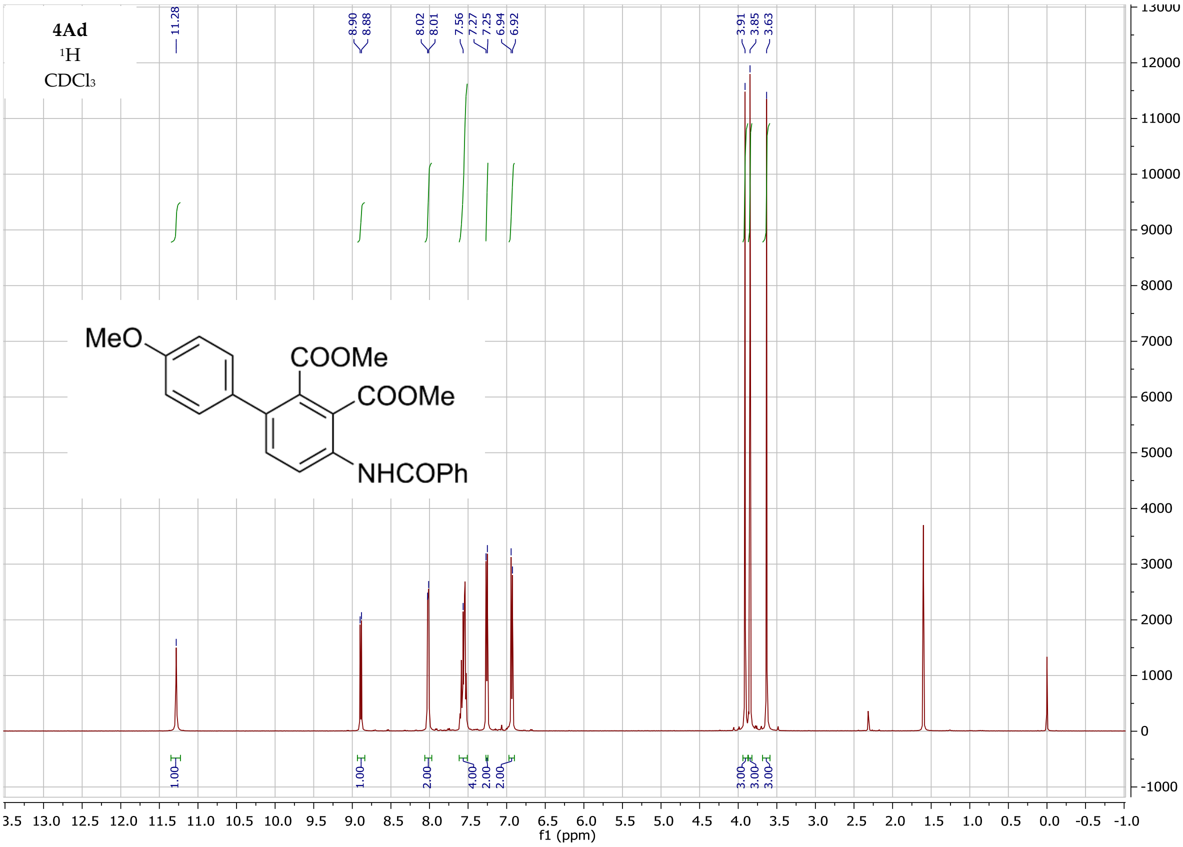
4Ad
1H
CDCl3
11.28
8.90
8.88
8.02
8.01
7.56
7.27
7.25
6.94
6.92
3.91
3.85
3.63
MeO
COOMe
COOMe
NHCOPh
1.00
1.00
2.00
4.00
2.00
2.00
3.00
3.00
3.00
f1 (ppm)

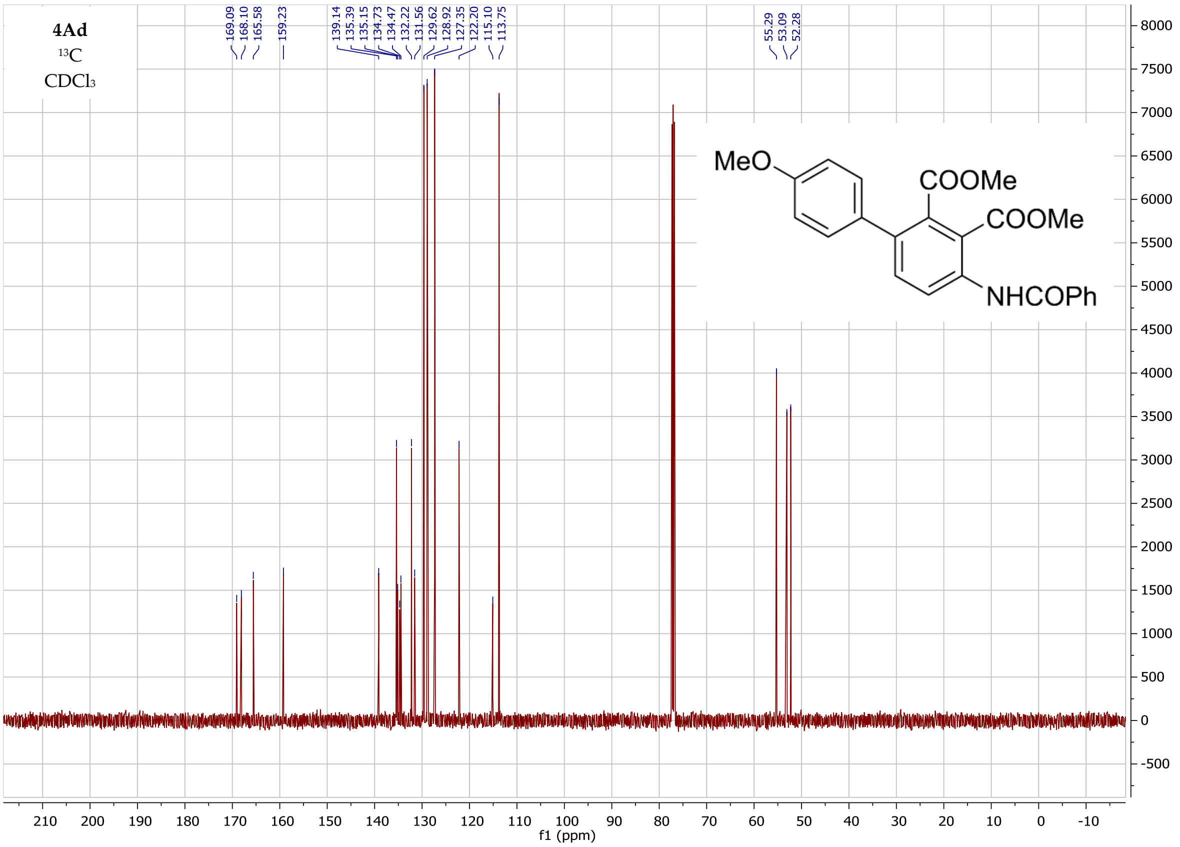
4Ad
13C
CDCl3
169.09
168.10
165.58
159.23
139.14
135.39
135.15
134.73
134.47
132.22
131.56
129.62
128.92
127.35
122.20
115.10
113.75
55.29
53.09
52.28
MeO
COOMe
COOMe
NHCOPh
f1 (ppm)

4Ae

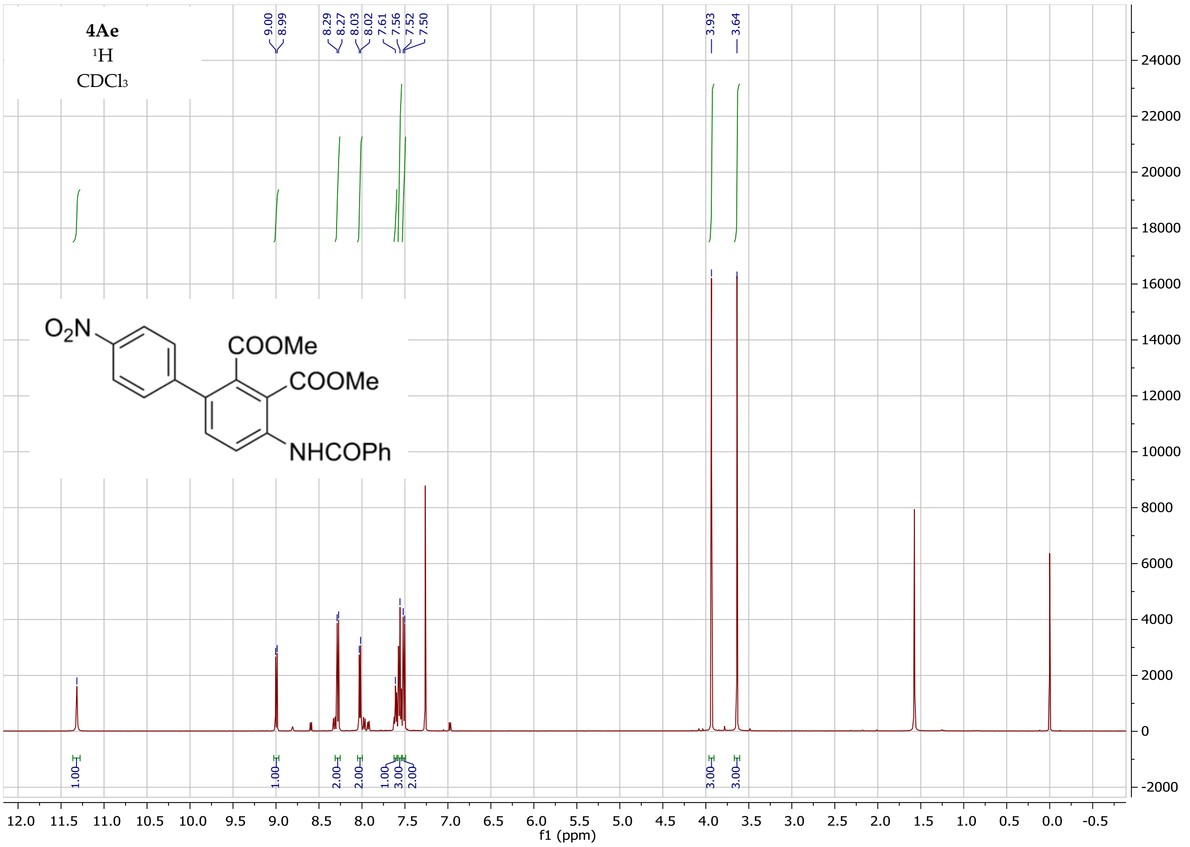

4Ae
1H
CDCl3
9.00
8.99
8.29
8.27
8.03
8.02
7.61
7.56
7.52
7.50
3.93
3.64
1.00
1.00
2.00
2.00
1.00
3.00
2.00
3.00
3.00
f1 (ppm)

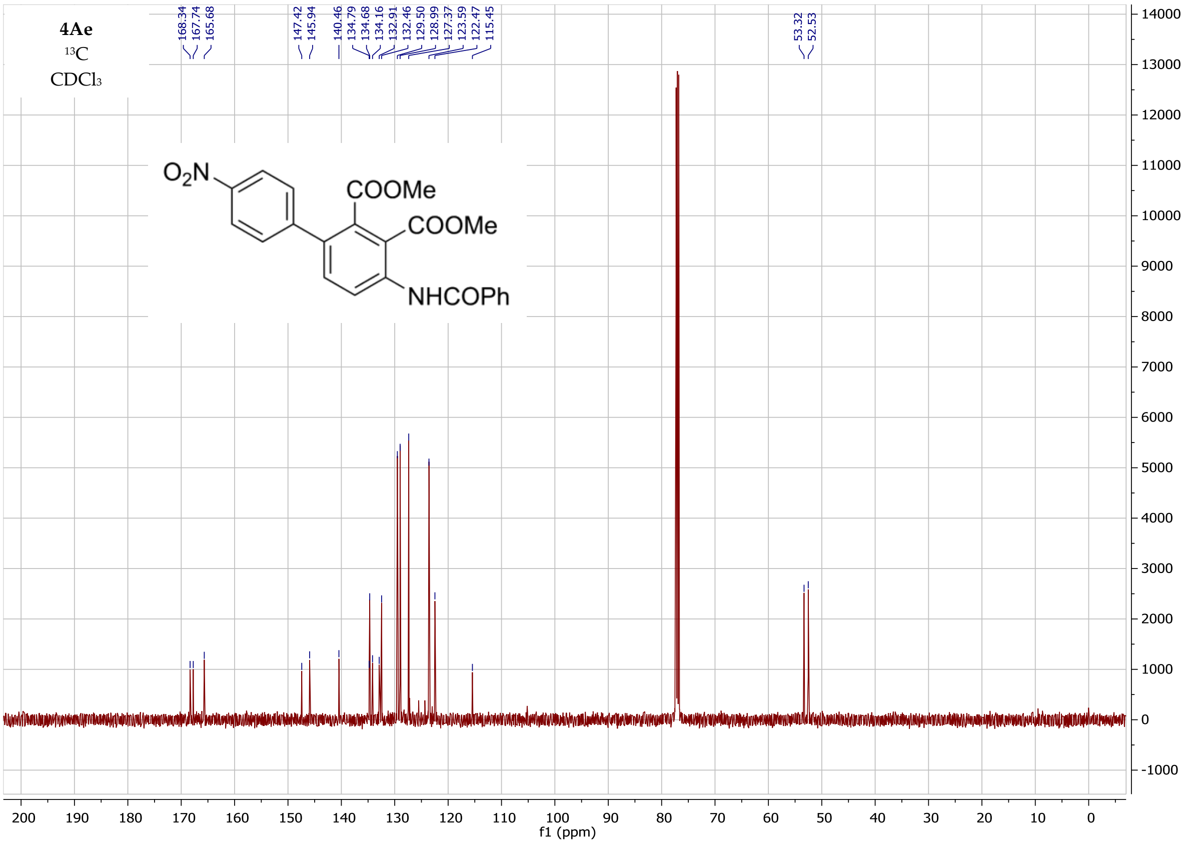

4Ae
13C
CDCl3
168.34
167.74
165.68
147.42
145.94
140.46
134.79
134.68
134.16
132.91
132.46
129.50
128.99
127.37
123.59
122.47
115.45
53.32
52.53
f1 (ppm)

4Af

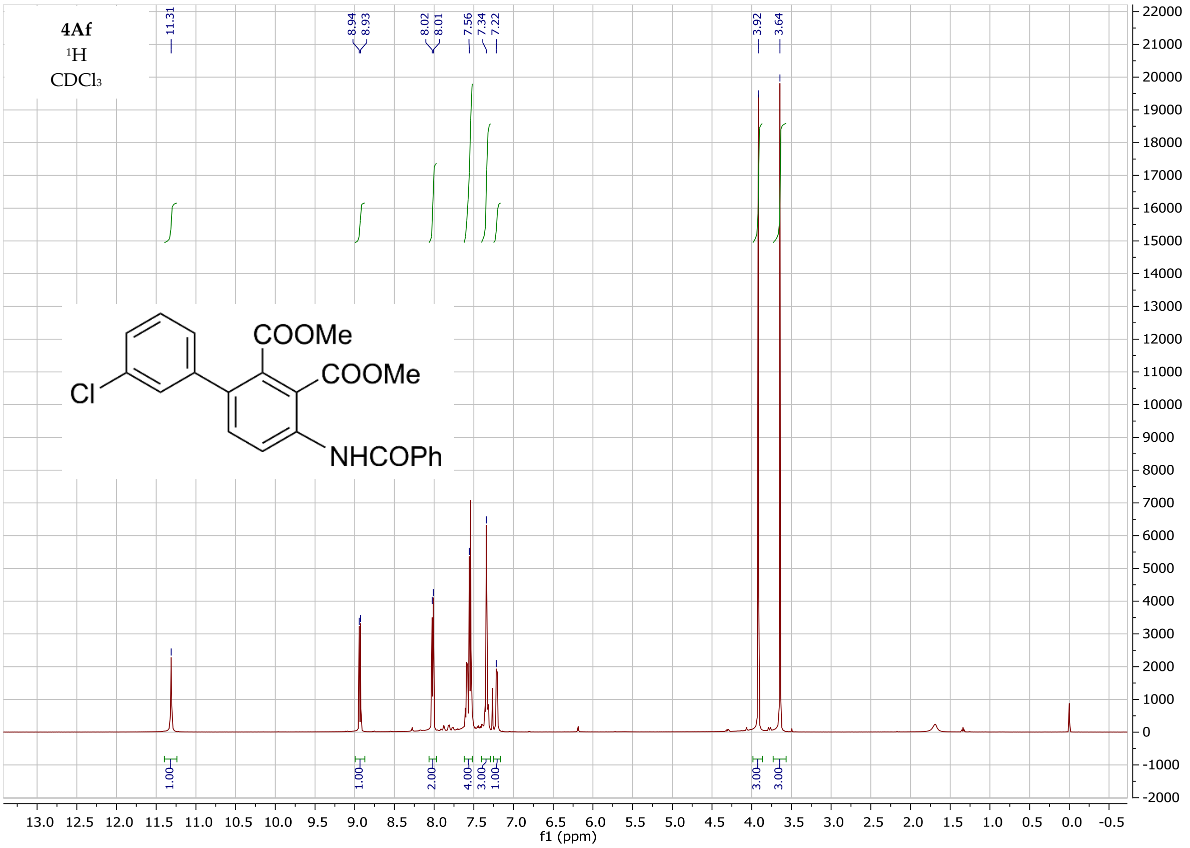

4Af
¹H
CDCl3
11.31
8.94
8.93
8.02
8.01
7.56
7.34
7.22
3.92
3.64
COOMe
COOMe
Cl
NHCOPh
1.00
1.00
2.00
4.00
3.00
1.00
3.00
3.00
f1 (ppm)

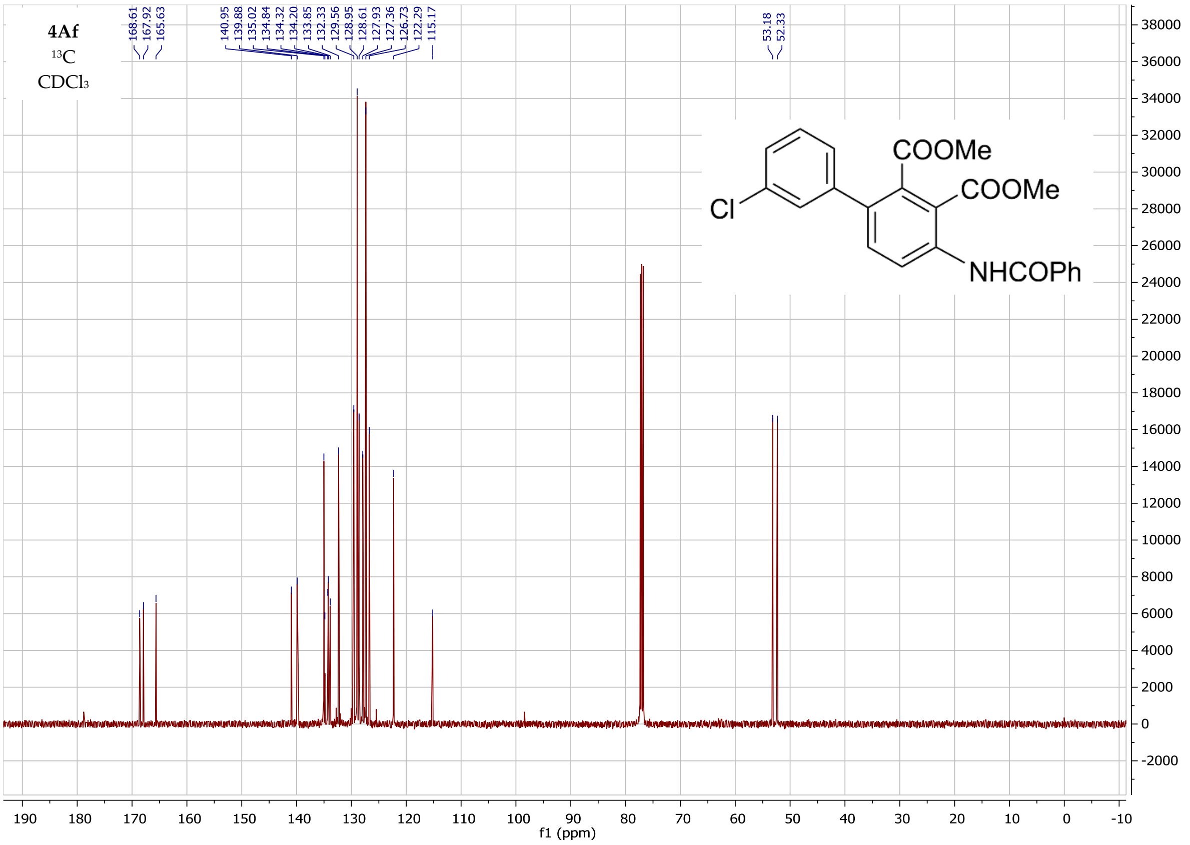

4Af
¹³C
CDCl3
168.61
167.92
165.63
140.95
139.88
135.02
134.84
134.32
134.20
133.85
132.33
129.56
128.95
128.61
127.93
127.36
126.73
122.29
115.17
53.18
52.33
COOMe
COOMe
Cl
NHCOPh
f1 (ppm)

4Ag

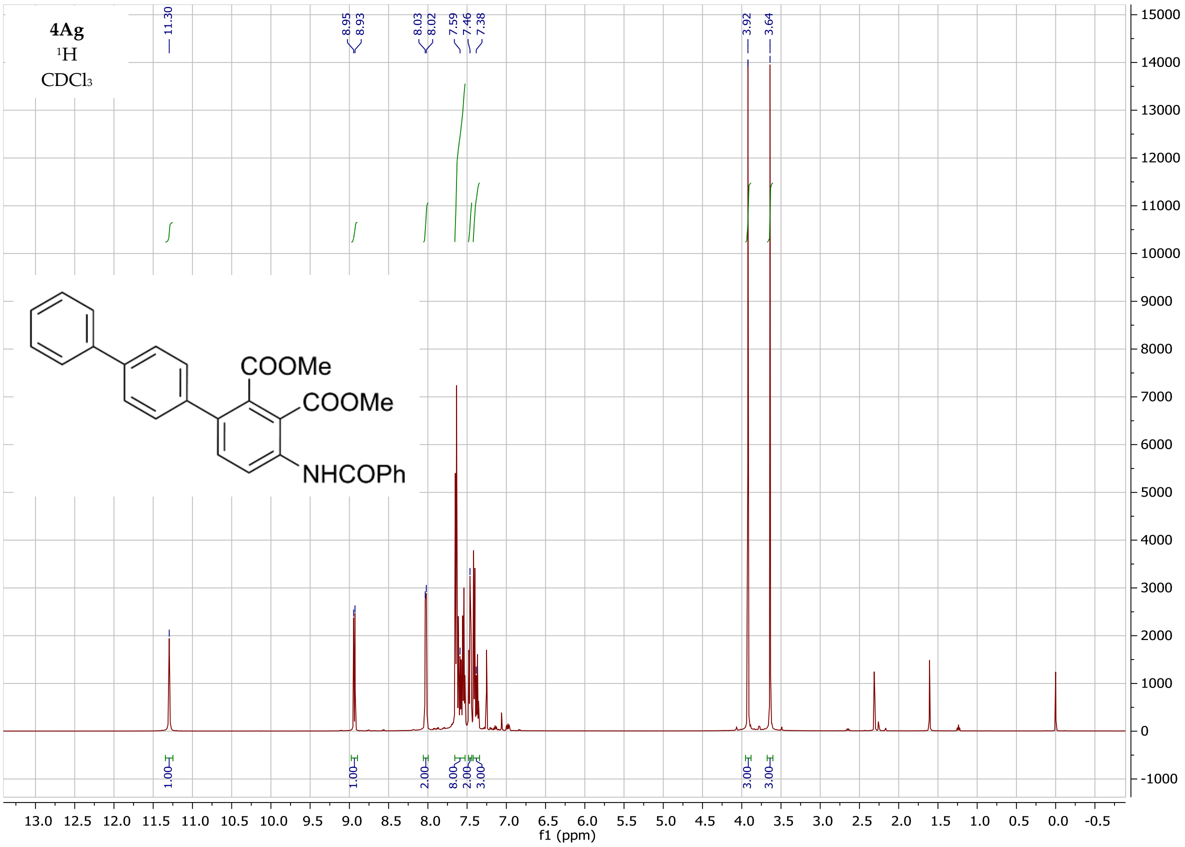
4Ag
1H
CDCl3
11.30
8.95
8.93
8.03
8.02
7.59
7.46
7.38
3.92
3.64
COOMe
COOMe
NHCOPh
1.00
1.00
2.00
8.00
2.00
3.00
3.00
3.00
f1 (ppm)

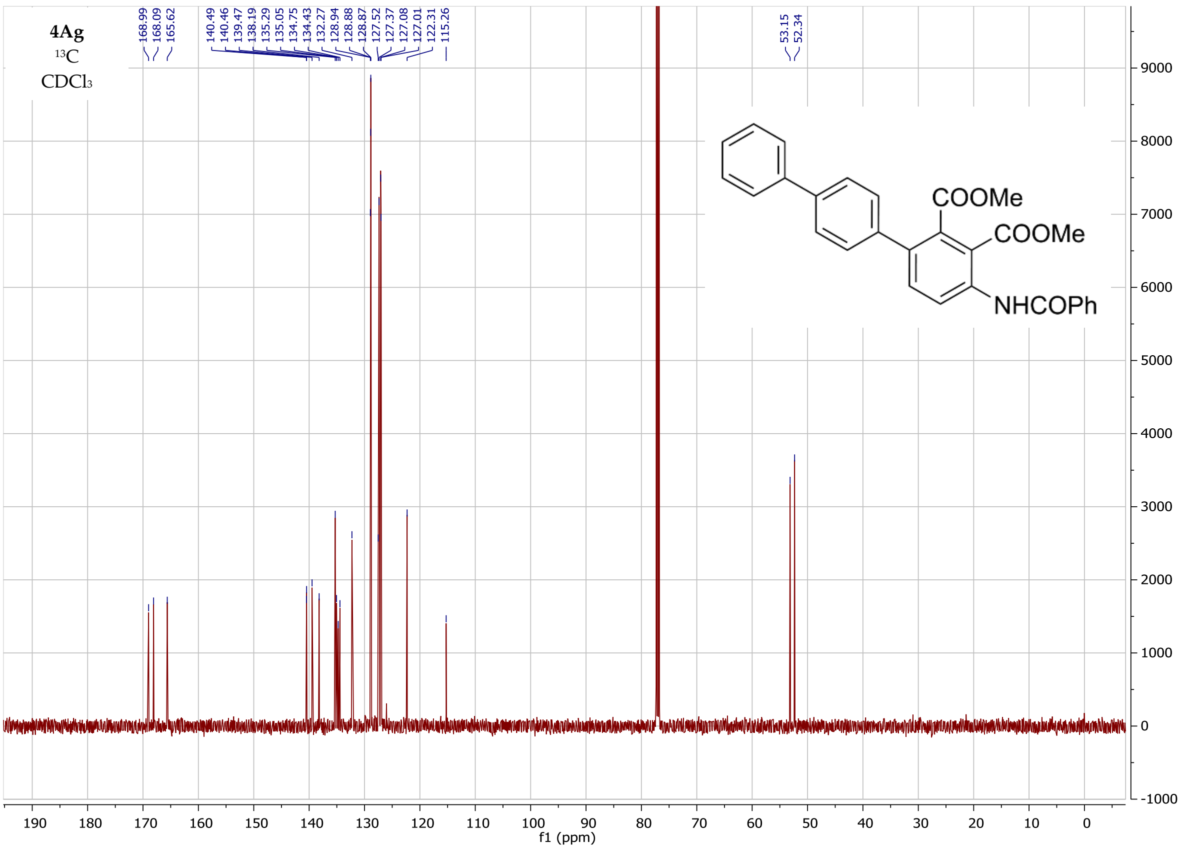
4Ag
13C
CDCl3
168.99
168.09
165.62
140.49
140.46
139.47
138.19
135.29
135.05
134.75
134.43
132.27
128.94
128.88
128.87
127.52
127.37
127.08
127.01
122.31
115.26
53.15
52.34
COOMe
COOMe
NHCOPh
f1 (ppm)

4Ah

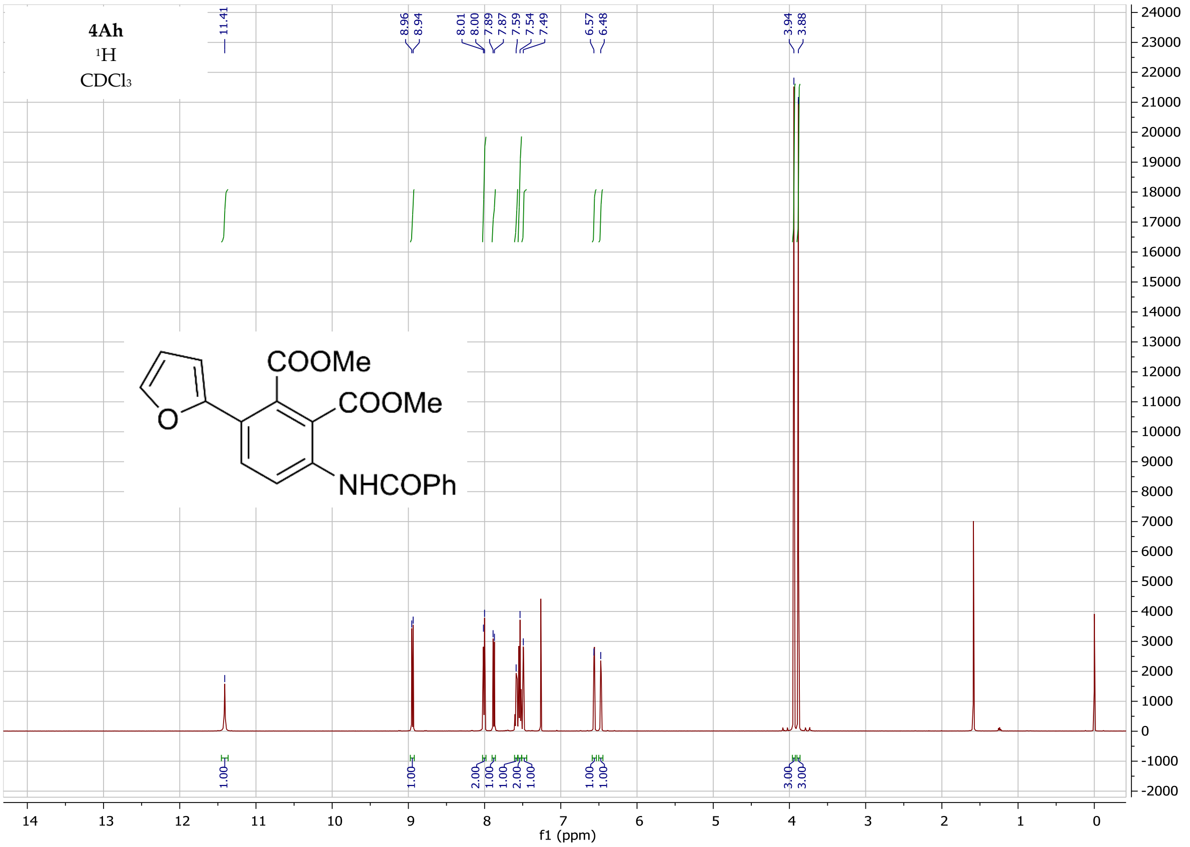
4Ah
1H
CDCl3
11.41
8.96
8.94
8.01
8.00
7.89
7.87
7.59
7.54
7.49
6.57
6.48
3.94
3.88
COOMe
COOMe
O
NHCOPh
1.00
1.00
2.00
1.00
1.00
2.00
1.00
1.00
1.00
3.00
3.00
f1 (ppm)

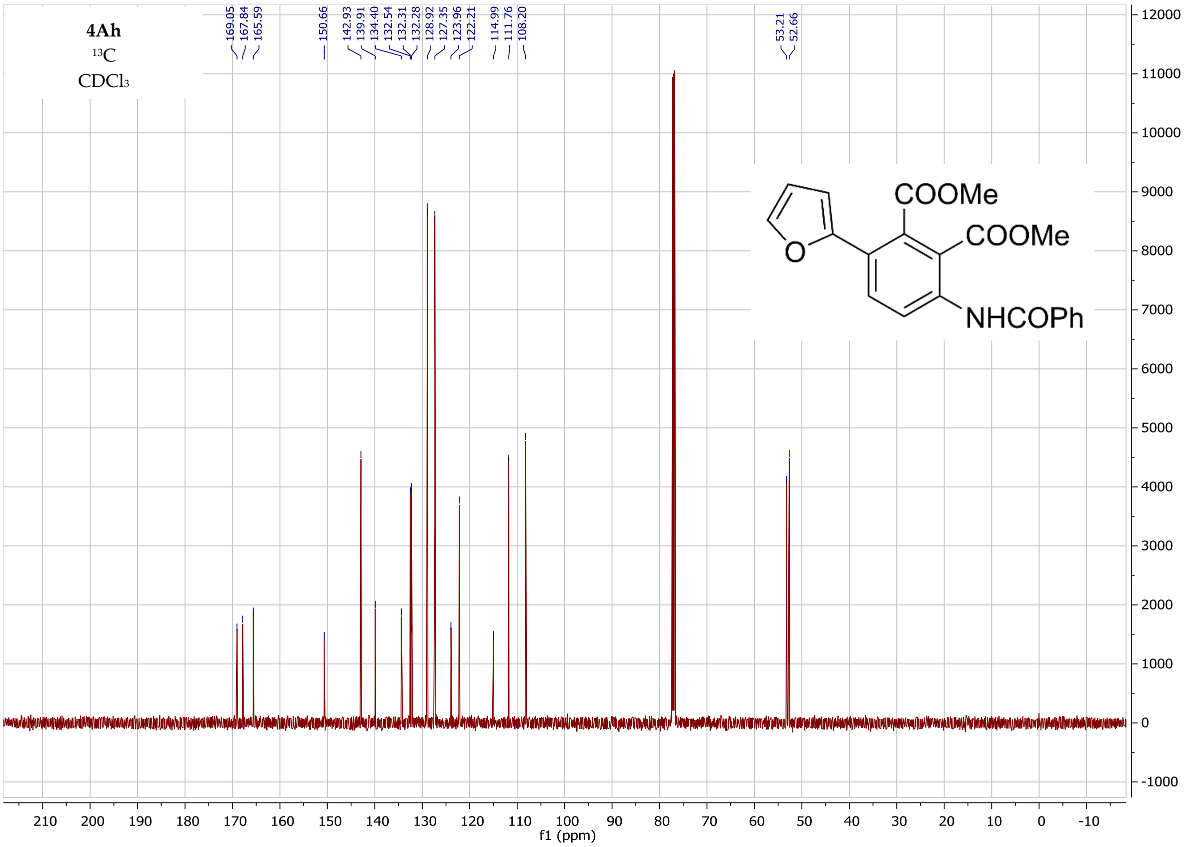
4Ah
13C
CDCl3
169.05
167.84
165.59
150.66
142.93
139.91
134.40
132.54
132.31
132.28
128.92
127.35
123.96
122.21
114.99
111.76
108.20
53.21
52.66
COOMe
COOMe
O
NHCOPh
f1 (ppm)

4Ai

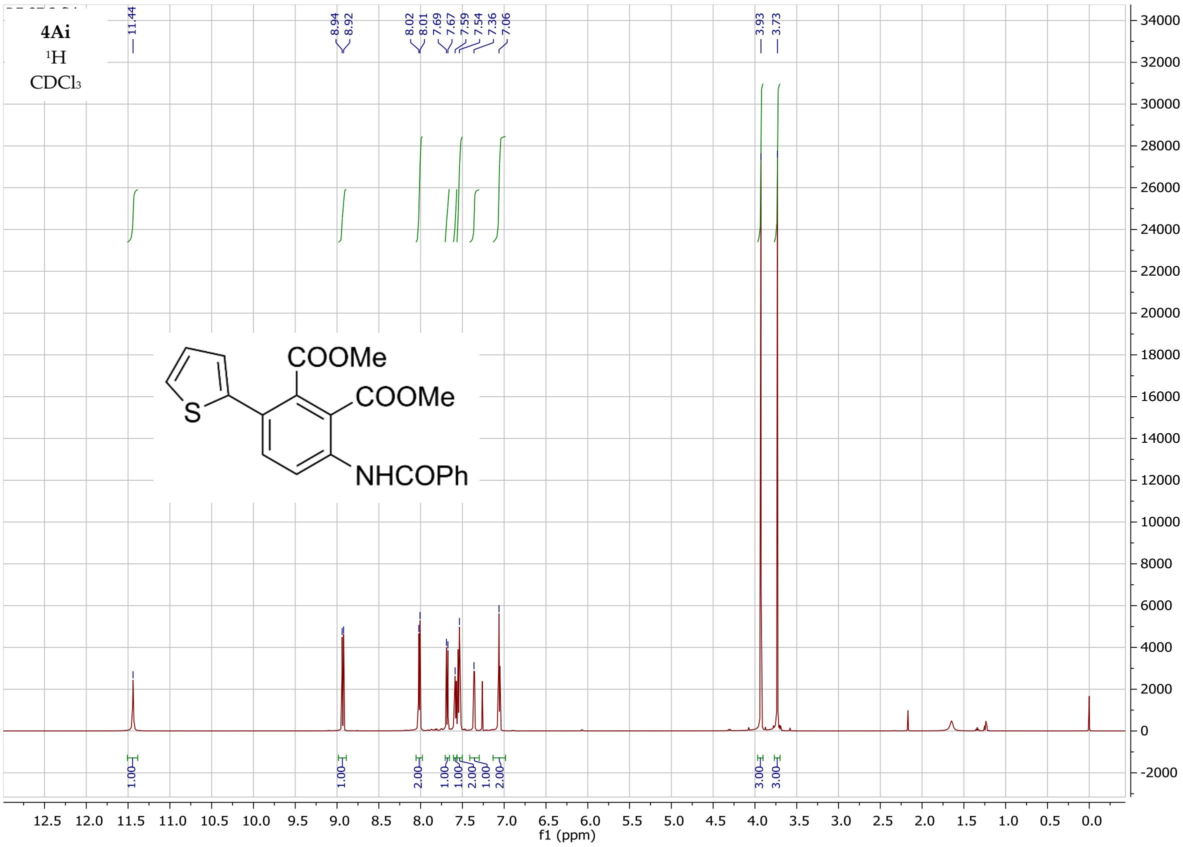
4Ai
1H
CDCl3
11.44
8.94
8.92
8.02
8.01
7.69
7.67
7.59
7.54
7.36
7.06
3.93
3.73
COOMe
COOMe
S
NHCOPh
1.00
1.00
2.00
1.00
1.00
2.00
1.00
2.00
3.00
3.00
f1 (ppm)

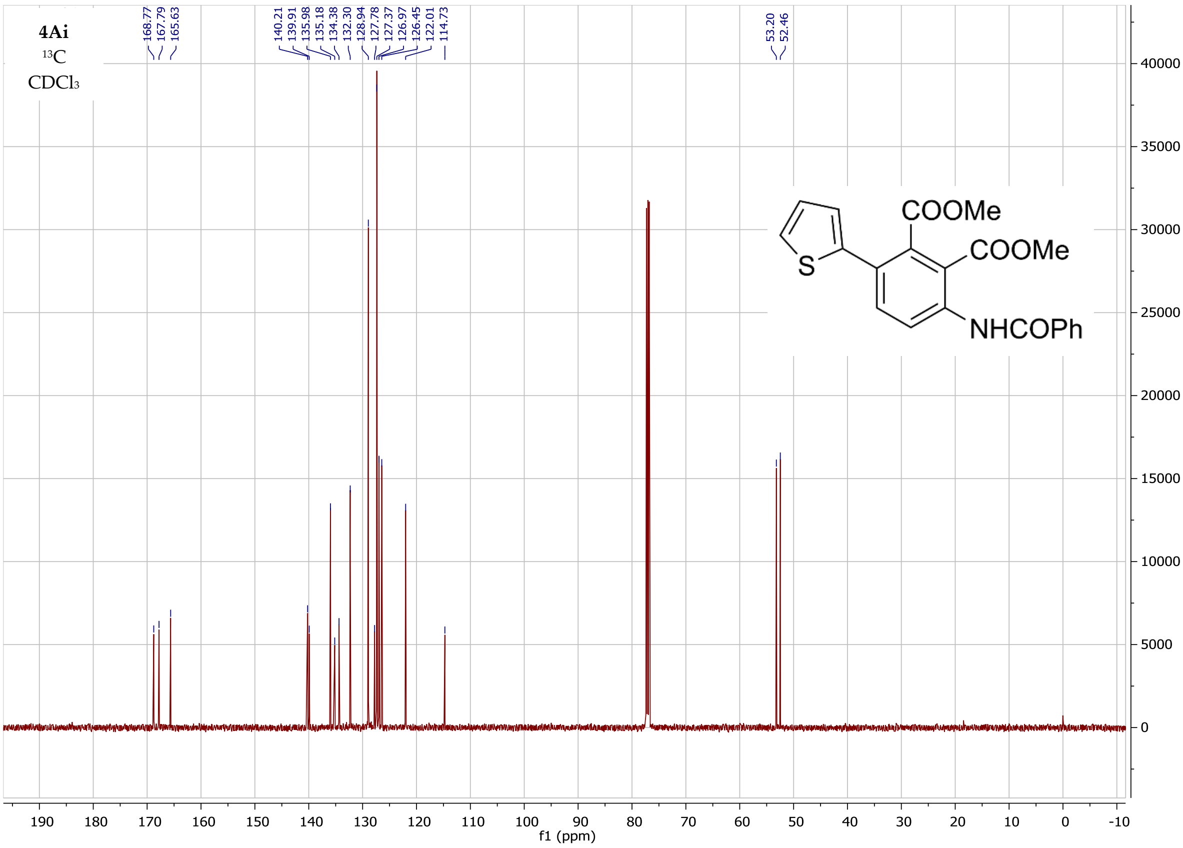
4Ai
13C
CDCl3
168.77
167.79
165.63
140.21
139.91
135.98
135.18
134.38
132.30
128.94
127.78
127.37
126.97
126.45
122.01
114.73
53.20
52.46
COOMe
COOMe
S
NHCOPh
f1 (ppm)

4Aj

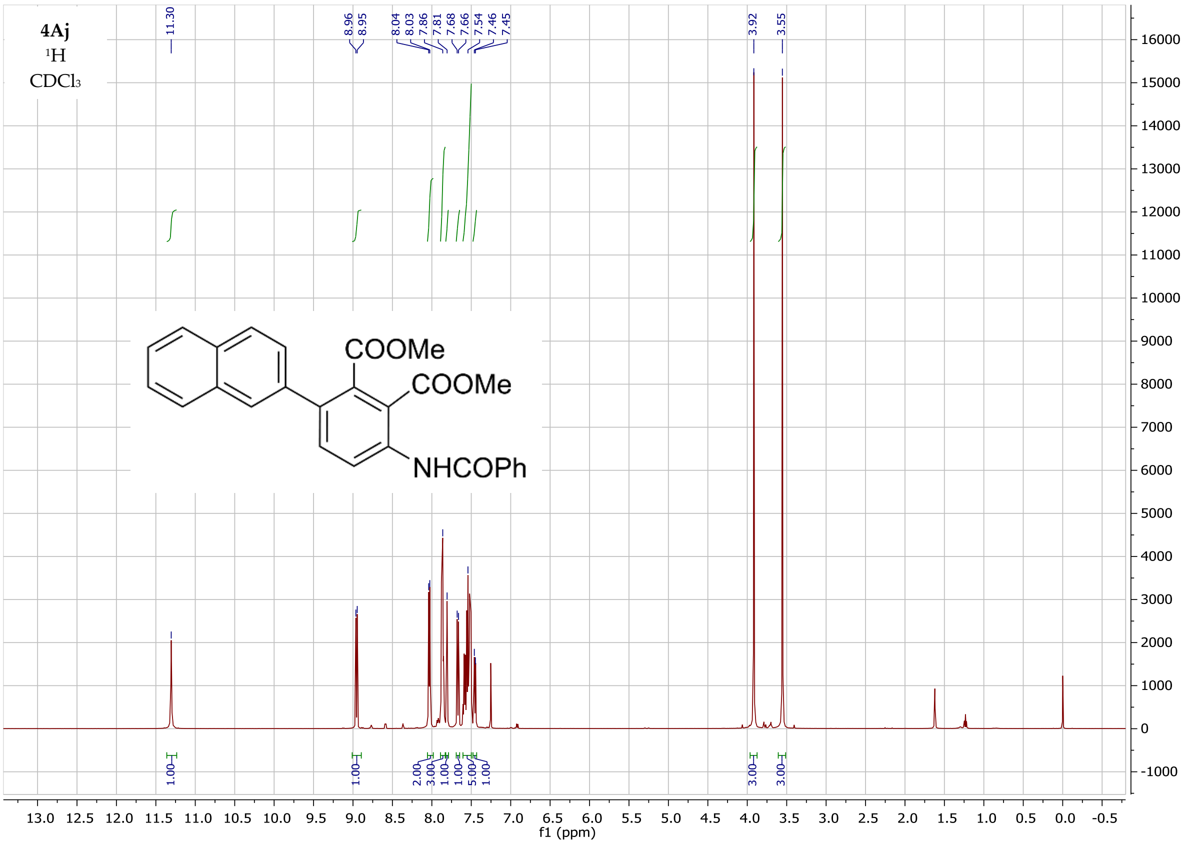
4Aj
1H
CDCl3
11.30
8.96
8.95
8.04
8.03
7.86
7.81
7.68
7.66
7.54
7.46
7.45
3.92
3.55
COOMe
COOMe
NHCOPh
1.00
1.00
2.00
3.00
1.00
1.00
5.00
1.00
3.00
3.00
f1 (ppm)

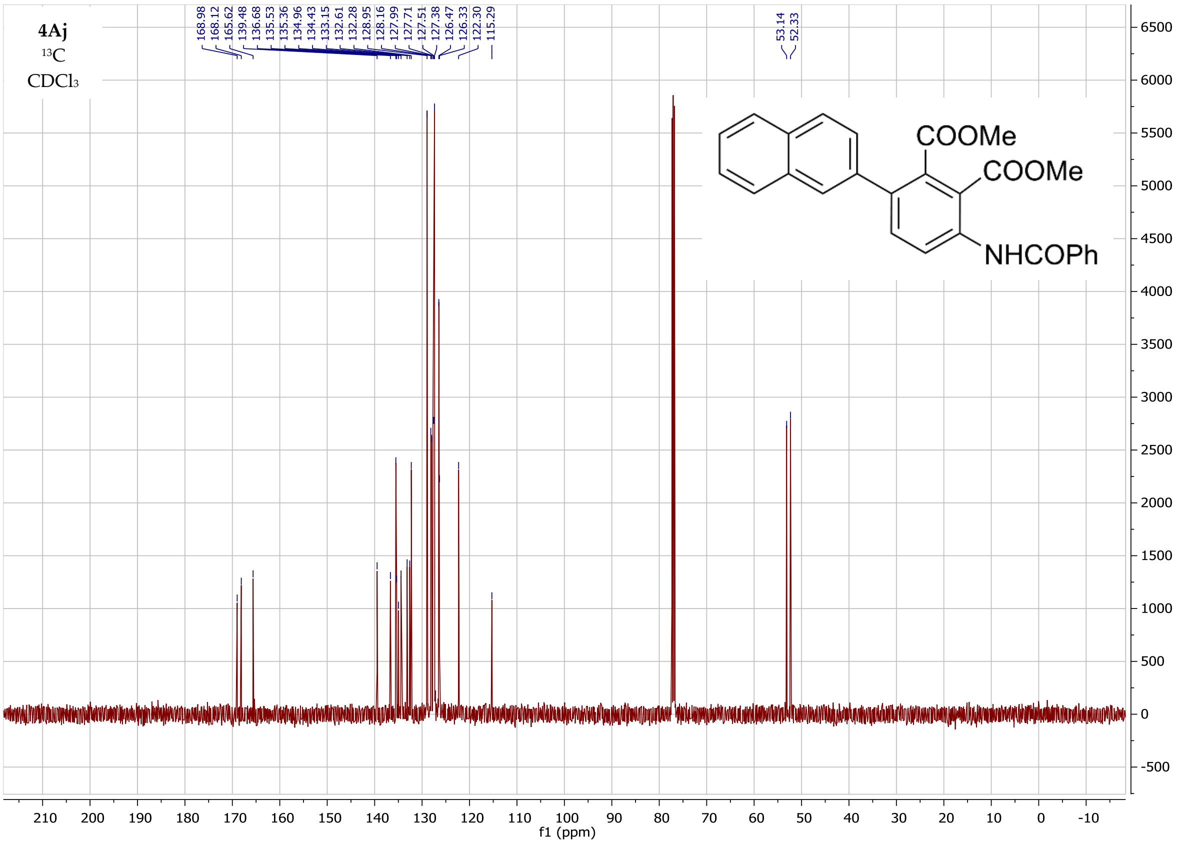
4Aj
13C
CDCl3
168.98
168.12
165.62
139.48
136.68
135.53
135.36
134.96
134.43
133.15
132.61
132.28
128.95
128.16
127.99
127.71
127.51
127.38
126.47
126.33
122.30
115.29
53.14
52.33
COOMe
COOMe
NHCOPh
f1 (ppm)

4Ak

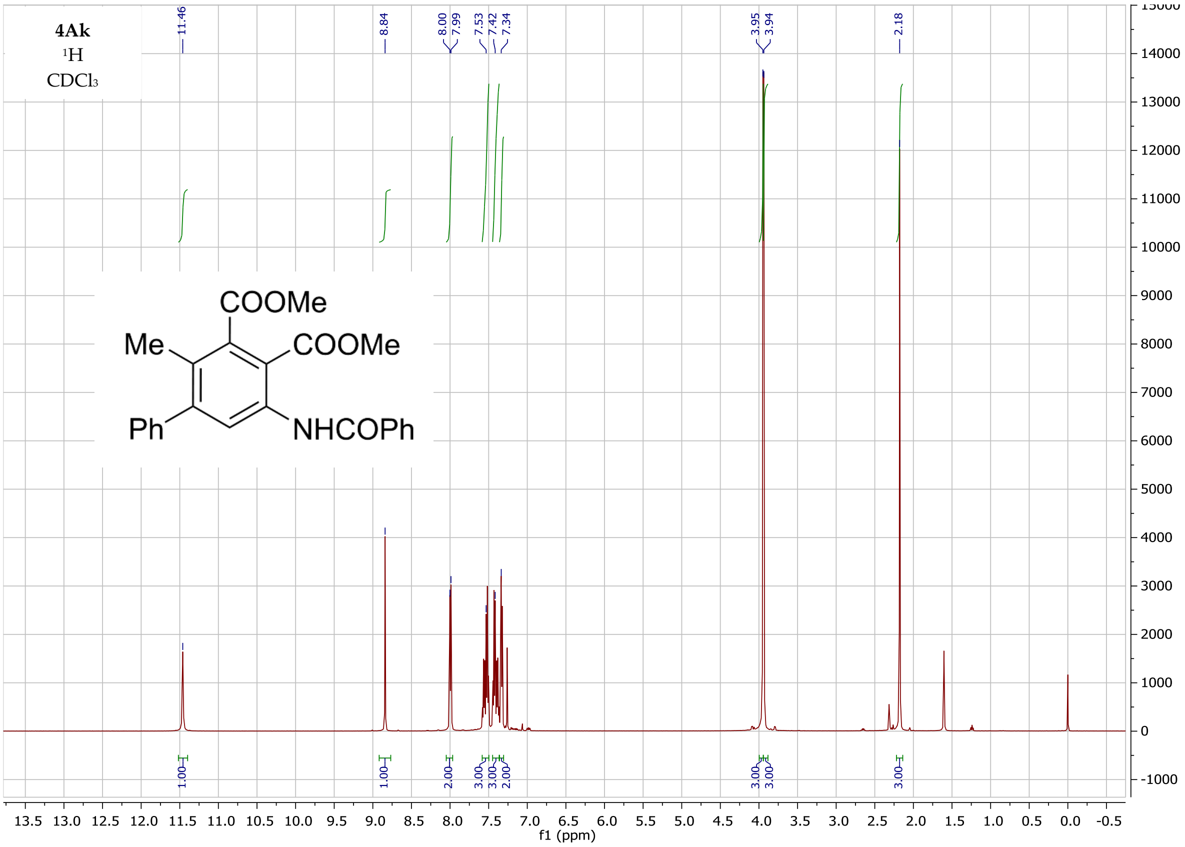
4Ak
¹H
CDCl3
11.46
8.84
8.00
7.99
7.53
7.42
7.34
3.95
3.94
2.18
COOMe
Me
COOMe
Ph
NHCOPh
1.00
1.00
2.00
3.00
3.00
2.00
3.00
3.00
3.00
f1 (ppm)

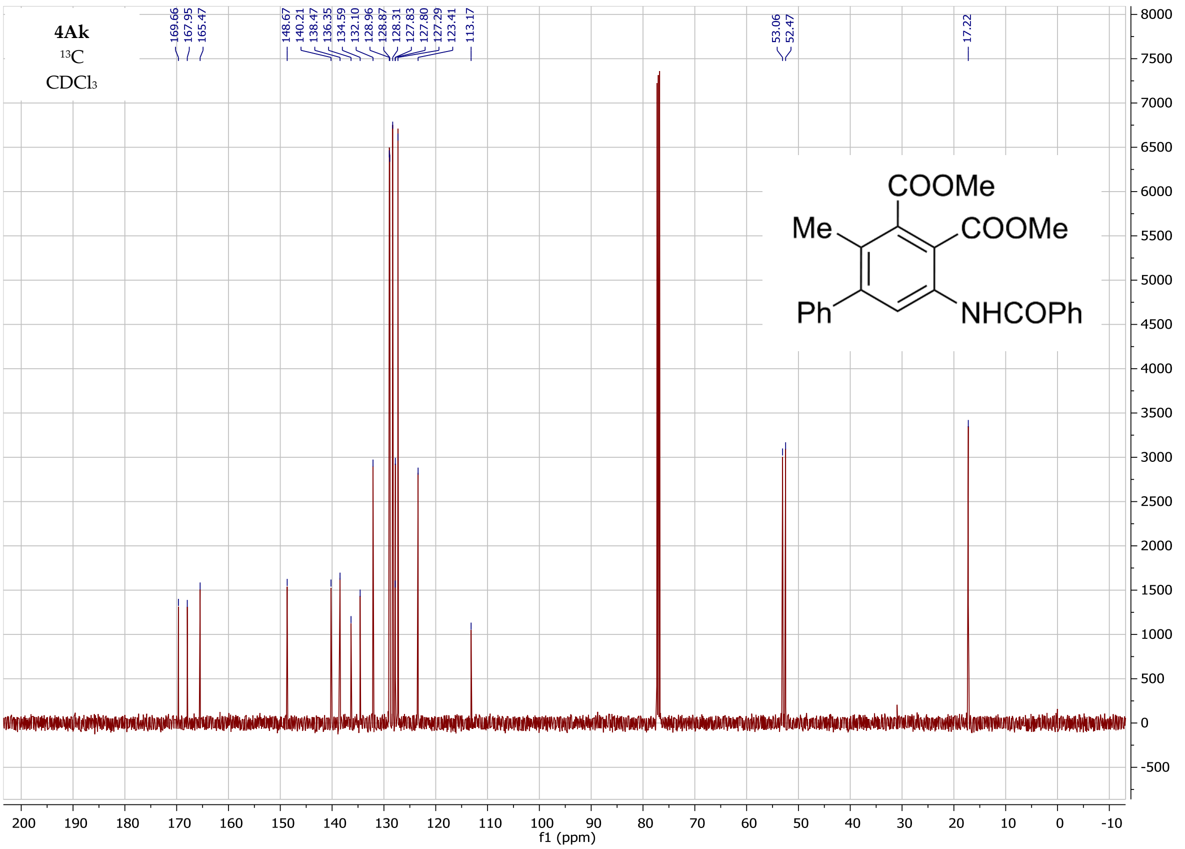
4Ak
¹³C
CDCl3
169.66
167.95
165.47
148.67
140.21
138.47
136.35
134.59
132.10
128.96
128.87
128.31
127.83
127.80
127.29
123.41
113.17
53.06
52.47
17.22
COOMe
Me
COOMe
Ph
NHCOPh
f1 (ppm)

4Al

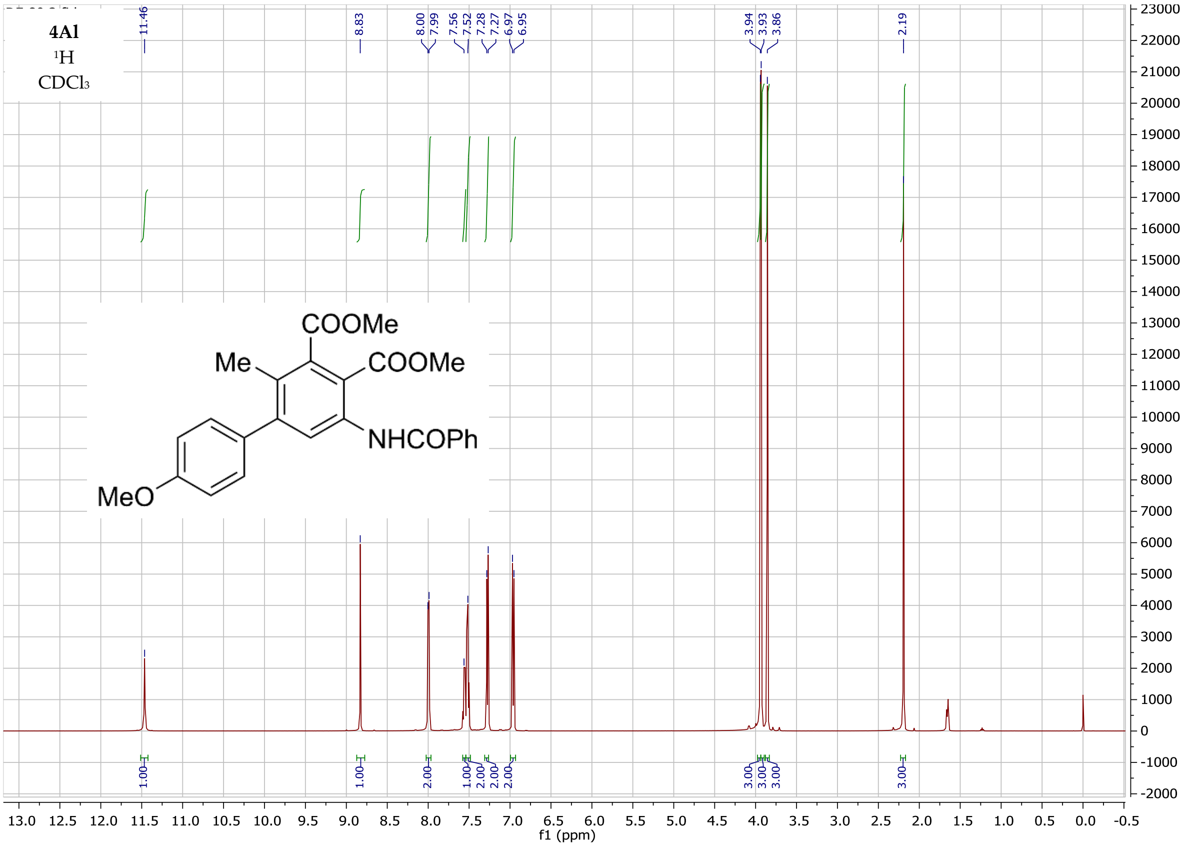
4Al
¹H
CDCl3
11.46
8.83
8.00
7.99
7.56
7.52
7.28
7.27
6.97
6.95
3.94
3.93
3.86
2.19
COOMe
Me
COOMe
NHCOPh
MeO
1.00
1.00
2.00
1.00
2.00
2.00
2.00
3.00
3.00
3.00
3.00
f1 (ppm)

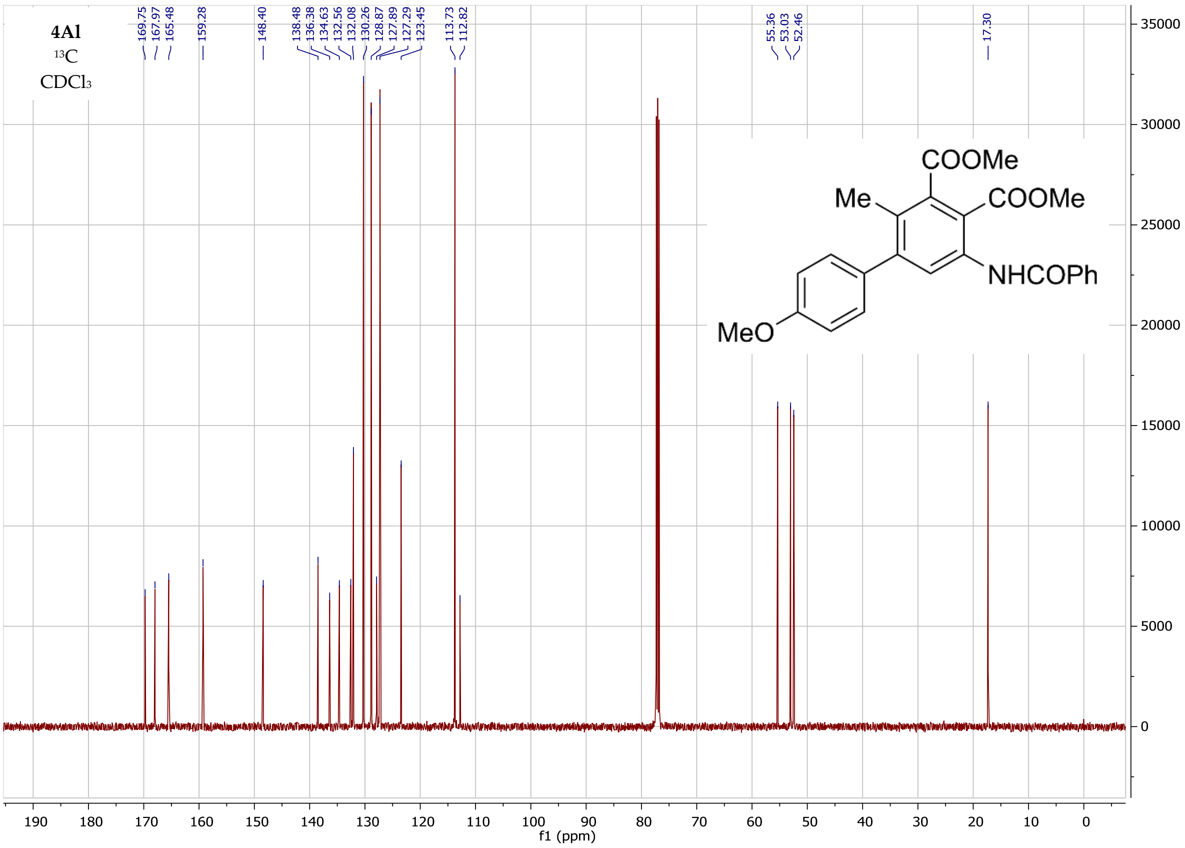
4Al
¹³C
CDCl3
169.75
167.97
165.48
159.28
148.40
138.48
136.38
134.63
132.56
132.08
130.26
128.87
127.89
127.29
123.45
113.73
112.82
55.36
53.03
52.46
17.30
COOMe
Me
COOMe
NHCOPh
MeO
f1 (ppm)

4Am

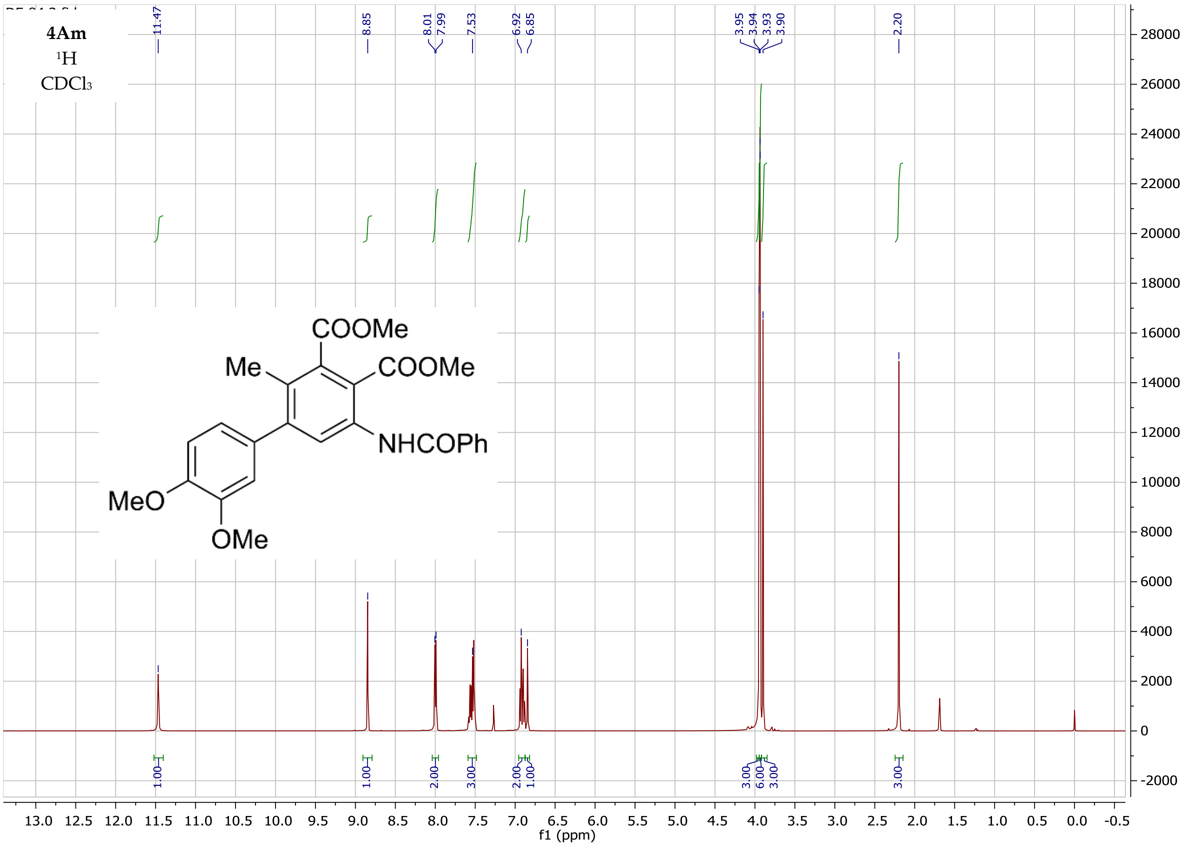
4Am
1H
CDCl3
11.47
8.85
8.01
7.99
7.53
6.92
6.85
3.95
3.94
3.93
3.90
2.20
COOMe
Me
COOMe
NHCOPh
MeO
OMe
1.00
1.00
2.00
3.00
2.00
1.00
3.00
6.00
3.00
3.00
f1 (ppm)

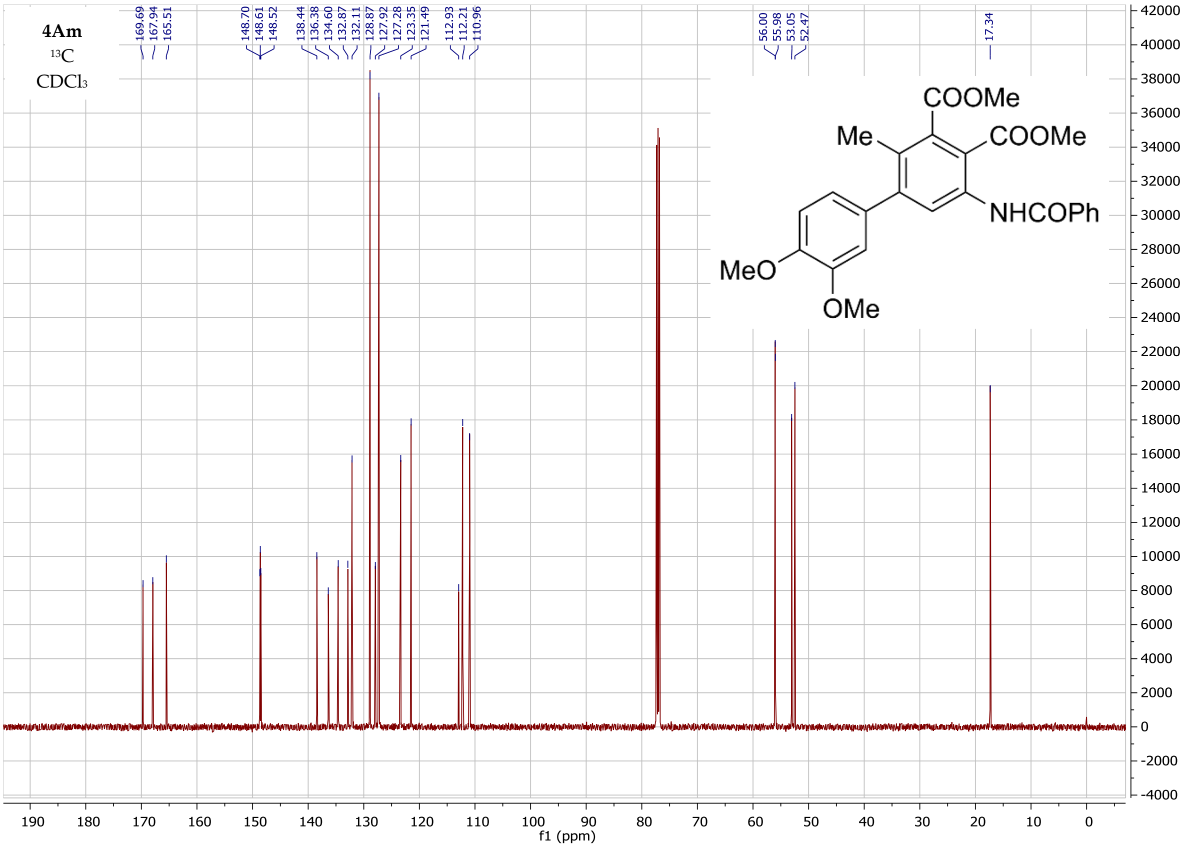
4Am
13C
CDCl3
169.69
167.94
165.51
148.70
148.61
148.52
138.44
136.38
134.60
132.87
132.11
128.87
127.92
127.28
123.35
121.49
112.93
112.21
110.96
56.00
55.98
53.05
52.47
17.34
COOMe
Me
COOMe
NHCOPh
MeO
OMe
f1 (ppm)

4An

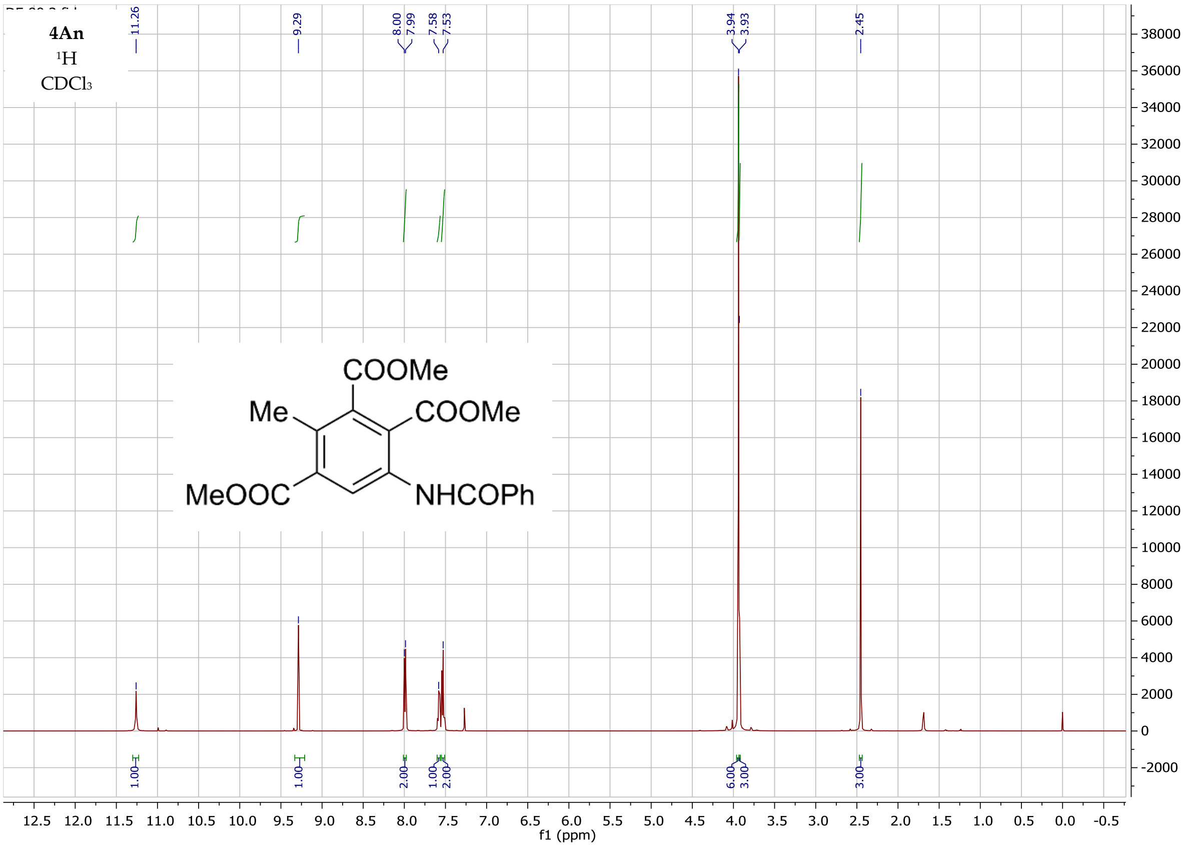
4An
1H
CDCl3
11.26
9.29
8.00
7.99
7.58
7.53
3.94
3.93
2.45
COOMe
Me
COOMe
MeOOC
NHCOPh
1.00
1.00
2.00
1.00
2.00
6.00
3.00
3.00
f1 (ppm)

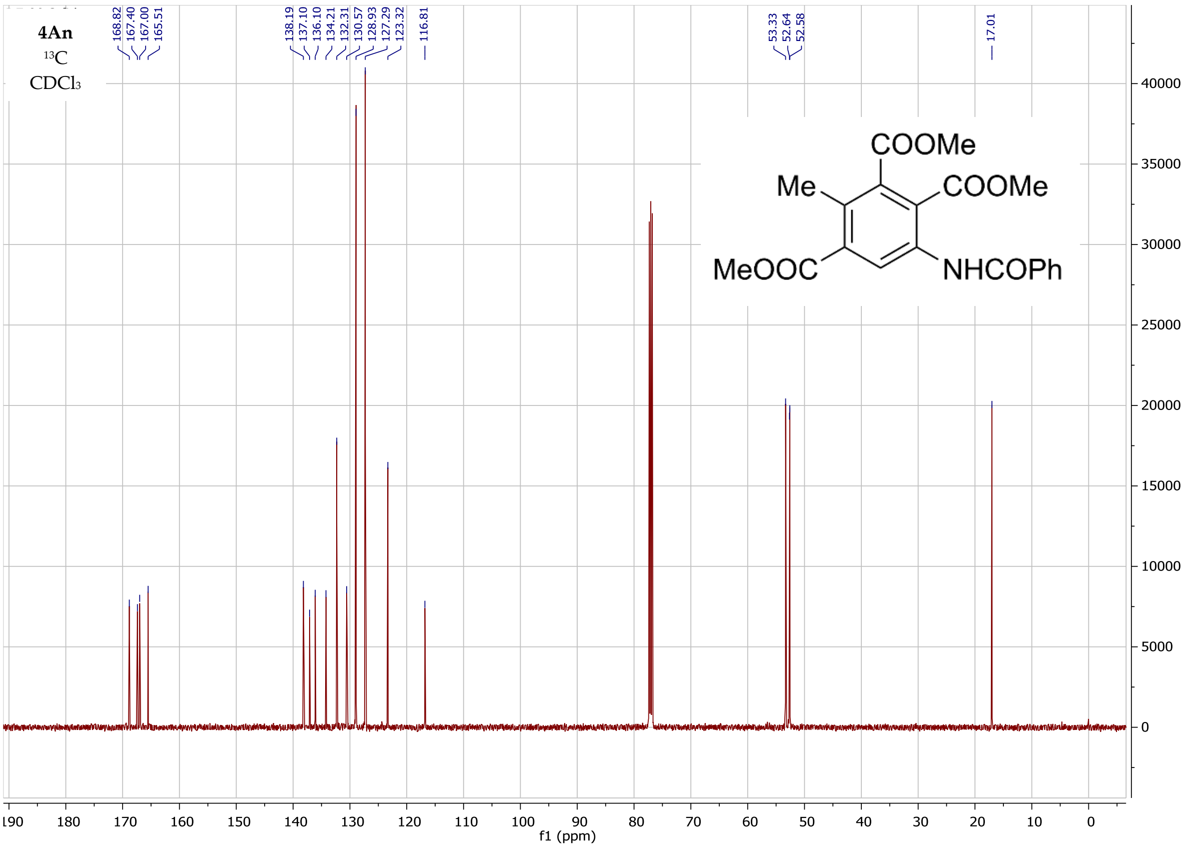
4An
13C
CDCl3
168.82
167.40
167.00
165.51
138.19
137.10
136.10
134.21
132.31
130.57
128.93
127.29
123.32
116.81
53.33
52.64
52.58
17.01
COOMe
Me
COOMe
MeOOC
NHCOPh
f1 (ppm)

4Ao

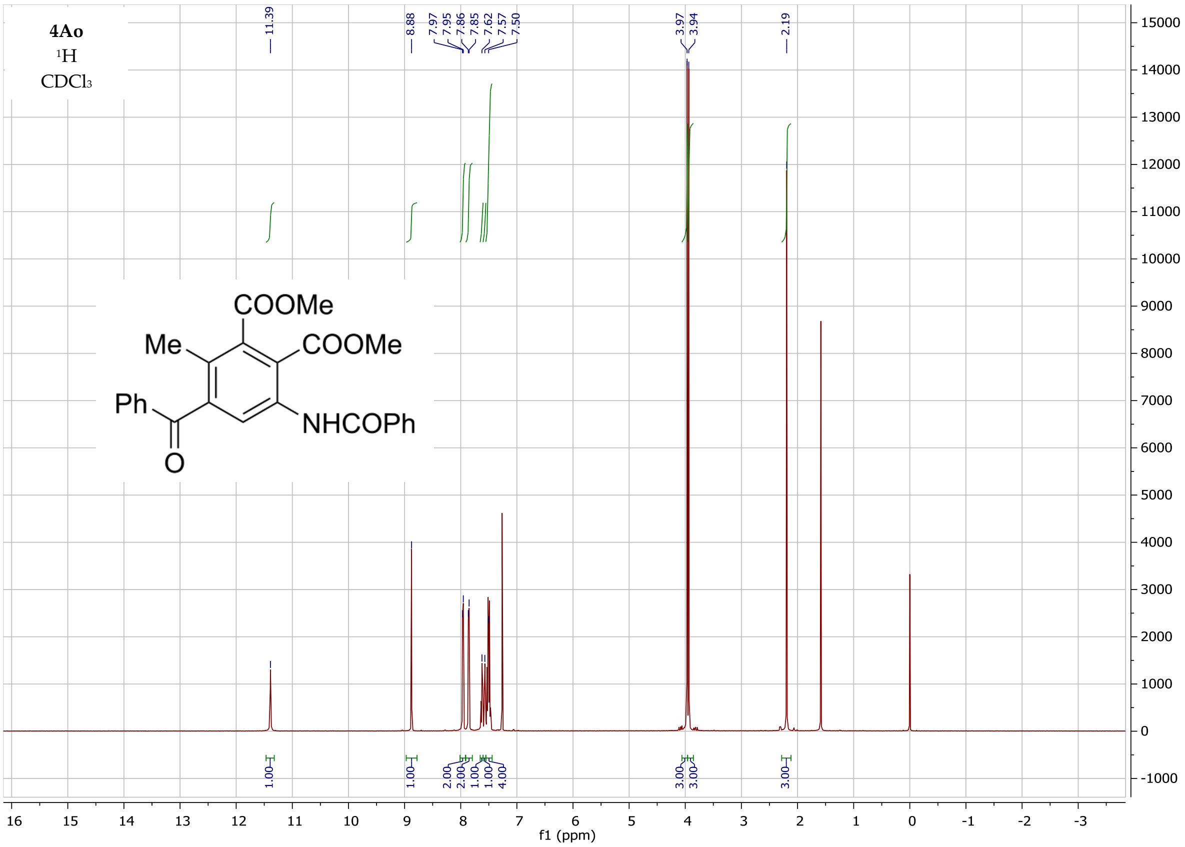

4Ao
1H
CDCl3
11.39
8.88
7.97
7.95
7.86
7.85
7.62
7.57
7.50
3.97
3.94
2.19
COOMe
Me
COOMe
Ph
NHCOPh
O
1.00
1.00
2.00
2.00
1.00
1.00
4.00
3.00
3.00
3.00
f1 (ppm)

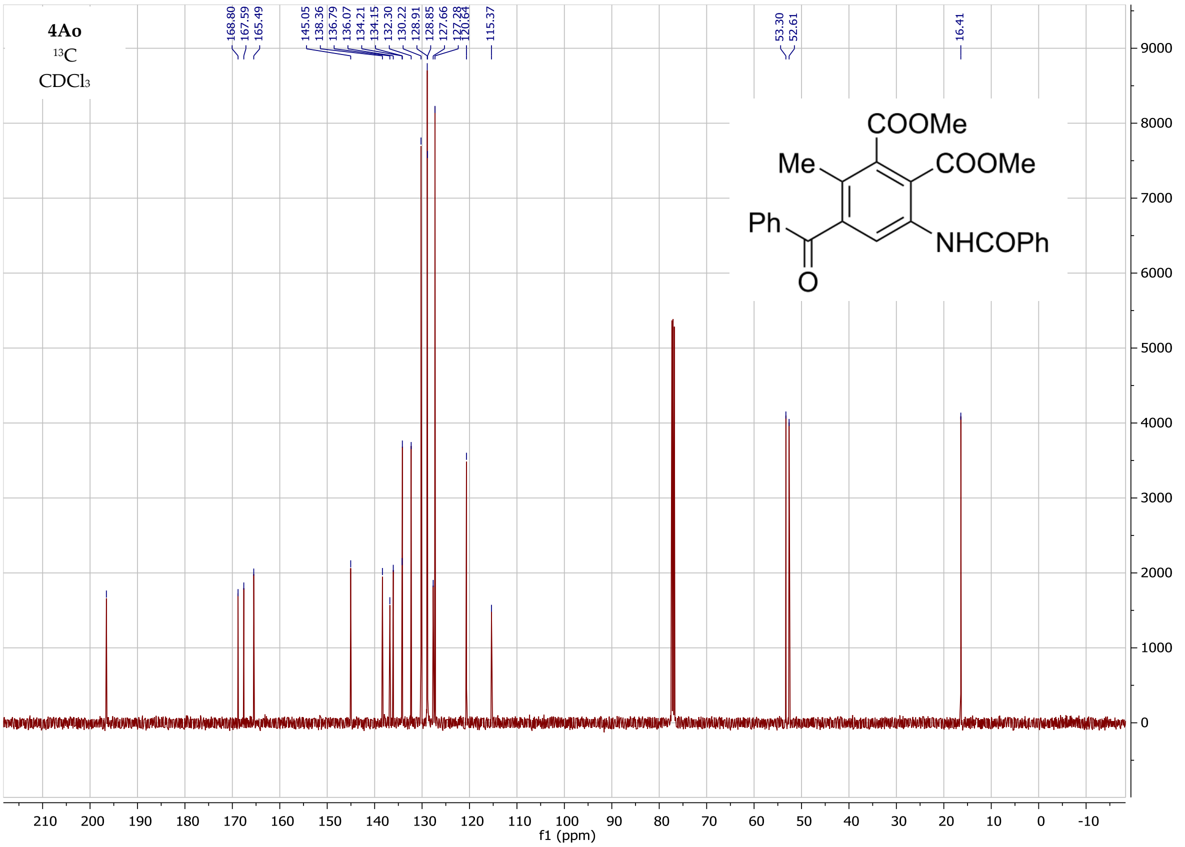

4Ao
13C
CDCl3
168.80
167.59
165.49
145.05
138.36
136.79
136.07
134.21
134.15
132.30
130.22
128.91
128.85
127.66
115.37
53.30
52.61
16.41
COOMe
Me
COOMe
Ph
NHCOPh
O
f1 (ppm)

4Ap

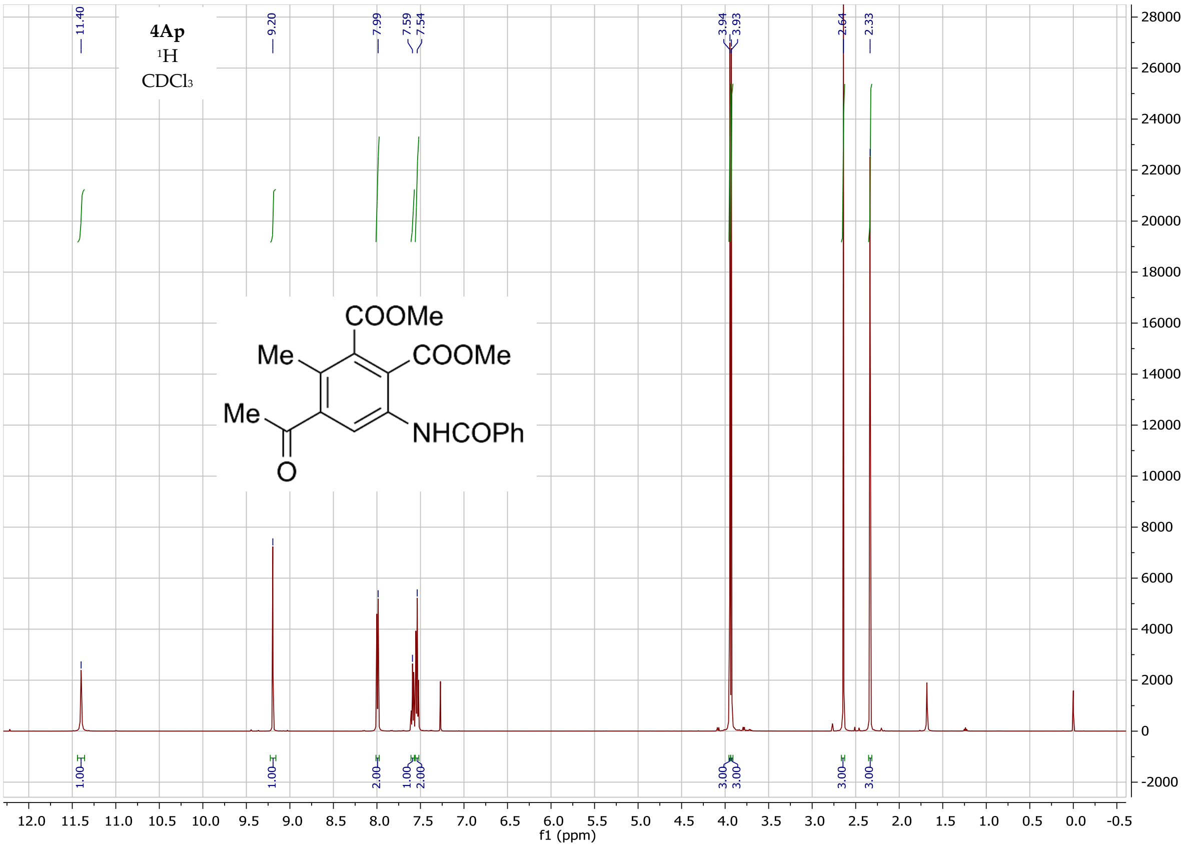
4Ap
1H
CDCl3
COOMe
Me
COOMe
Me
NHCOPh
O
11.40
9.20
7.99
7.59
7.54
3.94
3.93
2.64
2.33
1.00
1.00
2.00
1.00
2.00
3.00
3.00
3.00
3.00
f1 (ppm)

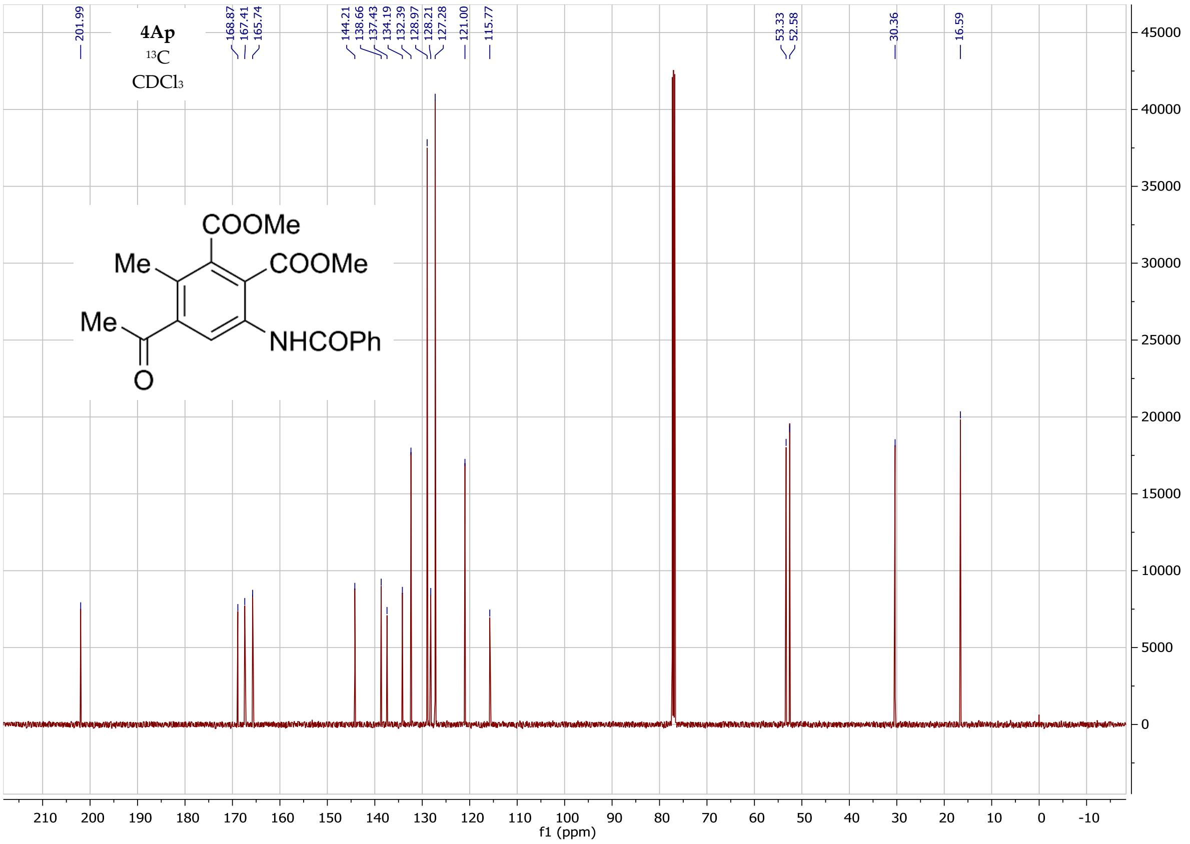
4Ap
13C
CDCl3
COOMe
Me
COOMe
Me
NHCOPh
O
201.99
168.87
167.41
165.74
144.21
138.66
137.43
134.19
132.39
128.97
128.21
127.28
121.00
115.77
53.33
52.58
30.36
16.59
f1 (ppm)

4Aq

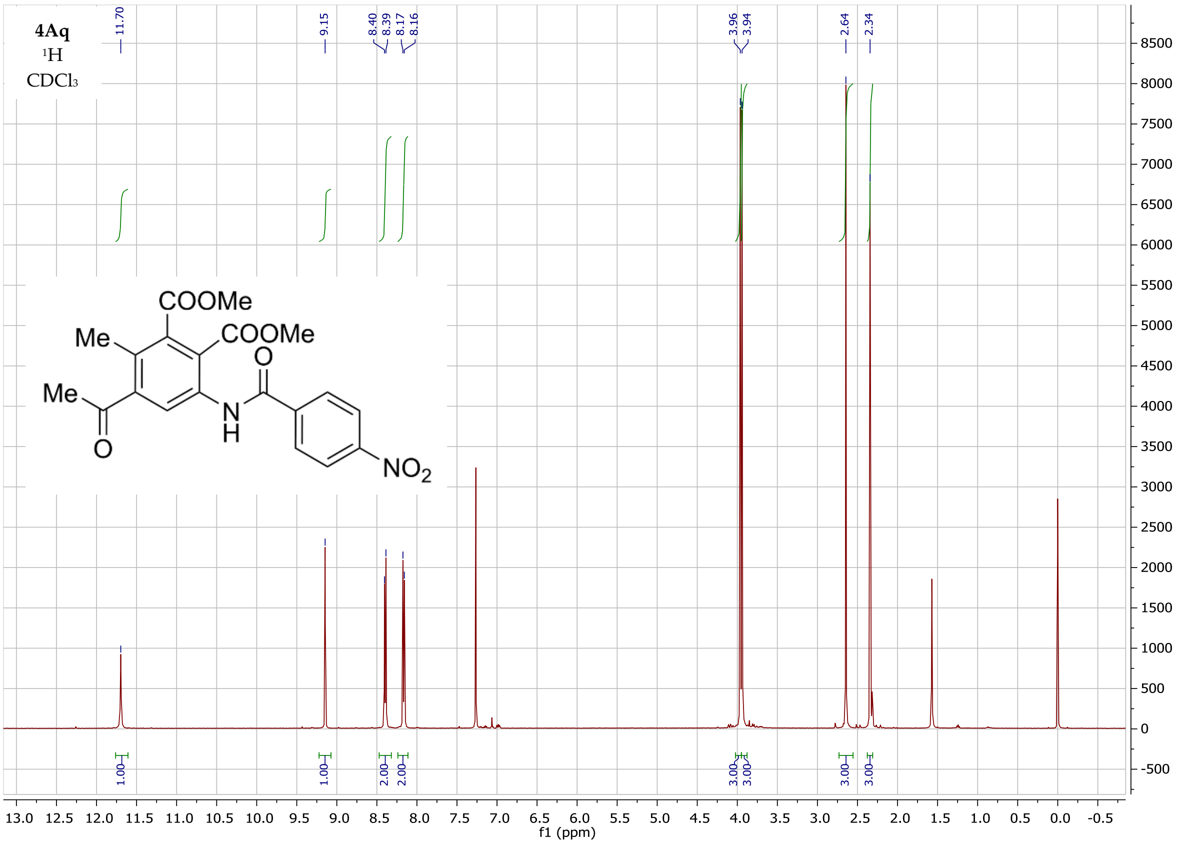
4Aq
1H
CDCl3
11.70
9.15
8.40
8.39
8.17
8.16
3.96
3.94
2.64
2.34
COOMe
Me
COOMe
O
Me
N
H
O
NO2
1.00
1.00
2.00
2.00
3.00
3.00
3.00
3.00
f1 (ppm)

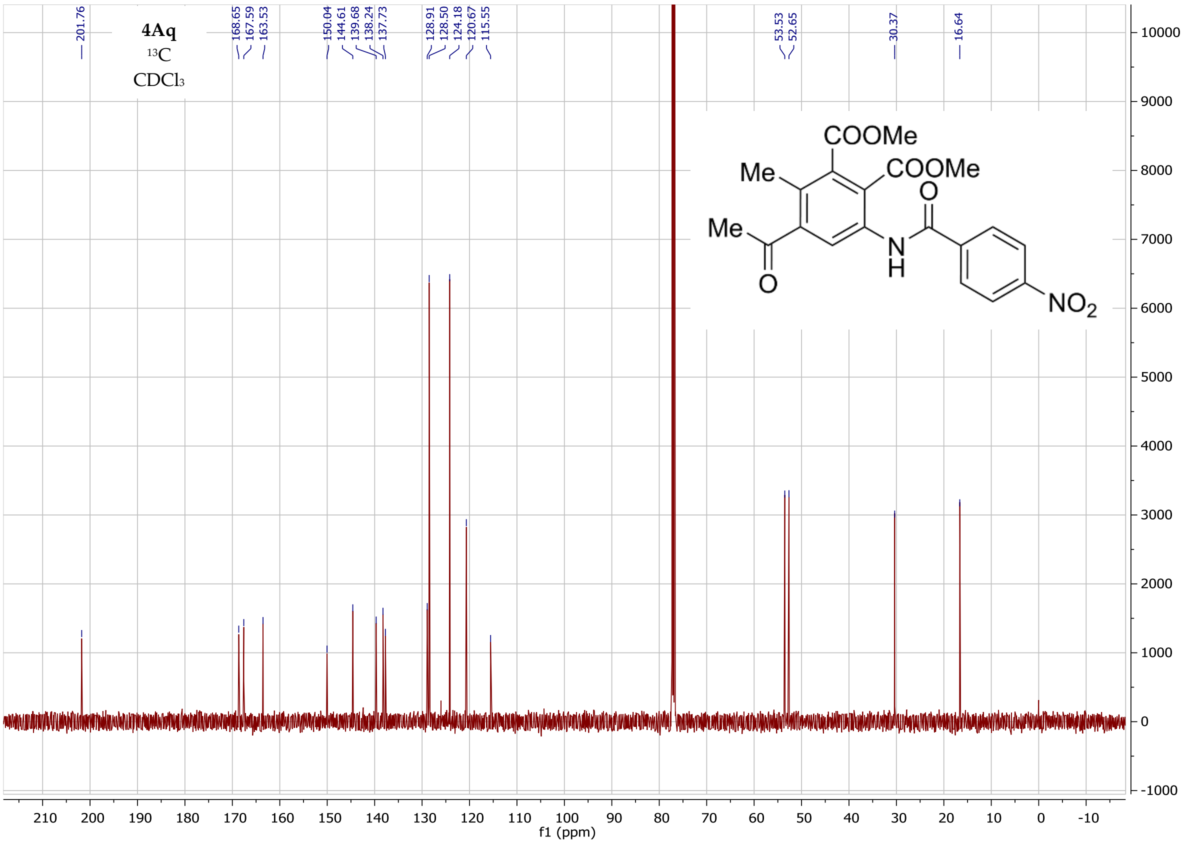
201.76
4Aq
13C
CDCl3
168.65
167.59
163.53
150.04
144.61
139.68
138.24
137.73
128.91
128.50
124.18
120.67
115.55
53.53
52.65
30.37
16.64
COOMe
Me
COOMe
O
Me
N
H
O
NO2
f1 (ppm)

4Ar

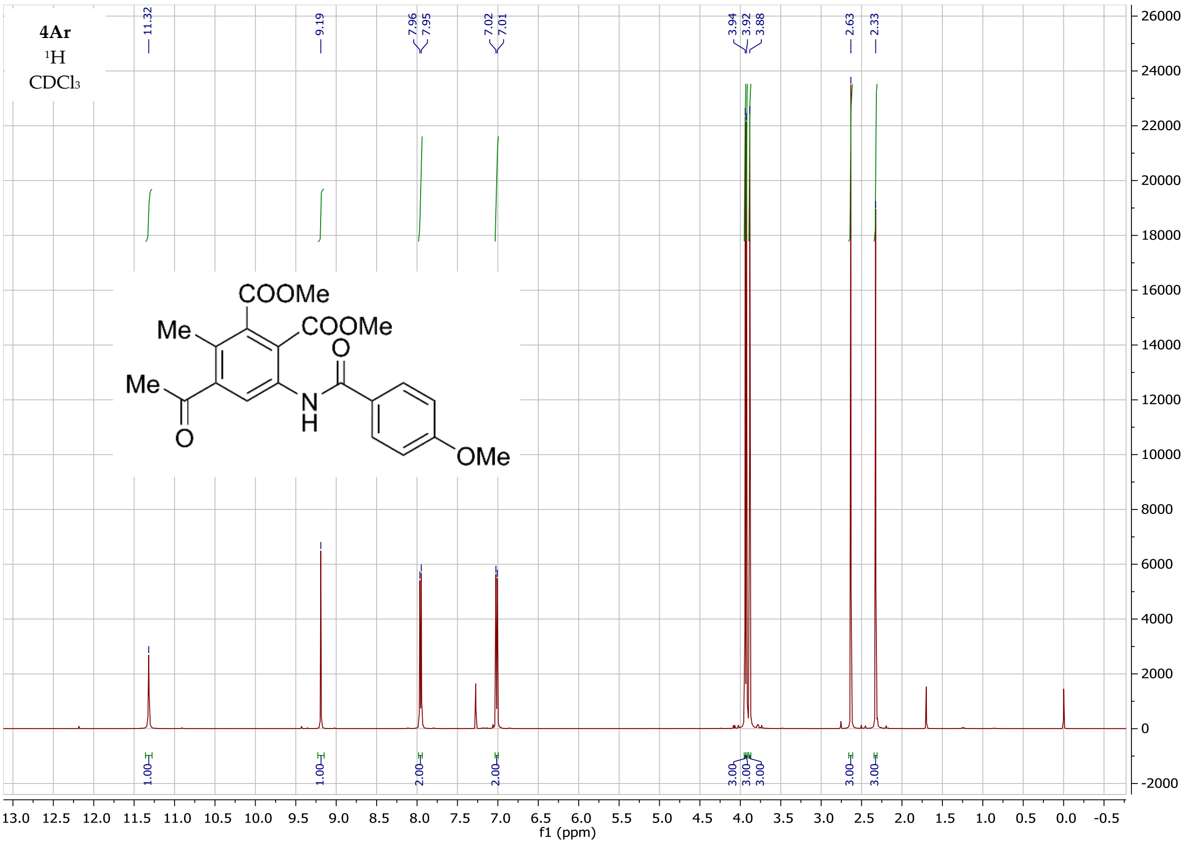
4Ar
1H
CDCl3
11.32
9.19
7.96
7.95
7.02
7.01
3.94
3.92
3.88
2.63
2.33
COOMe
Me
COOMe
Me
N
H
O
O
OMe
1.00
1.00
2.00
2.00
3.00
3.00
3.00
3.00
3.00
f1 (ppm)

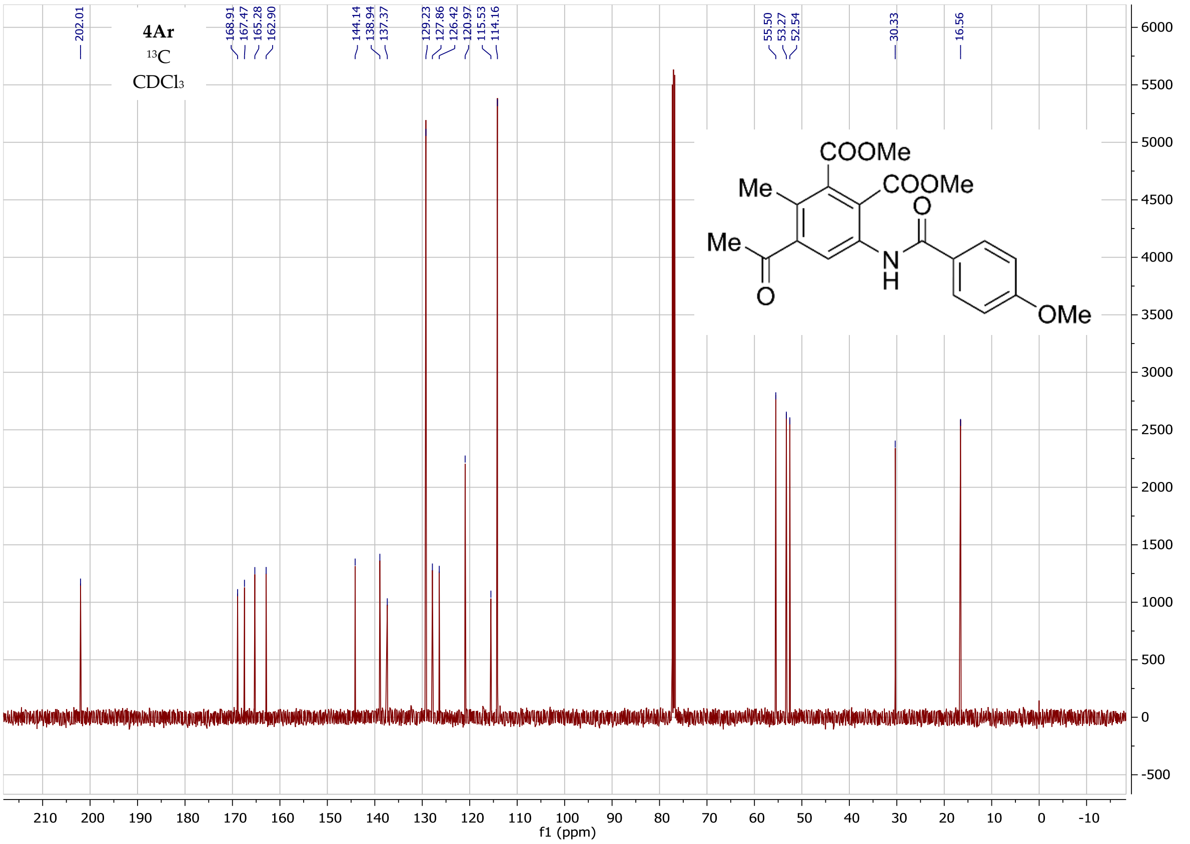
4Ar
13C
CDCl3
202.01
168.91
167.47
165.28
162.90
144.14
138.94
137.37
129.23
127.86
126.42
120.97
115.53
114.16
55.50
53.27
52.54
30.33
16.56
COOMe
Me
COOMe
Me
N
H
O
O
OMe
f1 (ppm)

4As

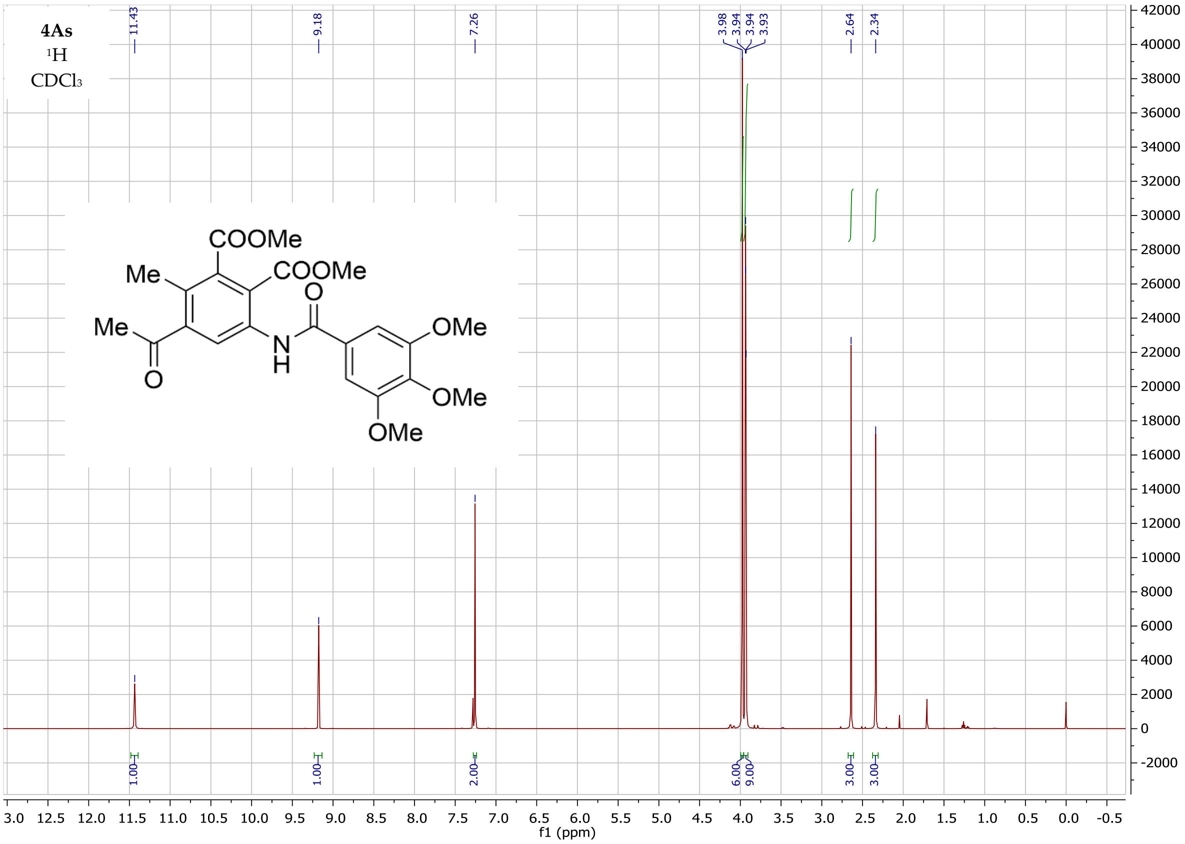
4As
1H
CDCl3
COOMe
COOMe
Me
Me
O
N
H
O
OMe
OMe
OMe
f1 (ppm)

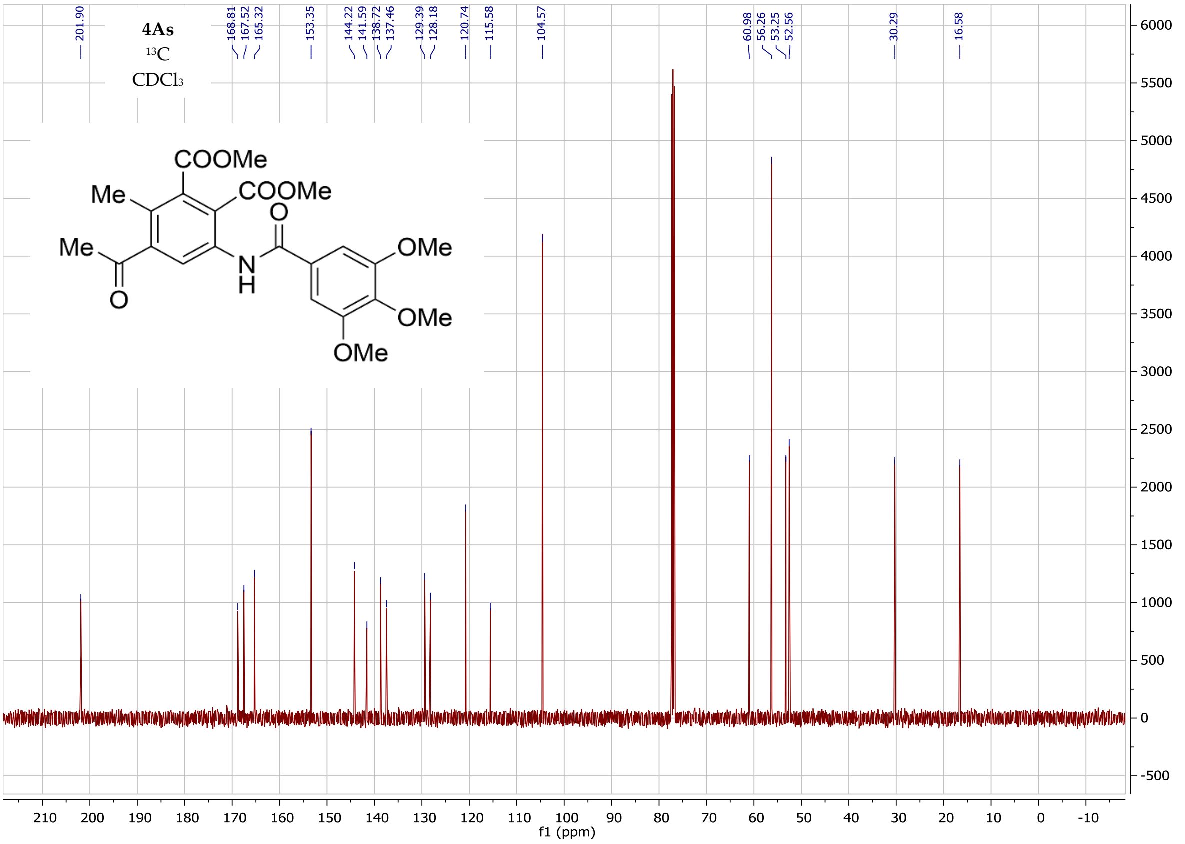
4As
13C
CDCl3
COOMe
COOMe
Me
Me
O
N
H
O
OMe
OMe
OMe
f1 (ppm)

4At

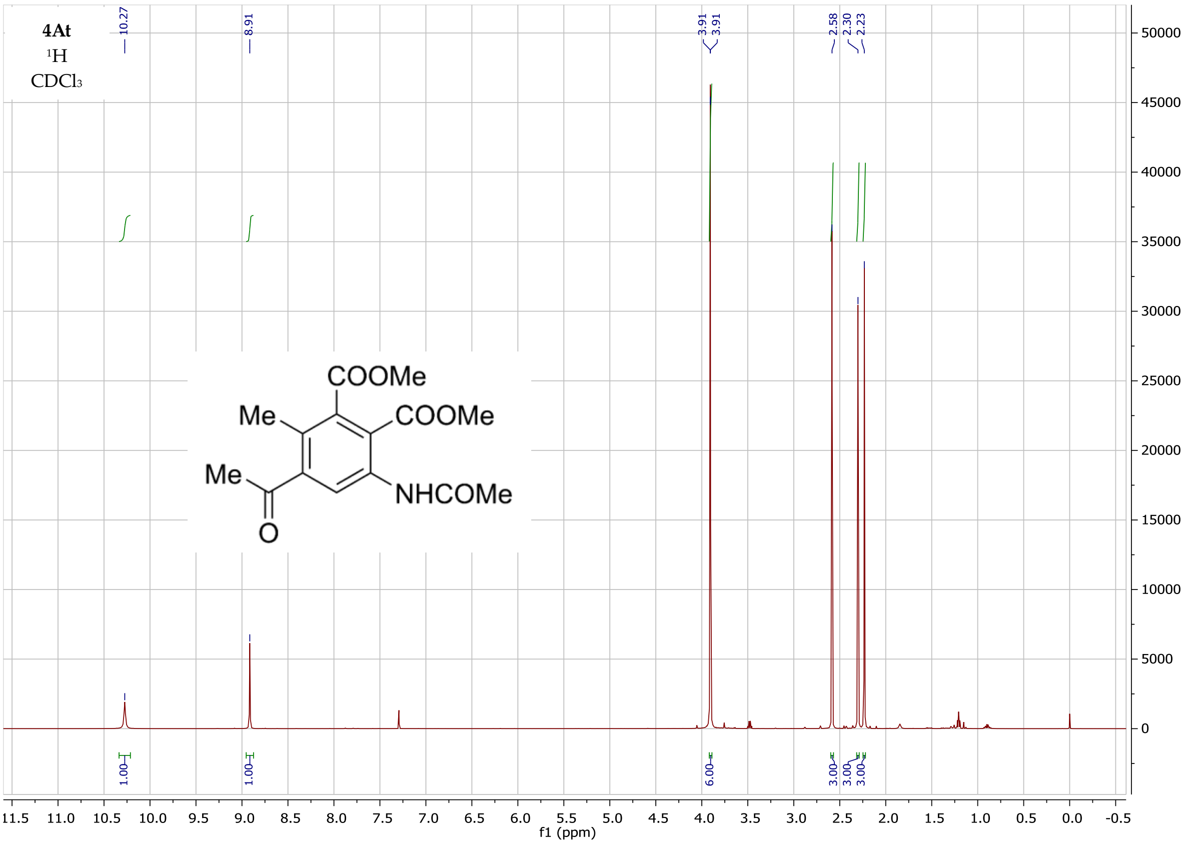
4At
1H
CDCl3
COOMe
Me
COOMe
Me
NHCOMe
O
10.27
8.91
3.91
3.91
2.58
2.30
2.23
1.00
1.00
6.00
3.00
3.00
3.00
f1 (ppm)

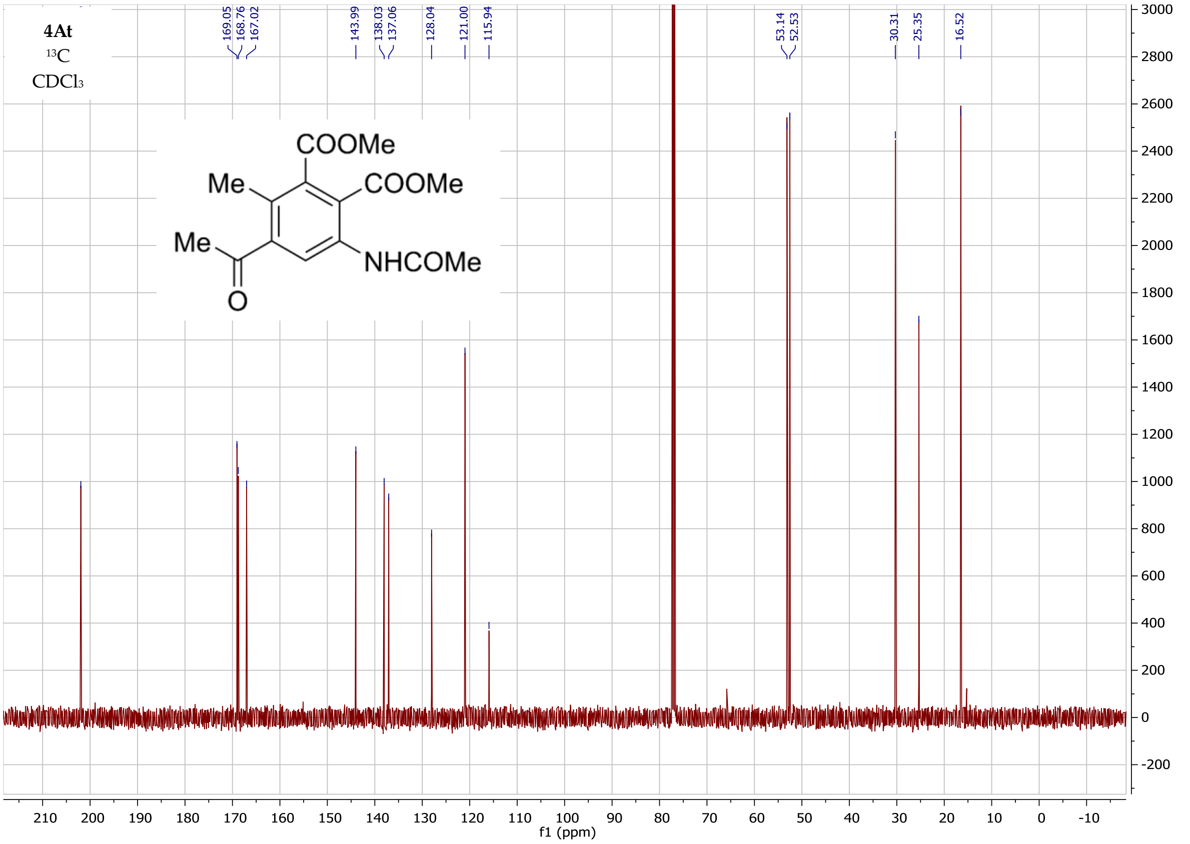
4At
13C
CDCl3
COOMe
Me
COOMe
Me
NHCOMe
O
169.05
168.76
167.02
143.99
138.03
137.06
128.04
121.00
115.94
53.14
52.53
30.31
25.35
16.52
f1 (ppm)

4Au

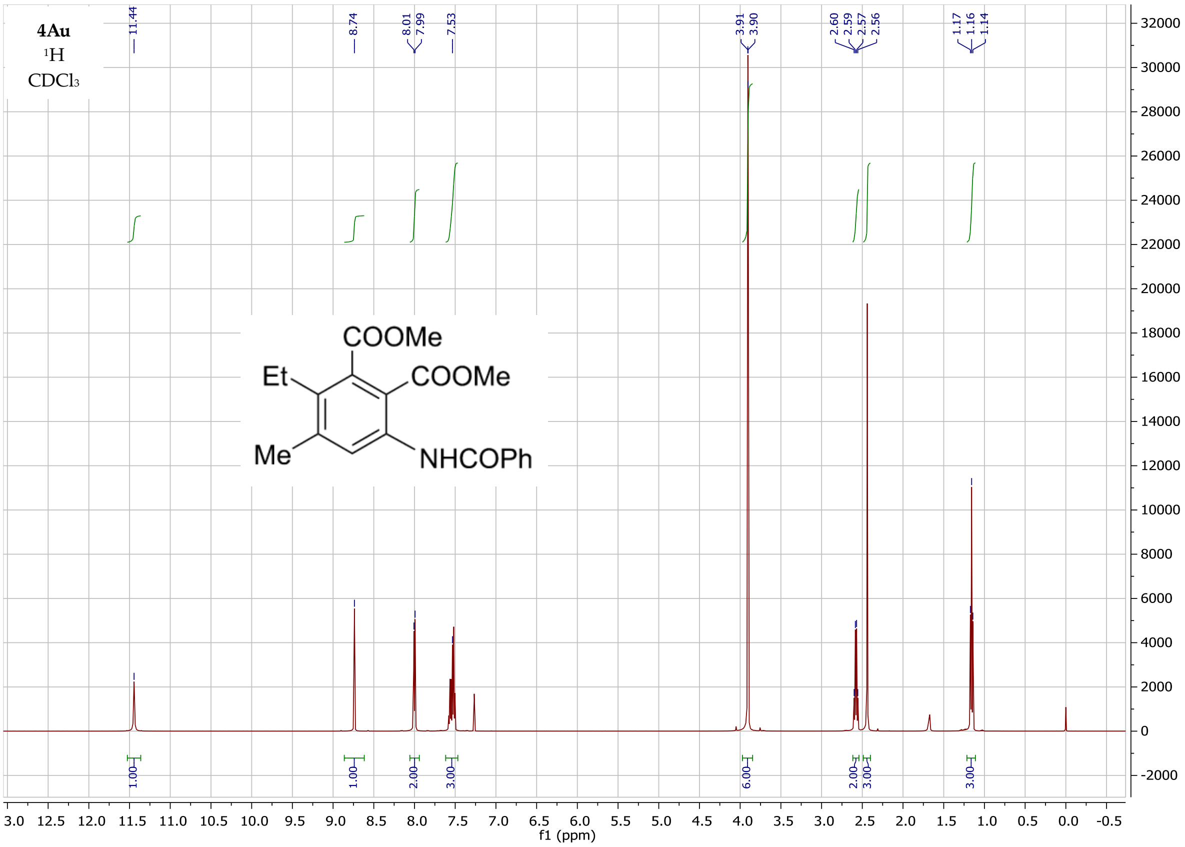

4Au
¹H
CDCl₃
11.44
8.74
8.01
7.99
7.53
3.91
3.90
2.60
2.59
2.57
2.56
1.17
1.16
1.14
COOMe
Et
COOMe
Me
NHCOPh
1.00
1.00
2.00
3.00
6.00
2.00
3.00
3.00
f1 (ppm)

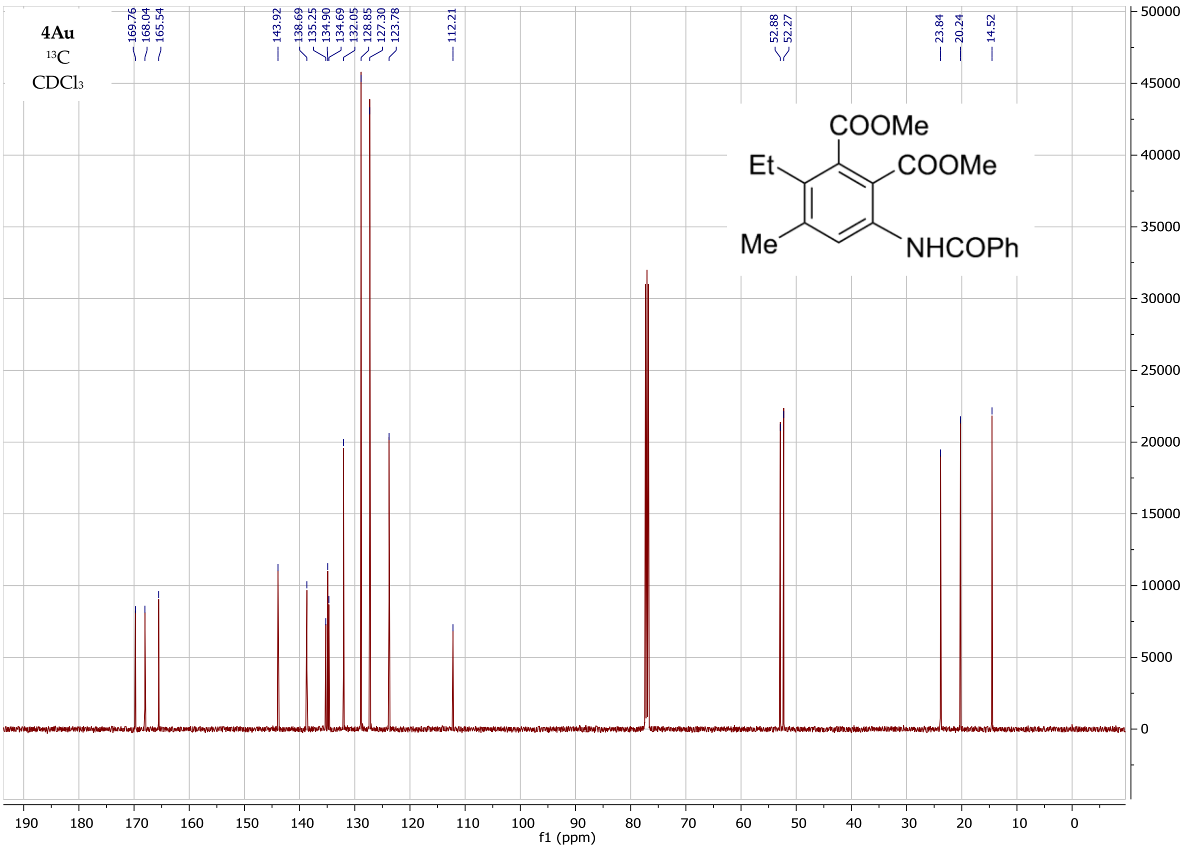

4Au
¹³C
CDCl₃
169.76
168.04
165.54
143.92
138.69
135.25
134.90
134.69
132.05
128.85
127.30
123.78
112.21
52.88
52.27
23.84
20.24
14.52
COOMe
Et
COOMe
Me
NHCOPh
f1 (ppm)

4Av

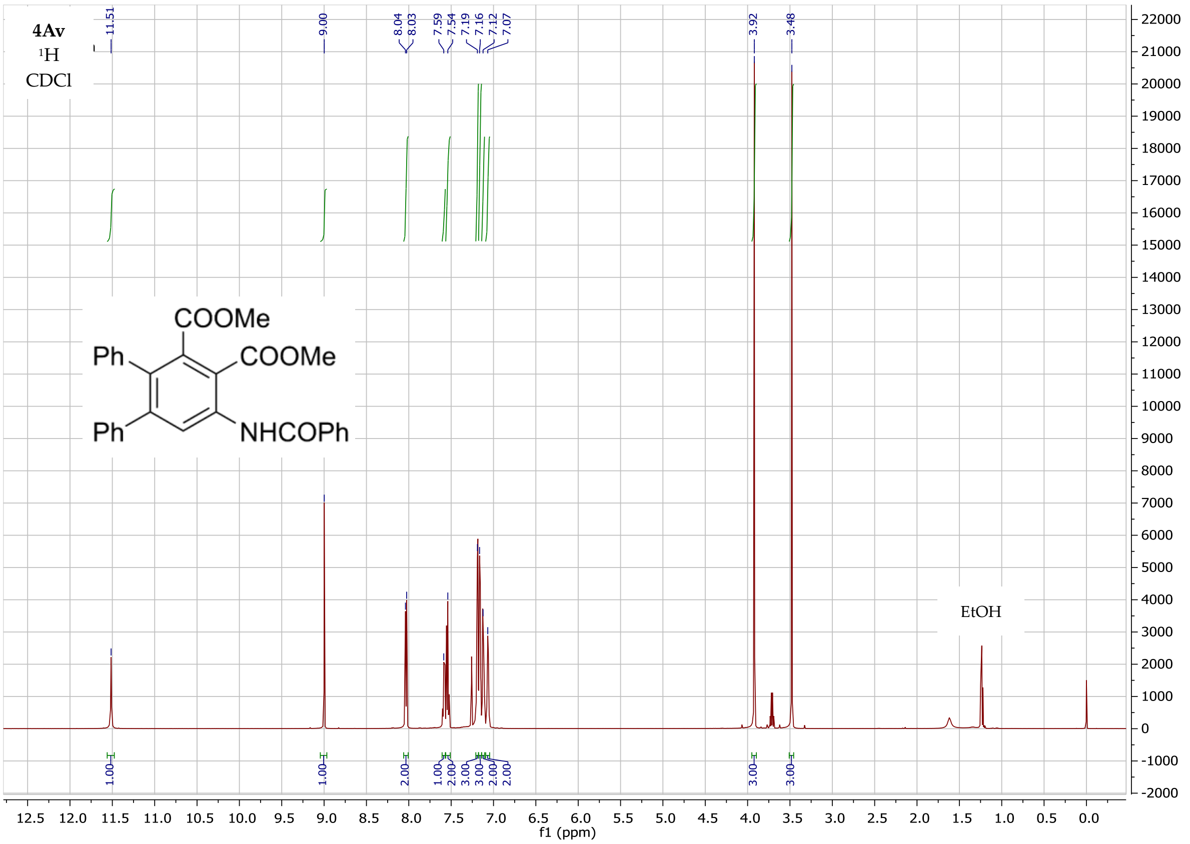
4Av
1H
CDCl
COOMe
Ph
COOMe
Ph
NHCOPh
EtOH
f1 (ppm)

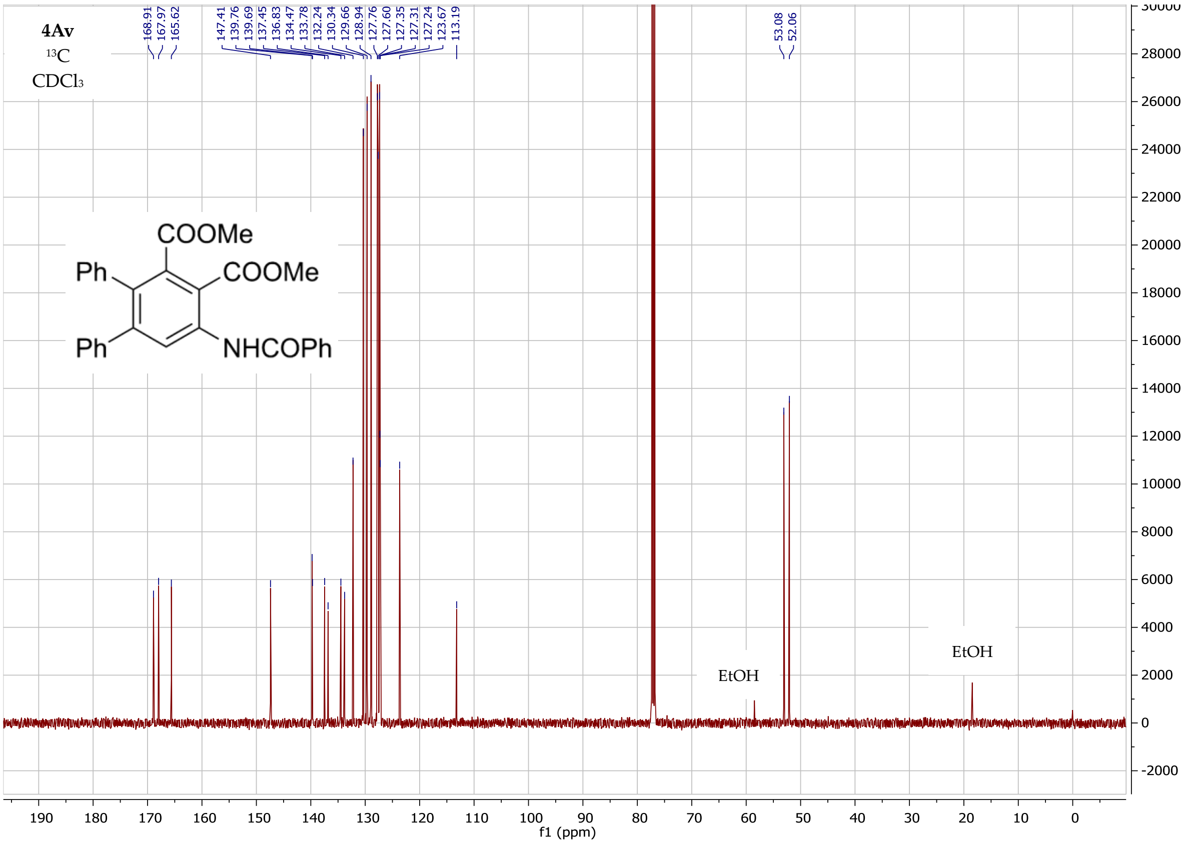
4Av
13C
CDCl3
COOMe
Ph
COOMe
Ph
NHCOPh
EtOH
EtOH
f1 (ppm)

4Aw

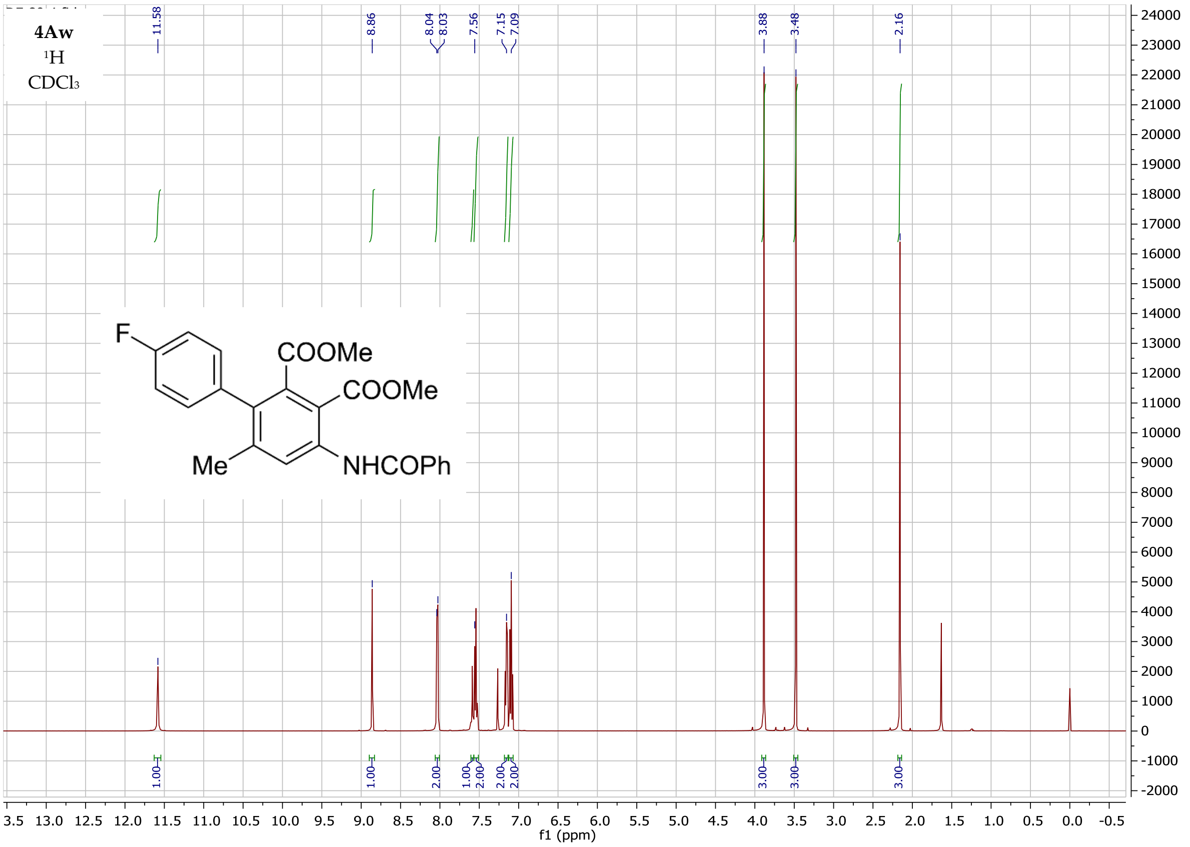
4Aw
1H
CDCl3
11.58
8.86
8.04
8.03
7.56
7.15
7.09
3.88
3.48
2.16
F
COOMe
COOMe
Me
NHCOPh
1.00
1.00
2.00
1.00
2.00
2.00
2.00
3.00
3.00
3.00
f1 (ppm)

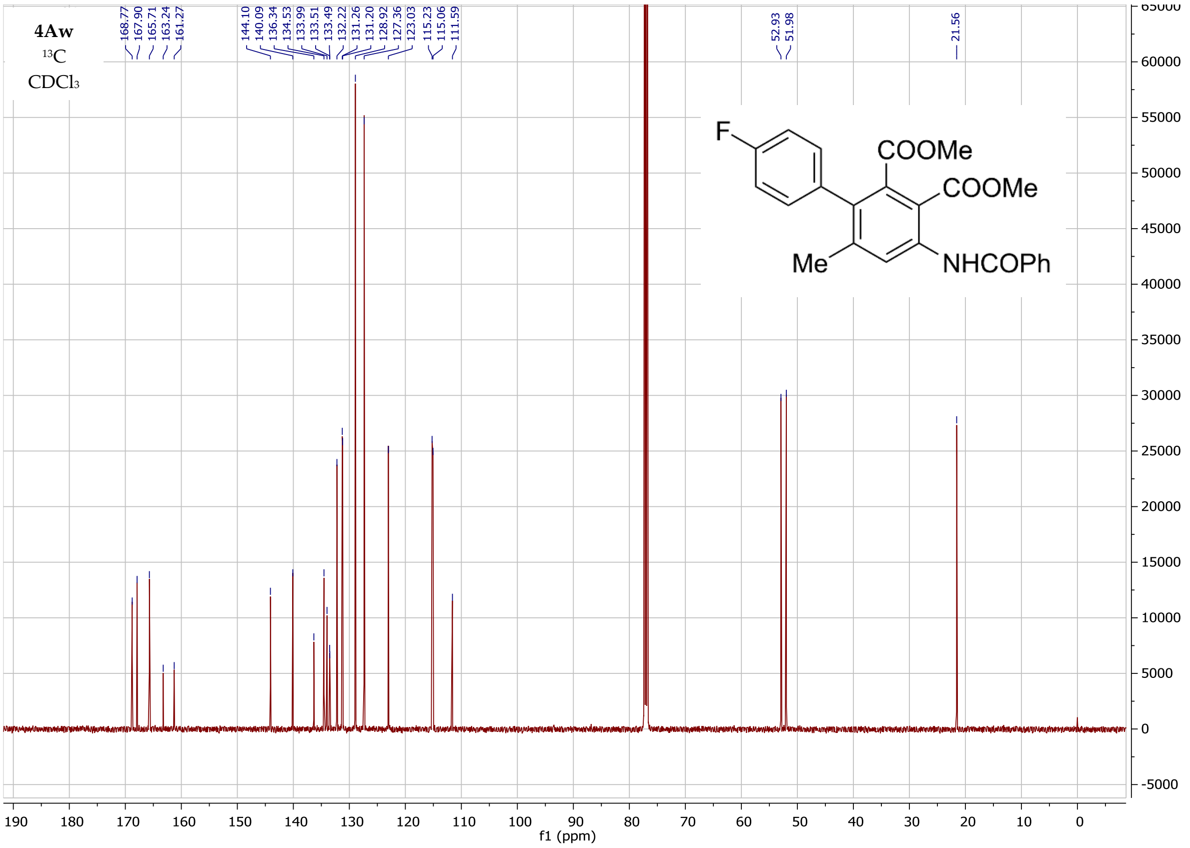
4Aw
13C
CDCl3
168.77
167.90
165.71
163.24
161.27
144.10
140.09
136.34
134.53
133.99
133.51
133.49
132.22
131.26
131.20
128.92
127.36
123.03
115.23
115.06
111.59
52.93
51.98
21.56
F
COOMe
COOMe
Me
NHCOPh
f1 (ppm)

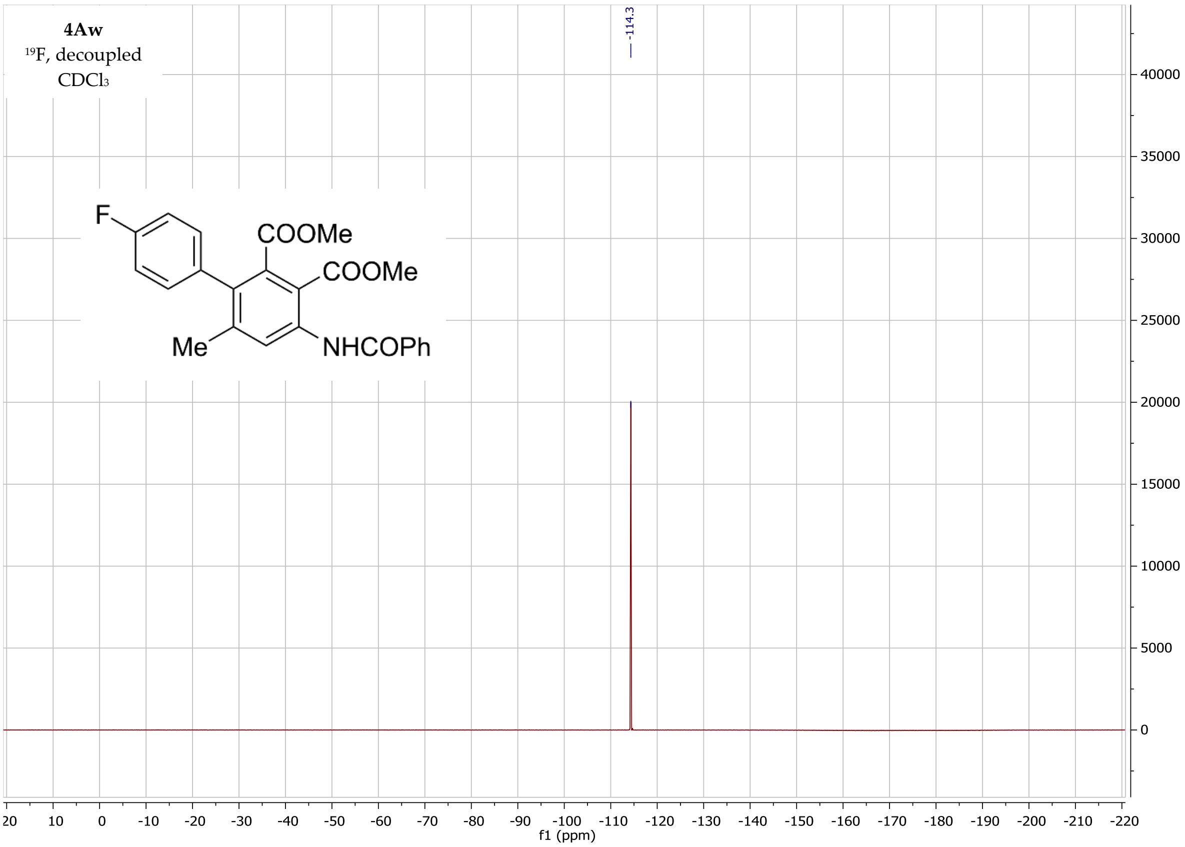
4Aw
19F, decoupled
CDCl3
F
COOMe
COOMe
Me
NHCOPh
-114.3
f1 (ppm)

4Ax

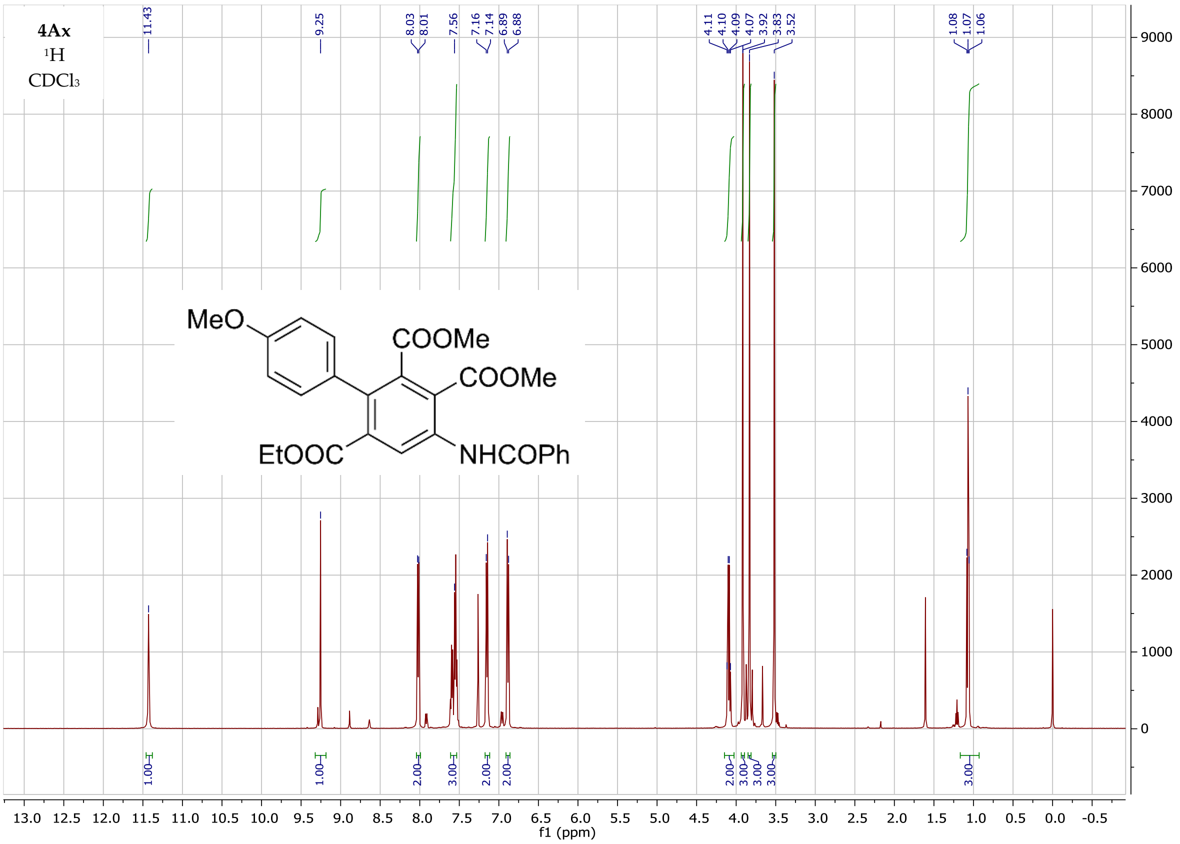

4Ax
^{1}H
CDCl3
MeO
COOMe
COOMe
EtOOC
NHCOPh
11.43
9.25
8.03
8.01
7.56
7.16
7.14
6.89
6.88
4.11
4.10
4.09
4.07
3.92
3.83
3.52
1.08
1.07
1.06
1.00
1.00
2.00
3.00
2.00
2.00
2.00
3.00
3.00
3.00
3.00
f1 (ppm)

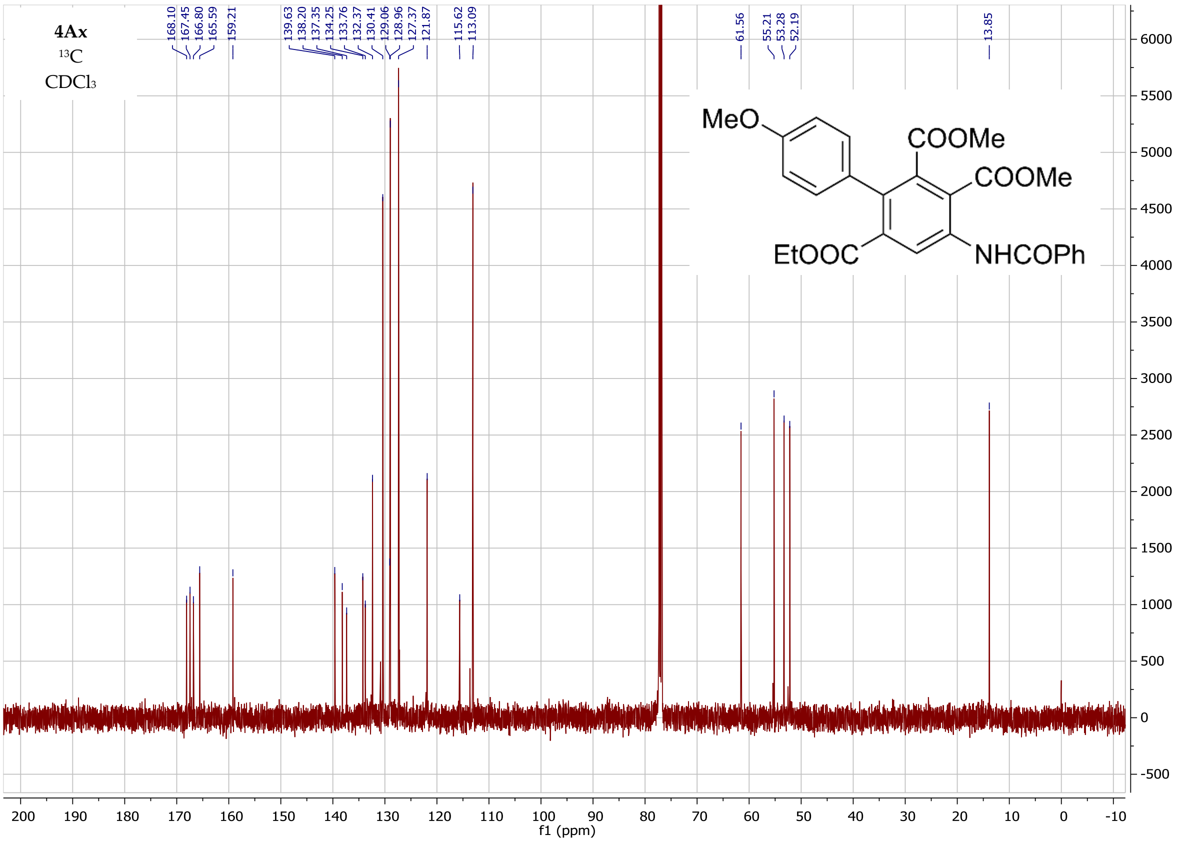

4Ax
^{13}C
CDCl3
MeO
COOMe
COOMe
EtOOC
NHCOPh
168.10
167.45
166.80
165.59
159.21
139.63
138.20
137.35
134.25
133.76
132.37
130.41
129.06
128.96
127.37
121.87
115.62
113.09
61.56
55.21
53.28
52.19
13.85
f1 (ppm)

4Ay

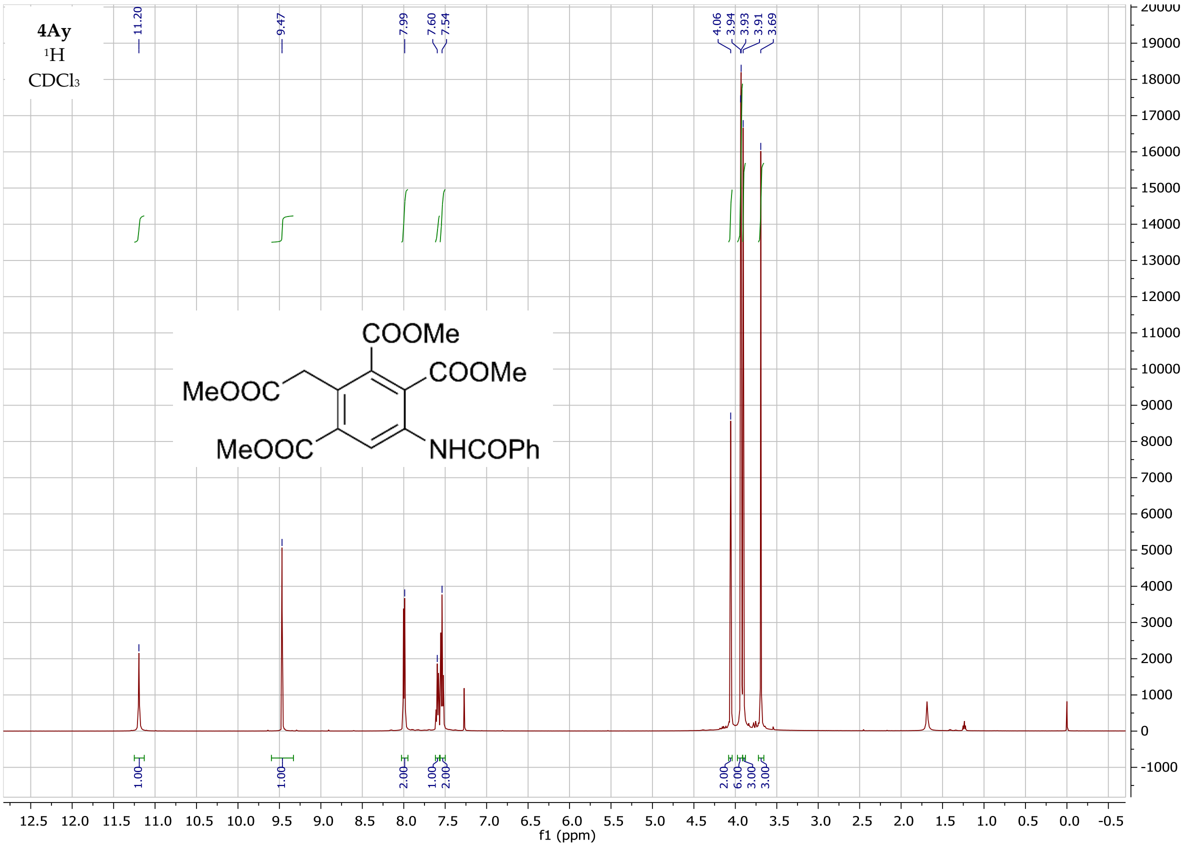
4Ay
1H
CDCl3
11.20
9.47
7.99
7.60
7.54
4.06
3.94
3.93
3.91
3.69
COOMe
MeOOC
COOMe
MeOOC
NHCOPh
1.00
1.00
2.00
1.00
2.00
2.00
6.00
3.00
3.00
f1 (ppm)

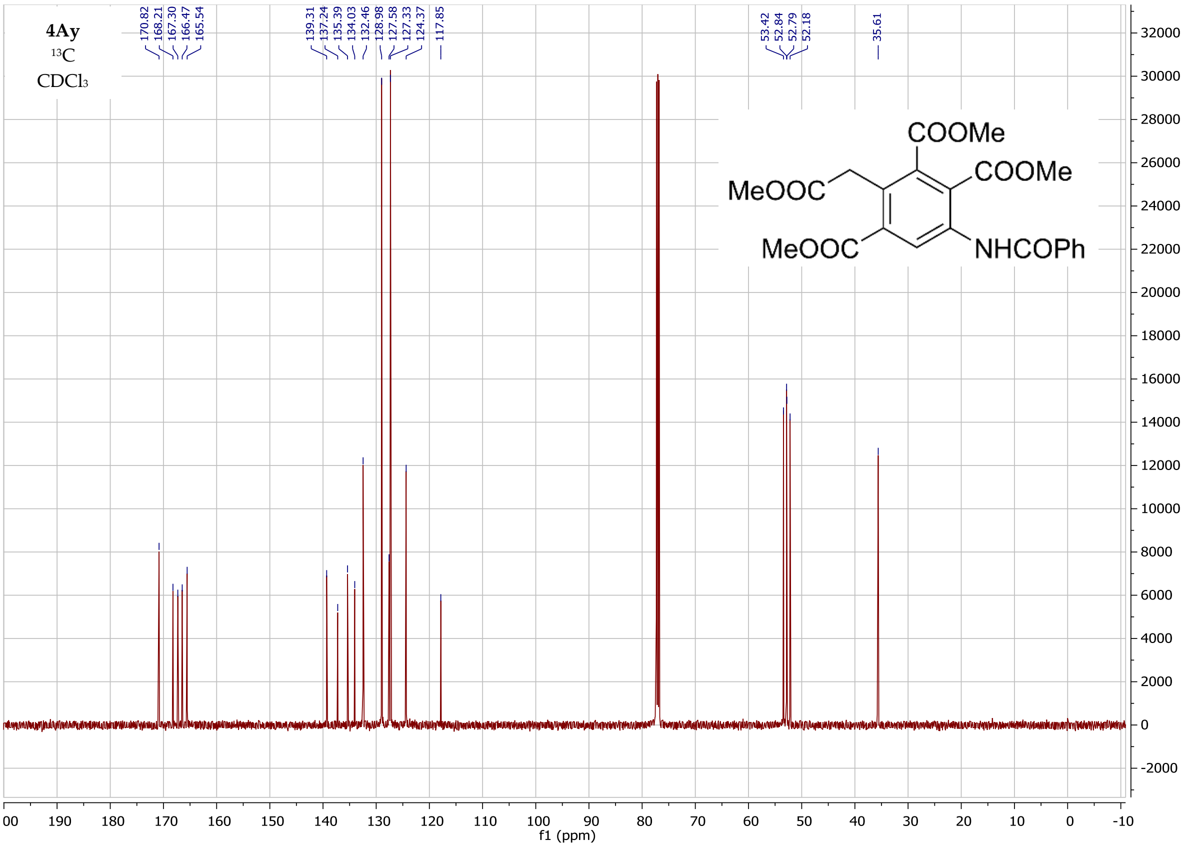
4Ay
13C
CDCl3
170.82
168.21
167.30
166.47
165.54
139.31
137.24
135.39
134.03
132.46
128.98
127.58
127.33
124.37
117.85
53.42
52.84
52.79
52.18
35.61
COOMe
MeOOC
COOMe
MeOOC
NHCOPh
f1 (ppm)

4Az

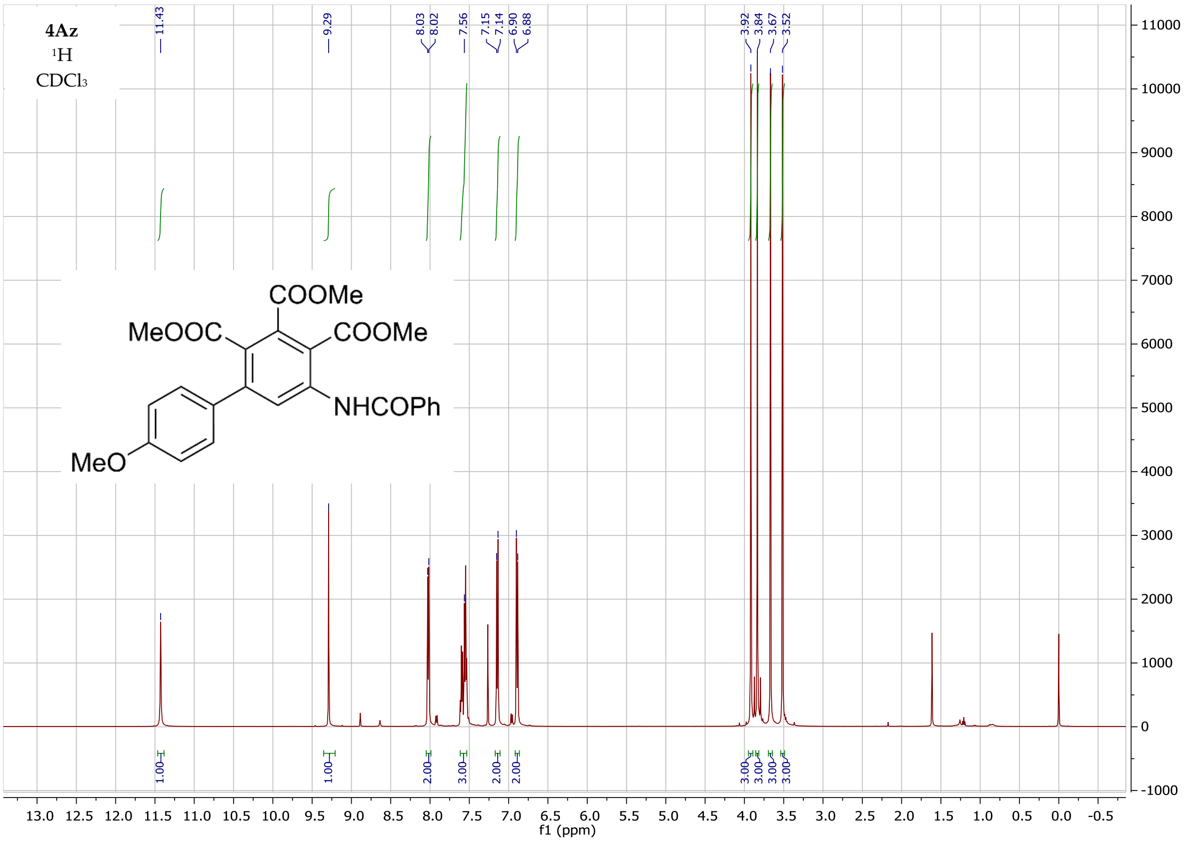
4Az
1H
CDCl3
11.43
9.29
8.03
8.02
7.56
7.15
7.14
6.90
6.88
3.92
3.84
3.67
3.52
COOMe
MeOOC
COOMe
NHCOPh
MeO
1.00
1.00
2.00
3.00
2.00
2.00
3.00
3.00
3.00
3.00
f1 (ppm)

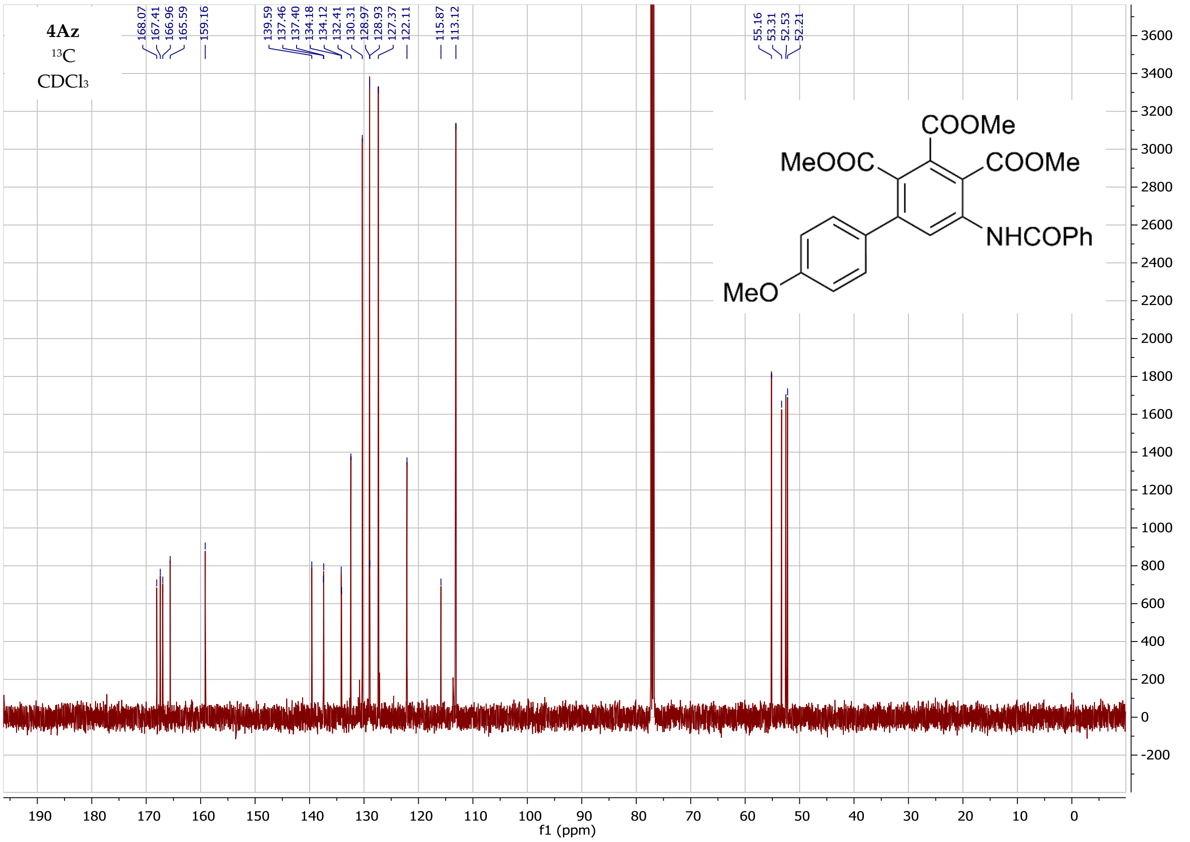
4Az
13C
CDCl3
168.07
167.41
166.96
165.59
159.16
139.59
137.46
137.40
134.18
134.12
132.41
130.31
128.97
128.93
127.37
122.11
115.87
113.12
55.16
53.31
52.53
52.21
COOMe
MeOOC
COOMe
NHCOPh
MeO
f1 (ppm)

4Aaa

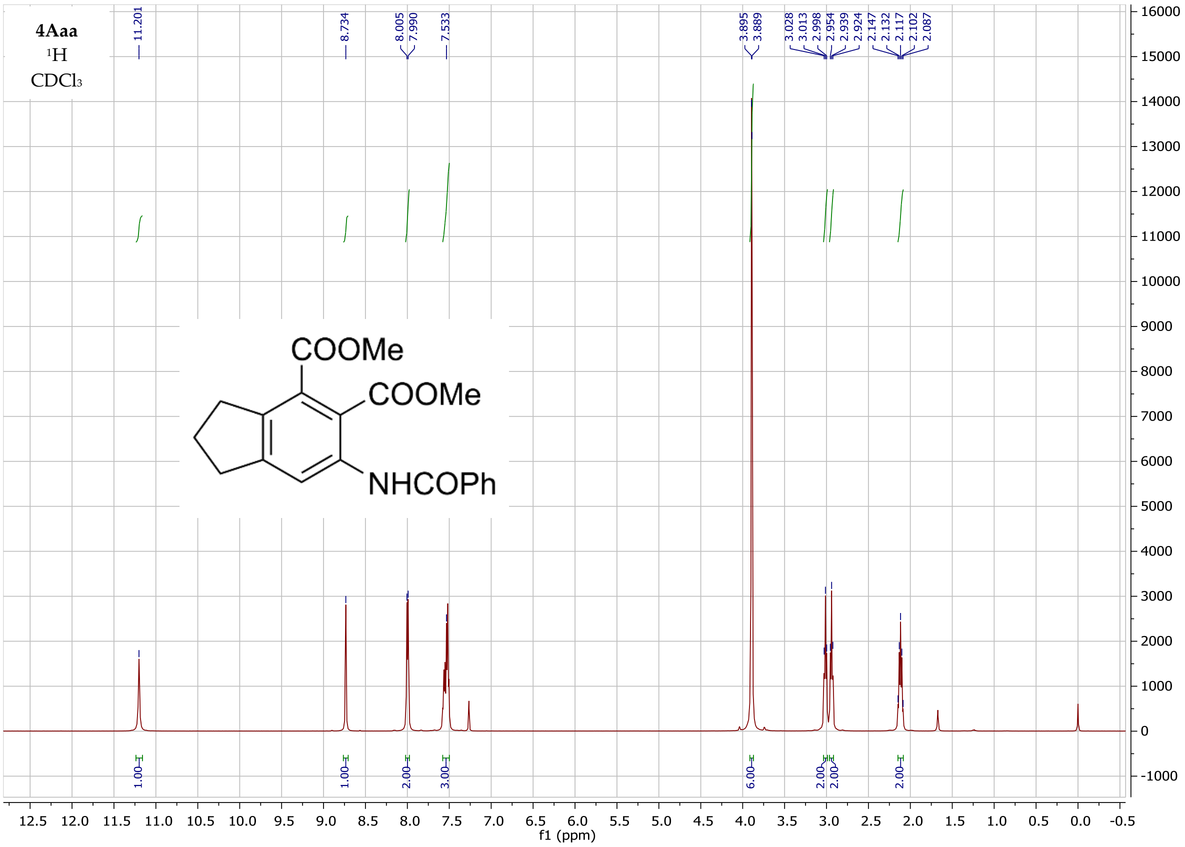
4Aaa
1H
CDCl3
11.201
8.734
8.005
7.990
7.533
3.895
3.889
3.028
3.013
2.998
2.954
2.939
2.924
2.147
2.132
2.117
2.102
2.087
1.00
1.00
2.00
3.00
6.00
2.00
2.00
2.00
COOMe
COOMe
NHCOPh
f1 (ppm)

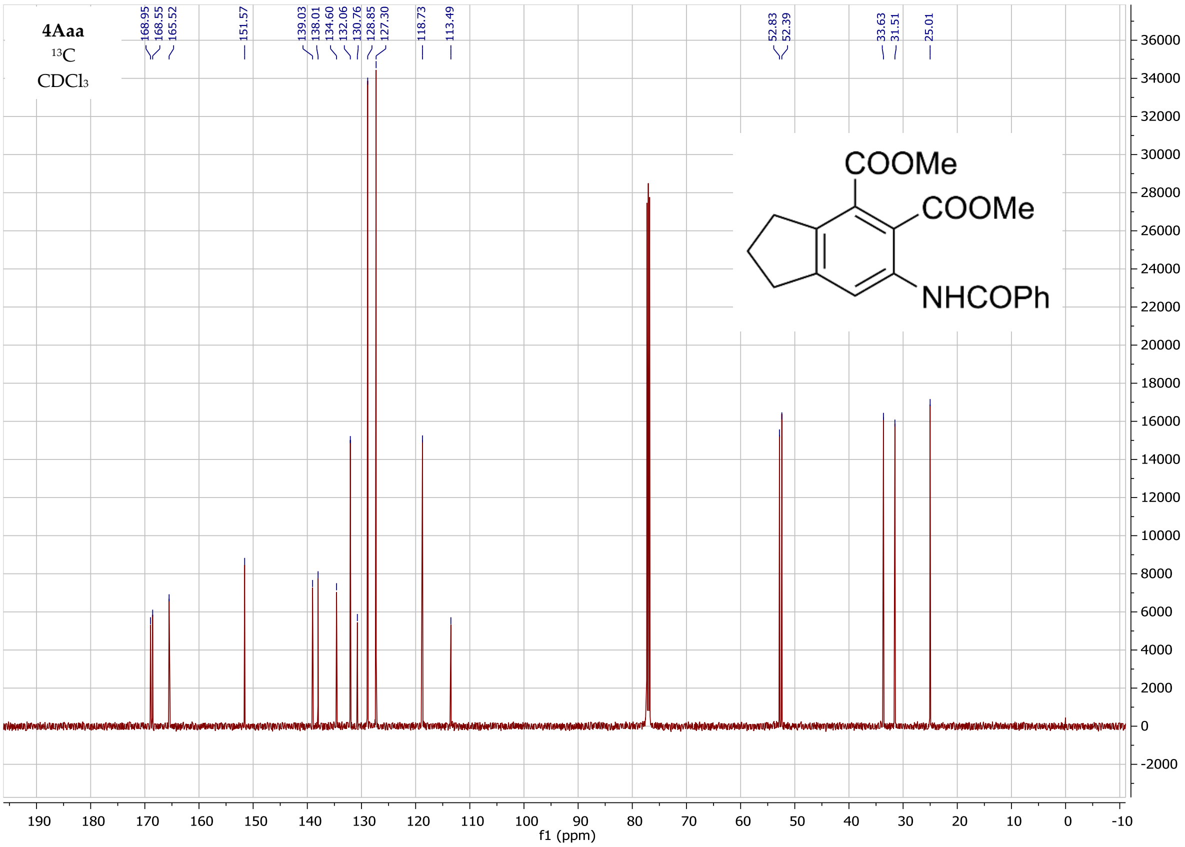
4Aaa
13C
CDCl3
168.95
168.55
165.52
151.57
139.03
138.01
134.60
132.06
130.76
128.85
127.30
118.73
113.49
52.83
52.39
33.63
31.51
25.01
COOMe
COOMe
NHCOPh
f1 (ppm)

4Aab

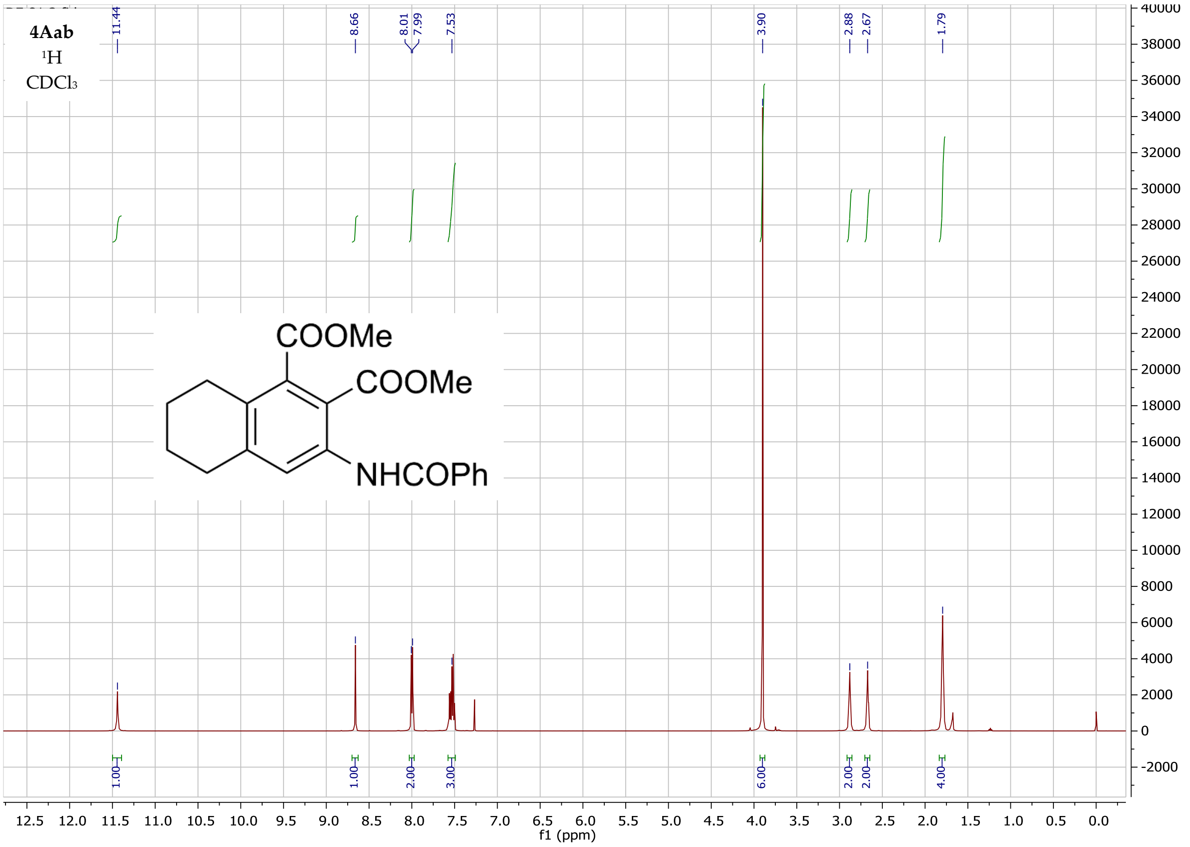

4Aab
^1H
$CDCl_3$
11.44
8.66
8.01
7.99
7.53
3.90
2.88
2.67
1.79
1.00
1.00
2.00
3.00
6.00
2.00
2.00
4.00
COOMe
COOMe
NHCOPh
f1 (ppm)

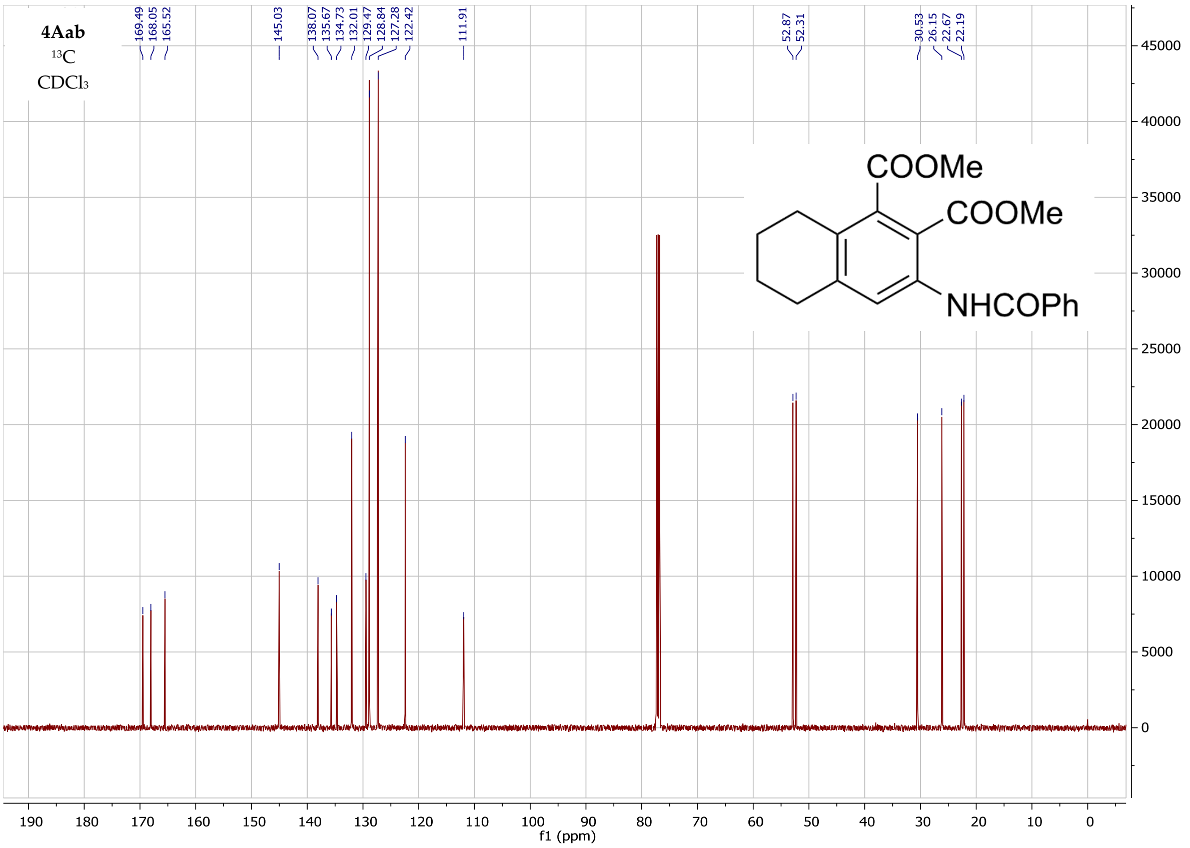

4Aab
^{13}C
$CDCl_3$
169.49
168.05
165.52
145.03
138.07
135.67
134.73
132.01
129.47
128.84
127.28
122.42
111.91
52.87
52.31
30.53
26.15
22.67
22.19
COOMe
COOMe
NHCOPh
f1 (ppm)

4Aac

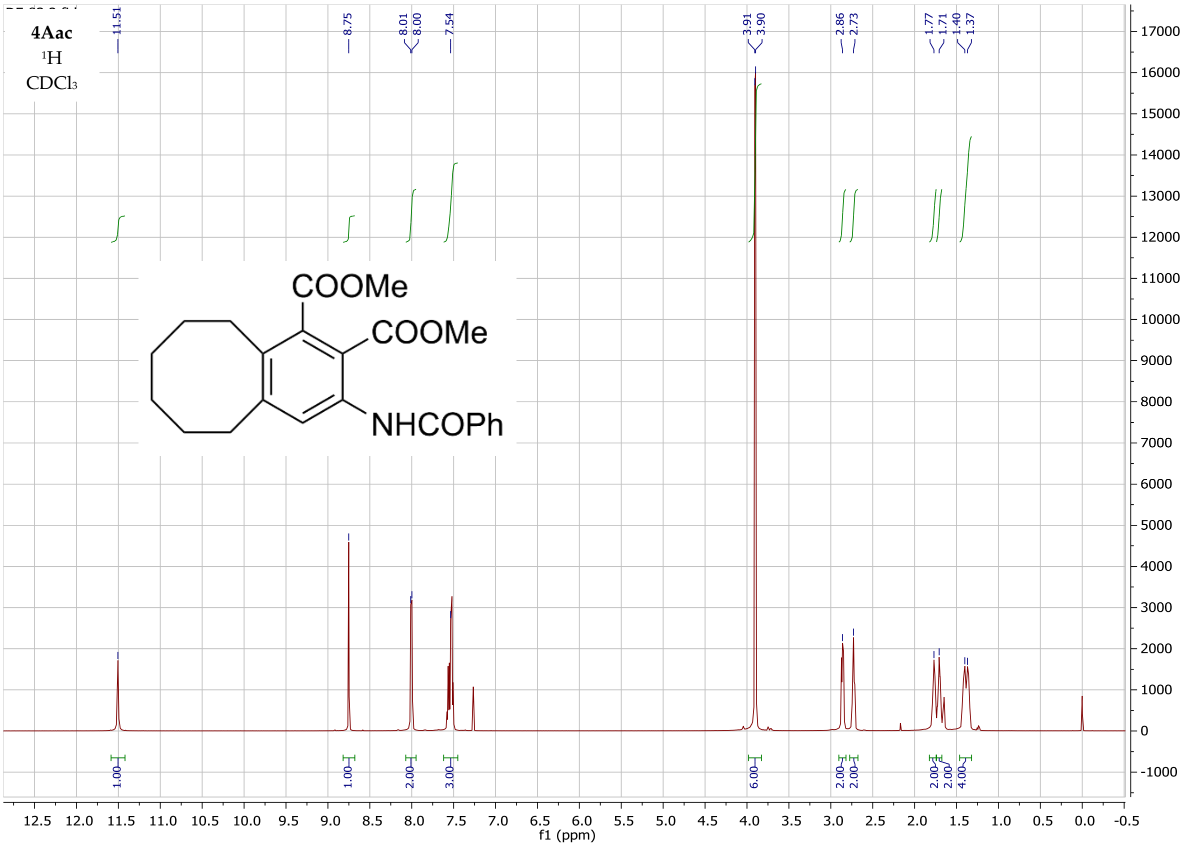
4Aac
1H
CDCl3
COOMe
COOMe
NHCOPh
11.51
8.75
8.01
8.00
7.54
3.91
3.90
2.86
2.73
1.77
1.71
1.40
1.37
1.00
1.00
2.00
3.00
6.00
2.00
2.00
2.00
2.00
4.00
f1 (ppm)

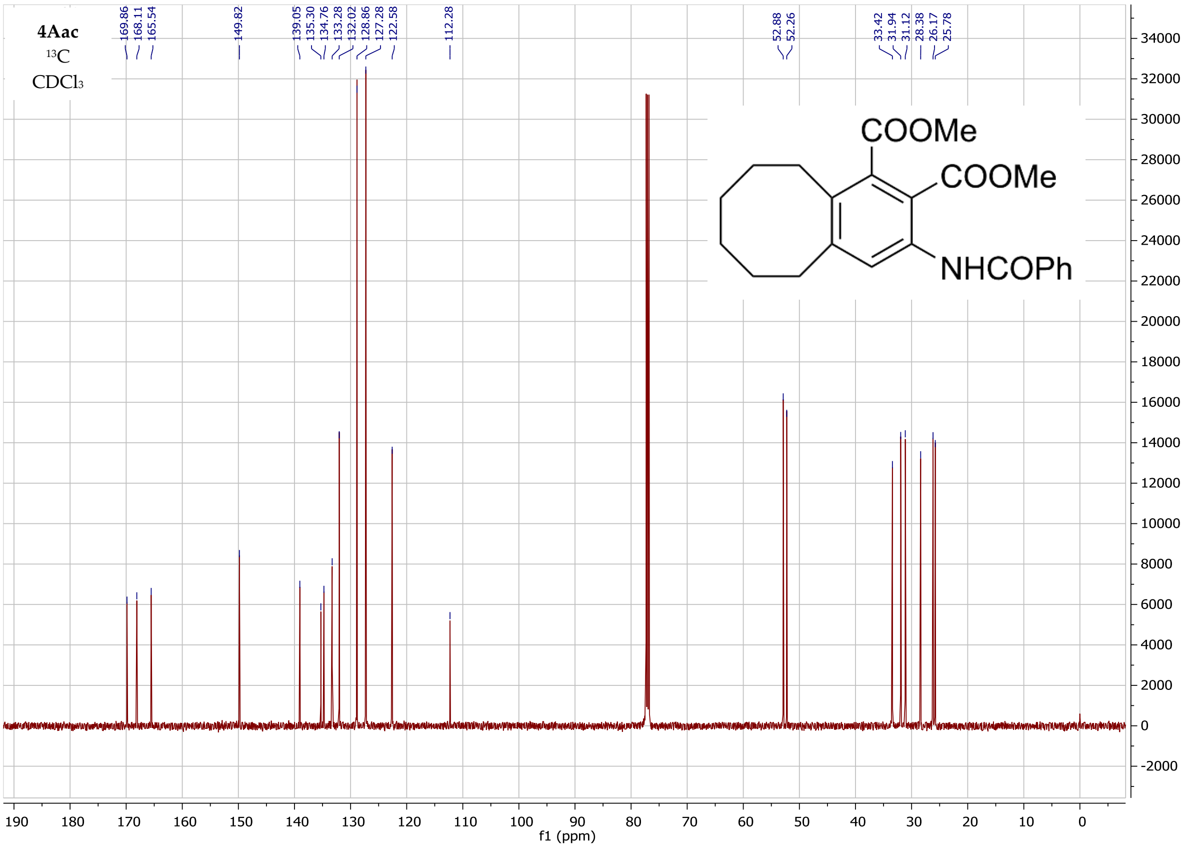
4Aac
13C
CDCl3
COOMe
COOMe
NHCOPh
169.86
168.11
165.54
149.82
139.05
135.30
134.76
133.28
132.02
128.86
127.28
122.58
112.28
52.88
52.26
33.42
31.94
31.12
28.38
26.17
25.78
f1 (ppm)

4Aad

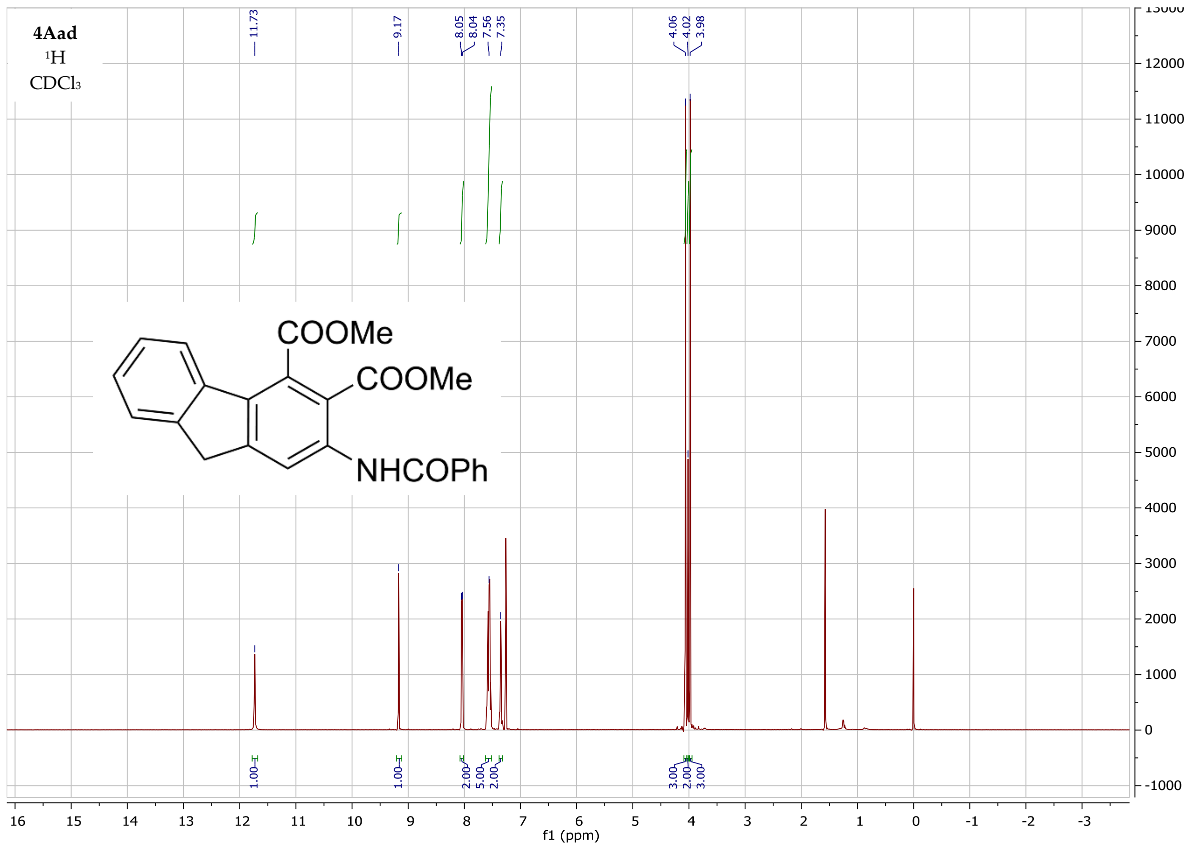
4Aad
1H
CDCl3
11.73
9.17
8.05
8.04
7.56
7.35
4.06
4.02
3.98
COOMe
COOMe
NHCOPh
1.00
1.00
2.00
5.00
2.00
3.00
2.00
3.00
f1 (ppm)

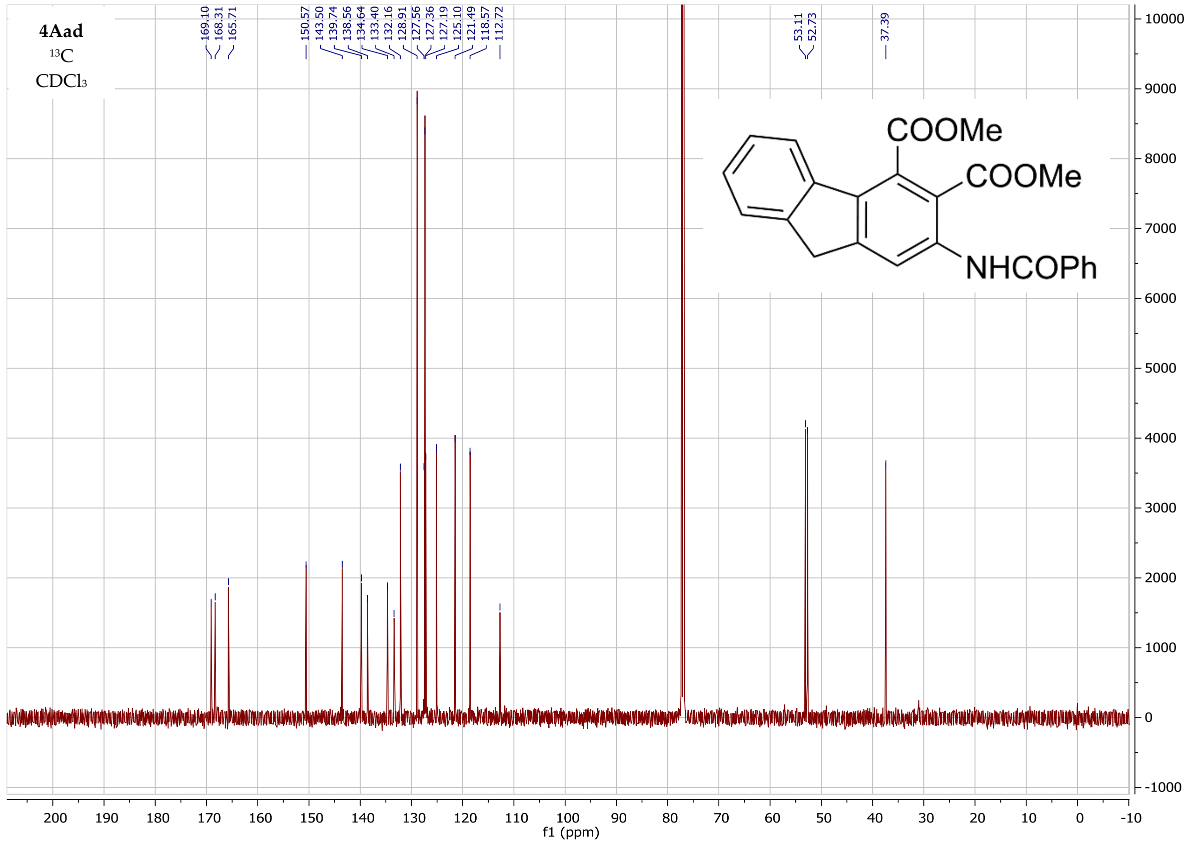
4Aad
13C
CDCl3
169.10
168.31
165.71
150.57
143.50
139.74
138.56
134.64
133.40
132.16
128.91
127.56
127.36
127.19
125.10
121.49
118.57
112.72
53.11
52.73
37.39
COOMe
COOMe
NHCOPh
f1 (ppm)

4Aae

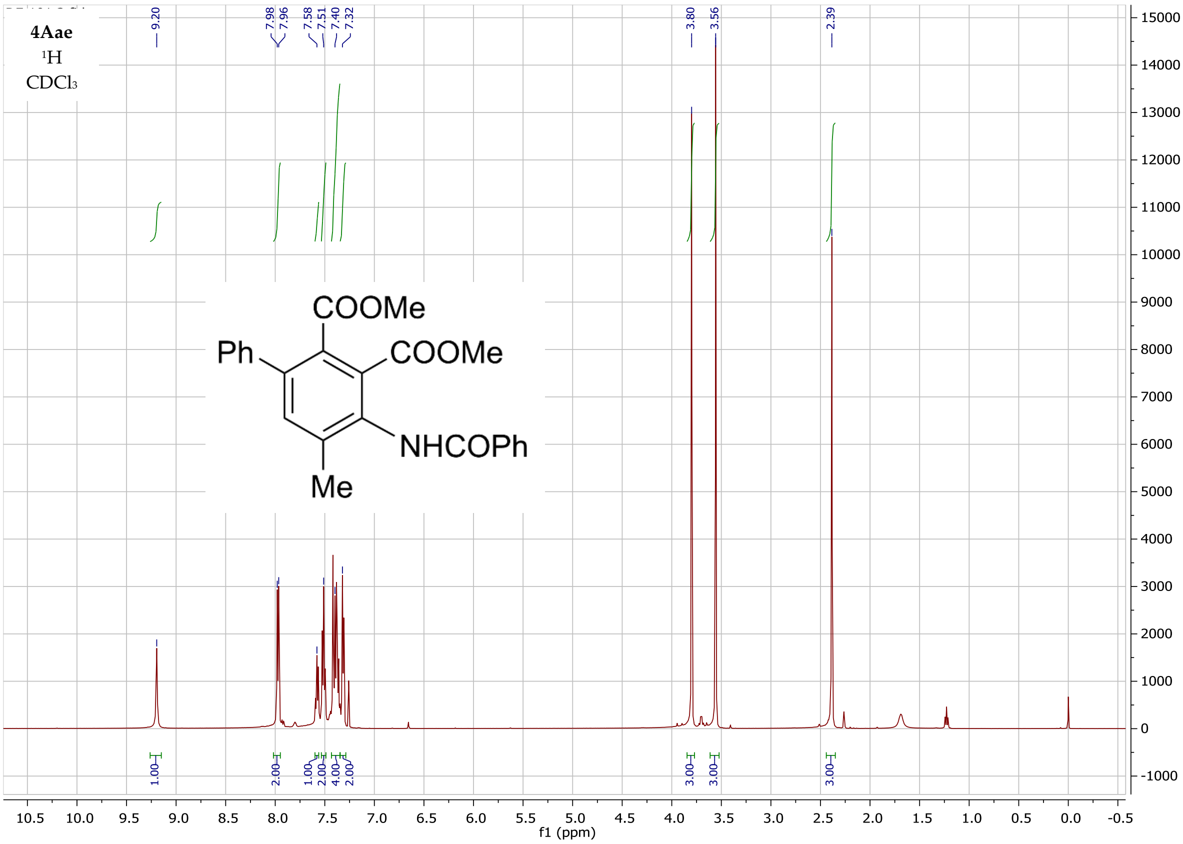
4Aae
1H
CDCl3
COOMe
Ph
COOMe
NHCOPh
Me
9.20
7.98
7.96
7.58
7.51
7.40
7.32
3.80
3.56
2.39
1.00
2.00
1.00
2.00
4.00
2.00
3.00
3.00
3.00
f1 (ppm)

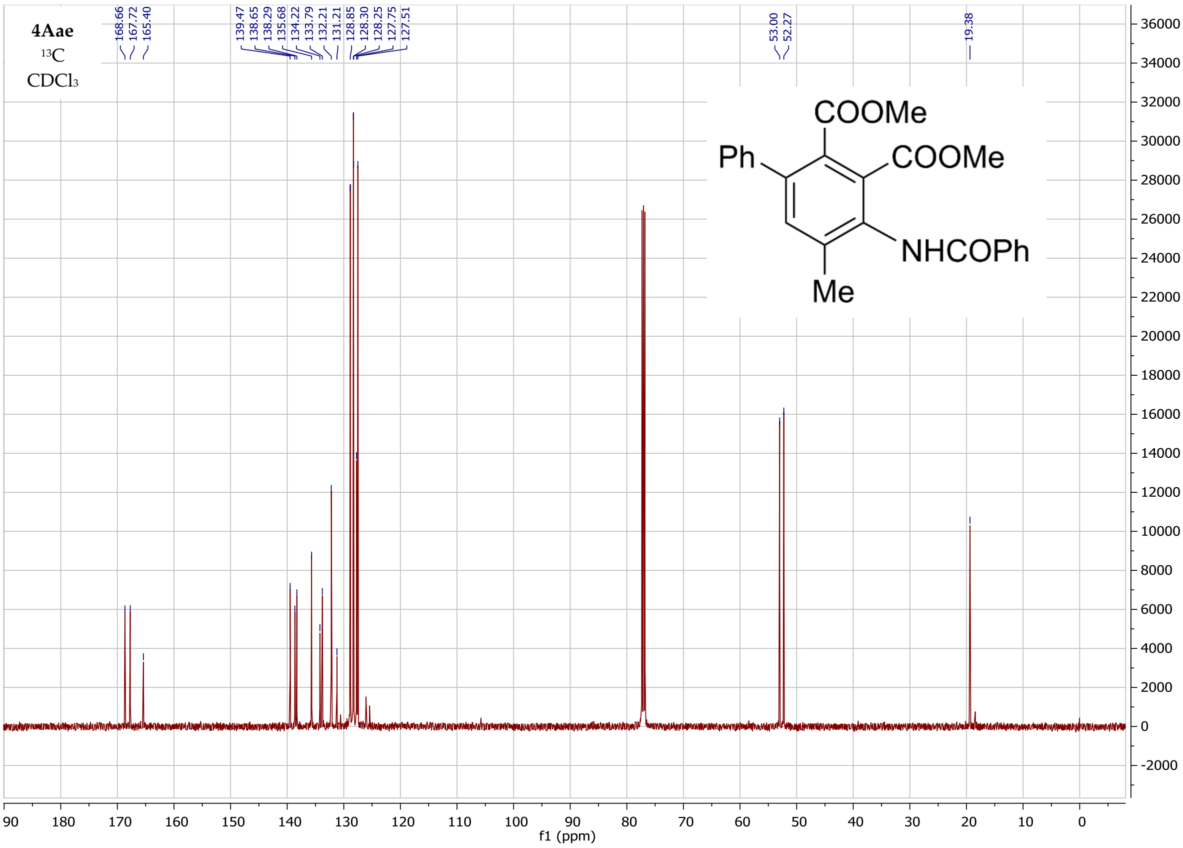
4Aae
13C
CDCl3
COOMe
Ph
COOMe
NHCOPh
Me
168.66
167.72
165.40
139.47
138.65
138.29
135.68
134.22
133.79
132.21
131.21
128.85
128.30
128.25
127.75
127.51
53.00
52.27
19.38
f1 (ppm)

4Aaf

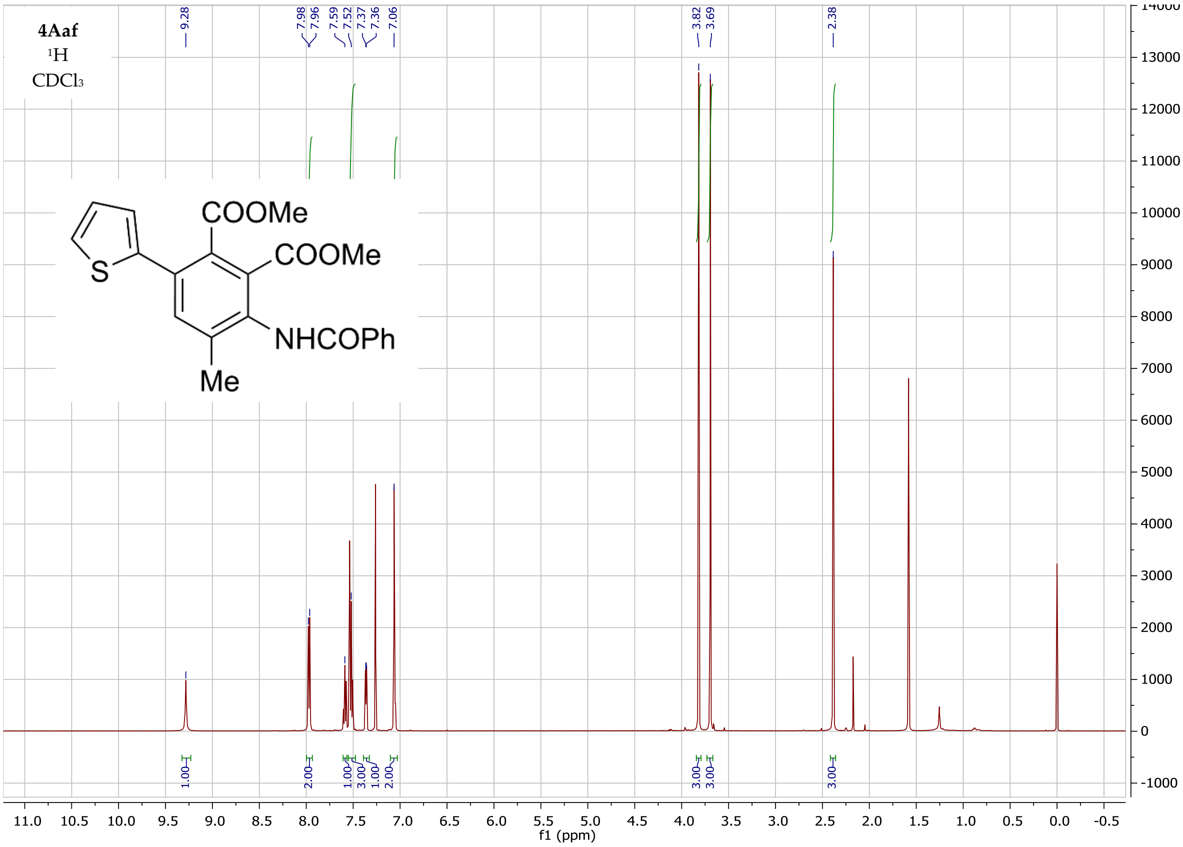
4Aaf
1H
CDCl3
9.28
7.98
7.96
7.59
7.52
7.37
7.36
7.06
3.82
3.69
2.38
COOMe
COOMe
NHCOPh
Me
S
1.00
2.00
1.00
3.00
1.00
2.00
3.00
3.00
3.00
f1 (ppm)

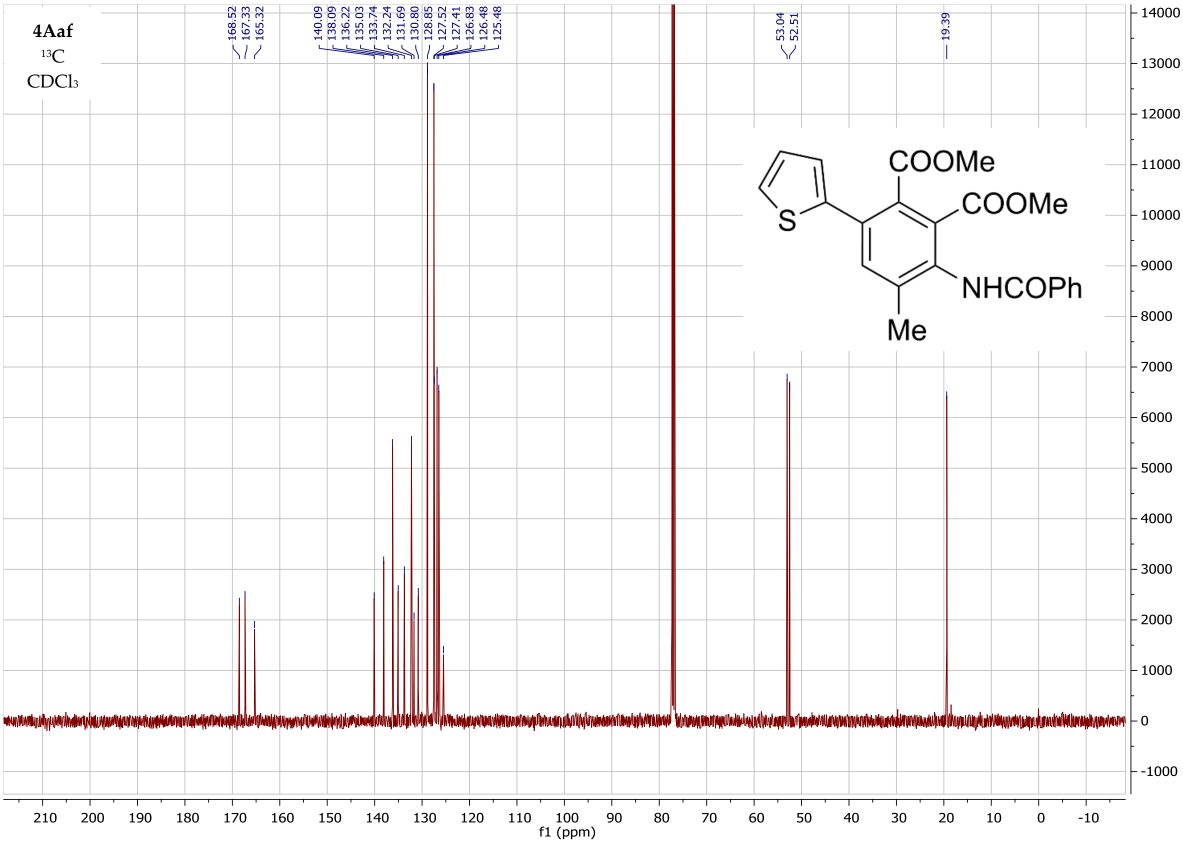
4Aaf
13C
CDCl3
168.52
167.33
165.32
140.09
138.09
136.22
135.03
133.74
132.24
131.69
130.80
128.85
127.52
127.41
126.83
126.48
125.48
53.04
52.51
19.39
COOMe
COOMe
NHCOPh
Me
S
f1 (ppm)

4Ba

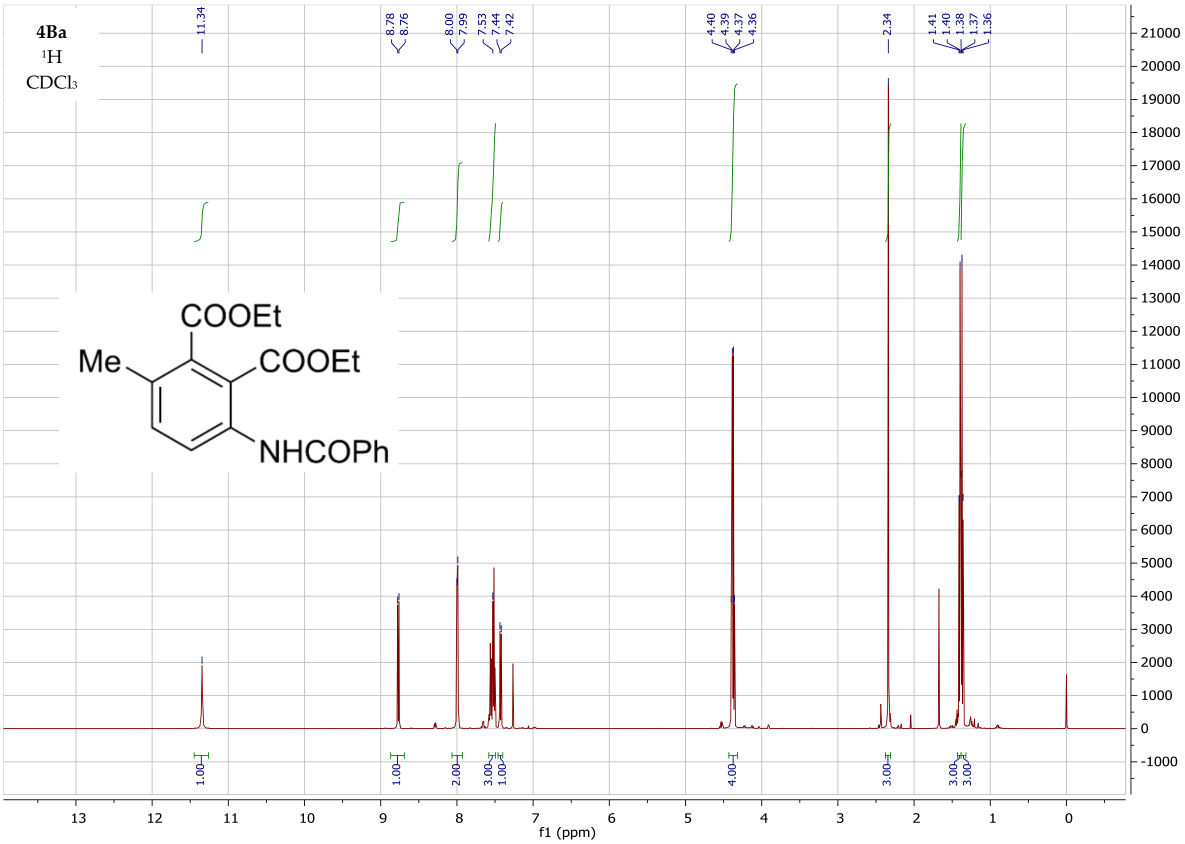
4Ba
1H
CDCl3
11.34
8.78
8.76
8.00
7.99
7.53
7.44
7.42
4.40
4.39
4.37
4.36
2.34
1.41
1.40
1.38
1.37
1.36
COOEt
Me
COOEt
NHCOPh
1.00
1.00
2.00
3.00
1.00
4.00
3.00
3.00
3.00
f1 (ppm)

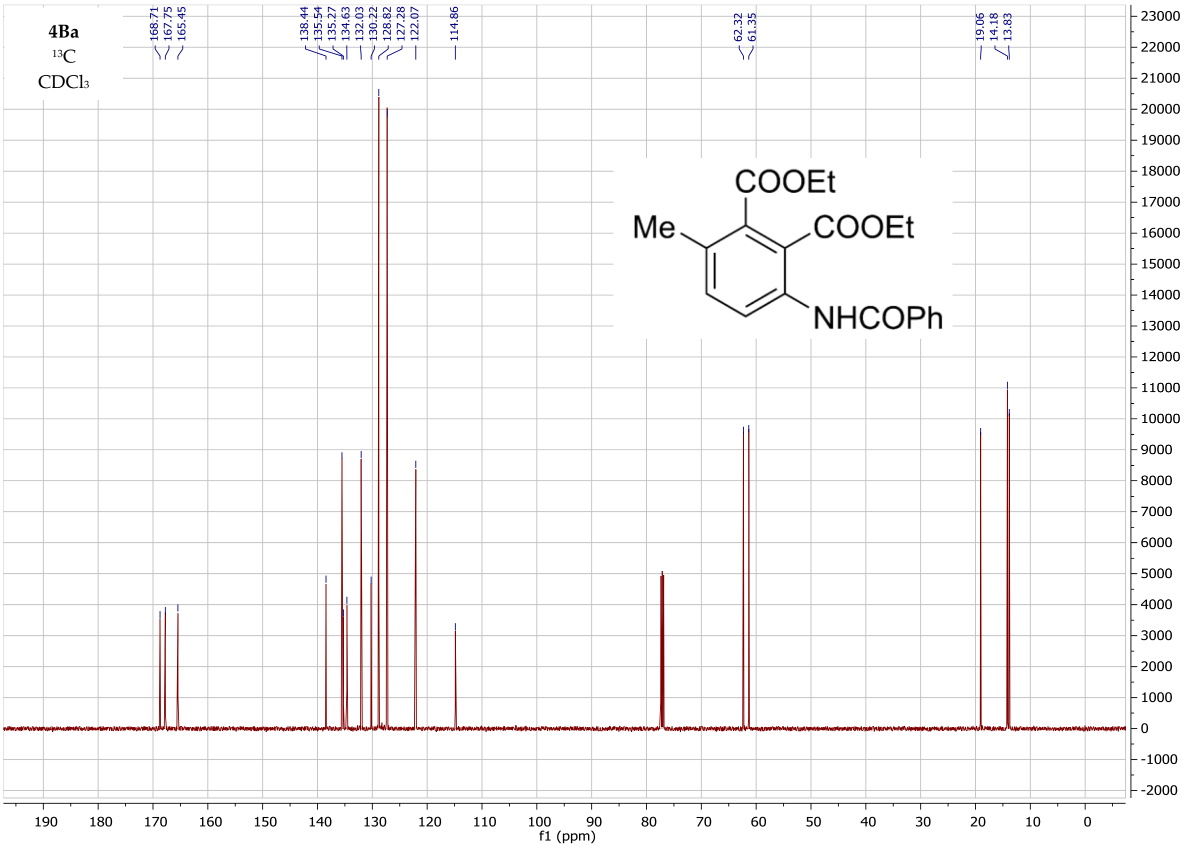
4Ba
13C
CDCl3
168.71
167.75
165.45
138.44
135.54
135.27
134.63
132.03
130.22
128.82
127.28
122.07
114.86
62.32
61.35
19.06
14.18
13.83
COOEt
Me
COOEt
NHCOPh
f1 (ppm)

4Bb

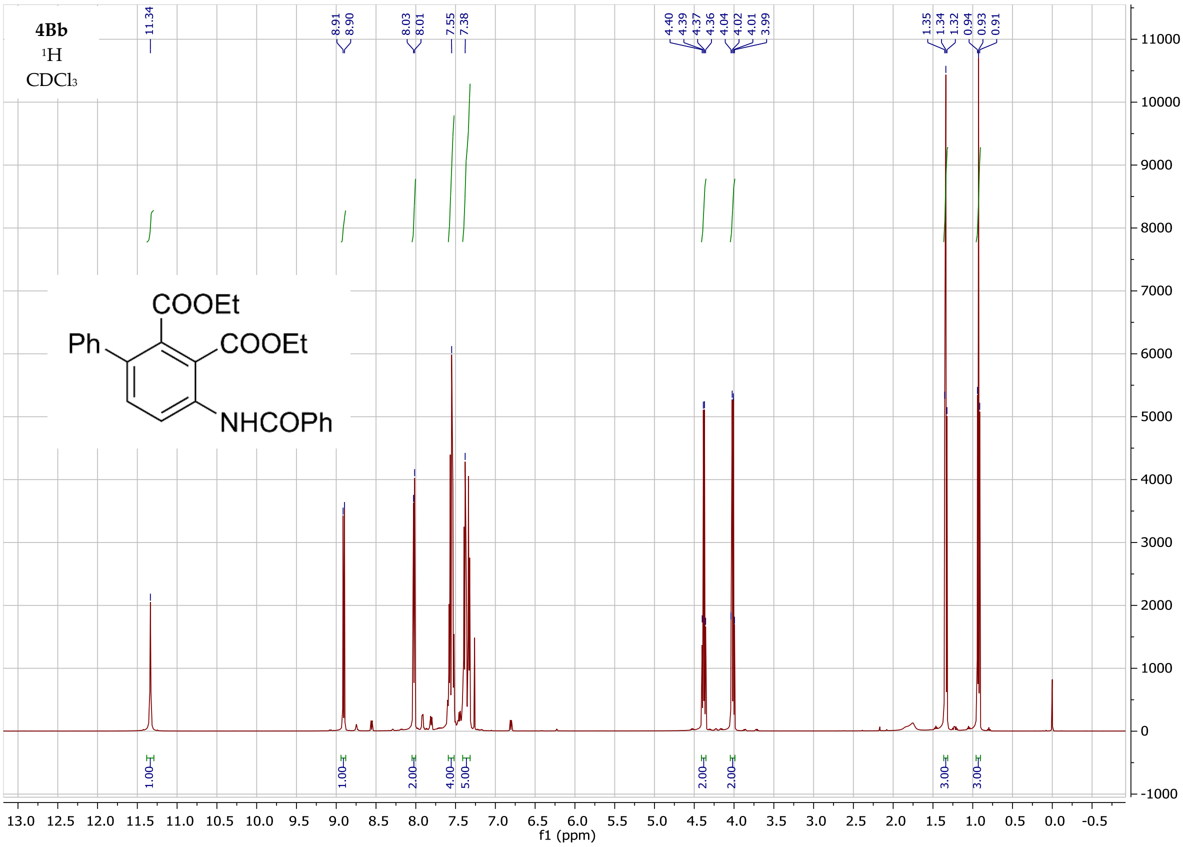
4Bb
1H
CDCl3
11.34
8.91
8.90
8.03
8.01
7.55
7.38
4.40
4.39
4.37
4.36
4.04
4.02
4.01
3.99
1.35
1.34
1.32
0.94
0.93
0.91
COOEt
Ph
COOEt
NHCOPh
1.00
1.00
2.00
4.00
5.00
2.00
2.00
3.00
3.00
f1 (ppm)

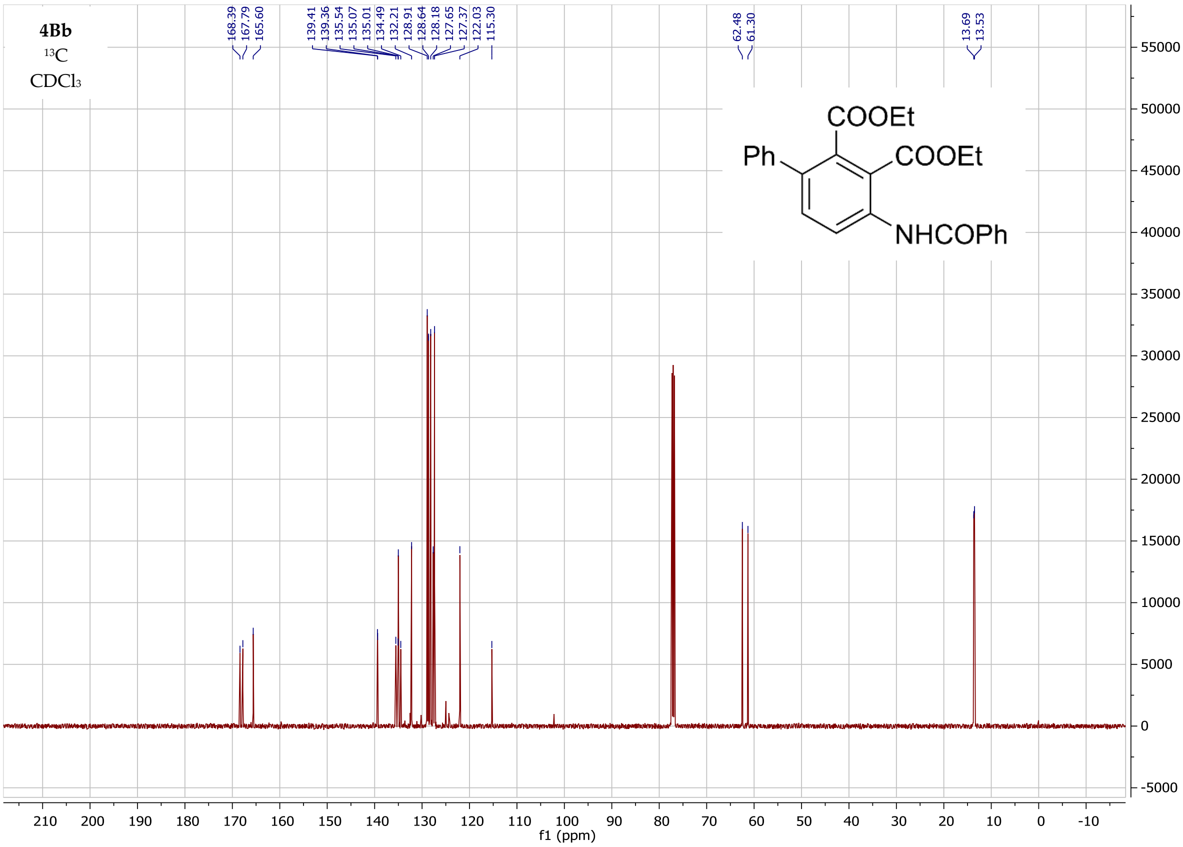
4Bb
13C
CDCl3
168.39
167.79
165.60
139.41
139.36
135.54
135.07
135.01
134.49
132.21
128.91
128.64
128.18
127.65
127.37
122.03
115.30
62.48
61.30
13.69
13.53
COOEt
Ph
COOEt
NHCOPh
f1 (ppm)

4Bc

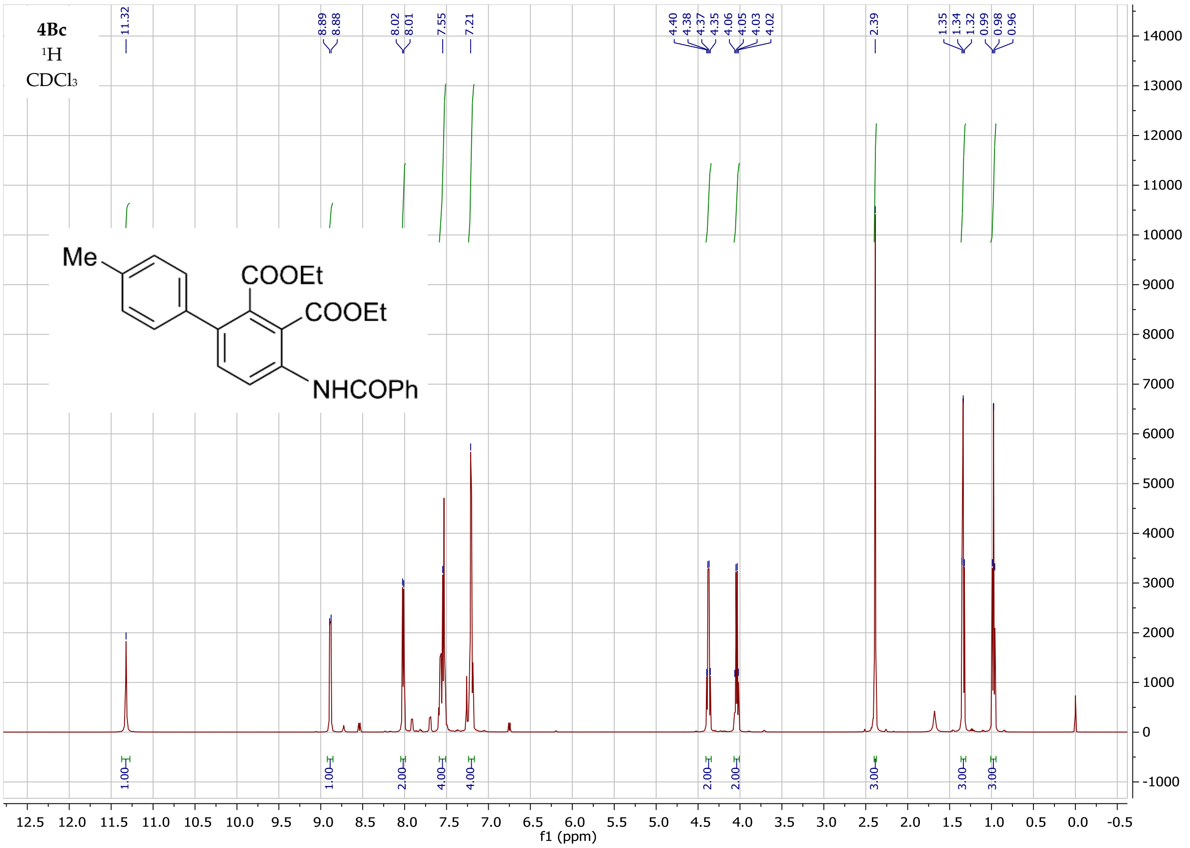
4Bc
1H
CDCl3
11.32
8.89
8.88
8.02
8.01
7.55
7.21
4.40
4.38
4.37
4.35
4.06
4.05
4.03
4.02
2.39
1.35
1.34
1.32
0.99
0.98
0.96
Me
COOEt
COOEt
NHCOPh
1.00
1.00
2.00
4.00
4.00
2.00
2.00
3.00
3.00
3.00
f1 (ppm)

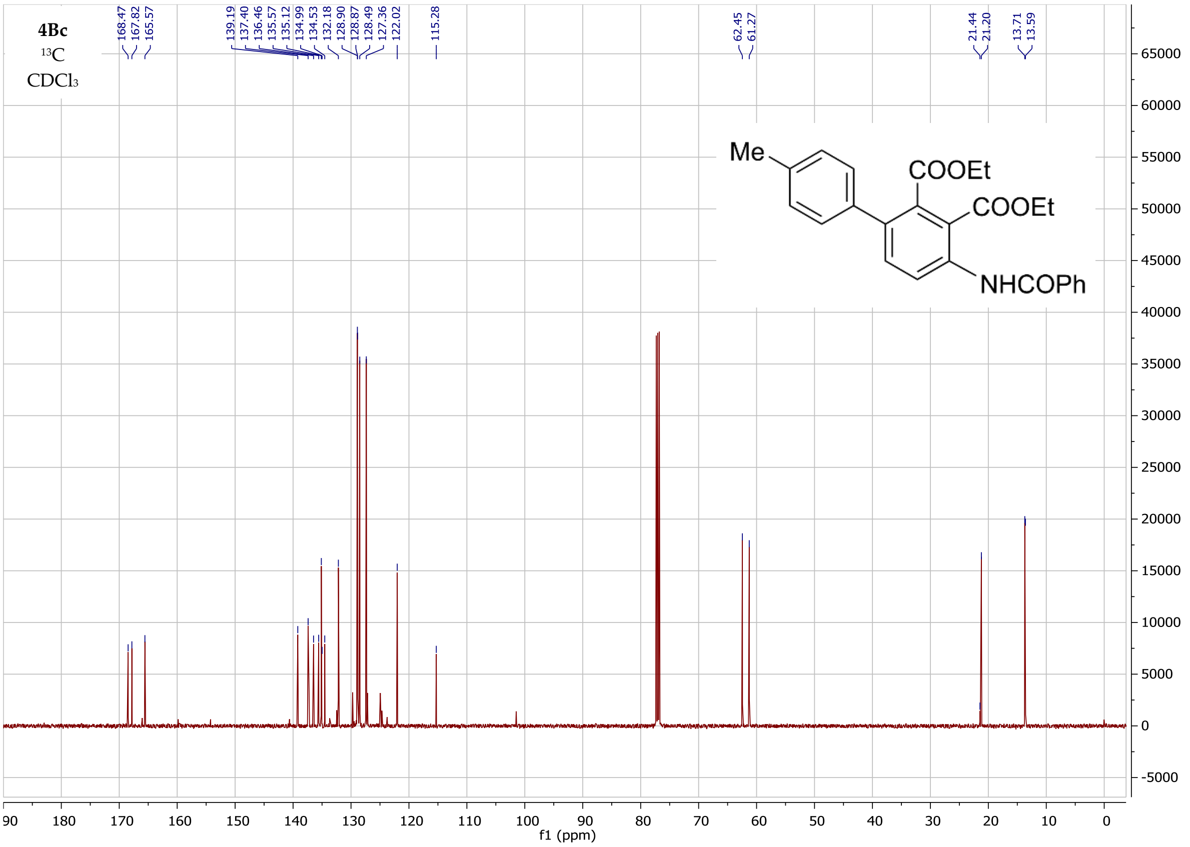
4Bc
13C
CDCl3
168.47
167.82
165.57
139.19
137.40
136.46
135.57
135.12
134.99
134.53
132.18
128.90
128.87
128.49
127.36
122.02
115.28
62.45
61.27
21.44
21.20
13.71
13.59
Me
COOEt
COOEt
NHCOPh
f1 (ppm)

4Bd

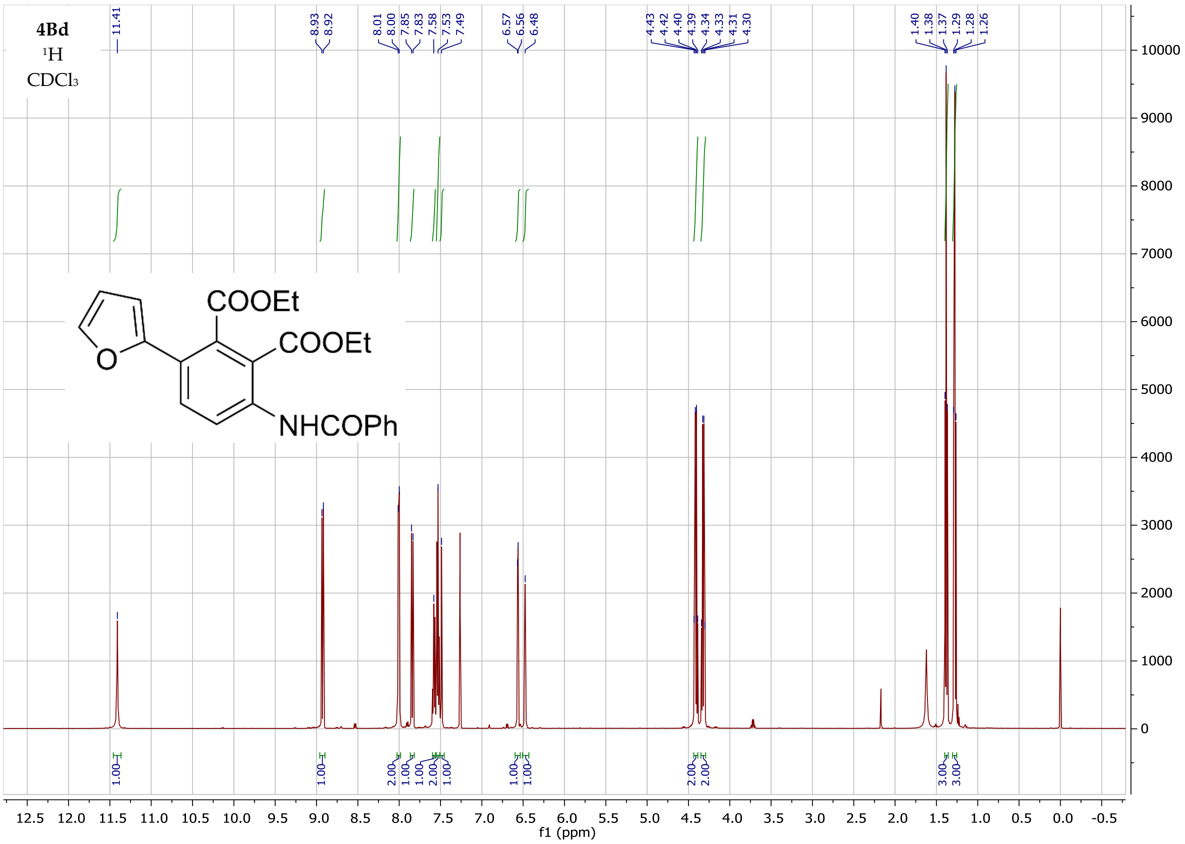

4Bd
¹H
CDCl₃
COOEt
COOEt
NHCOPh
O
f1 (ppm)

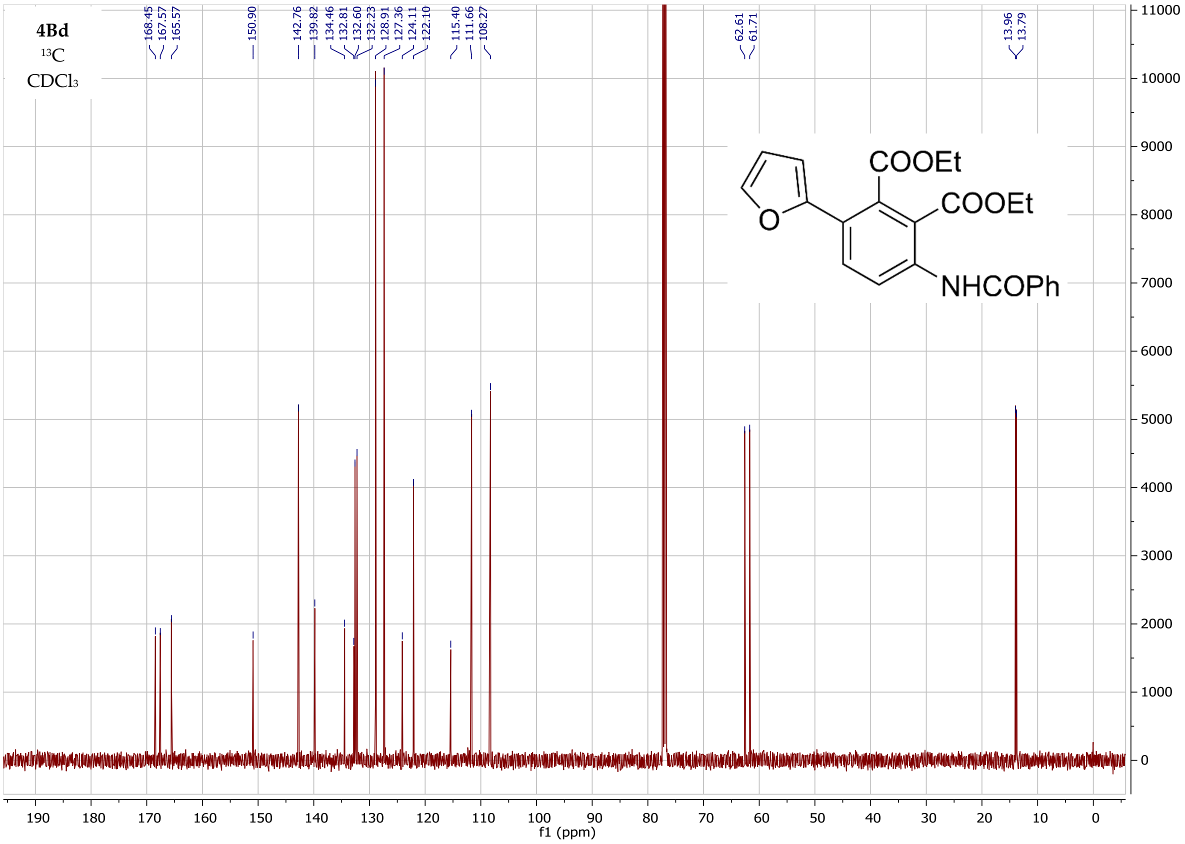

4Bd
¹³C
CDCl₃
COOEt
COOEt
NHCOPh
O
f1 (ppm)

4Be

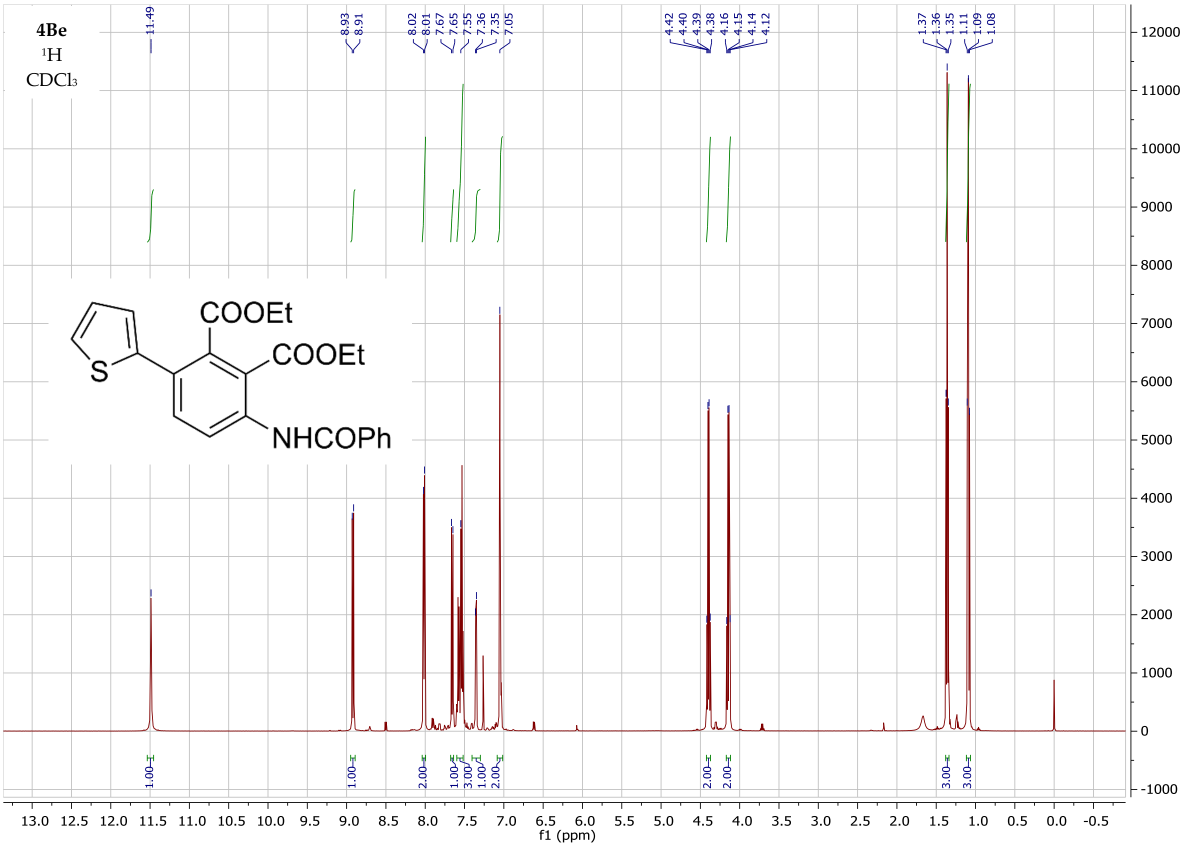
4Be
1H
CDCl3
COOEt
COOEt
NHCOPh
S
f1 (ppm)

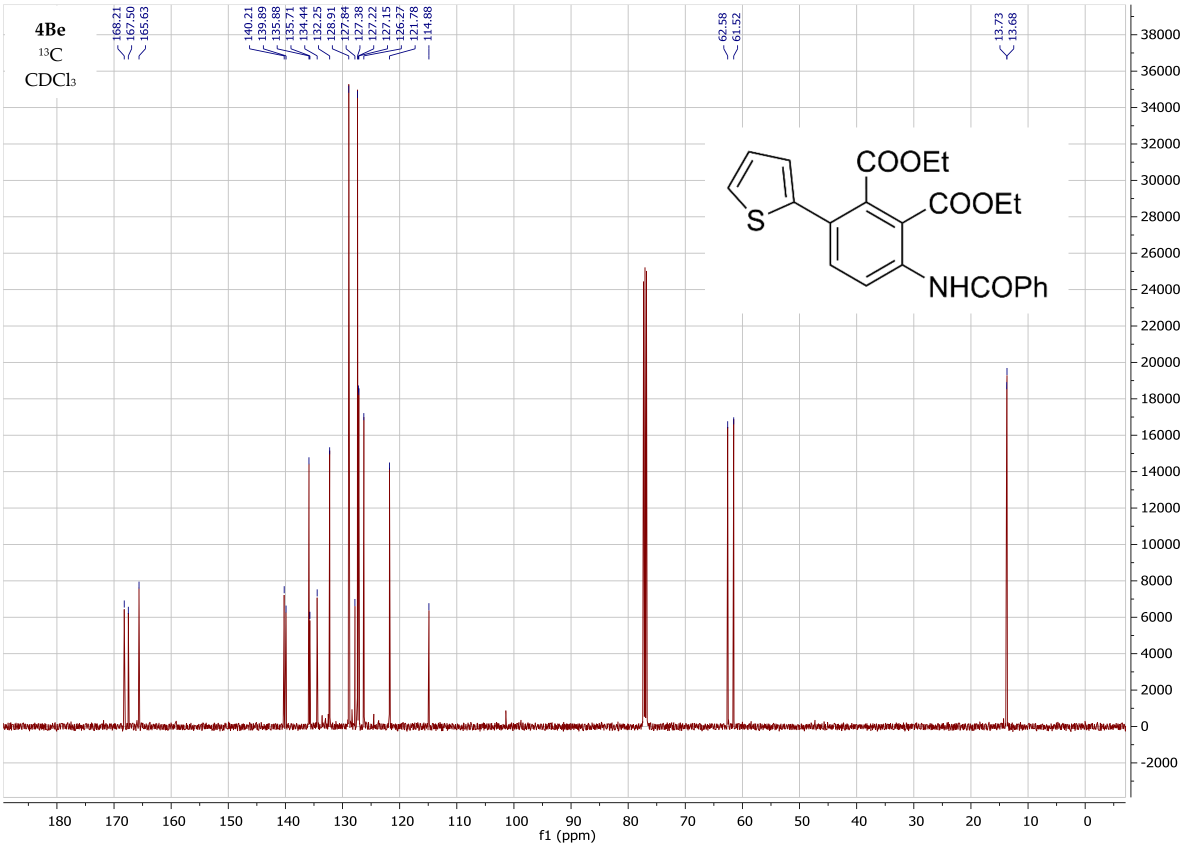
4Be
13C
CDCl3
COOEt
COOEt
NHCOPh
S
f1 (ppm)

4Bf

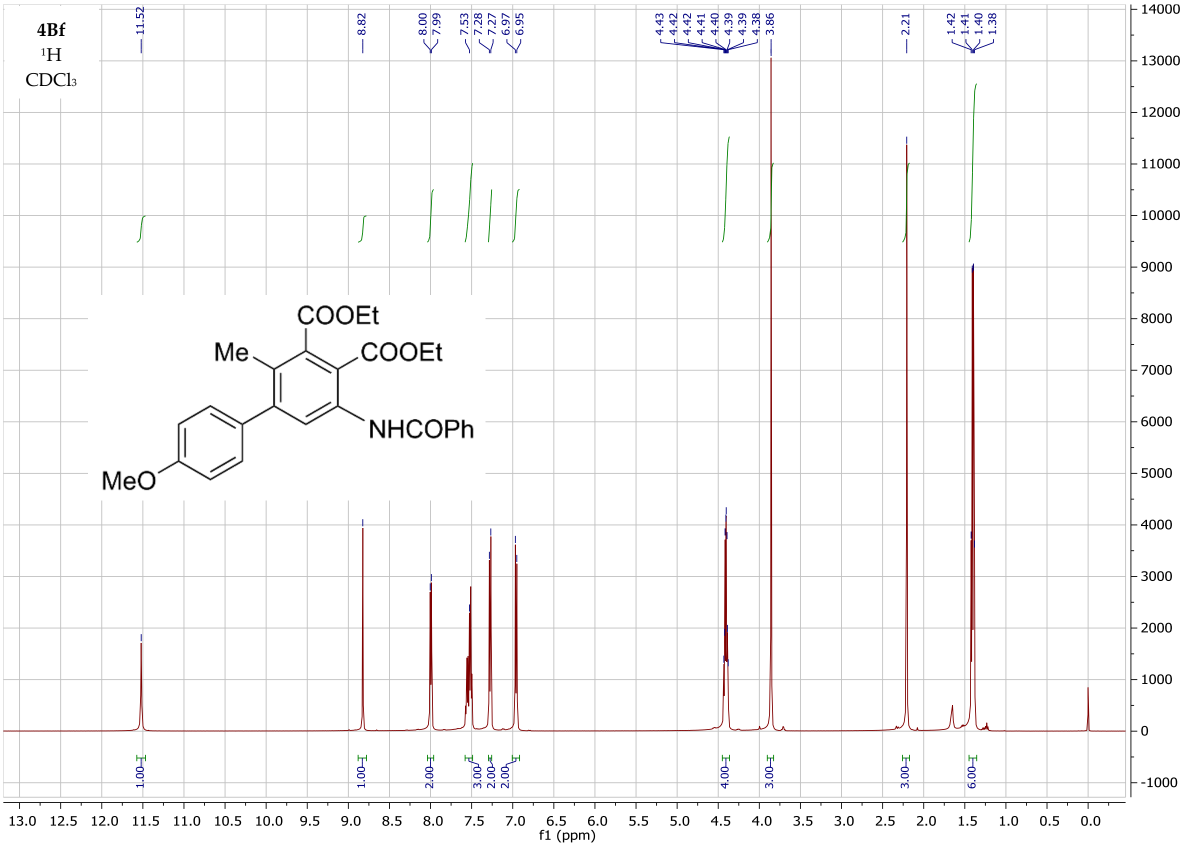

4Bf
^{1}H
CDCl$_3$
11.52
8.82
8.00
7.99
7.53
7.28
7.27
6.97
6.95
4.43
4.42
4.42
4.41
4.40
4.39
4.39
4.38
3.86
2.21
1.42
1.41
1.40
1.38
COOEt
Me
COOEt
NHCOPh
MeO
1.00
1.00
2.00
3.00
2.00
2.00
4.00
3.00
3.00
6.00
f1 (ppm)

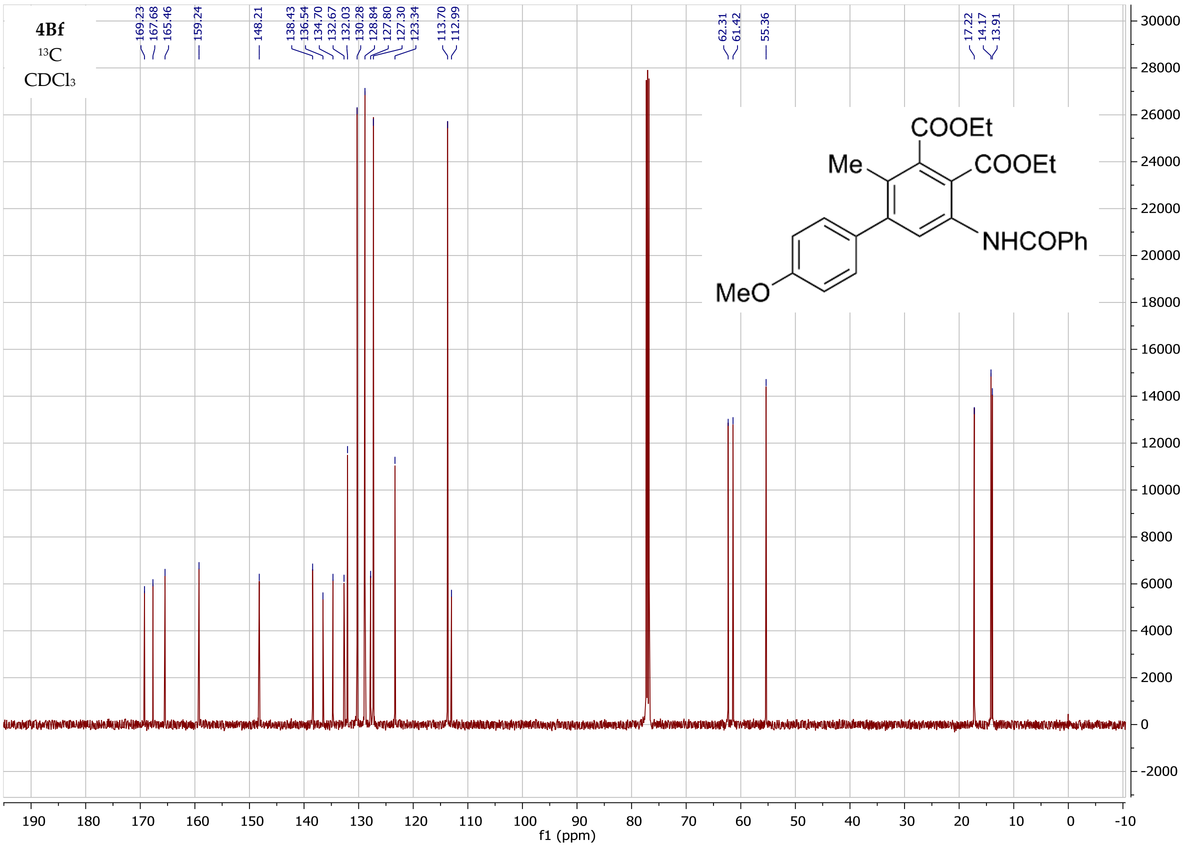

4Bf
^{13}C
CDCl$_3$
169.23
167.68
165.46
159.24
148.21
138.43
136.54
134.70
132.67
132.03
130.28
128.84
127.80
127.30
123.34
113.70
112.99
62.31
61.42
55.36
17.22
14.17
13.91
COOEt
Me
COOEt
NHCOPh
MeO
f1 (ppm)

4Bg

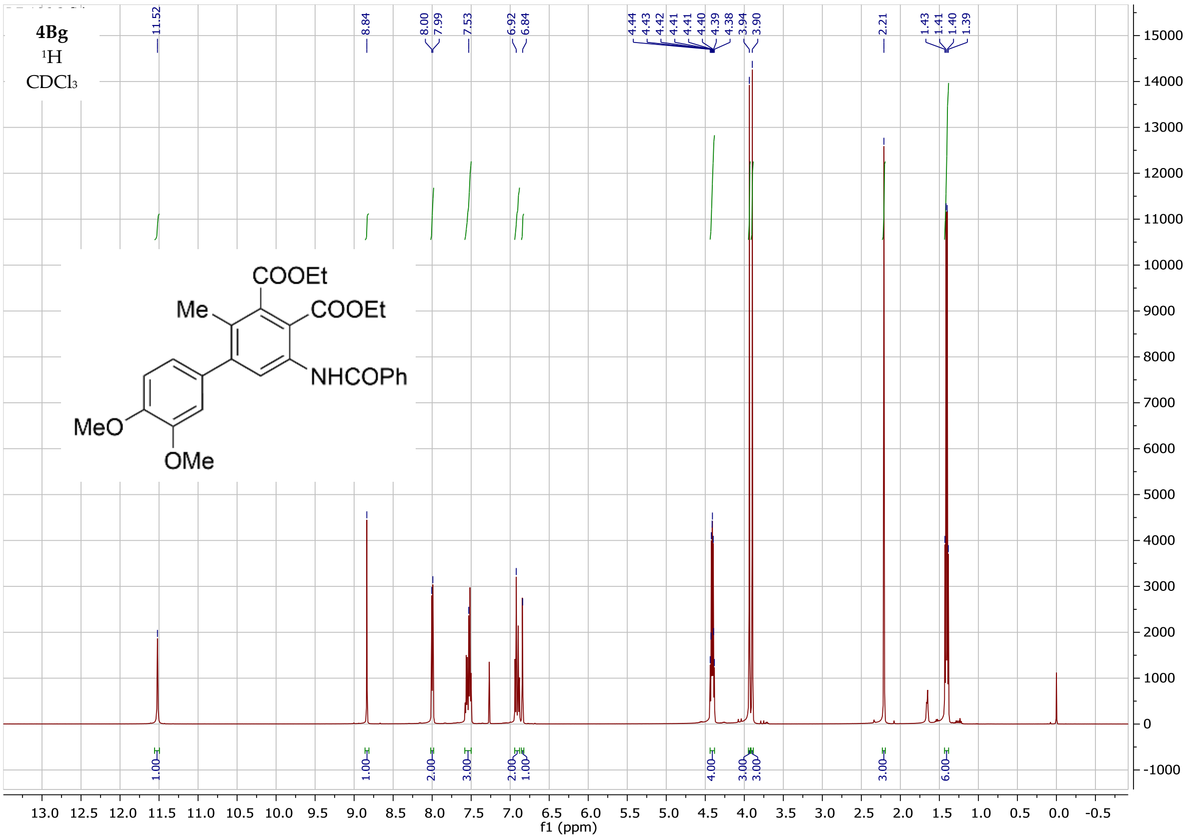
4Bg
1H
CDCl3
COOEt
Me
COOEt
NHCOPh
MeO
OMe
11.52
8.84
8.00
7.99
7.53
6.92
6.84
4.44
4.43
4.42
4.41
4.41
4.40
4.39
4.38
3.94
3.90
2.21
1.43
1.41
1.40
1.39
1.00
1.00
2.00
3.00
2.00
1.00
4.00
3.00
3.00
3.00
6.00
f1 (ppm)

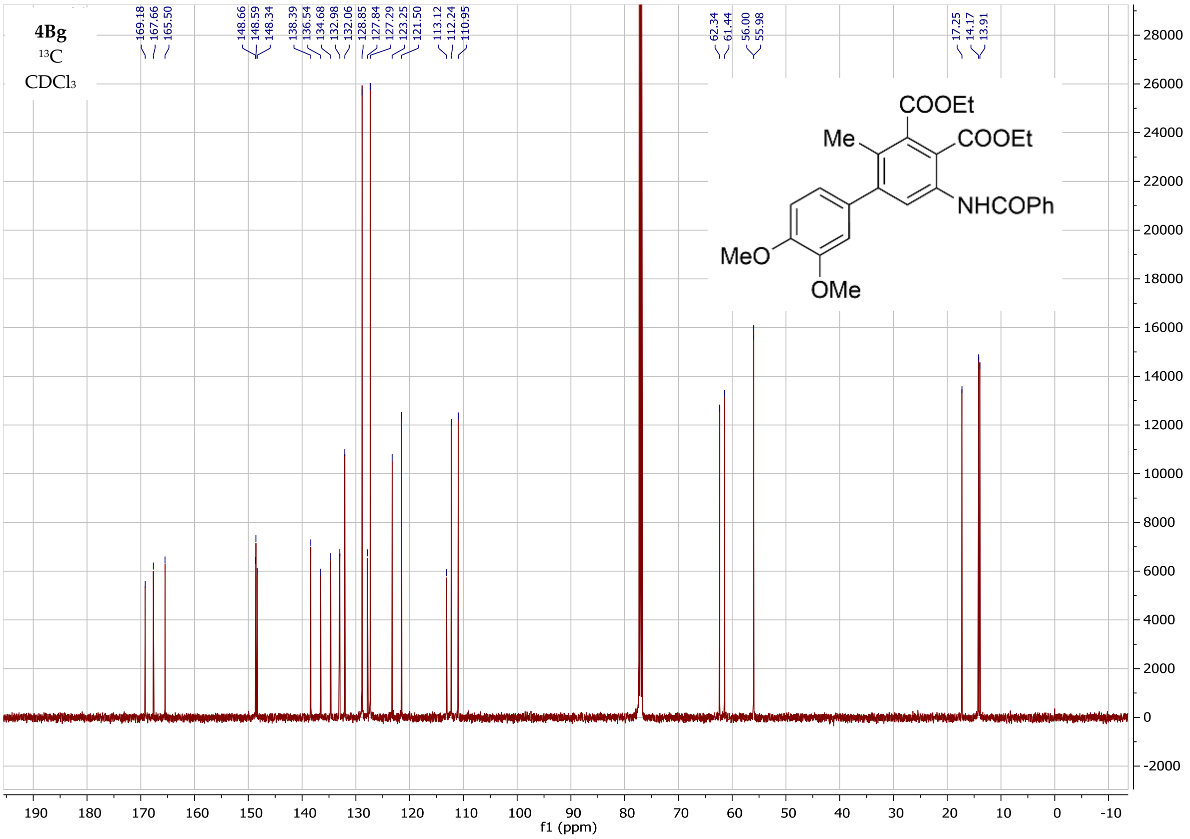
4Bg
13C
CDCl3
169.18
167.66
165.50
148.66
148.59
148.34
138.39
136.54
134.68
132.98
132.06
128.85
127.84
127.29
123.25
121.50
113.12
112.24
110.95
62.34
61.44
56.00
55.98
17.25
14.17
13.91
COOEt
Me
COOEt
NHCOPh
MeO
OMe
f1 (ppm)

4Bh

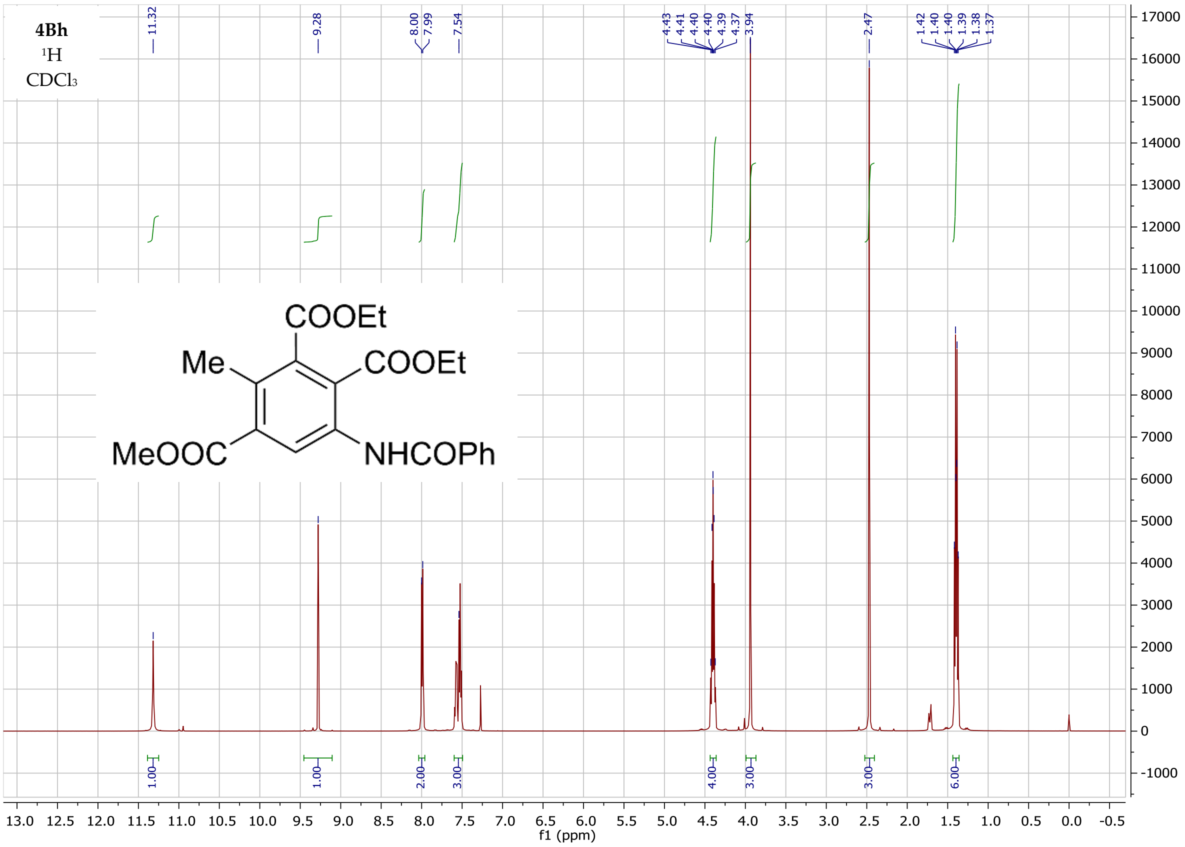
4Bh
1H
CDCl3
11.32
9.28
8.00
7.99
7.54
4.43
4.41
4.40
4.40
4.39
4.37
3.94
2.47
1.42
1.40
1.40
1.39
1.38
1.37
COOEt
Me
COOEt
MeOOC
NHCOPh
1.00
1.00
2.00
3.00
4.00
3.00
3.00
6.00
f1 (ppm)

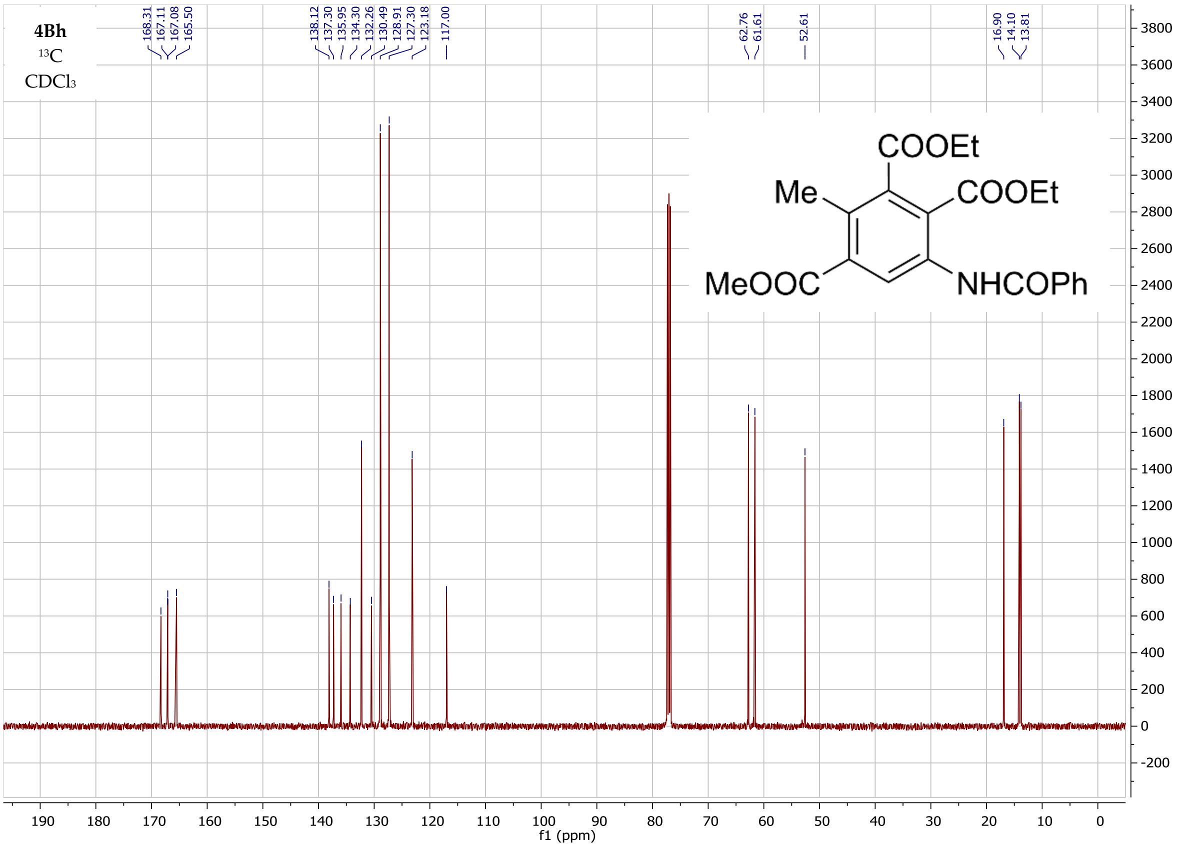
4Bh
13C
CDCl3
168.31
167.11
167.08
165.50
138.12
137.30
135.95
134.30
132.26
130.49
128.91
127.30
123.18
117.00
62.76
61.61
52.61
16.90
14.10
13.81
COOEt
Me
COOEt
MeOOC
NHCOPh
f1 (ppm)

4Bi

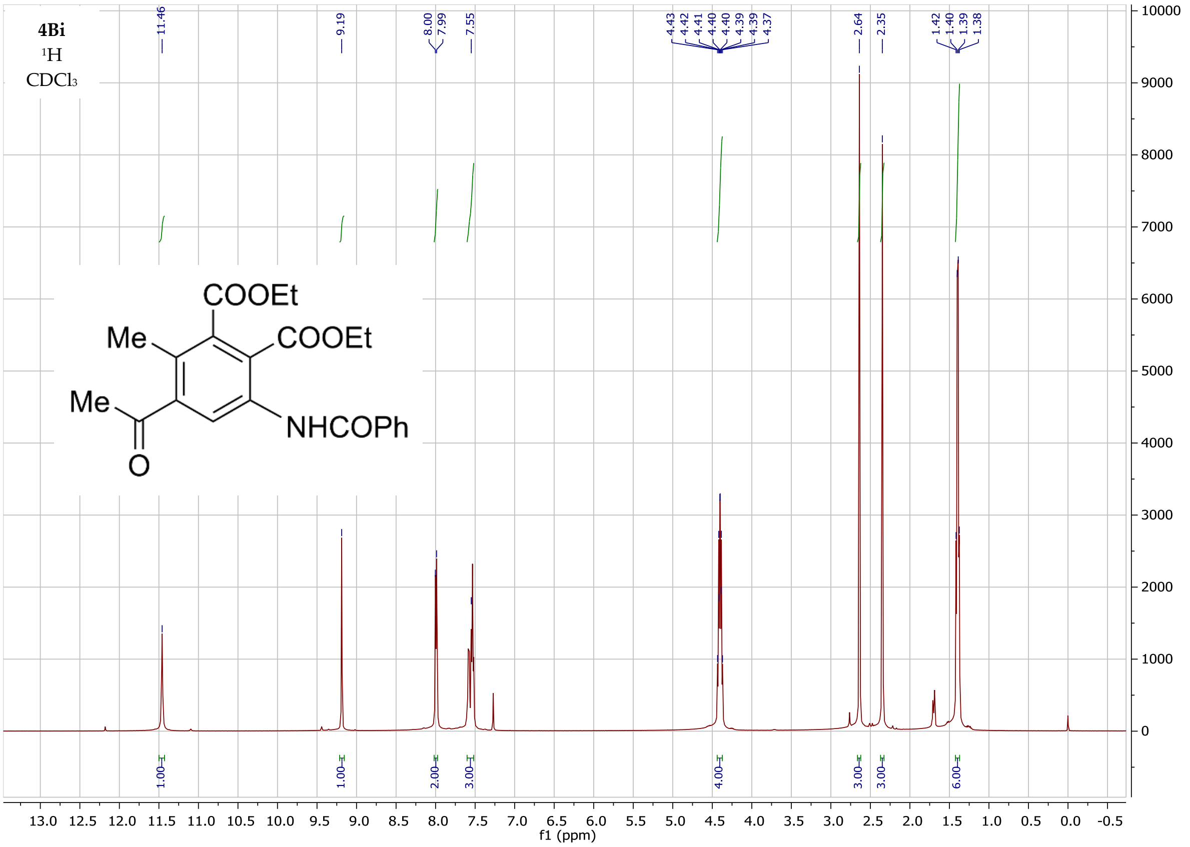
4Bi
1H
CDCl3
11.46
9.19
8.00
7.99
7.55
4.43
4.42
4.41
4.40
4.40
4.39
4.39
4.37
2.64
2.35
1.42
1.40
1.39
1.38
COOEt
Me
COOEt
Me
NHCOPh
O
1.00
1.00
2.00
3.00
4.00
3.00
3.00
6.00
f1 (ppm)

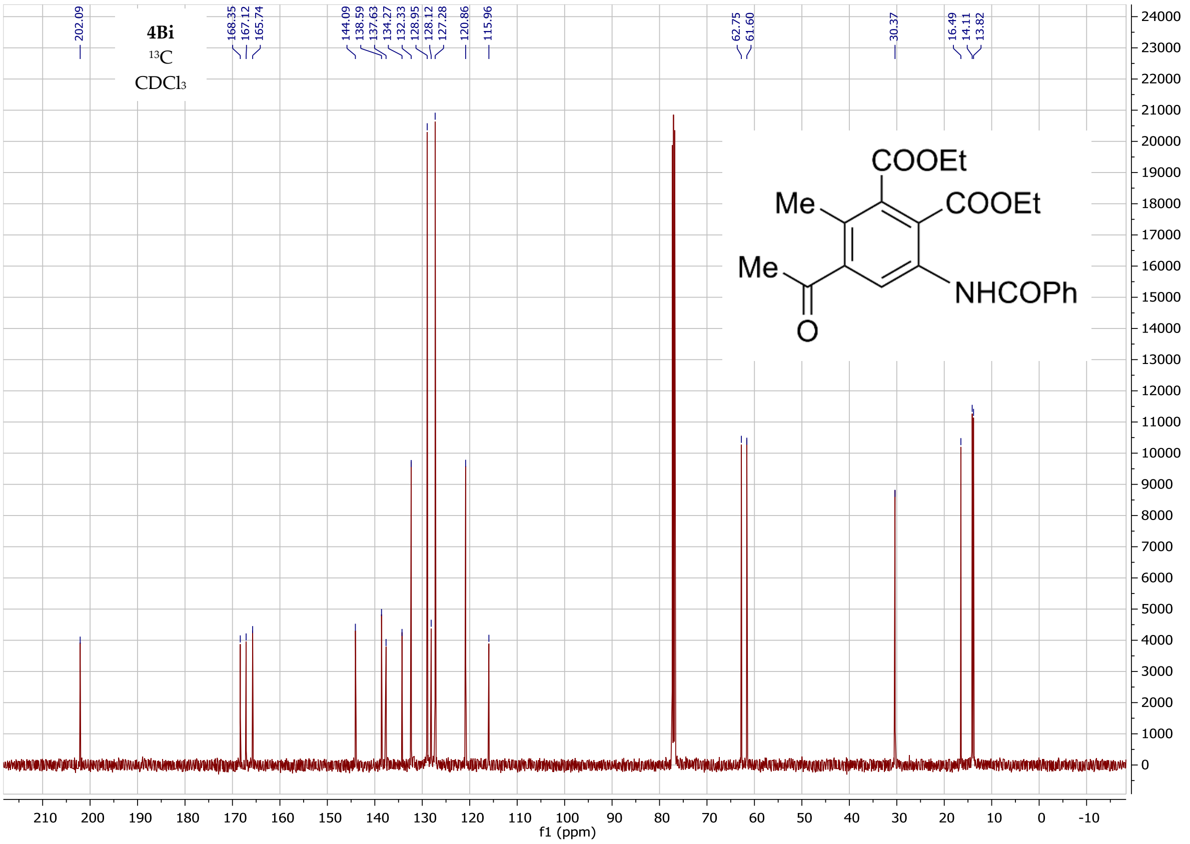
4Bi
13C
CDCl3
202.09
168.35
167.12
165.74
144.09
138.59
137.63
134.27
132.33
128.95
128.12
127.28
120.86
115.96
62.75
61.60
30.37
16.49
14.11
13.82
COOEt
Me
COOEt
Me
NHCOPh
O
f1 (ppm)

4Bj

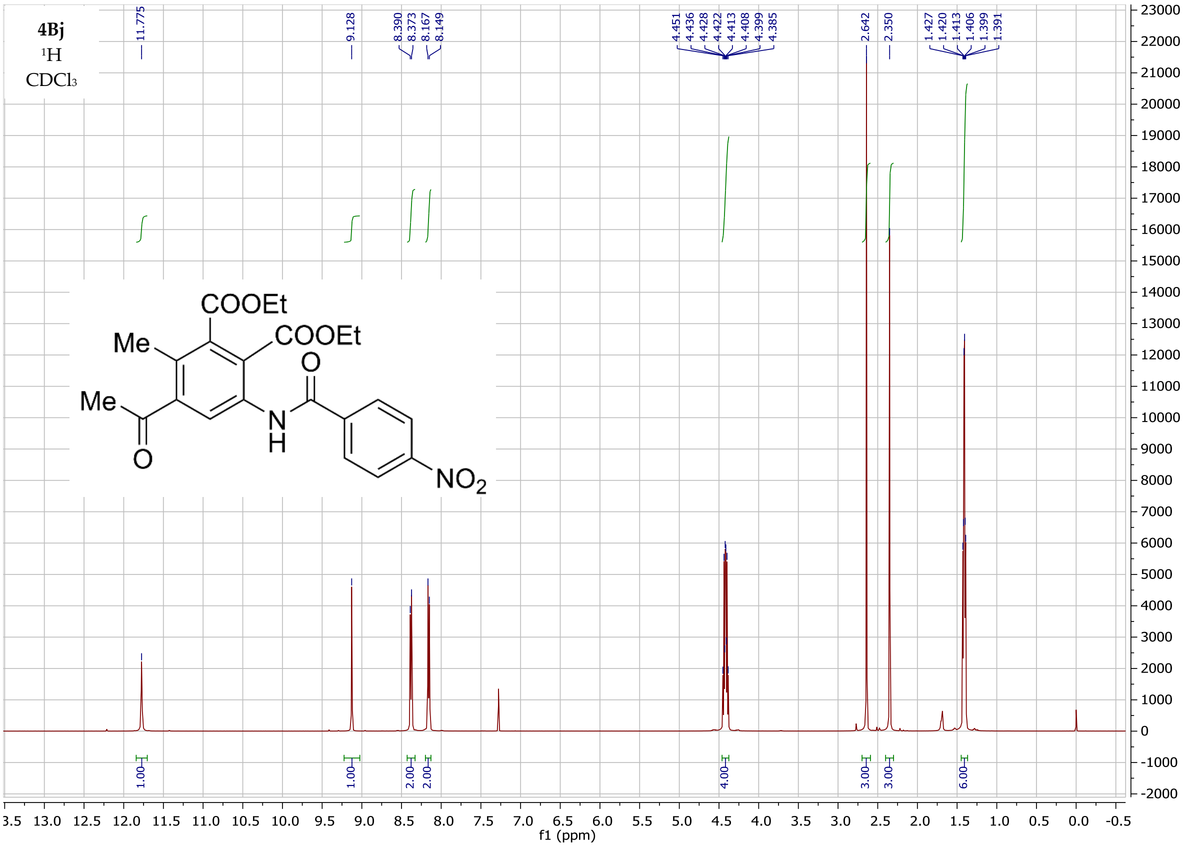

4Bj
^{1}H
CDCl3
COOEt
COOEt
Me
Me
O
N
H
O
NO2
f1 (ppm)

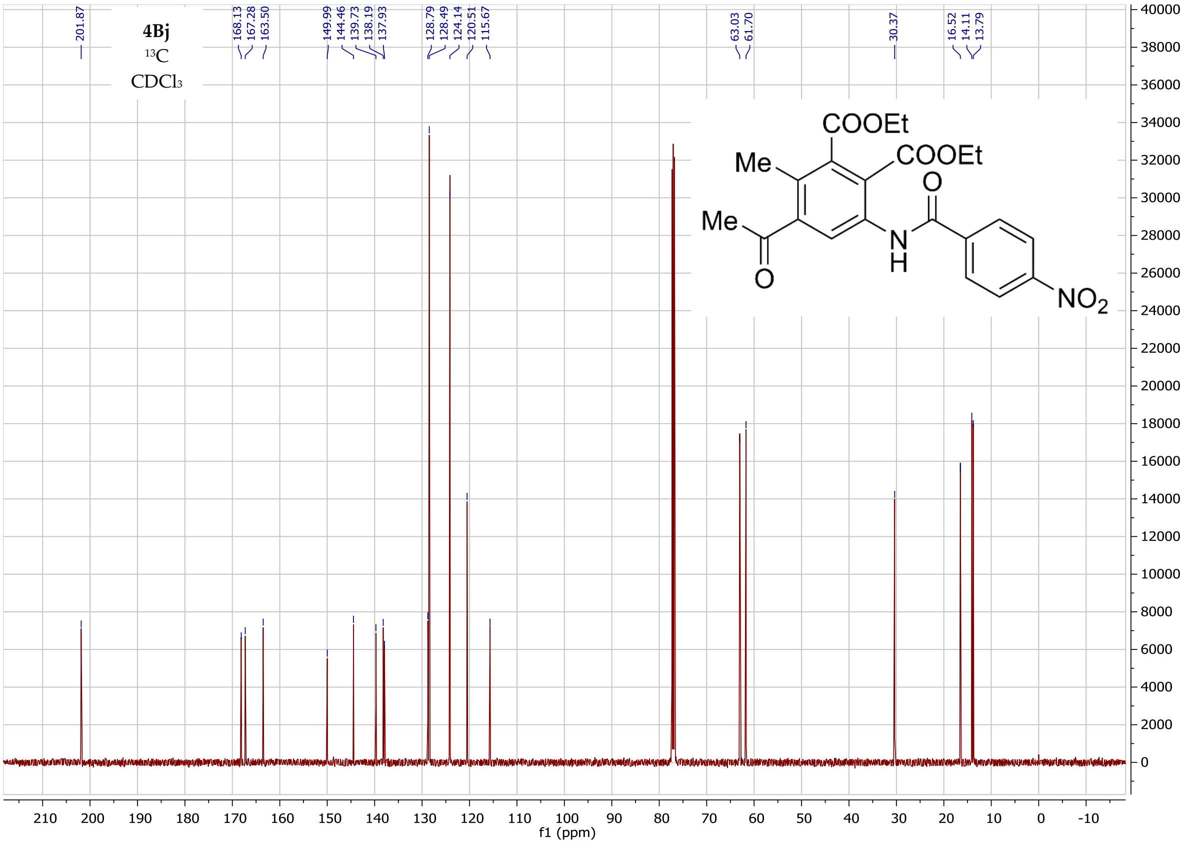

4Bj
^{13}C
CDCl3
COOEt
COOEt
Me
Me
O
N
H
O
NO2
f1 (ppm)

4Bk

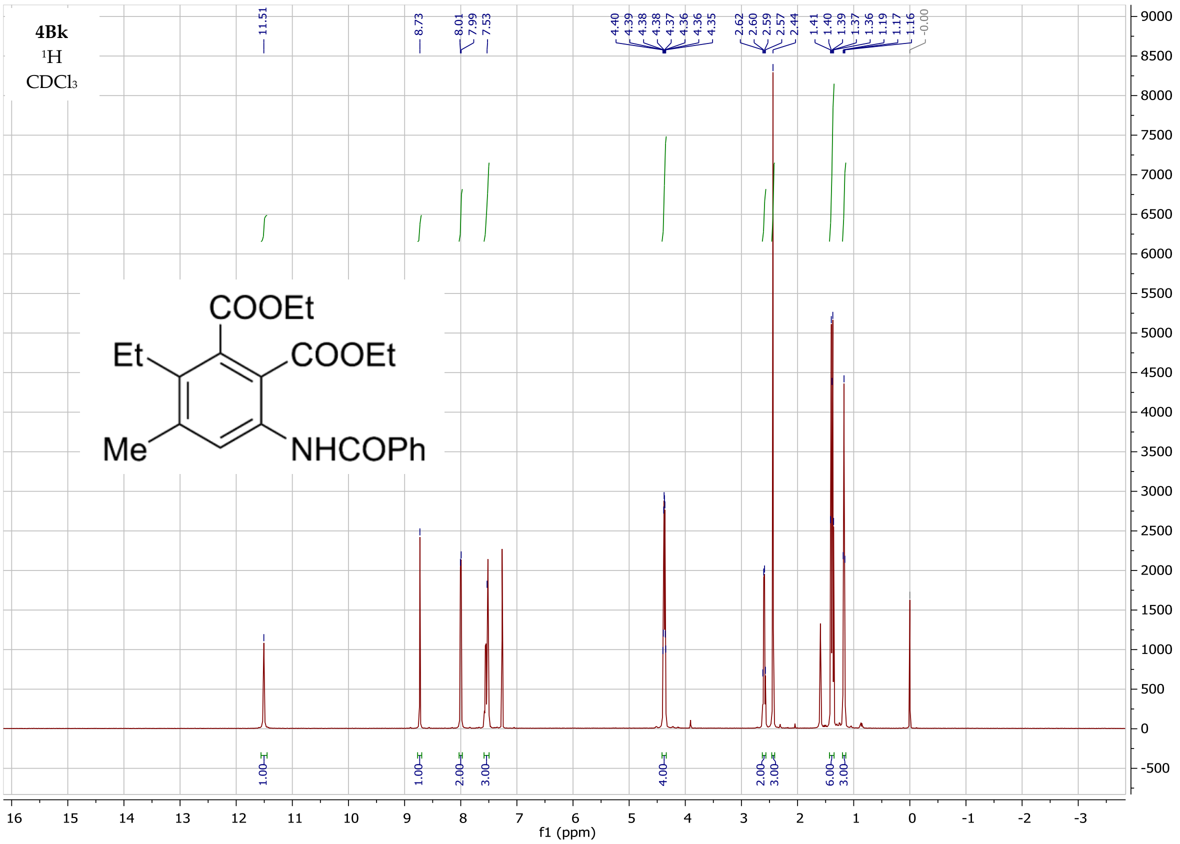
4Bk
1H
CDCl3
11.51
8.73
8.01
7.99
7.53
4.40
4.39
4.38
4.38
4.37
4.36
4.36
4.35
2.62
2.60
2.59
2.57
2.44
1.41
1.40
1.39
1.37
1.36
1.19
1.17
1.16
-0.00
COOEt
Et
COOEt
Me
NHCOPh
1.00
1.00
2.00
3.00
4.00
2.00
3.00
6.00
3.00
f1 (ppm)

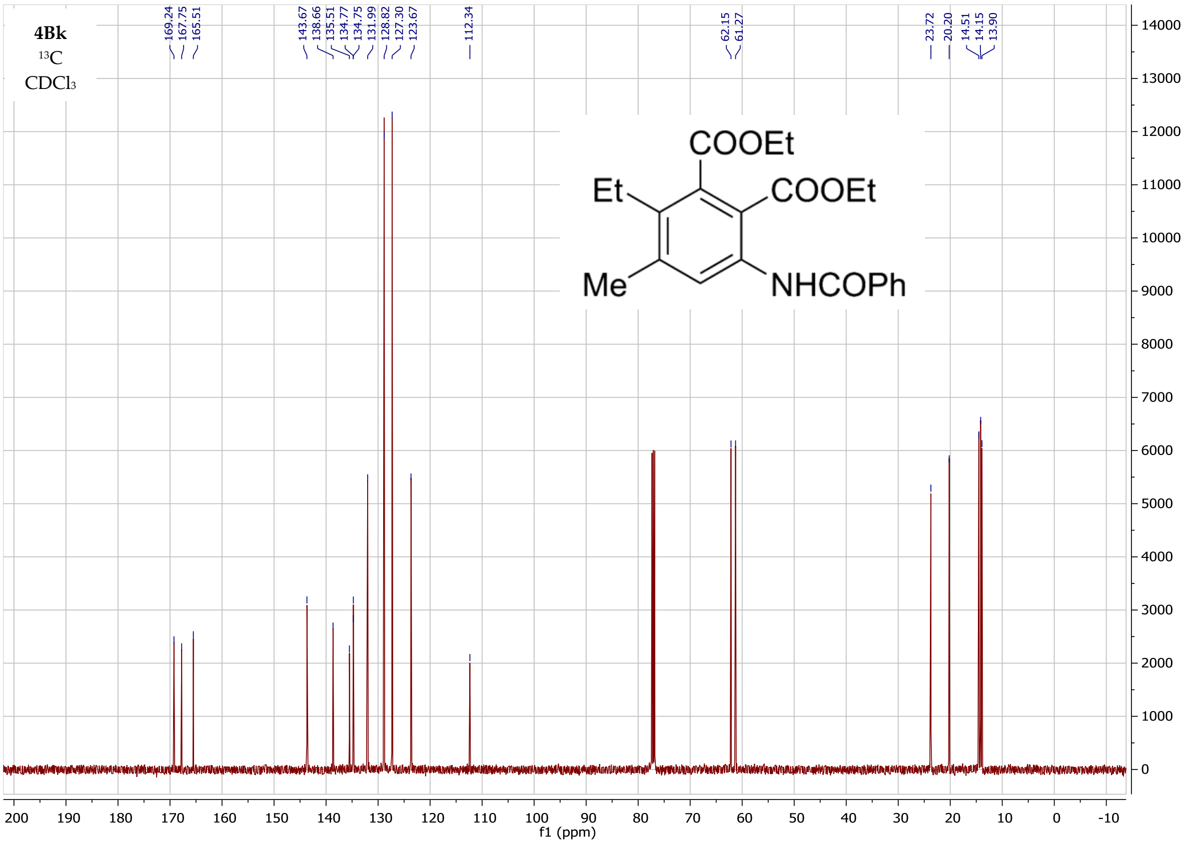
4Bk
13C
CDCl3
169.24
167.75
165.51
143.67
138.66
135.51
134.77
134.75
131.99
128.82
127.30
123.67
112.34
62.15
61.27
23.72
20.20
14.51
14.15
13.90
COOEt
Et
COOEt
Me
NHCOPh
f1 (ppm)

4Bl

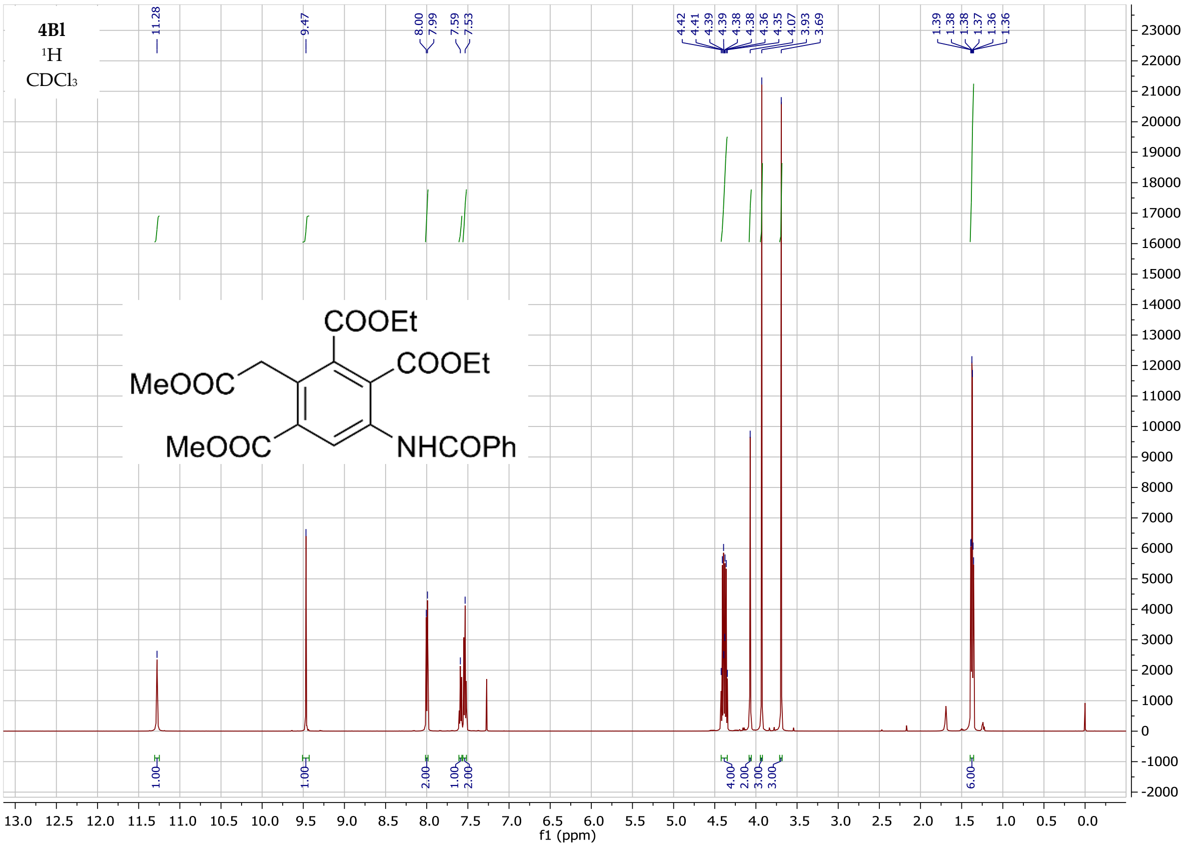
4Bl
^{1}H
CDCl$_3$
COOEt
COOEt
MeOOC
MeOOC
NHCOPh
11.28
9.47
8.00
7.99
7.59
7.53
4.42
4.41
4.39
4.39
4.38
4.38
4.36
4.35
4.07
3.93
3.69
1.39
1.38
1.38
1.37
1.36
1.36
1.00
1.00
2.00
1.00
2.00
4.00
2.00
3.00
3.00
6.00
f1 (ppm)

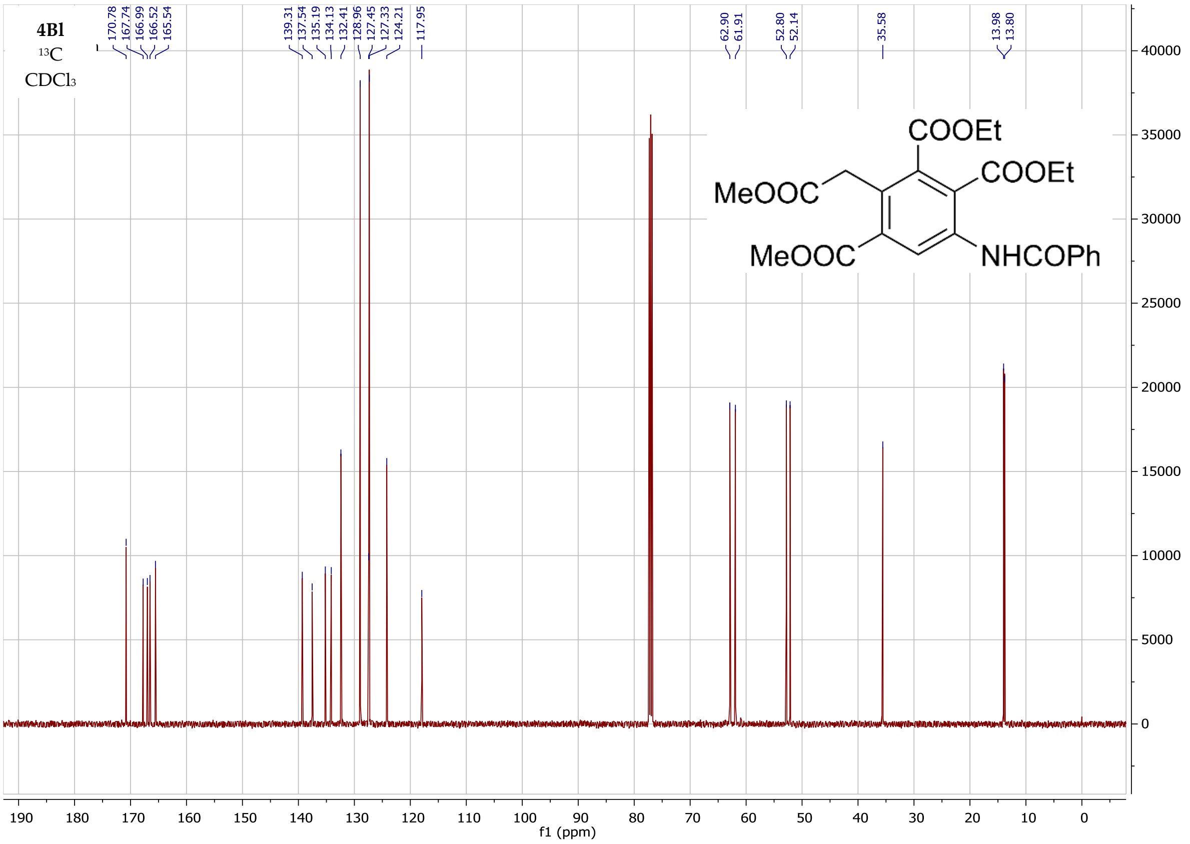
4Bl
^{13}C
CDCl$_3$
170.78
167.74
166.99
166.52
165.54
139.31
137.54
135.19
134.13
132.41
128.96
127.45
127.33
124.21
117.95
62.90
61.91
52.80
52.14
35.58
13.98
13.80
COOEt
COOEt
MeOOC
MeOOC
NHCOPh
f1 (ppm)

4Bm

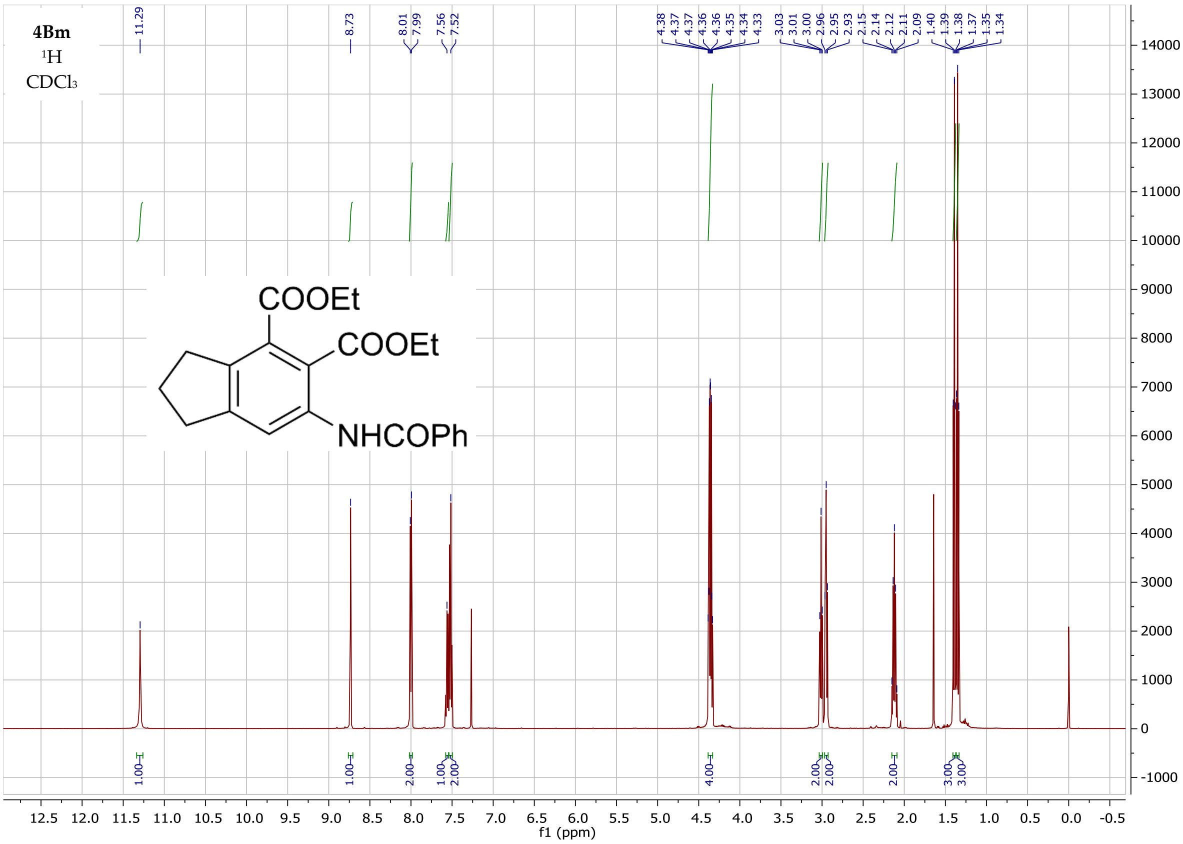

4Bm
^{1}H
CDCl$_3$
COOEt
COOEt
NHCOPh
11.29
8.73
8.01
7.99
7.56
7.52
4.38
4.37
4.37
4.36
4.36
4.35
4.34
4.33
3.03
3.01
3.00
2.96
2.95
2.93
2.15
2.14
2.12
2.11
2.09
1.40
1.39
1.38
1.37
1.35
1.34
1.00
1.00
2.00
1.00
2.00
4.00
2.00
2.00
2.00
3.00
3.00
f1 (ppm)

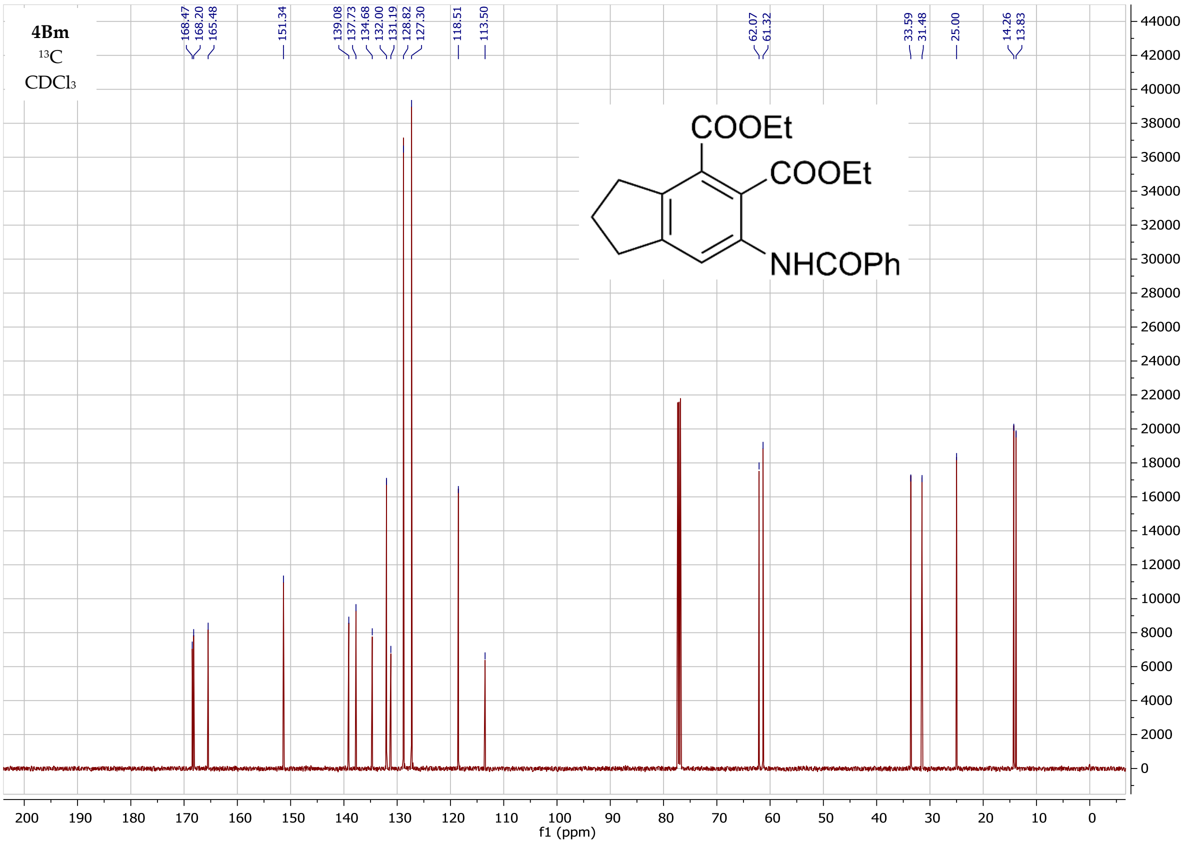

4Bm
^{13}C
CDCl$_3$
COOEt
COOEt
NHCOPh
168.47
168.20
165.48
151.34
139.08
137.73
134.68
132.00
131.19
128.82
127.30
118.51
113.50
62.07
61.32
33.59
31.48
25.00
14.26
13.83
f1 (ppm)

4Bn

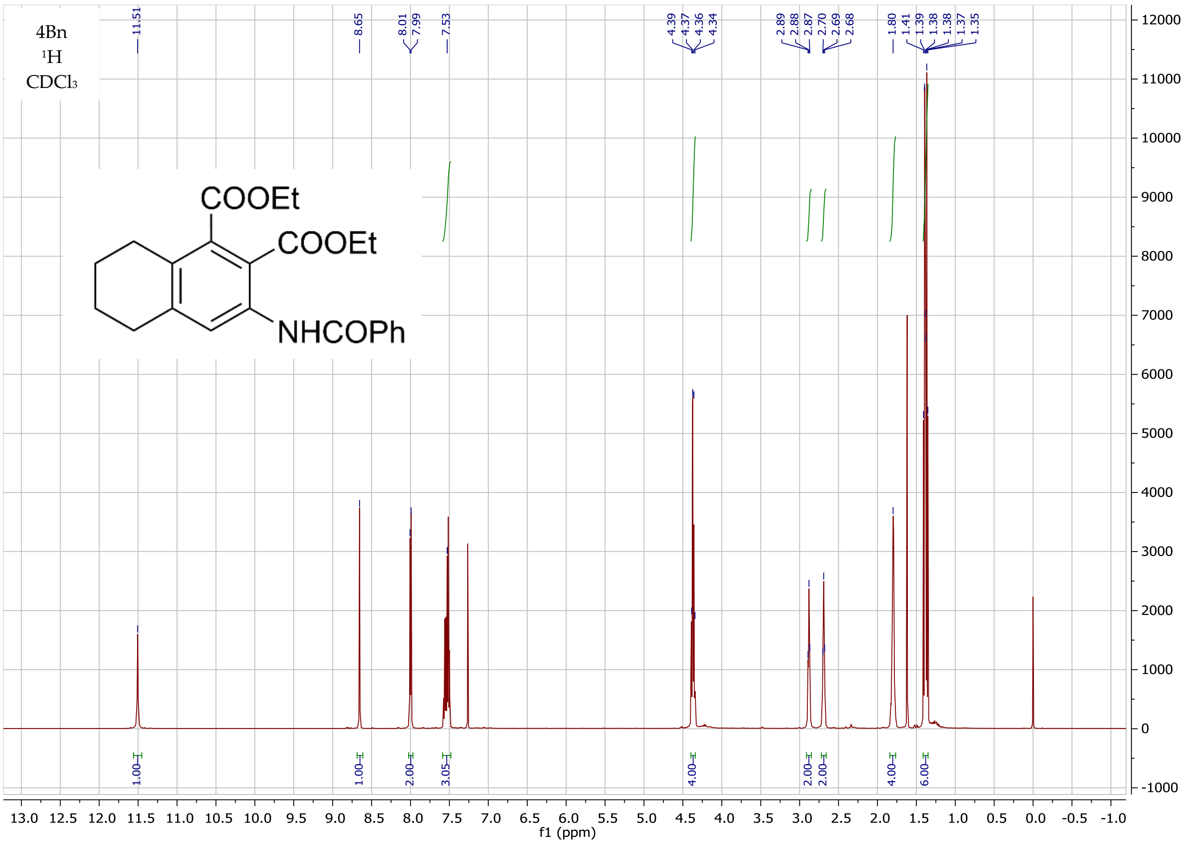

4Bn
¹H
CDCl₃
COOEt
COOEt
NHCOPh
11.51
8.65
8.01
7.99
7.53
4.39
4.37
4.36
4.34
2.89
2.88
2.87
2.70
2.69
2.68
1.80
1.41
1.39
1.38
1.38
1.37
1.35
1.00
1.00
2.00
3.05
4.00
2.00
2.00
4.00
6.00
f1 (ppm)

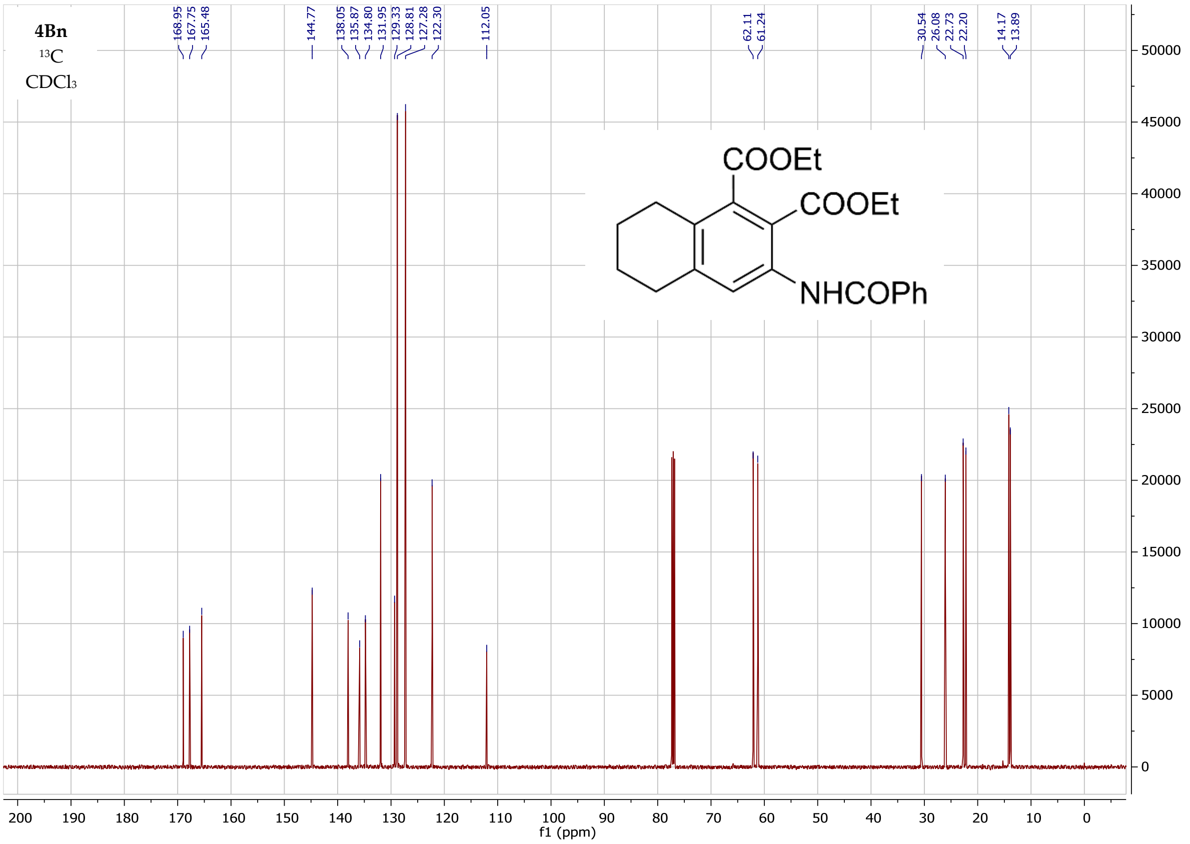

4Bn
¹³C
CDCl₃
168.95
167.75
165.48
144.77
138.05
135.87
134.80
131.95
129.33
128.81
127.28
122.30
112.05
62.11
61.24
30.54
26.08
22.73
22.20
14.17
13.89
COOEt
COOEt
NHCOPh
f1 (ppm)

4Bo

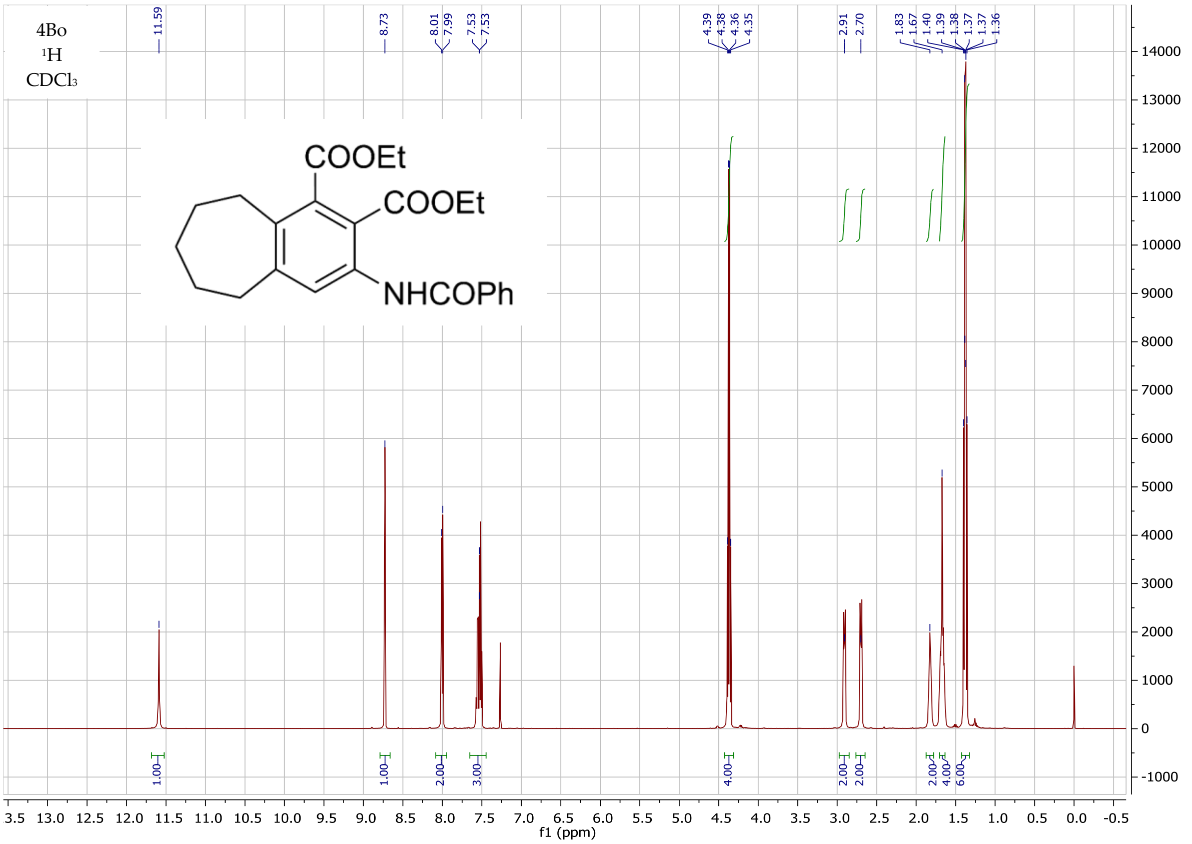
4Bo
1H
CDCl3
11.59
8.73
8.01
7.99
7.53
7.53
4.39
4.38
4.36
4.35
2.91
2.70
1.83
1.67
1.40
1.39
1.38
1.37
1.37
1.36
COOEt
COOEt
NHCOPh
1.00
1.00
2.00
3.00
4.00
2.00
2.00
2.00
4.00
6.00
f1 (ppm)

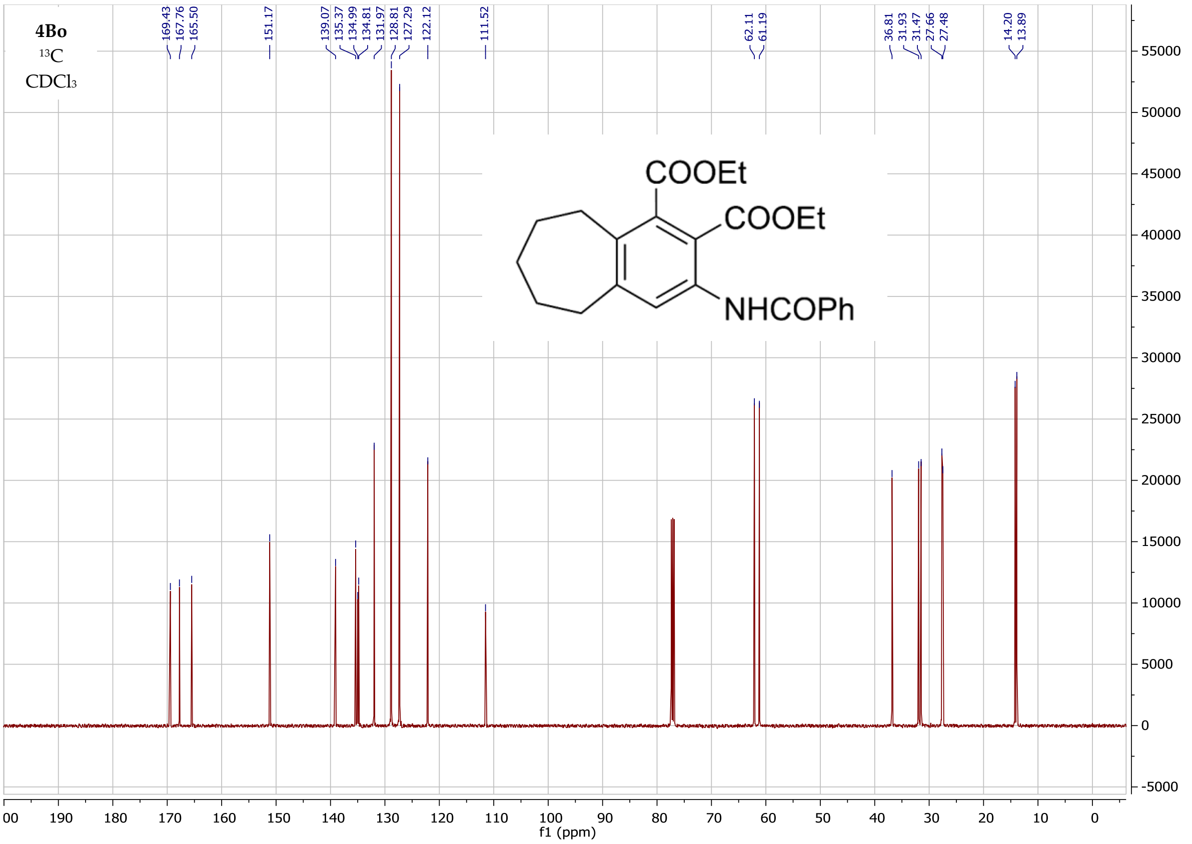
4Bo
13C
CDCl3
169.43
167.76
165.50
151.17
139.07
135.37
134.99
134.81
131.97
128.81
127.29
122.12
111.52
62.11
61.19
36.81
31.93
31.47
27.66
27.48
14.20
13.89
COOEt
COOEt
NHCOPh
f1 (ppm)

4Ca

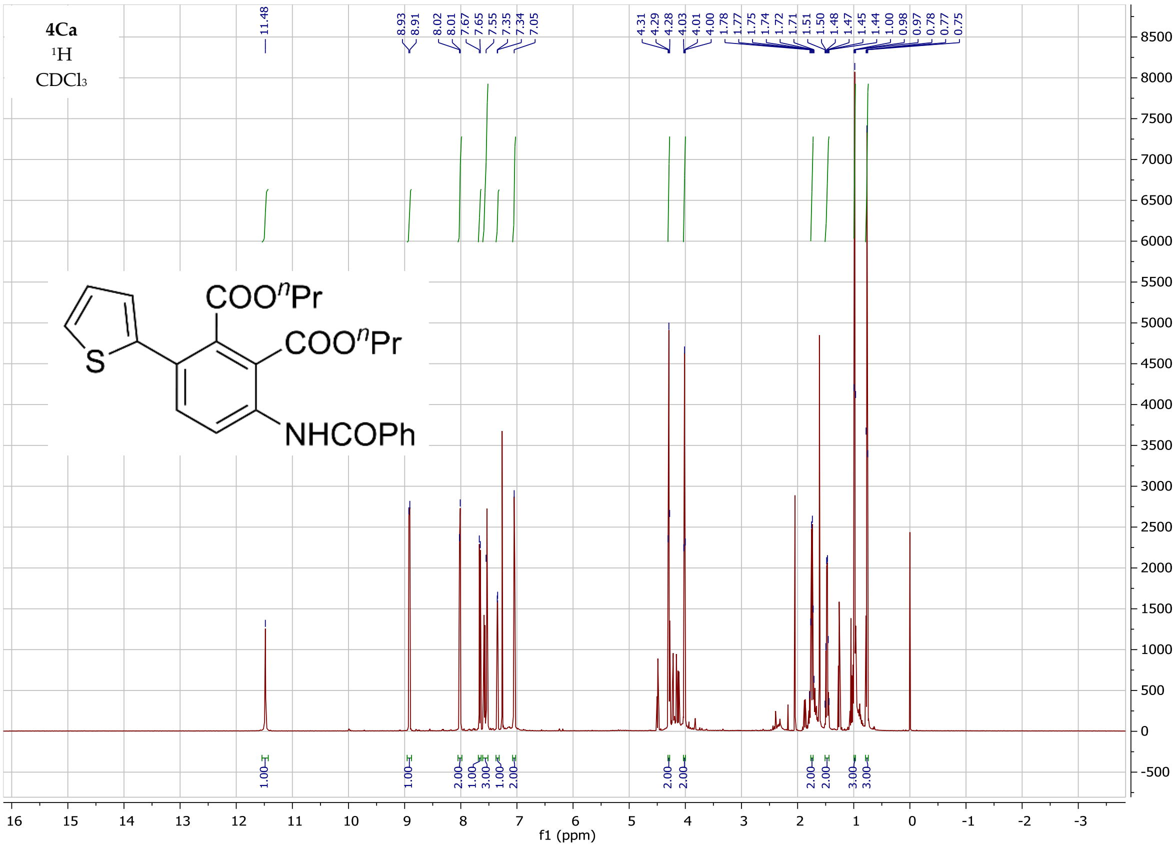
4Ca
1H
CDCl3
11.48
8.93
8.91
8.02
8.01
7.67
7.65
7.55
7.35
7.34
7.05
4.31
4.29
4.28
4.03
4.01
4.00
1.78
1.77
1.75
1.74
1.72
1.71
1.51
1.50
1.48
1.47
1.45
1.44
1.00
0.98
0.97
0.78
0.77
0.75
COOnPr
COOnPr
S
NHCOPh
1.00
1.00
2.00
1.00
3.00
1.00
2.00
2.00
2.00
2.00
2.00
3.00
3.00
f1 (ppm)

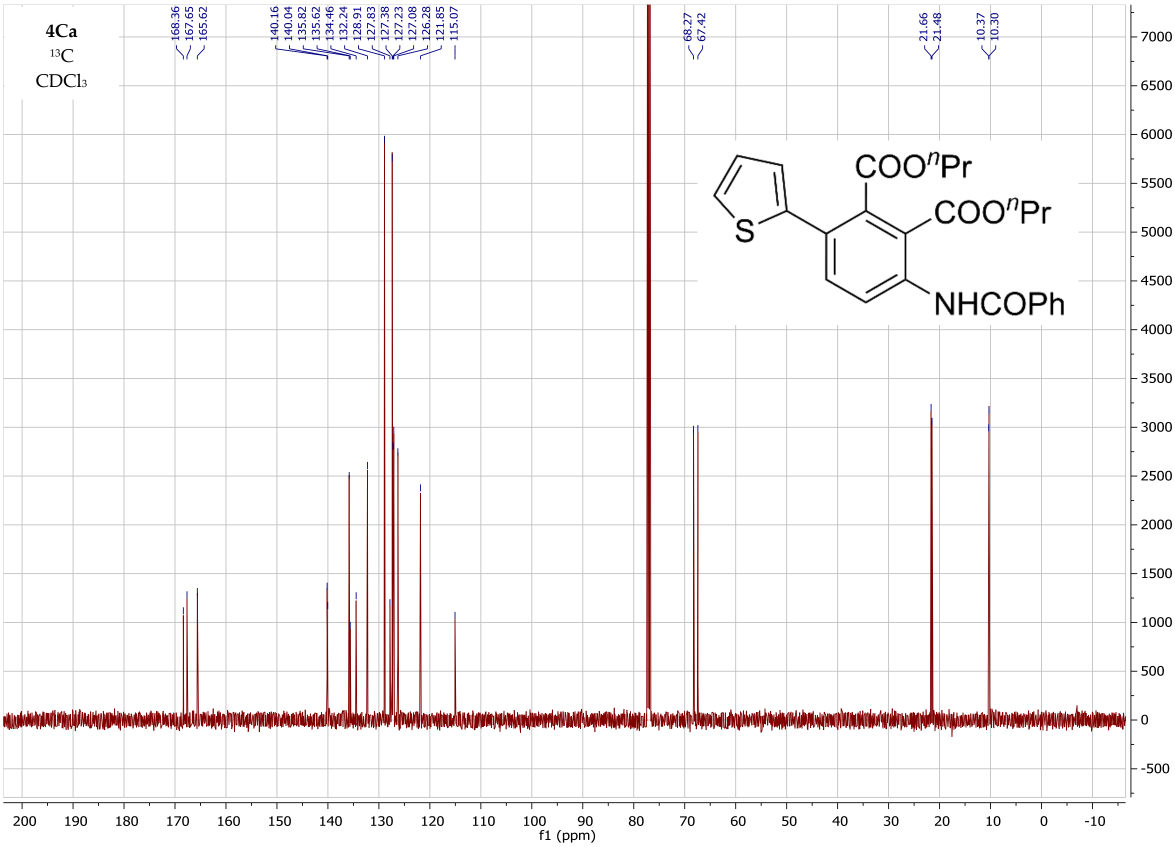
4Ca
13C
CDCl3
168.36
167.65
165.62
140.16
140.04
135.82
135.62
134.46
132.24
128.91
127.83
127.38
127.23
127.08
126.28
121.85
115.07
68.27
67.42
21.66
21.48
10.37
10.30
COOnPr
COOnPr
S
NHCOPh
f1 (ppm)

4Cb

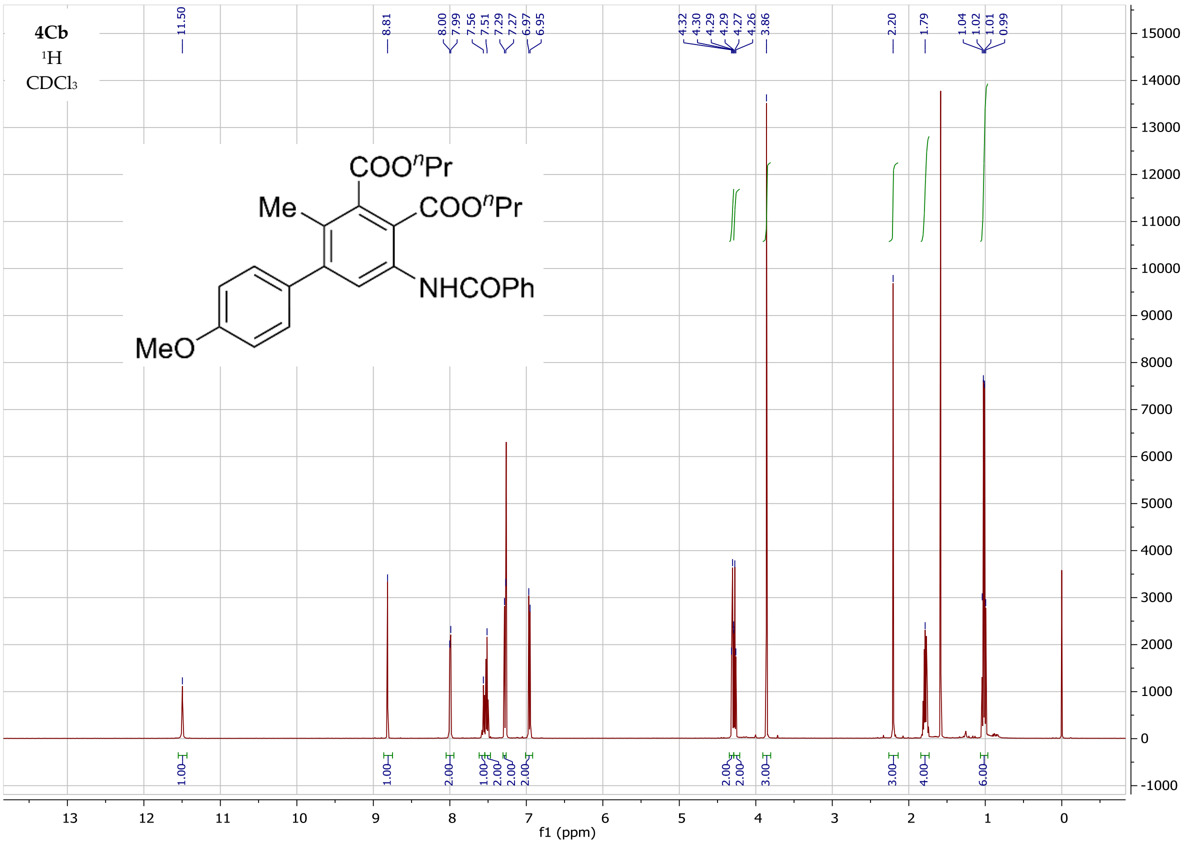
4Cb
1H
CDCl3
COOnPr
Me
COOnPr
NHCOPh
MeO
11.50
8.81
8.00
7.99
7.56
7.51
7.29
7.27
6.97
6.95
4.32
4.30
4.29
4.29
4.27
4.26
3.86
2.20
1.79
1.04
1.02
1.01
0.99
1.00
1.00
2.00
1.00
2.00
2.00
2.00
2.00
2.00
3.00
3.00
4.00
6.00
f1 (ppm)

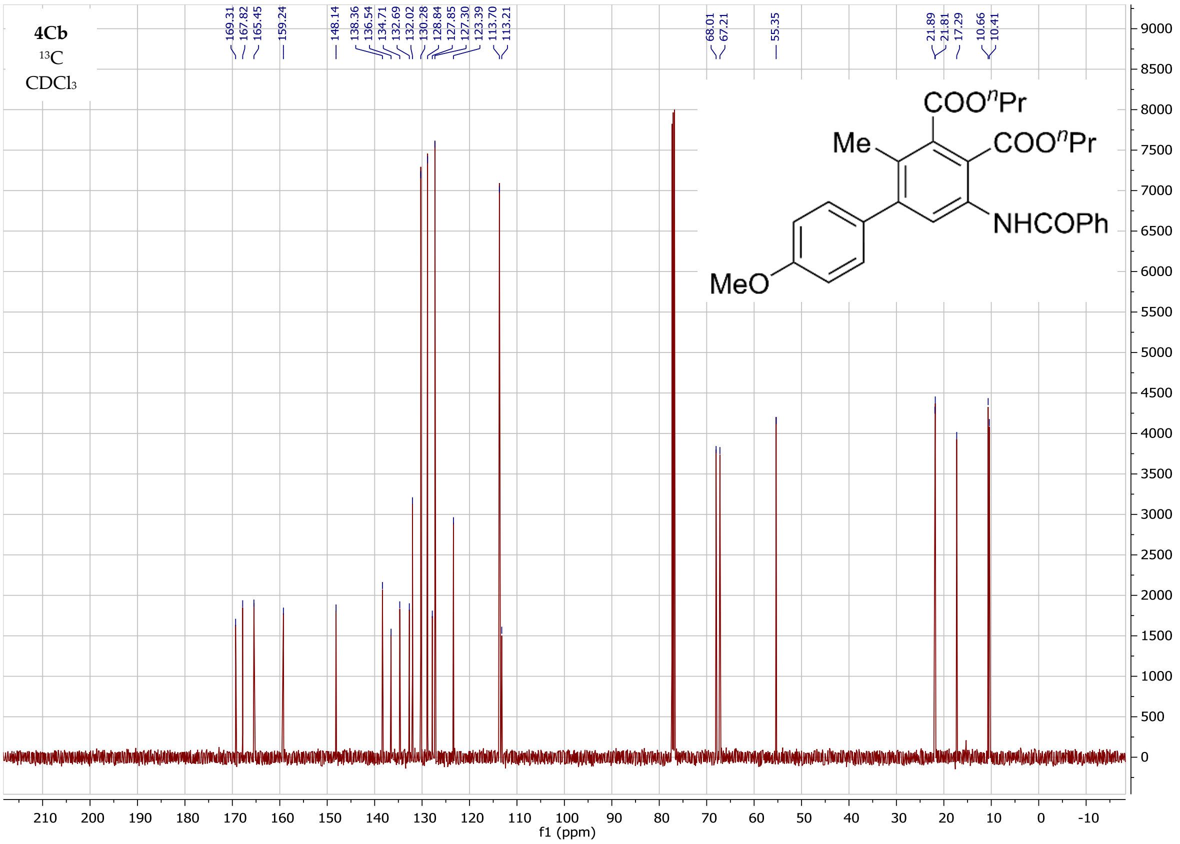
4Cb
13C
CDCl3
169.31
167.82
165.45
159.24
148.14
138.36
136.54
134.71
132.69
132.02
130.28
128.84
127.85
127.30
123.39
113.70
113.21
68.01
67.21
55.35
21.89
21.81
17.29
10.66
10.41
COOnPr
Me
COOnPr
NHCOPh
MeO
f1 (ppm)

4Cc

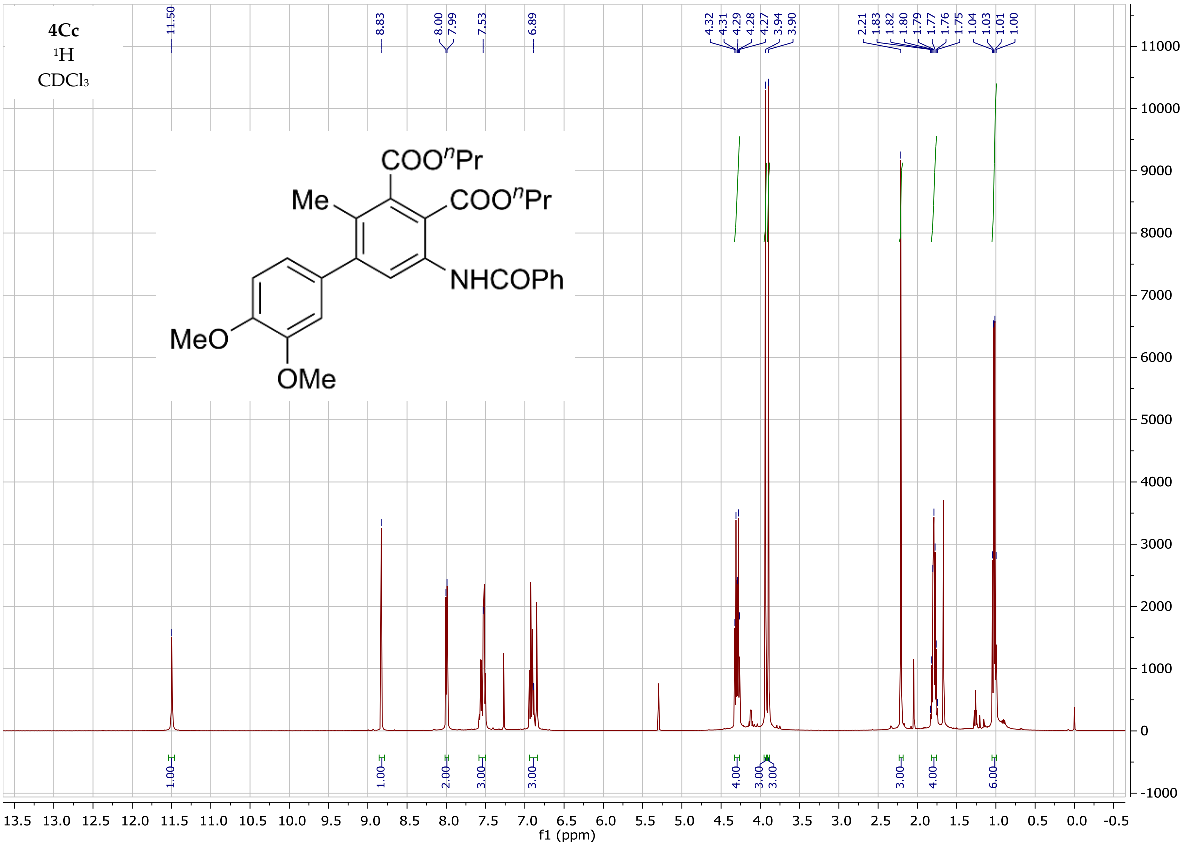

4Cc
1H
CDCl3
COO^{n}Pr
COO^{n}Pr
Me
NHCOPh
MeO
OMe
f1 (ppm)

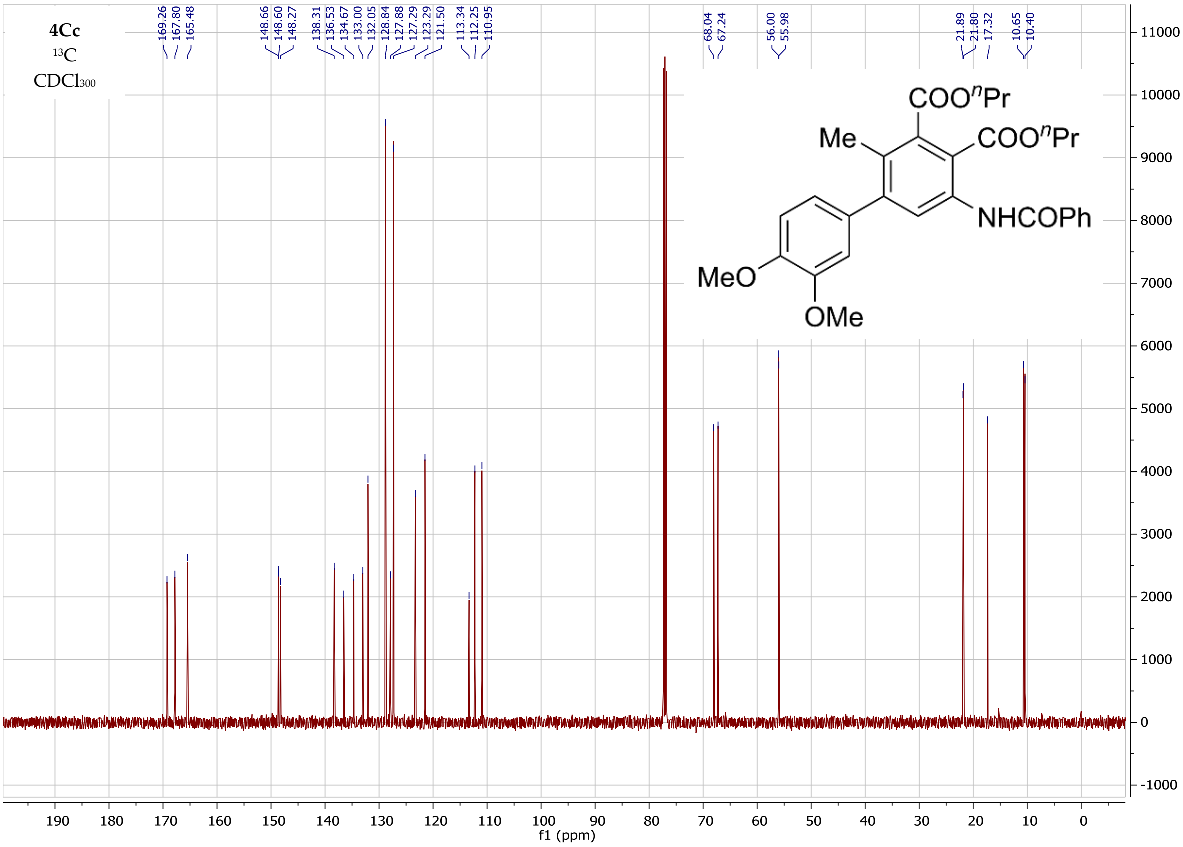

4Cc
13C
CDCl3
COO^{n}Pr
COO^{n}Pr
Me
NHCOPh
MeO
OMe
f1 (ppm)

4Cd

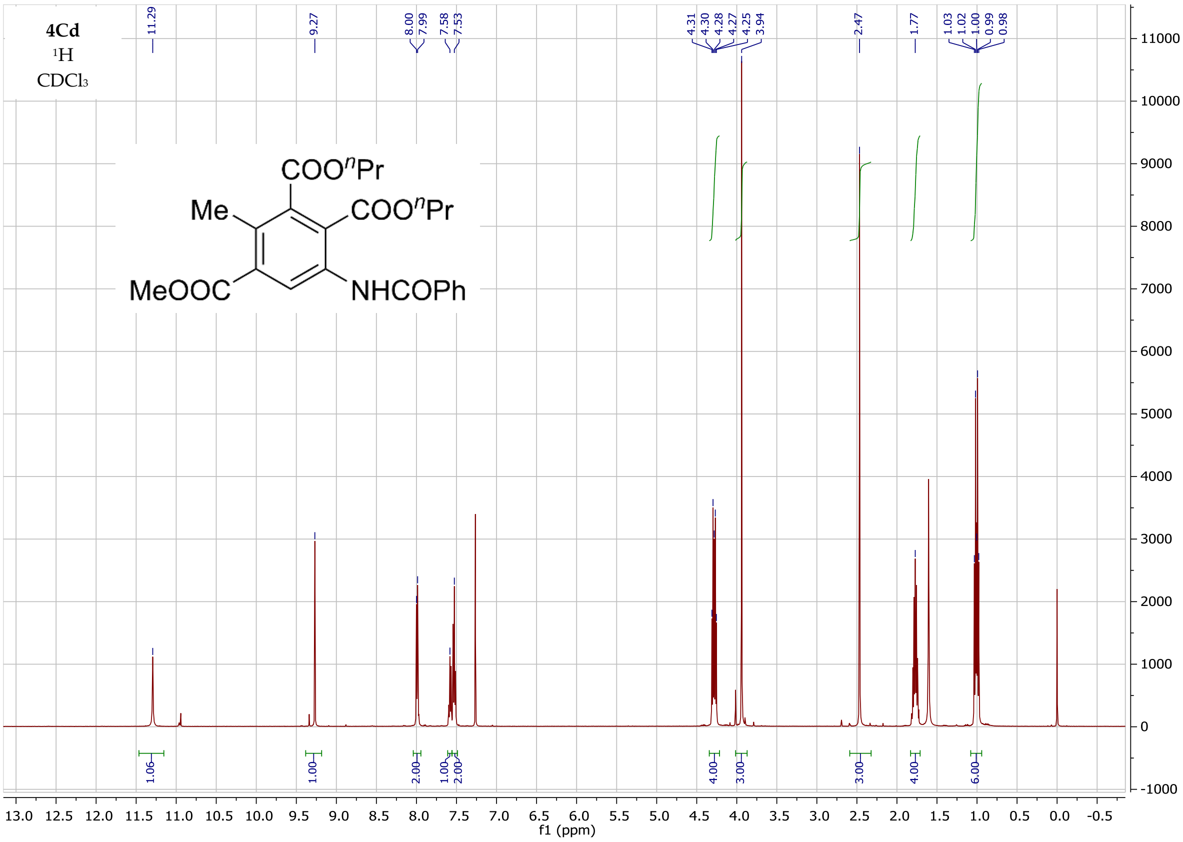

4Cd
1H
CDCl3
COOnPr
Me
COOnPr
MeOOC
NHCOPh
11.29
9.27
8.00
7.99
7.58
7.53
4.31
4.30
4.28
4.27
4.25
3.94
2.47
1.77
1.03
1.02
1.00
0.99
0.98
1.06
1.00
2.00
1.00
2.00
4.00
3.00
3.00
4.00
6.00
f1 (ppm)

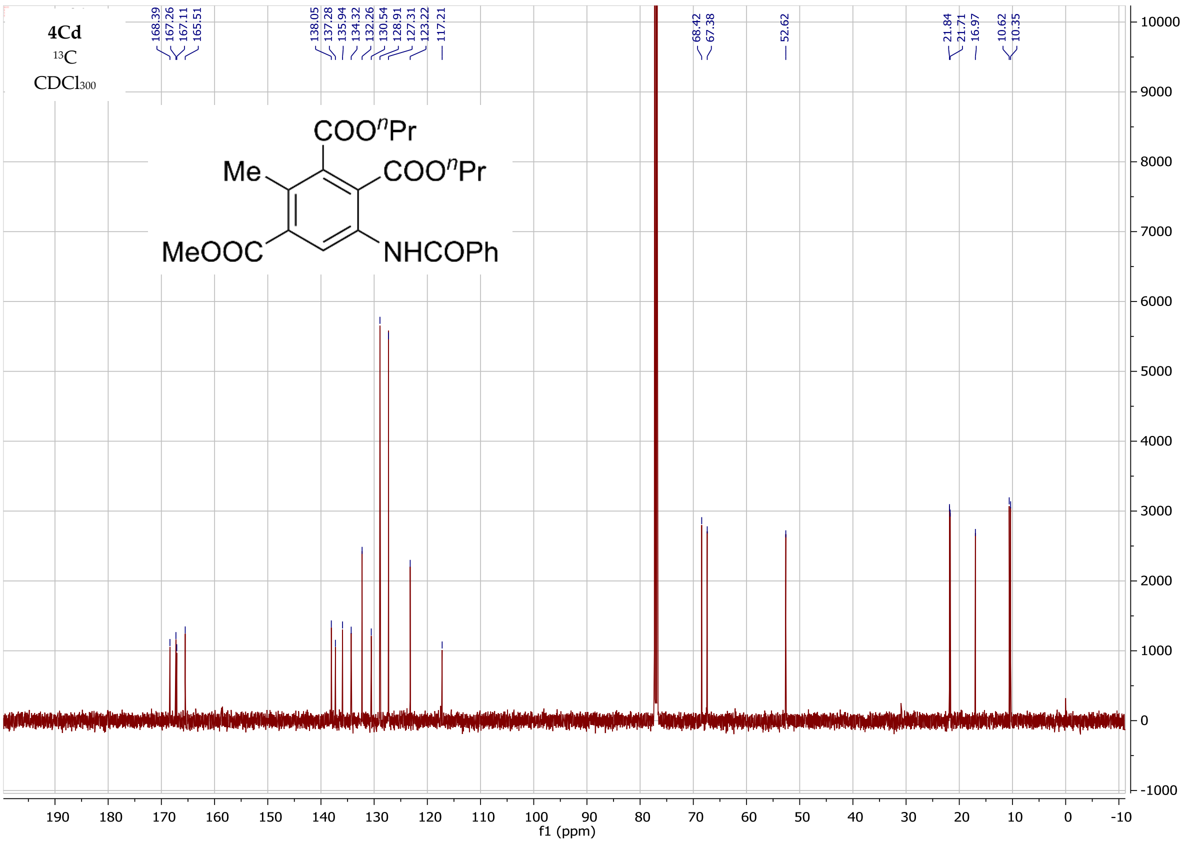

4Cd
13C
CDCl3
COOnPr
Me
COOnPr
MeOOC
NHCOPh
168.39
167.26
167.11
165.51
138.05
137.28
135.94
134.32
132.26
130.54
128.91
127.31
123.22
117.21
68.42
67.38
52.62
21.84
21.71
16.97
10.62
10.35
f1 (ppm)

4Ce

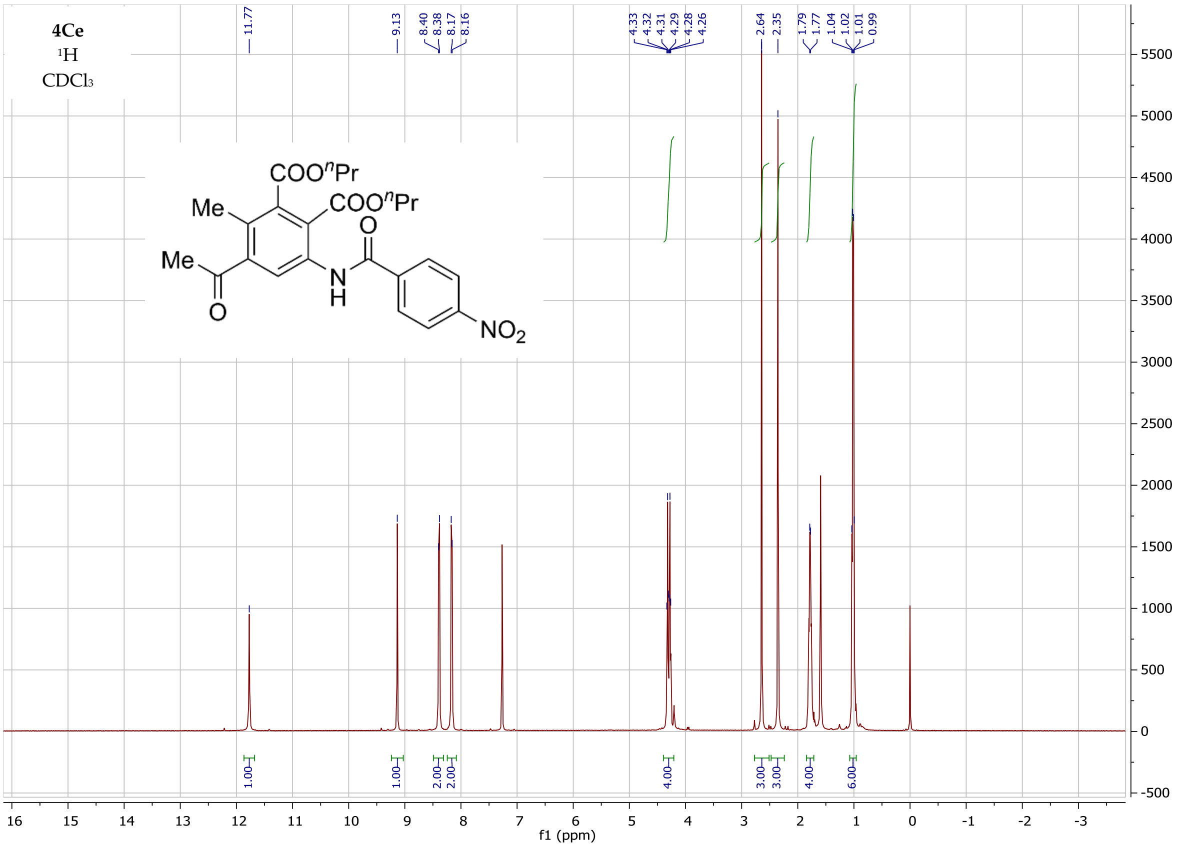

4Ce
1H
CDCl3
11.77
9.13
8.40
8.38
8.17
8.16
4.33
4.32
4.31
4.29
4.28
4.26
2.64
2.35
1.79
1.77
1.04
1.02
1.01
0.99
COOnPr
COOnPr
Me
Me
O
N
H
O
NO2
1.00
1.00
2.00
2.00
4.00
3.00
3.00
4.00
6.00
f1 (ppm)

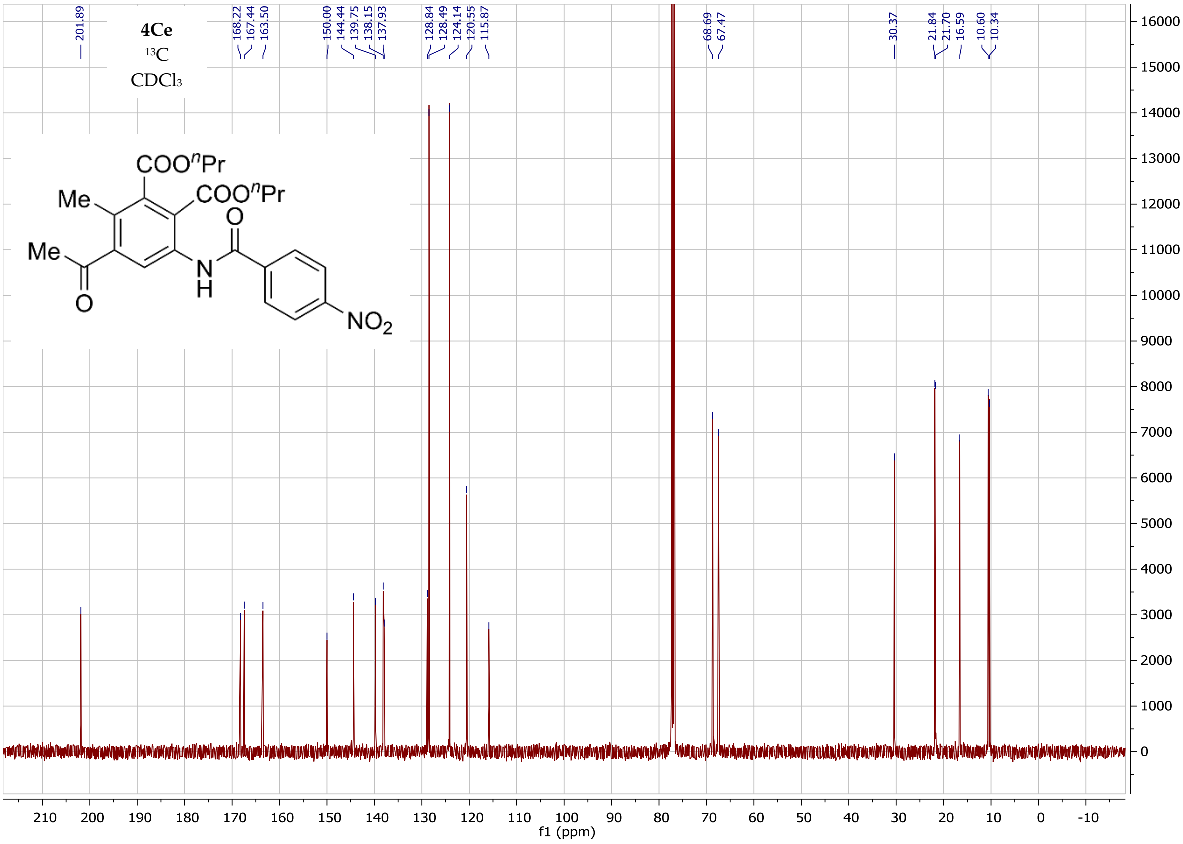

4Ce
13C
CDCl3
201.89
168.22
167.44
163.50
150.00
144.44
139.75
138.15
137.93
128.84
128.49
124.14
120.55
115.87
68.69
67.47
30.37
21.84
21.70
16.59
10.60
10.34
COOnPr
COOnPr
Me
Me
O
N
H
O
NO2
f1 (ppm)

4Cf

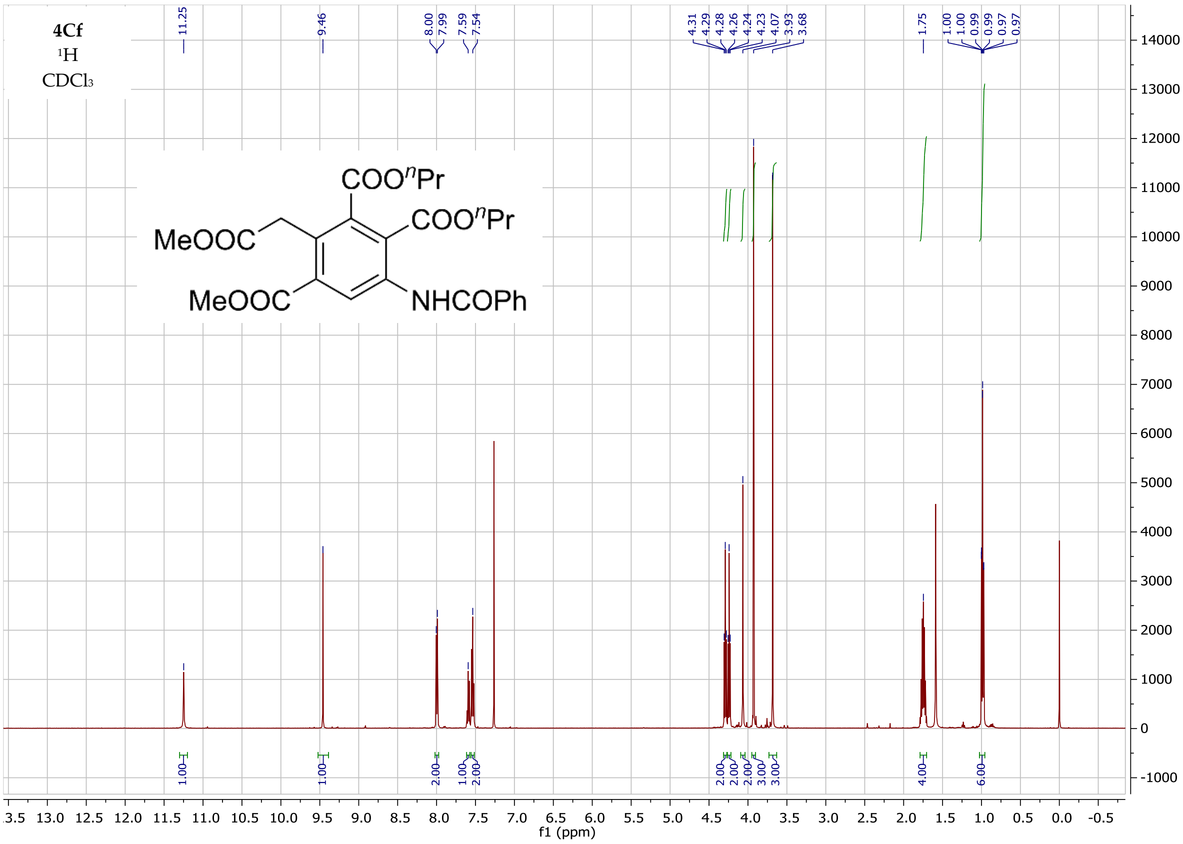
4Cf
1H
CDCl3
COOnPr
COOnPr
MeOOC
MeOOC
NHCOPh
11.25
9.46
8.00
7.99
7.59
7.54
4.31
4.29
4.28
4.26
4.24
4.23
4.07
3.93
3.68
1.75
1.00
1.00
0.99
0.99
0.97
0.97
1.00
1.00
2.00
1.00
2.00
2.00
2.00
2.00
3.00
3.00
4.00
6.00
f1 (ppm)

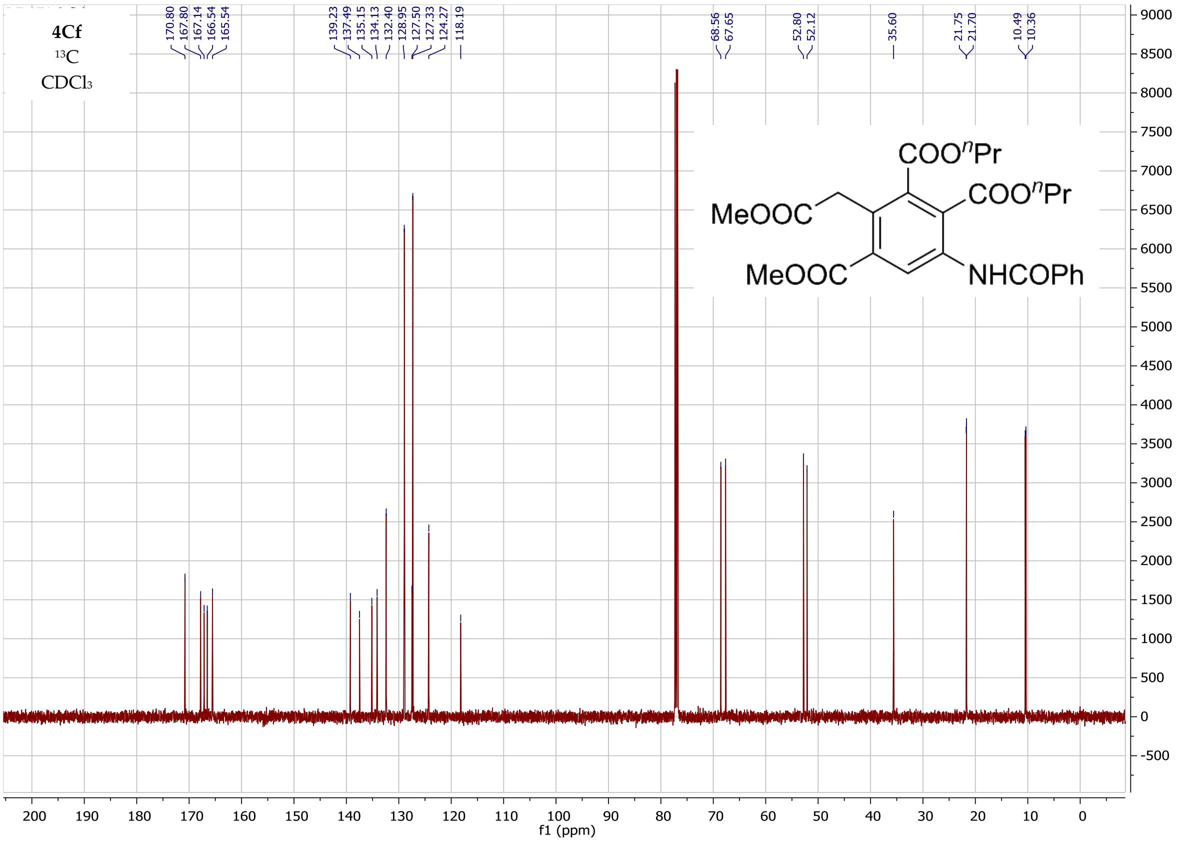
4Cf
13C
CDCl3
COOnPr
COOnPr
MeOOC
MeOOC
NHCOPh
170.80
167.80
167.14
166.54
165.54
139.23
137.49
135.15
134.13
132.40
128.95
127.50
127.33
124.27
118.19
68.56
67.65
52.80
52.12
35.60
21.75
21.70
10.49
10.36
f1 (ppm)

4Cg

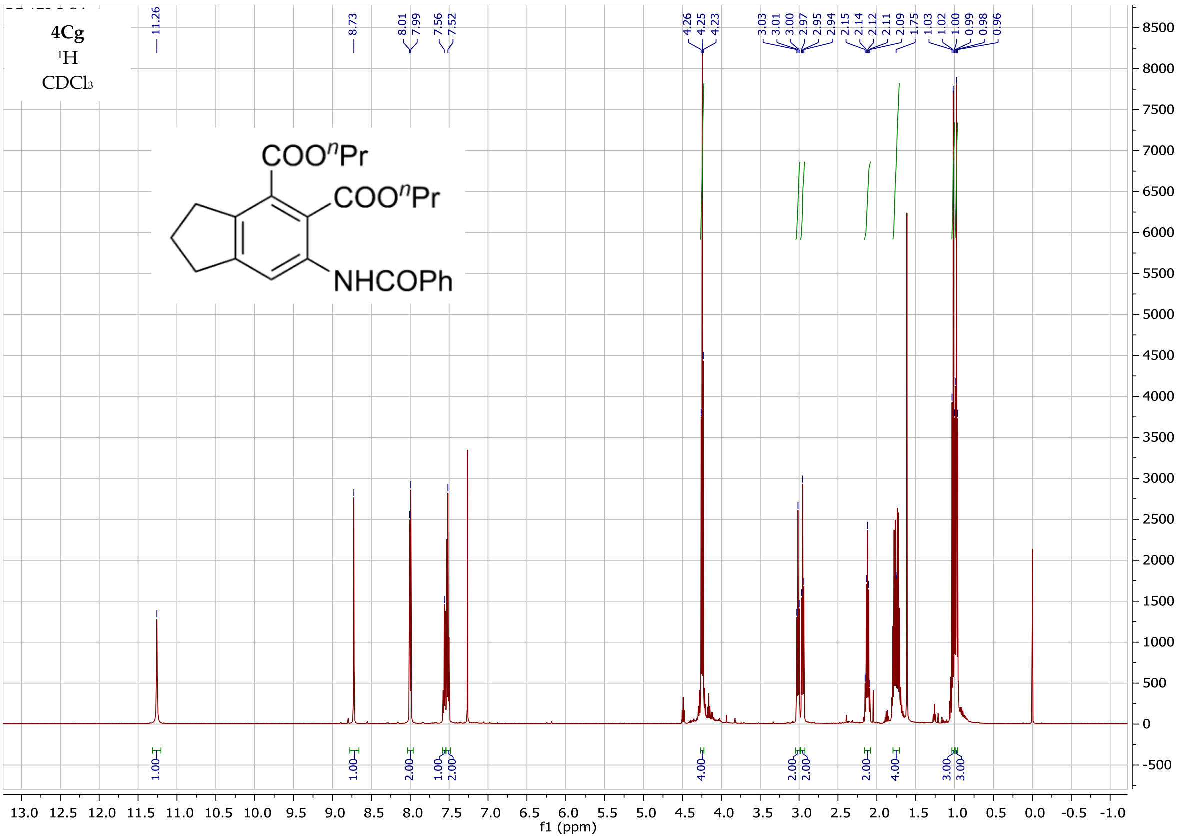
4Cg
1H
CDCl3
COOnPr
COOnPr
NHCOPh
11.26
8.73
8.01
7.99
7.56
7.52
4.26
4.25
4.23
3.03
3.01
3.00
2.97
2.95
2.94
2.15
2.14
2.12
2.11
2.09
1.75
1.03
1.02
1.00
0.99
0.98
0.96
1.00
1.00
2.00
1.00
2.00
4.00
2.00
2.00
2.00
4.00
3.00
3.00
f1 (ppm)

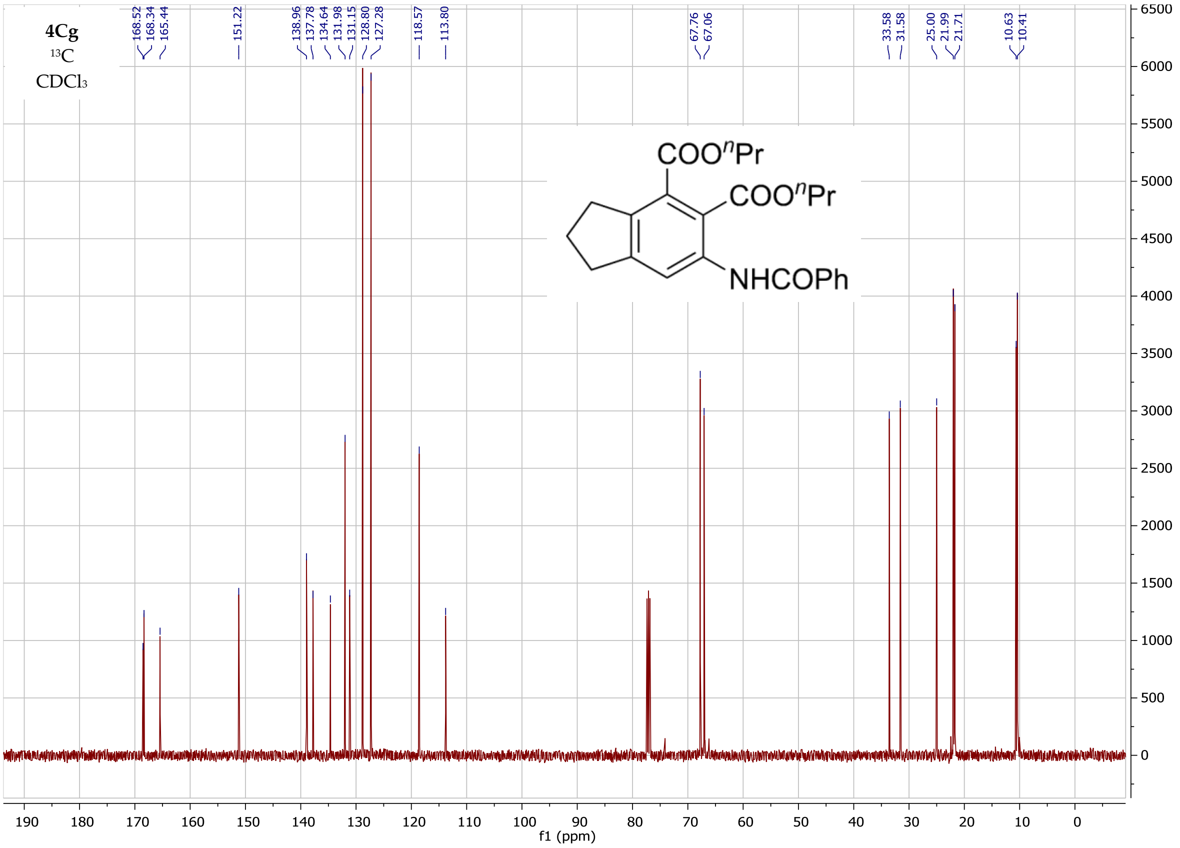
4Cg
13C
CDCl3
COOnPr
COOnPr
NHCOPh
168.52
168.34
165.44
151.22
138.96
137.78
134.64
131.98
131.15
128.80
127.28
118.57
113.80
67.76
67.06
33.58
31.58
25.00
21.99
21.71
10.63
10.41
f1 (ppm)

4Da

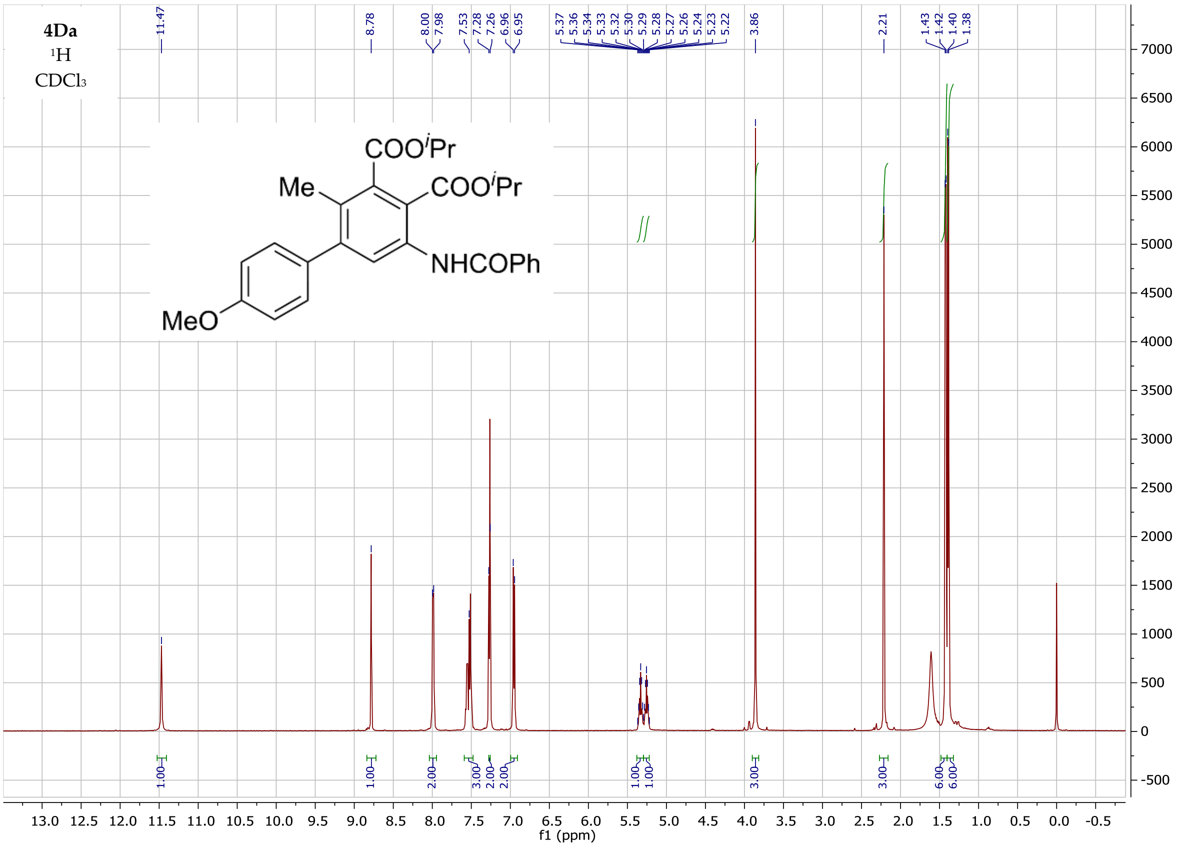

4Da
¹H
CDCl₃
COOⁱPr
COOⁱPr
Me
NHCOPh
MeO
f1 (ppm)

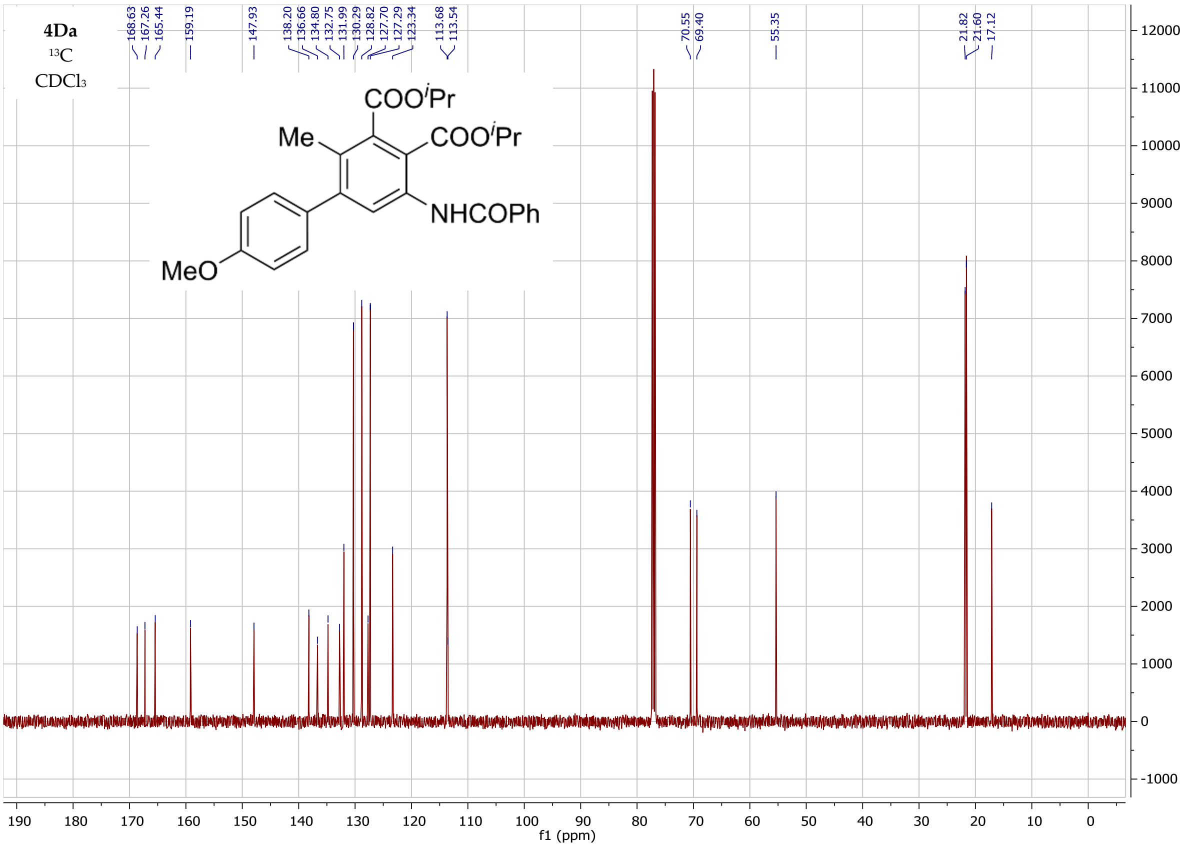

4Da
¹³C
CDCl₃
COOⁱPr
COOⁱPr
Me
NHCOPh
MeO
f1 (ppm)

4Db

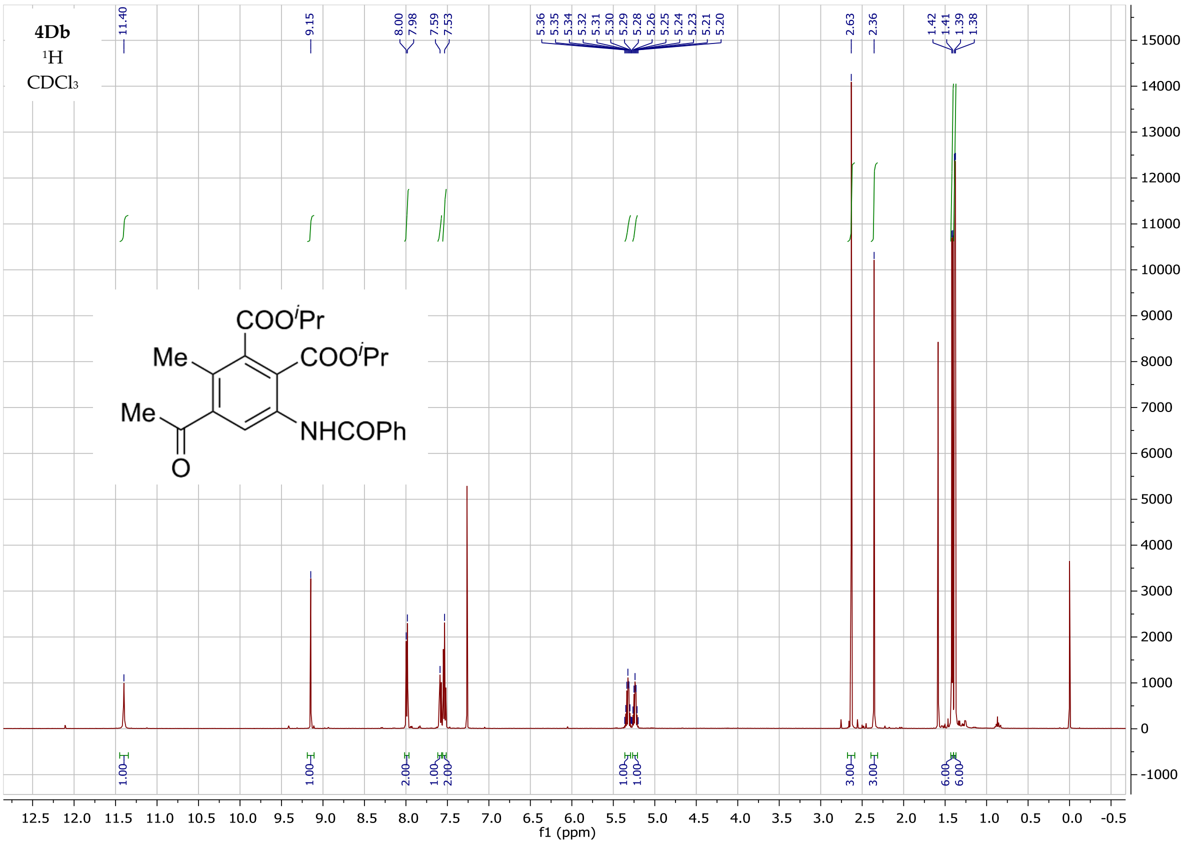
4Db
1H
CDCl3
COOiPr
Me
COOiPr
Me
NHCOPh
O
11.40
9.15
8.00
7.98
7.59
7.53
5.36
5.35
5.34
5.32
5.31
5.30
5.29
5.28
5.26
5.25
5.24
5.23
5.21
5.20
2.63
2.36
1.42
1.41
1.39
1.38
1.00
1.00
2.00
1.00
2.00
1.00
1.00
3.00
3.00
6.00
6.00
f1 (ppm)

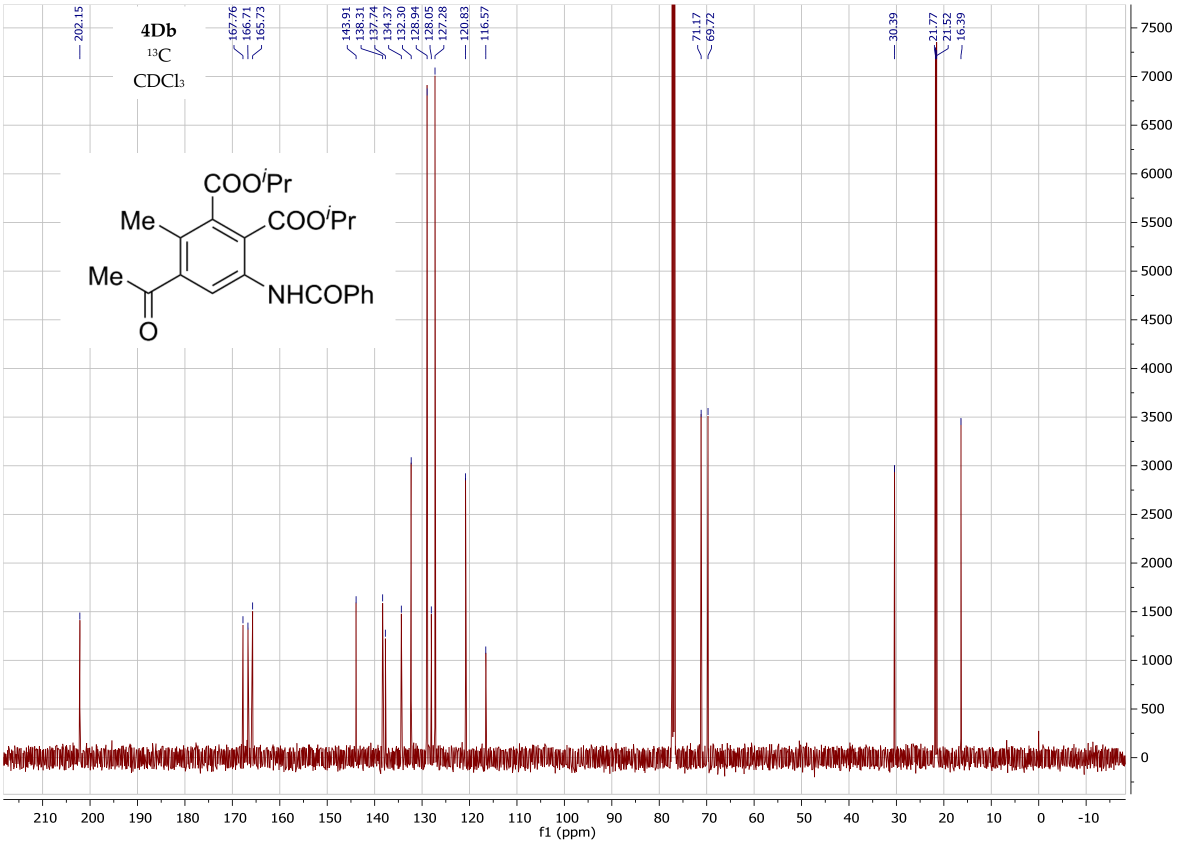
4Db
13C
CDCl3
COOiPr
Me
COOiPr
Me
NHCOPh
O
202.15
167.76
166.71
165.73
143.91
138.31
137.74
134.37
132.30
128.94
128.05
127.28
120.83
116.57
71.17
69.72
30.39
21.77
21.52
16.39
f1 (ppm)

4Ea

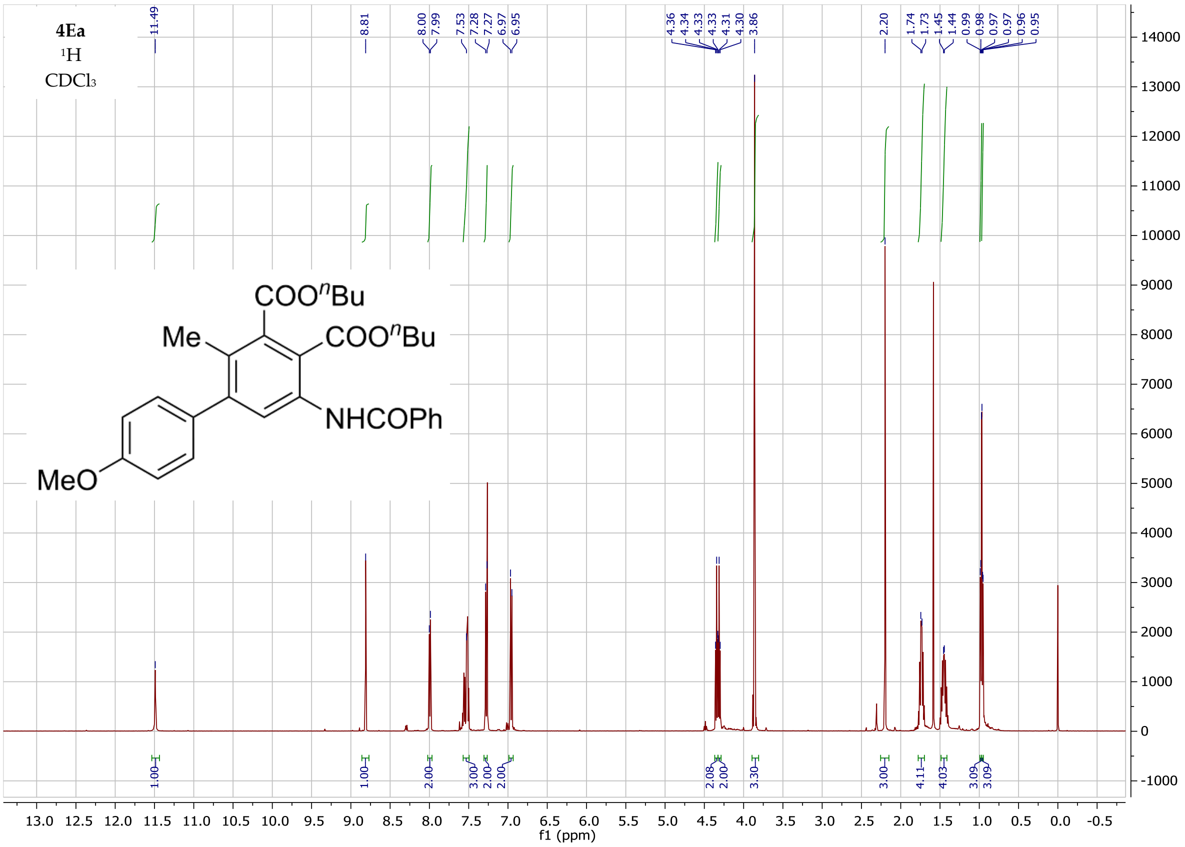
4Ea
1H
CDCl3
COOnBu
Me
COOnBu
NHCOPh
MeO
f1 (ppm)

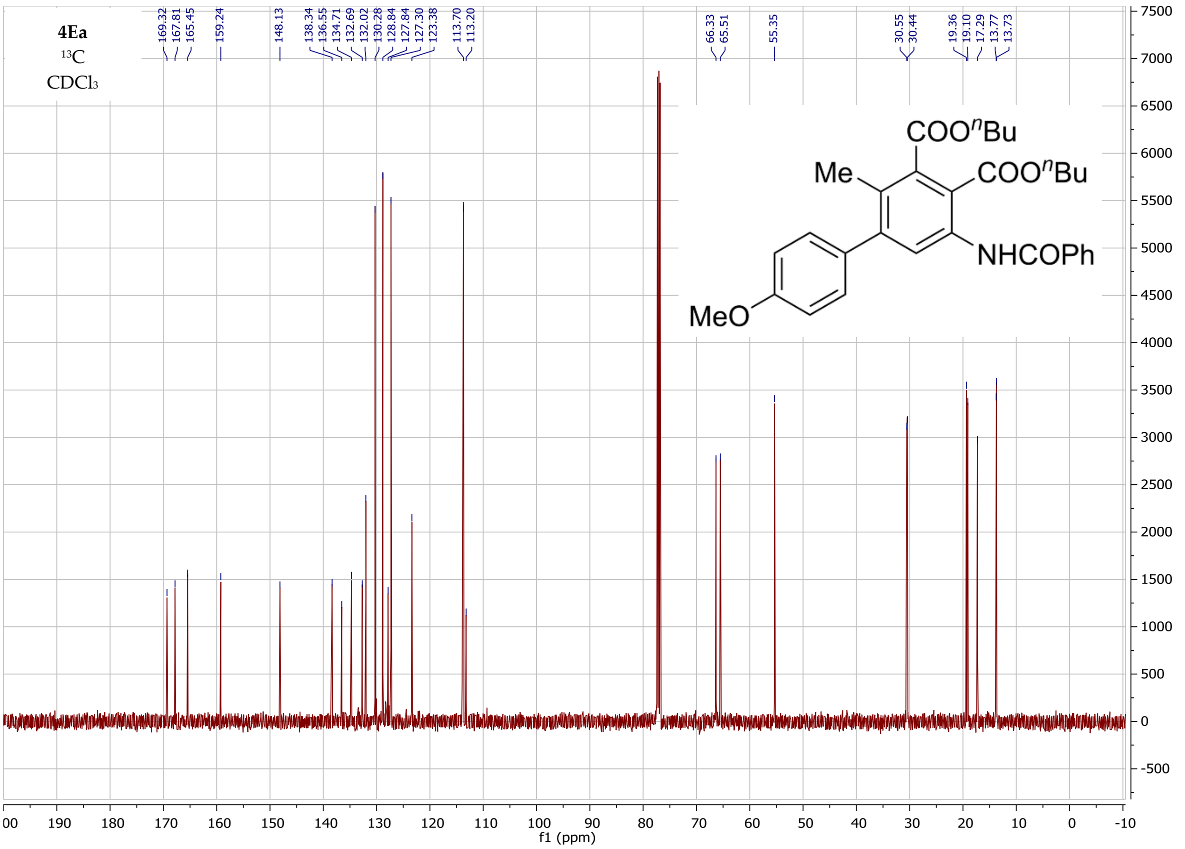
4Ea
13C
CDCl3
COOnBu
Me
COOnBu
NHCOPh
MeO
f1 (ppm)

4Fa

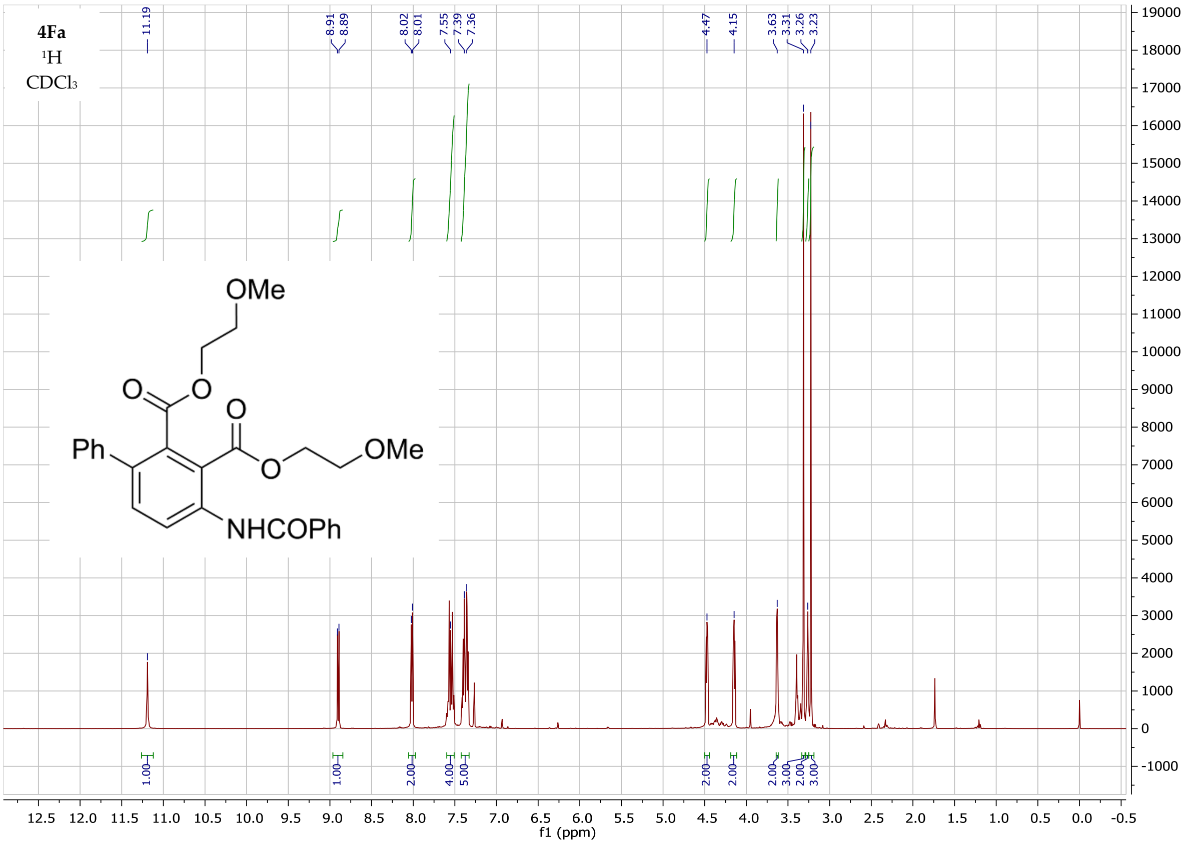

4Fa
^{1}H
$CDCl_3$
11.19
8.91
8.89
8.02
8.01
7.55
7.39
7.36
4.47
4.15
3.63
3.31
3.26
3.23
1.00
1.00
2.00
4.00
5.00
2.00
2.00
2.00
3.00
2.00
3.00
OMe
O
O
O
O
Ph
OMe
NHCOPh
f1 (ppm)

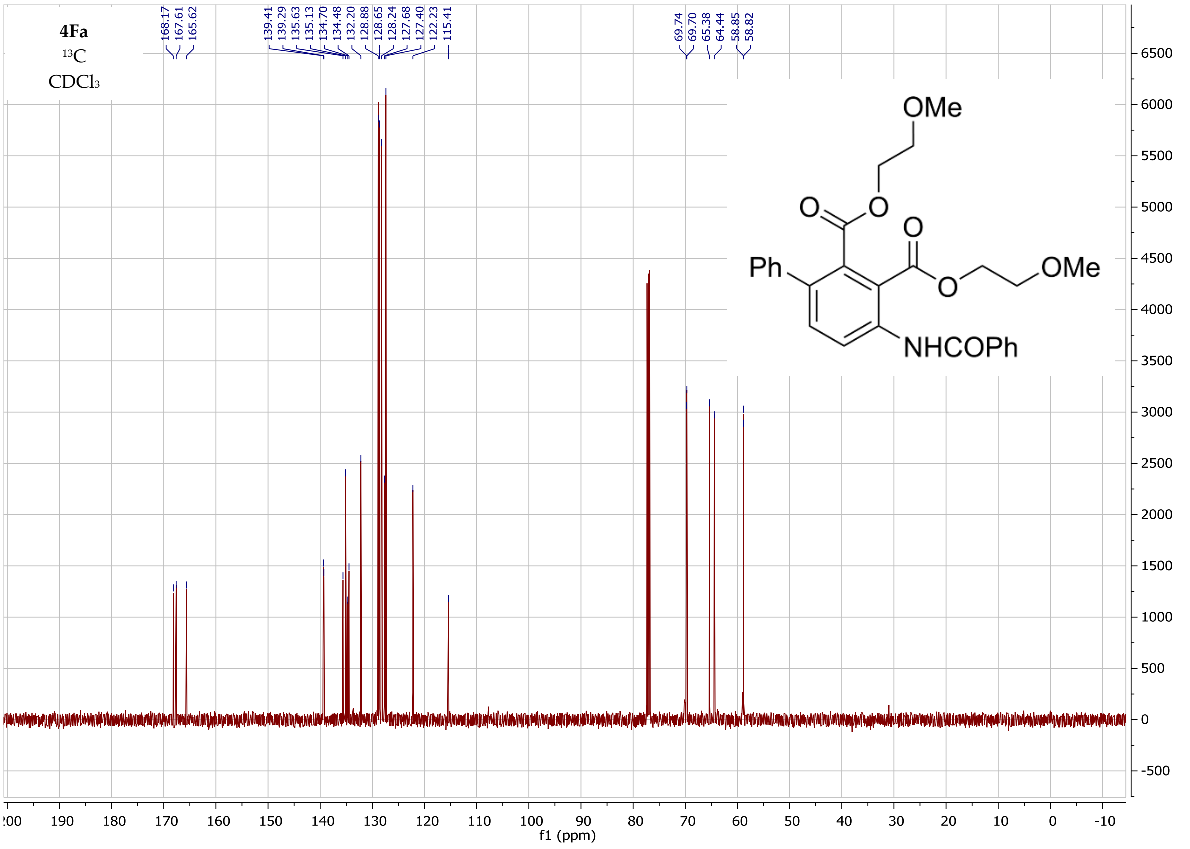

4Fa
^{13}C
$CDCl_3$
168.17
167.61
165.62
139.41
139.29
135.63
135.13
134.70
134.48
132.20
128.88
128.65
128.24
127.68
127.40
122.23
115.41
69.74
69.70
65.38
64.44
58.85
58.82
OMe
O
O
O
O
Ph
OMe
NHCOPh
f1 (ppm)

4Fb

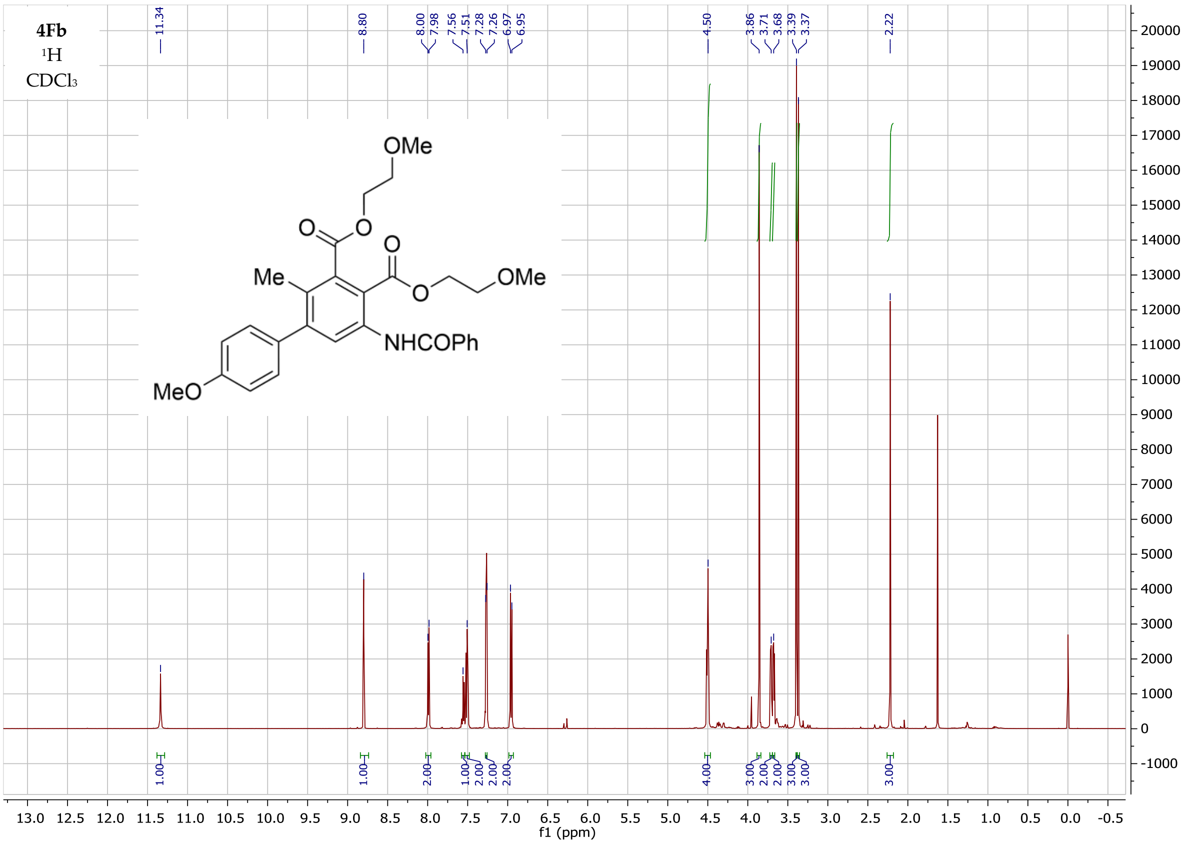
4Fb
1H
CDCl3
11.34
8.80
8.00
7.98
7.56
7.51
7.28
7.26
6.97
6.95
4.50
3.86
3.71
3.68
3.39
3.37
2.22
OMe
O
O
Me
O
O
OMe
NHCOPh
MeO
1.00
1.00
2.00
1.00
2.00
2.00
2.00
4.00
3.00
2.00
2.00
3.00
3.00
3.00
f1 (ppm)

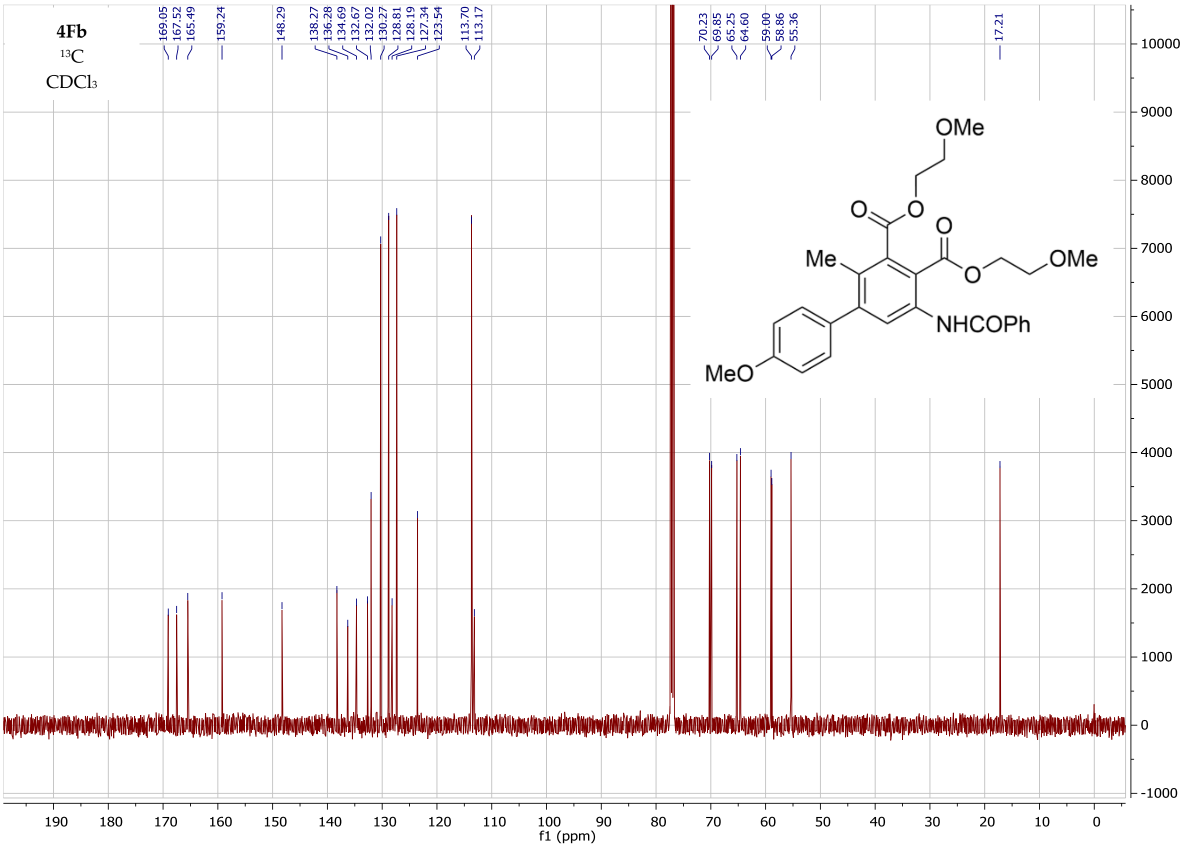
4Fb
13C
CDCl3
169.05
167.52
165.49
159.24
148.29
138.27
136.28
134.69
132.67
132.02
130.27
128.81
128.19
127.34
123.54
113.70
113.17
70.23
69.85
65.25
64.60
59.00
58.86
55.36
17.21
OMe
O
O
Me
O
O
OMe
NHCOPh
MeO
f1 (ppm)

4Fc

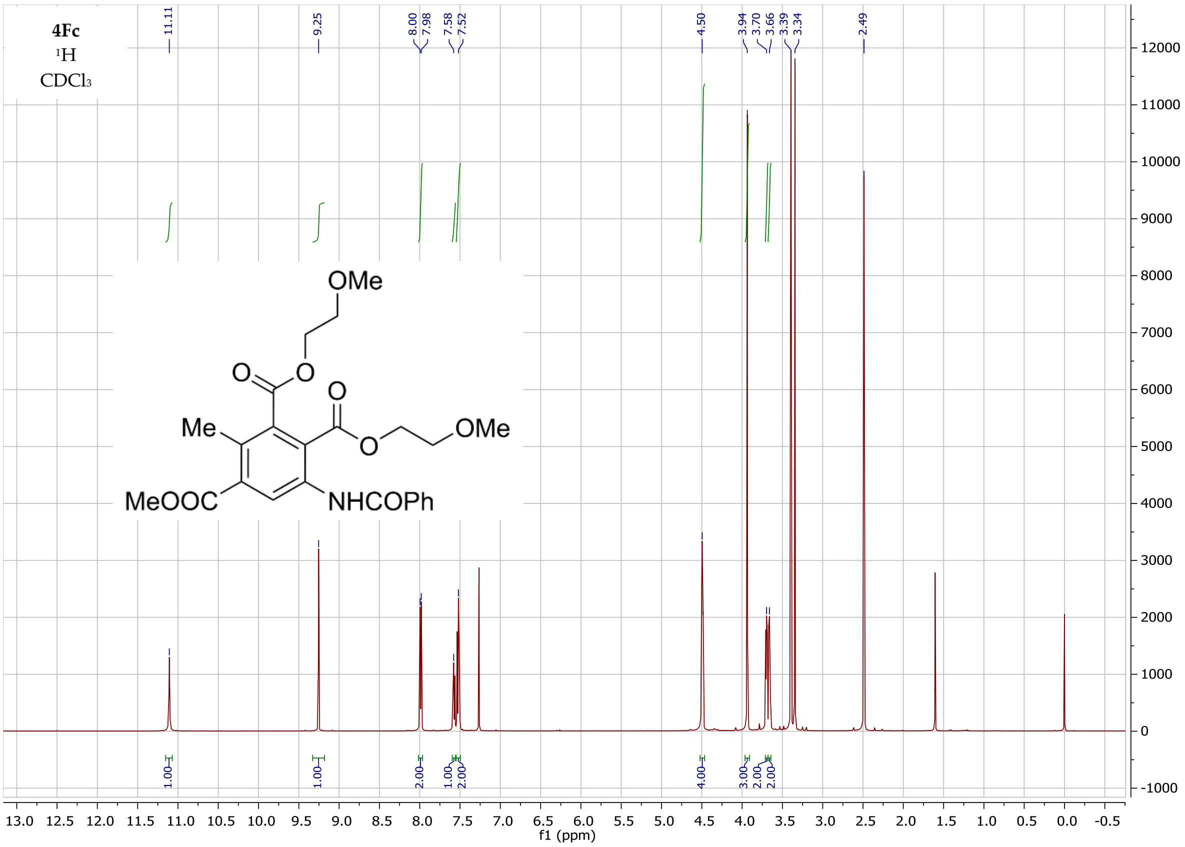
4Fc
¹H
CDCl3
OMe
O
O
O
Me
O
OMe
MeOOC
NHCOPh
f1 (ppm)

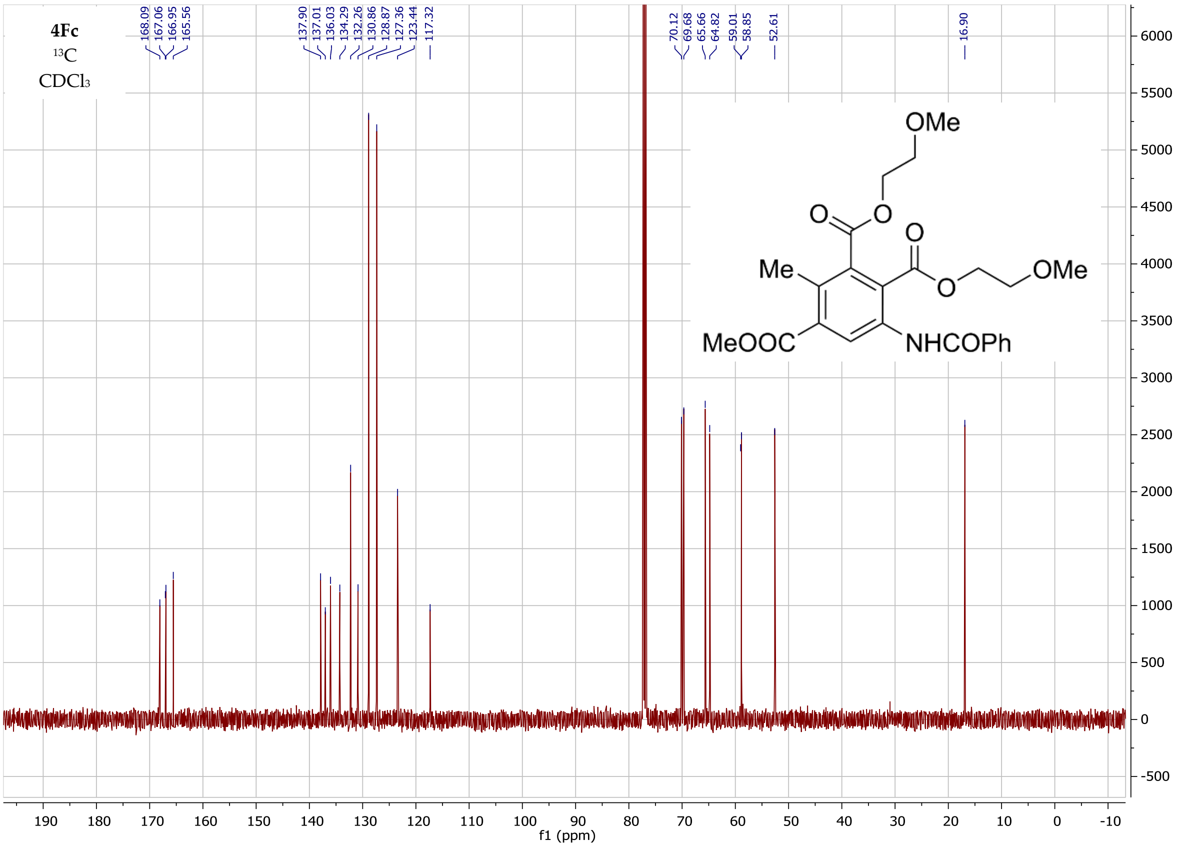
4Fc
¹³C
CDCl3
OMe
O
O
O
Me
O
OMe
MeOOC
NHCOPh
f1 (ppm)

6

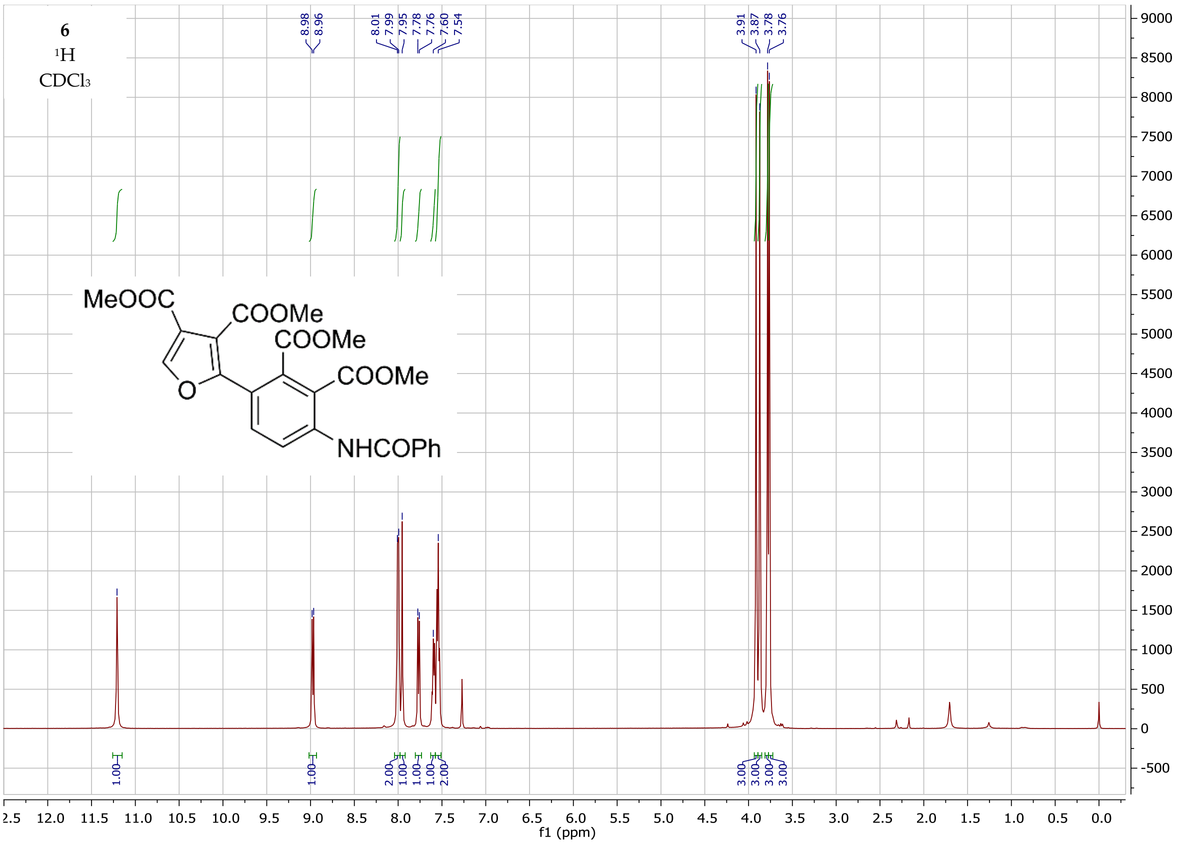
6
1H
CDCl3
MeOOC
COOMe
COOMe
COOMe
NHCOPh
f1 (ppm)

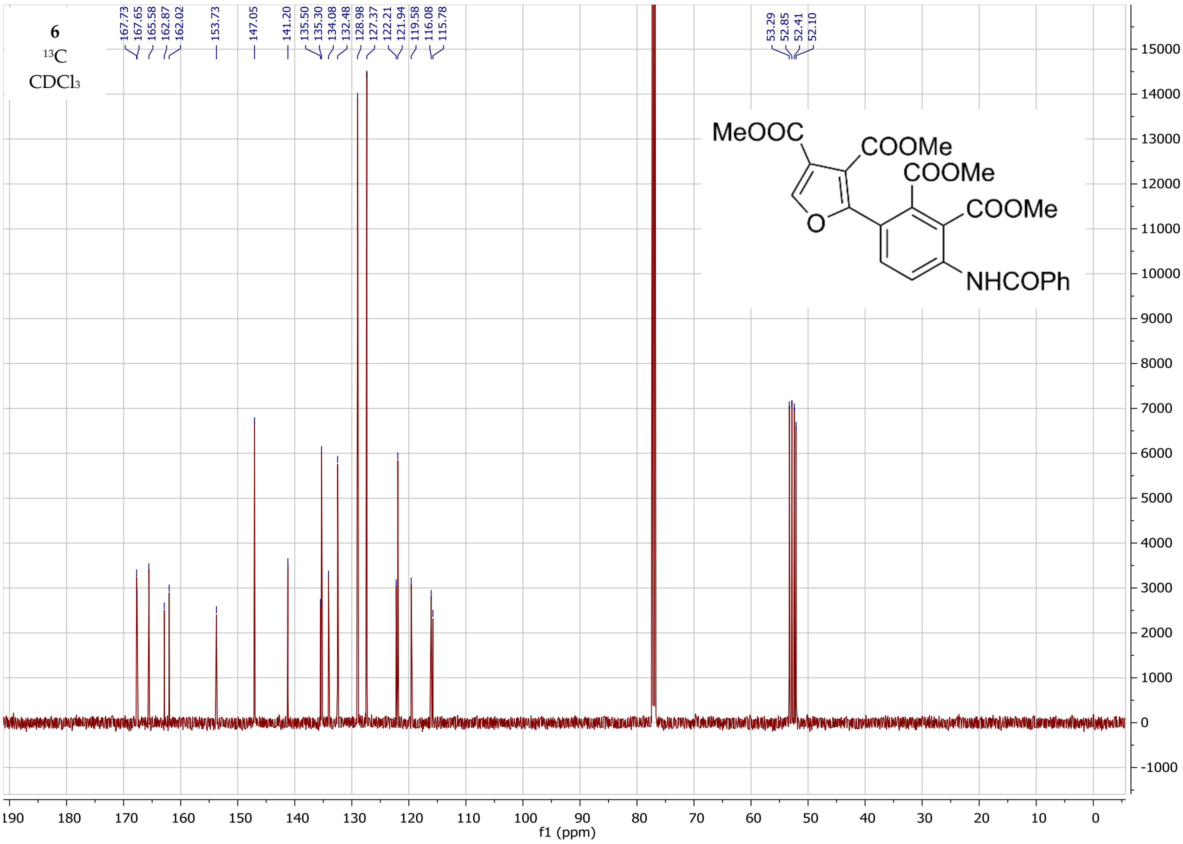
6
13C
CDCl3
MeOOC
COOMe
COOMe
COOMe
NHCOPh
f1 (ppm)

Coordinates of the optimized structures were obtained using DFT with ωB97X-D4 density functional and def2-TZVP basis set along with def2/J auxiliary basis set. Transition state structures were calculated using Nudged Elastic Band with Transition State (NEB-TS) optimization method. In all calculations xylene solvent was treated *via* the conductor-like polarizable continuum model (CPCM). Electronic energies of the optimized structures were calculated using DLPNO-CCSD(T) theory with def2-TZVPP basis set and def2-TZVPP/C auxiliary basis set. ORCA v6.0.1 was used.

For each optimized structure the electronic energies determined using DLPNO-CCSD(T) theory are labelled as $E_{\mathrm{el}}^{\mathrm{sp}}$. The Gibbs free energies without the electronic energy, calculated using ωB97X-D4/def2-TZVP are labelled as $G - E_{\mathrm{el}}$. All energies are given in Hartrees (Ha). Imaginary frequencies of transition state structures TS1 and TS2 are also reported.

Abbreviations used in this file to designate the structures are (*cf.* Scheme 1 in the main article):

R = reactants (3-acylamino-2*H*-pyran-2-ones **1** and dimethyl acetylenedicarboxylate **2A**)

TS1 = transition state for the reaction **1** + **2A** → **3**

IM = intermediate bicyclic product **3**

TS2 = transition state for the reaction **3** → **4A** + CO_2

P = products (dialkyl 3-acylaminophthalates **4A** and + CO_2)

Schematic representations of the reaction coordinate for **R** → **P** are given for each case (see also Figure 1 in the main article). Images of the transition states **TS1** and **TS2** are included. On figures showing **TS1**, general electron density transfer (GEDT) values are given, and the direction of the electron flux is indicated with an arrow. For **TS1**, the distances between the relevant carbon atoms on the diene and the dienophile are indicated in the figures.

Each case is labelled according to the designation of 3-acylamino-2*H*-pyran-2-on **1** (*cf.* Scheme 1 and Table 1 in the main article).

CASE 1a

R

44

C	1.55077856718361	2.25644221383553	-3.12993195017840
C	0.53568706675764	-2.23287963434132	-0.81578146125945
C	0.24433617644859	1.95093855553335	-1.13663713958196
C	1.23118973244187	1.20653730529431	-0.58806353188406
C	0.42034370298731	2.49724906403421	-2.44843779335698
C	0.15284840387934	-1.60983560012617	-1.76402833529236
O	2.55158931929817	1.52221893271382	-2.56805025056134
C	2.47136811428200	0.98151674535886	-1.32442084823124
O	3.41552590068732	0.35500774608449	-0.89729783372691
C	-0.24720845174285	-0.86866717736573	-2.95470601057043
C	0.95986132101350	-3.03295844205444	0.32867203709085
O	0.36552705895600	-4.00948216935356	0.70522685031172
O	2.06567985043411	-2.53911996977379	0.87310138030718
C	2.55853415491130	-3.25579710449729	2.02364965441305
O	0.49516147751785	-0.66869327404665	-3.88212277364153
O	-1.50949087726354	-0.46773523315658	-2.87165288160077
C	-2.00915169233801	0.24131504859905	-4.02249227128794
N	1.23840183093536	0.59498200861921	0.66531967589943
C	1.88540739429464	2.69090190576227	-4.51314441928791
C	0.14936956734216	0.34606507126024	1.44922574633872
O	-0.98549546479839	0.67423021448183	1.14446336195283
C	0.44965667001012	-0.37483556395029	2.72898918561253
C	0.89021671844317	-1.70226659821735	5.13193824610764
C	-0.50437717413227	-1.26158440975115	3.22061700435691
C	1.62321840153491	-0.15067160915378	3.44441906370773
C	1.83784984751359	-0.80794531170495	4.64893604245982
C	-0.27891371779529	-1.93259573367154	4.41403154090475
H	-0.67466273879582	2.12155070323600	-0.59615320470819
H	-0.36023416200223	3.09170010089400	-2.90293462779484
H	2.80625902237397	-4.27954175769669	1.74247470332746
H	3.44674887291528	-2.71490586861522	2.33911575965561
H	1.80539045846701	-3.25434830405503	2.81083585606151
H	-2.00314455653432	-0.41596846768580	-4.89266050698275
H	-3.02521942228576	0.52894963873648	-3.76385138227677
H	-1.39379576443623	1.11881872584679	-4.21772662982177
H	2.10590738830744	0.12666714736453	0.88561716913310
H	2.81297099532391	3.26889868051158	-4.51770961491867
H	1.07958970018177	3.30103385271590	-4.92031018362072
H	2.02986708775886	1.81458054393115	-5.15062750929712
H	1.06064711874298	-2.21861281960491	6.07056947979929
H	-1.41783877116454	-1.41907669729469	2.65876987858628
H	2.36078474866775	0.55643726962283	3.07994671036490
H	2.74575070799460	-0.61931949907433	5.21142431905642
H	-1.01804180695754	-2.63278571663568	4.78728838701195

0 imaginary frequencies

$E_{\mathrm{el}}^{\mathrm{sp}}$ (DLPNO-CCSD(T)) [Ha] = -1313.445691393408

$G - E_{\mathrm{el}}$ [Ha] = 0.28173846

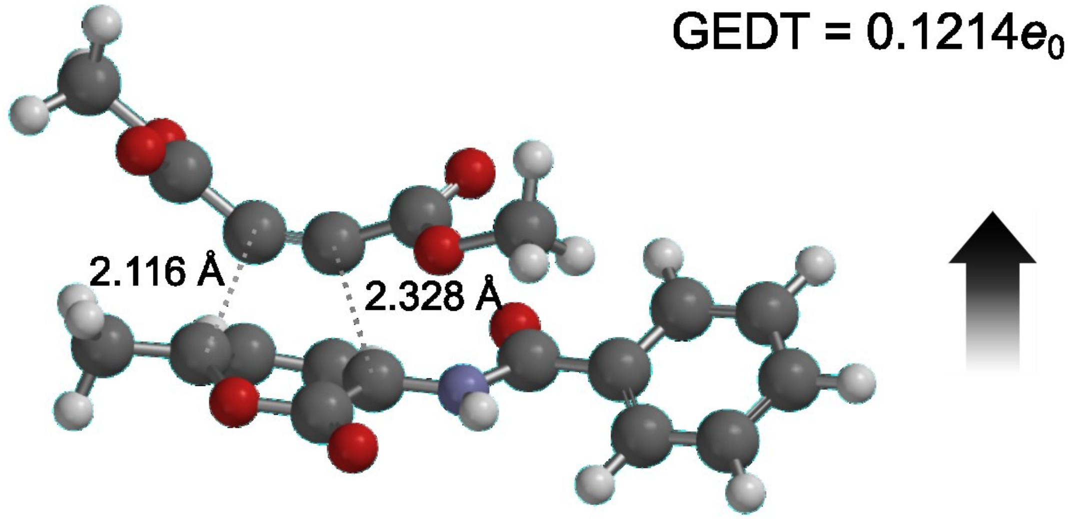

TS1

44

C	0.45760801892183	1.86952052055019	-2.59639732034281
C	-0.09293780132500	-0.52423129859338	-1.31974367037397
C	-0.58543587875864	2.13362471548042	-0.46909066616074
C	0.42434442745534	1.30616212377164	0.02302431271893
C	-0.57601695932906	2.39530700413387	-1.81912416906217
C	-0.24516847205715	-0.12325762551950	-2.47923955704141
O	1.66866234522306	1.62330047147614	-1.98818394062828
C	1.70621192340171	1.24263726792397	-0.69463018414003
O	2.75285216272829	0.91185784458094	-0.18806691775101
C	-0.66440727076079	-0.44911681804898	-3.84467772662031
C	-0.12401247607468	-1.50890612921650	-0.24889737537343
O	-1.13036490528099	-1.85067744839283	0.32177974118053
O	1.10215632645235	-1.94611137956575	0.03977174039832
C	1.18462425378110	-2.89254111446914	1.12189386417186
O	0.00109871426591	-1.08035044773930	-4.62615008015096
O	-1.86650550300906	0.06528561281905	-4.10947633719771
C	-2.36830353009957	-0.18928701014852	-5.43455957605877
N	0.53271166517560	0.84976966224586	1.32710650791365
C	0.64689619373203	2.18792376838373	-4.04662693944208
C	-0.50134996138380	0.68011763593033	2.21824423617138
O	-1.64042177466112	1.03185426705240	1.98141179351899
C	-0.11927431834845	0.00607117345093	3.49966774287977
C	0.46925432982658	-1.30157952300521	5.88151753704271
C	-1.10101437775068	-0.73934573746864	4.14760543325334
C	1.15591184890071	0.10393470563858	4.05248762075068
C	1.44629610938991	-0.54552067184203	5.24514893962354
C	-0.80465954398283	-1.39844753106157	5.33124630379960
H	-1.40925460752184	2.42170148329236	0.16484217217796
H	-1.41354528163625	2.86996491992641	-2.31369988970101
H	0.61921153937259	-3.79209845853078	0.87706950139176
H	2.24312850177232	-3.11955486012577	1.22473886825313
H	0.79377948030081	-2.45080916304190	2.03946759340623
H	-2.46264789515583	-1.26268487884618	-5.60050519524743
H	-3.34256392637741	0.29241483494774	-5.47258472504457
H	-1.69561185867270	0.23939984578623	-6.17854290510310
H	1.43854002023375	0.46179762097398	1.54854382786802

H	1.20204413943679	3.12503893293847	-4.13665187759358
H	-0.32050664655299	2.30508615386291	-4.53430290537678
H	1.21714218547211	1.40380181242129	-4.54568178999157
H	0.69941102718547	-1.81234108160468	6.81038603954898
H	-2.08912842432945	-0.80117919367781	3.70731829869364
H	1.92343423011903	0.70603390382348	3.57742915878530
H	2.43605417748860	-0.45687944755431	5.67918209627231
H	-1.56824220756760	-1.98768646295815	5.82695041858111

1 imaginary frequency (-443.70 cm^{-1})
E_{el}^{sp} (DLPNO-CCSD(T)) [Ha] = -1313.409403790151
$G - E_{el}$ [Ha] = 0.28404484

IM
44

C	0.89102062807562	1.62610515224381	-2.41909621866531
C	0.58345923546687	-0.45003094967135	-1.26414831889649
C	-0.23623880475256	1.68981511105830	-0.33948153254560
C	0.77319784022860	0.56519326941468	-0.12529278968064
C	-0.18689954584620	2.23097551429095	-1.54310481381005
C	0.61744190053077	0.12598875831305	-2.46099270483030
O	2.16168623298783	1.75943833450062	-1.68976520108147
C	2.15508593826728	1.18999548928475	-0.47818609800158
O	3.12350102910261	1.14745840521014	0.22332724217006
C	0.50033142381543	-0.57823682641427	-3.77544560446027
C	0.41889573436497	-1.92600753767975	-1.15464569272177
O	-0.09404096195614	-2.59166228473396	-2.01875364343396
O	0.92264582082900	-2.43789464799681	-0.02732089492821
C	0.81363559087755	-3.86802010243283	0.11912199264037
O	1.42907611978640	-1.10253282641458	-4.33306069865153
O	-0.73098869133899	-0.47370394345427	-4.26506527144617
C	-0.94594253522099	-1.10903286312253	-5.53990883808641
N	0.76206701424909	0.01452990423400	1.19608005794061
C	1.08851880459910	2.29495753089616	-3.75636666208810
C	0.94455329389152	0.79845136913860	2.29529536157694
O	0.88736881303672	2.01685204305304	2.25425693898983
C	1.17964732805390	0.05749393066761	3.57817231530417
C	1.60534152262527	-1.21682047171817	6.01449469616167
C	0.73462001804925	0.64275826285349	4.76036468152589
C	1.85352237415032	-1.16067197190430	3.62122351956748
C	2.06892232605914	-1.79389028554247	4.83859335488860
C	0.93824279307998	0.00313401170491	5.97425339828369
H	-0.87919908625372	1.98721899440388	0.47453698105278
H	-0.80959096273737	3.03715353379052	-1.90774006743364
H	1.35724044558231	-4.36371483369277	-0.68480294096797
H	1.26000208740914	-4.09361882162955	1.08471683445592
H	-0.23477388732568	-4.16504143122065	0.09718790222999
H	-0.74792676864035	-2.17795406216801	-5.45695380882672
H	-1.99052701651327	-0.92817419220845	-5.78188020935722
H	-0.29201875419450	-0.66903823571767	-6.29347590692691
H	0.89434137459013	-0.97826183569796	1.27861863090604
H	1.32922840727850	3.34785836388277	-3.60385701828583
H	0.16684854666992	2.22610394923022	-4.33766087888002
H	1.89946877582621	1.81995871243758	-4.31038121710858
H	1.77146900108823	-1.71366427195844	6.96436430907098
H	0.23016056683491	1.60086480354152	4.71428750719571
H	2.24810082738269	-1.60633731330691	2.71415538146194
H	2.60740939996784	-2.73481861458048	4.86914105921464

H	0.58109780002265	0.45828787911559	6.89158186647741

0 imaginary frequencies
$E_{\mathrm{el}}^{\mathrm{sp}}$ (DLPNO-CCSD(T)) [Ha] = -1313.488264474423
$G - E_{\mathrm{el}}$ [Ha] = 0.28880427

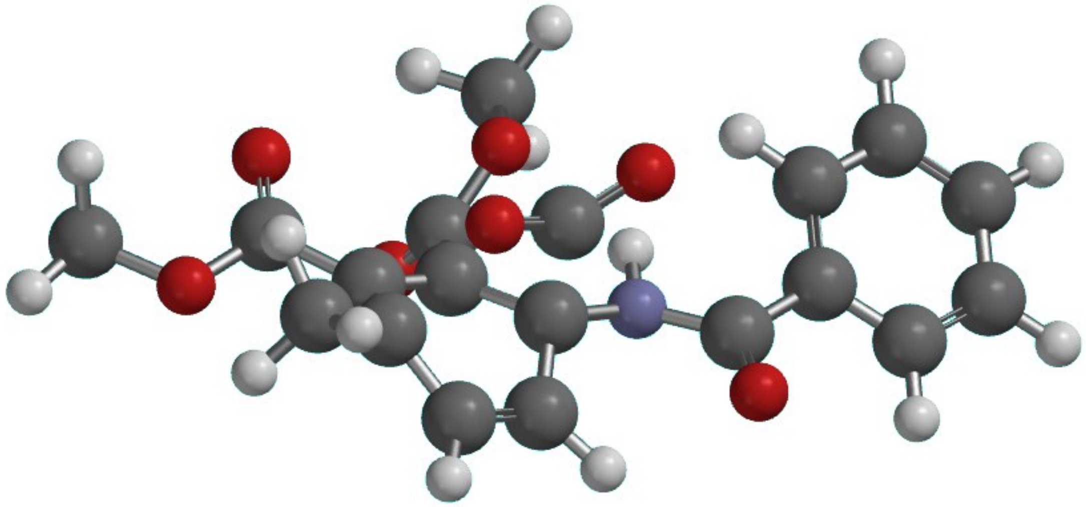

TS2
44

C	-0.26570272791502	1.82652778967114	-2.45802620153827
C	-0.54444858518074	-0.07502663835230	-1.07129927062887
C	-0.99880749788246	2.18261756015234	-0.23701848764833
C	-0.33078934740502	0.89917571894551	0.00820212304905
C	-0.95356524924982	2.64790036984189	-1.49353370569186
C	-0.50004158907948	0.39664920757744	-2.33195391039274
O	1.48177819616653	1.79412707534186	-1.53757431108176
C	1.36567449600819	1.33010074422955	-0.39128266714962
O	2.11706105972242	1.08761383437846	0.51824924159771
C	-0.51071581018501	-0.54186526522985	-3.49232346199362
C	-0.73655866835449	-1.53241882715314	-0.77321879813492
O	-1.75665543173470	-2.12130528361339	-1.00864146870452
O	0.33284160013493	-2.05205423190293	-0.17207616334394
C	0.24520692070131	-3.45450970183250	0.15596976202438
O	-0.04034540084695	-1.65247312639698	-3.45670113442331
O	-1.11599456157049	-0.01847495744815	-4.55684601897439
C	-1.16111820634915	-0.85983849480180	-5.72367359714819
N	-0.35690699629034	0.36898891174634	1.32794054194084
C	0.17359337705508	2.44802858304212	-3.74049236865941
C	-0.07073444733600	1.11725729793566	2.44155420230485
O	-0.17595014443842	2.33029929344153	2.48250023752185
C	0.33267363581747	0.32021649841158	3.64584571235747
C	1.07940538068747	-1.05975321198229	5.94186556611267
C	-0.06543009573499	0.78082306958287	4.89792841192367
C	1.12114558672930	-0.82361481739664	3.54691303676956
C	1.49668732404370	-1.50922915731336	4.69513742381418
C	0.29828355437300	0.08698105275272	6.04247974233514
H	-1.36974948601539	2.75033160375175	0.60158602974805
H	-1.27746319961615	3.64306447688659	-1.77135169861731
H	0.10184651705009	-4.03465310919036	-0.75538210181798

H	1.19420837982859	-3.70022723068866	0.62605967328424
H	-0.58493327586475	-3.62614300165784	0.84164535677457
H	-1.71472993180857	-1.77289463683609	-5.50371022100833
H	-1.67130152478783	-0.27408862366064	-6.48478520571612
H	-0.14927559425763	-1.11137405635294	-6.04319464058141
H	-0.09320902297569	-0.60008988081665	1.39729065397750
H	0.62973557456741	3.41595416518189	-3.53265534654016
H	-0.70287359080872	2.60129603597928	-4.37495500602395
H	0.88274018930263	1.81616230516697	-4.27267660831711
H	1.37064796672450	-1.59813555396085	6.83743213084155
H	-0.66089188320337	1.68420928487522	4.96180515468656
H	1.48363694621871	-1.16249174810149	2.58192358507116
H	2.12414691475970	-2.39021450383063	4.61570817251422
H	-0.02312135099981	0.44255117962679	7.01533563548692

1 imaginary frequency (-580.35 cm^{-1})
$E_{\mathrm{el}}^{\mathrm{sp}}$ (DLPNO-CCSD(T)) [Ha] = -1313.459412671241
$G - E_{\mathrm{el}}$ [Ha] = 0.28569508

P
44

C	0.05289146262843	1.97602690891475	-2.65515016430722
C	0.40677706858943	0.04425869396559	-1.21817763051975
C	-0.01736083555754	2.19510929656160	-0.23633923897517
C	0.24763965340393	0.83714029502175	-0.07752971585823
C	-0.10785584547373	2.74215227865540	-1.50273285500520
C	0.30436730831325	0.61645179776459	-2.48936117883491
O	3.48057430443945	1.45041724251528	-1.50765689036085
C	3.44575008133249	0.75763645871897	-0.58046364710650
O	3.41876229705621	0.06474160318441	0.34973434890154
C	0.46832541411795	-0.25073157660264	-3.70115880310123
C	0.81609887690772	-1.39240752041271	-1.12616235285658
O	1.74138854243090	-1.86102928458125	-1.74117625022468
O	0.05310850461366	-2.10108824528724	-0.28452341882912
C	0.41070374893038	-3.48857723758280	-0.13829918493372
O	1.24008516281877	-0.03312149840228	-4.59973102114081
O	-0.37652981663350	-1.28900385373984	-3.67478899306487
C	-0.21351228514755	-2.24463018207317	-4.73503331499080
N	0.37163953680483	0.26570662043631	1.20397262615434
C	-0.05678709106066	2.61793647199651	-4.01379982814110
C	0.98098153887627	0.85159527373733	2.27878674786393
O	1.34784493331733	2.01357954353316	2.29921482056088
C	1.16181805246998	-0.05094924430786	3.46414455018893
C	1.53768701093283	-1.62546475666670	5.72726360680523
C	1.10756058948566	0.52248106006663	4.73181921992215
C	1.42224884828374	-1.41292144438500	3.33291494561906
C	1.61410960442310	-2.19703785893911	4.46324432361374
C	1.28452793475072	-0.26410829911070	5.86042291068142
H	-0.14370381965381	2.81652960896177	0.63761649213438
H	-0.32087284436603	3.80140388439672	-1.60217192431958
H	1.43828122007209	-3.57636048280677	0.21610662966243
H	-0.28681570821729	-3.89341835135364	0.59142393550905
H	0.30806817656297	-4.00017392854978	-1.09535027034291
H	0.78728011304054	-2.67697976009246	-4.69145732305598
H	-0.97004995611191	-3.00686741364710	-4.56139023366784
H	-0.36497975473286	-1.76647659916118	-5.70315932861780
H	0.12145994057286	-0.70554043615654	1.28377873201827
H	-0.47073593393486	3.62298096773668	-3.92122562130433

H	-0.69959506848845	2.04037968636792	-4.68169785427246
H	0.92395126760514	2.68830962142022	-4.48835296072583
H	1.68362009615149	-2.23910339015543	6.60975042763747
H	0.92459870025790	1.58704353472874	4.82004685368453
H	1.51514610192835	-1.86520054830625	2.35146058374864
H	1.83164118825194	-3.25403457400825	4.35543948573080
H	1.22986168000782	0.18478563764439	6.84613876412061

0 imaginary frequencies
$E_{\mathrm{el}}^{\mathrm{sp}}$ (DLPNO-CCSD(T)) [Ha] = -1313.566766364466
$G - E_{\mathrm{el}}$ [Ha] = 0.28222460

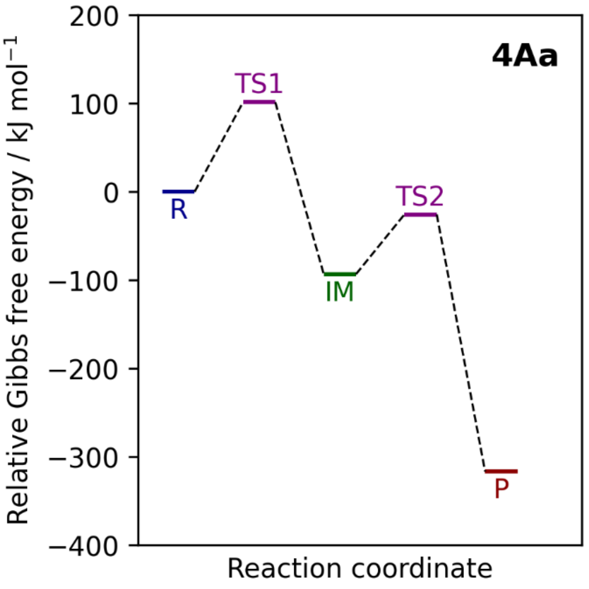

CASE 1aa

R

48

C	2.65177122023406	0.94764823495745	-3.14490593057672
C	0.85717744547356	-2.23437157065111	0.05326869242995
C	0.86476768439482	1.45359138127485	-1.64293468781947
C	1.63006386484316	0.99831703618484	-0.62230530117202
C	1.40919376845183	1.40854743685096	-2.96110395076703
C	-0.12630648325349	-1.89792936880341	-0.54151323648614
O	3.44435231823705	0.51494530320037	-2.13658467027041
C	2.98283325498288	0.49510716529636	-0.85780691332290
O	3.70714699962369	0.06249343546218	0.01591420881307
C	-1.29333540607134	-1.40754136159235	-1.26760044481826
C	2.08106108237729	-2.62403461746606	0.74738852434029
O	2.85692469816529	-3.43716109218071	0.31922881382049
O	2.19931812760763	-1.95913773959958	1.89174987987712
C	3.42336392601871	-2.18755888863042	2.61680385607828
O	-1.22445480540173	-0.88145869067594	-2.34838937566501
O	-2.40542185495974	-1.62249993870217	-0.57657386908687
C	-3.60755129588027	-1.09282750550132	-1.16778626969206
N	1.26719772351479	0.90843964285774	0.72615058918552
C	3.13196241425721	0.97640054166373	-4.55767420492456
C	-0.00690771229637	0.84069662342643	1.20647565051942
O	-0.99581896021735	1.00696836419946	0.50810454812668
C	-0.11272500805417	0.51039699254938	2.66341608738627
C	-0.40530289686607	-0.18337514446608	5.34022725256883
C	-1.24183170073341	-0.18629540059290	3.08434617180817
C	0.86595561049614	0.86808976019439	3.58761746511705
C	0.71556635809479	0.52604050416578	4.92485185694440
C	-1.38369829181806	-0.53979097686698	4.41837273808975
C	0.81628837141564	1.83865241673492	-4.27678725784347
C	1.80768428893850	1.24669938372551	-5.31320757163580
H	-0.13639880010833	1.81460613342557	-1.46097224394852
H	4.27139708155467	-1.87901104047822	2.00546002034979
H	3.51148726902066	-3.24172060081314	2.88016769391571
H	3.34462468946051	-1.57188560689860	3.50985350590949
H	-4.40769072319681	-1.36000480730122	-0.48189908529068
H	-3.52275634011660	-0.00973918025845	-1.26018505395388
H	-3.77288007372721	-1.53998216873679	-2.14834031726301
H	2.01003755646420	0.56175187120841	1.31585826101910
H	3.86374560785030	1.78061148432613	-4.69349796337859
H	3.61102891531803	0.04106665419008	-4.85569420253003
H	-0.51903905796089	-0.45403221330392	6.38453175050969
H	-1.99584884053102	-0.45465516787185	2.35375321709986
H	1.73595913971924	1.43697649971307	3.27745747894254
H	1.47268523811309	0.81822525912468	5.64434362867953
H	-2.25906195750821	-1.09310078187857	4.74061807536360
H	0.76987765059709	2.93140640283462	-4.33752598436232
H	-0.19859453892973	1.46029317246025	-4.41794322398406
H	1.95048701137402	1.90266040852826	-6.17260009320448
H	1.41166543103170	0.29828175471434	-5.68250811489843

0 imaginary frequencies

$E_{\mathrm{el}}^{\mathrm{sp}}$ (DLPNO-CCSD(T)) [Ha] = -1390.718619013395

$G - E_{\mathrm{el}}$ [Ha] = 0.31685035

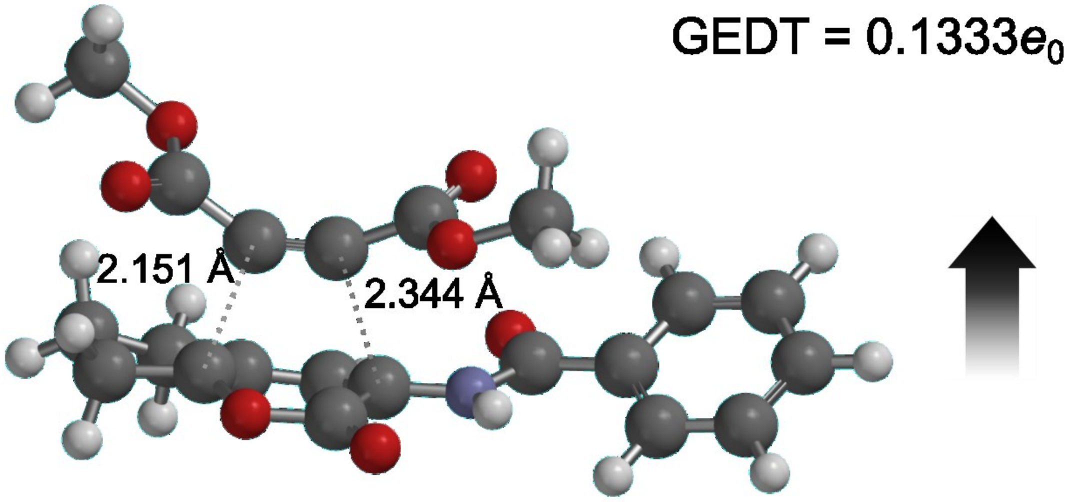

TS1

48

C	0.96854135480858	0.63539426418744	-2.65453343877993
C	0.01778544230985	-1.08086170673408	-0.68679149921774
C	-0.16112183984550	1.76213015527316	-0.88824159424236
C	0.71300400839222	1.06585478683720	-0.05207848395271
C	-0.03470352066042	1.50645648112626	-2.23282768243484
C	-0.02802784685578	-1.12405939775018	-1.92072241763912
O	2.10117620859385	0.45771358219605	-1.91484604412739
C	2.00408101610624	0.57352994965576	-0.57079330605005
O	2.95442498170759	0.31189250256935	0.12928231667089
C	-0.38324496162562	-1.92125408969003	-3.09457153376018
C	-0.19165874778058	-1.56985736820697	0.66558125685556
O	-1.24689833514078	-1.50670415062891	1.24818219597986
O	0.93758515613702	-2.05022086990336	1.18908956366983
C	0.84028111332818	-2.51628012596622	2.54774011832176
O	0.41145993259301	-2.51855189598602	-3.77795485007054
O	-1.70057900755408	-1.89661753559281	-3.31139782146676
C	-2.15207800635826	-2.66060693188246	-4.44463797319187
N	0.70614284763149	1.10533375123411	1.33307126358176
C	1.07789457258626	0.60003652801988	-4.15014418375536
C	-0.36485769587019	1.41315101541032	2.13876563707872
O	-1.42562602622210	1.81698215672106	1.70396256531923
C	-0.12923898821610	1.19179500478228	3.60136015573895
C	0.17735748196258	0.74365455139535	6.32838213772903
C	-1.23793947130371	0.89155900791455	4.38853876305666
C	1.13323852392160	1.27707350909679	4.18490973294564
C	1.28341230371526	1.05803580815897	5.54800225684998
C	-1.08363158759640	0.66013967716474	5.74713366259884
C	-0.85081465306822	1.95120154626373	-3.41539629388040
C	-0.32641096391922	1.07267073522457	-4.57769011192235
H	-0.96119049408494	2.35438618048728	-0.47125369439032
H	0.13114090197137	-3.34215383725921	2.61173066286538
H	0.51926131686707	-1.70527664679840	3.20277024761760
H	1.84207983017047	-2.84848604920474	2.80984942160222
H	-3.23101347935031	-2.52697144138977	-4.47153297168849

H	-1.69329852592072	-2.28284072470534	-5.35955860549984
H	-1.89684080663824	-3.71258562002973	-4.31438873693502
H	1.53273980537554	0.70430152500760	1.75267807805889
H	1.85157153402333	1.31638117761316	-4.44746637960358
H	1.35525424930024	-0.37701629853889	-4.54384755782577
H	0.29747015485653	0.56785268869834	7.39202309953043
H	-2.21330374877402	0.83335806033017	3.92033902040835
H	2.00409660057261	1.54000258060053	3.59348553962017
H	2.26553730274923	1.13731377541983	6.00079119678343
H	-1.94778969388822	0.41504196508649	6.35471474353769
H	-0.65422565879243	3.01179298077452	-3.60394248473783
H	-1.92477070728304	1.84136639035156	-3.25362651173500
H	-0.30286155460835	1.61211000920598	-5.52449260824511
H	-0.98341031832288	0.21183234346009	-4.70964685126833

1 imaginary frequency (-404.25 cm^{-1})
$E_{\mathrm{el}}^{\mathrm{sp}}$ (DLPNO-CCSD(T)) [Ha] = -1390.688261451636
$G - E_{\mathrm{el}}$ [Ha] = 0.31987979

IM
48

C	1.19926355663258	0.50207029056080	-2.54396310562050
C	0.69360225862353	-0.91900705914254	-0.70396648841004
C	0.05203301433319	1.47982583465311	-0.71692254048902
C	0.96459686128218	0.44670676869238	-0.04807270915501
C	0.16803407082886	1.49219057393679	-2.03053655168935
C	0.79284776062388	-0.86685493481871	-2.02890802864564
O	2.47548082688029	0.79922482581394	-1.89809436924749
C	2.40248305804081	0.76485265114787	-0.55784750049080
O	3.35812776145811	0.92926280298689	0.14321166196663
C	0.63358426999245	-2.01515770692529	-2.97237862039466
C	0.39441588483864	-2.21568974421560	-0.03654454628964
O	-0.16442652315174	-3.12454106652103	-0.59804788754940
O	0.83126170509300	-2.28396988031514	1.22499856797338
C	0.58859954514710	-3.52944178803933	1.90899561915212
O	1.52062059139486	-2.78339628168482	-3.23962346335381
O	-0.57493941244998	-2.01540842333138	-3.52691790263121
C	-0.82096677163797	-3.07090095049760	-4.47551534131825
N	0.89970065979610	0.45813729809938	1.38253681533588
C	1.27664717692694	0.73789487427477	-4.04368676645856
C	1.14505406055712	1.58719040753984	2.10292083965577
O	1.20807365698362	2.69612040199740	1.59725458303410
C	1.29188642080993	1.38132050972004	3.58233061392501
C	1.55936380213553	1.11793268717325	6.33976286066945
C	0.90685150724860	2.42045940934163	4.42499991001063
C	1.82658283609574	0.21503514101385	4.12466789839804
C	1.96313580673492	0.08637198035540	5.50096845229365
C	1.03140301423813	2.28610426846455	5.79996894093734
C	-0.44062454524441	2.23307154078166	-3.18411119090260
C	-0.09929214843289	1.33446996830198	-4.38875407393053
H	-0.56656116681308	2.11599576065221	-0.10224981810652
H	1.09774074768718	-4.34092594658809	1.38952678786962
H	-0.48214319343541	-3.72872789308716	1.94999798262149
H	0.99582945090251	-3.39379323604324	2.90815337940581
H	-1.84004464560003	-2.91761478130147	-4.82263622280736
H	-0.11533389105378	-3.00273645701050	-5.30409706873161
H	-0.72174475035564	-4.03906483949141	-3.98414703097241
H	0.93105150580800	-0.43084464292836	1.84963887057069

H	2.06369058521846	1.47575813814955	-4.21749694806019
H	1.52501062574796	-0.15871089190648	-4.61328844648263
H	1.66403541615401	1.01514408084887	7.41457010204793
H	0.51121649255552	3.33011510610324	3.98855562374258
H	2.17502597965826	-0.58569857393210	3.48068727190966
H	2.39390937501260	-0.81746602475481	5.91785215403240
H	0.72121093360878	3.09501083105155	6.45241133026664
H	0.04744468095226	3.20791874171483	-3.28979316161171
H	-1.51024907122050	2.40770104118976	-3.06020562390739
H	-0.08407913905108	1.88034740018694	-5.33292441395124
H	-0.83940864155520	0.53451278778258	-4.47364044461126

0 imaginary frequencies
$E_{\mathrm{el}}^{\mathrm{sp}}$ (DLPNO-CCSD(T)) [Ha] = -1390.767555941575
$G - E_{\mathrm{el}}$ [Ha] = 0.32447882

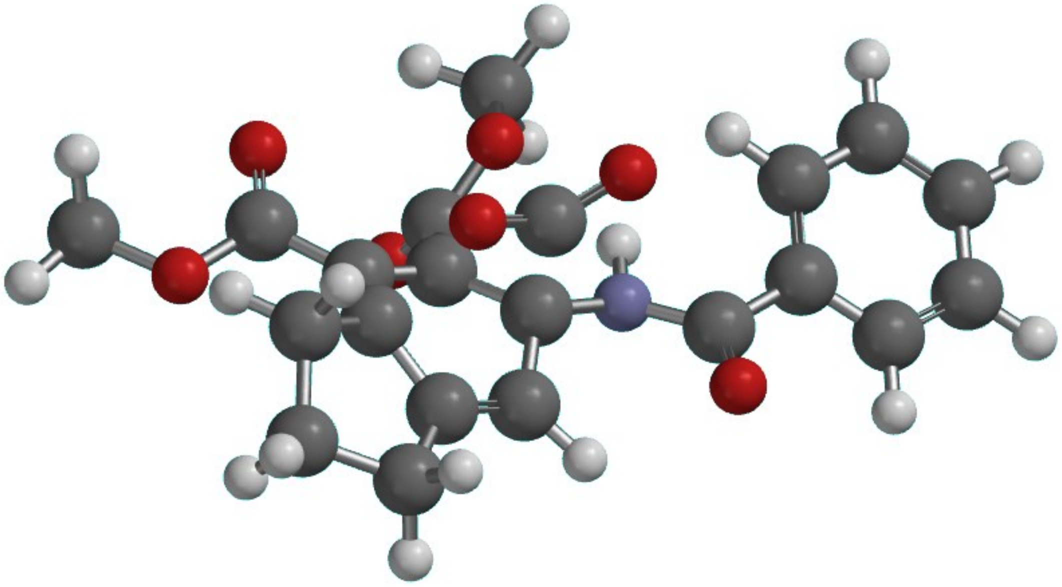

TS2
48

C	-0.02112278691743	0.44719510824025	-2.59189560849205
C	-0.40256978541998	-0.72565696289925	-0.59106678498915
C	-0.76615222035371	1.71281319234717	-0.73635741593965
C	-0.15892293326308	0.59553738564110	0.01157981229347
C	-0.67341748557784	1.62846969710774	-2.06824415214325
C	-0.31608828689037	-0.80707500484873	-1.93431608468346
O	1.70522186228211	0.72570716108498	-1.70339181734256
C	1.54750194985931	0.76244042491074	-0.46870302383205
O	2.28397671060406	0.88122520842850	0.47851666953727
C	-0.37923457266512	-2.12967725529668	-2.62095898425844
C	-0.67762897865275	-1.92864962929083	0.26117833466147
O	-1.72875117764474	-2.51027298521355	0.25336331687117
O	0.35761673776988	-2.21818319610700	1.04828186897435
C	0.18712084948522	-3.36408065216113	1.90855666052722
O	0.03051808312424	-3.15405720194553	-2.13280388489622
O	-0.96400755324867	-2.05123871889459	-3.81589018867177

C	-1.06882235873231	-3.29137996648561	-4.53922830440611
N	-0.22996851250833	0.63276773423785	1.43288273440880
C	0.30421340001005	0.68675991977662	-4.03813830223030
C	0.08049094158558	1.74457661492582	2.17415616135943
O	0.03459302804238	2.88157900767496	1.73951927560209
C	0.43155989193344	1.46060337320519	3.60441349189640
C	1.08299355715218	1.04273046409819	6.27529821023655
C	0.02993076180106	2.37691619286018	4.57246999437812
C	1.17679148474957	0.34343362843359	3.97411180890079
C	1.50480079566972	0.13861594159658	5.30827139869195
C	0.34570121592835	2.16319529758443	5.90606385676885
C	-0.93114758418374	2.60810146353607	-3.18344698424735
C	-0.73047086493397	1.75300505429311	-4.44980336251318
H	-1.10911515249937	2.58026898764721	-0.19422037978968
H	0.02372087295908	-4.25473978551560	1.30209841063340
H	-0.66031718101272	-3.20689285680863	2.57622418741894
H	1.11463226312336	-3.44306511884918	2.47021639481191
H	-1.55786824909791	-3.03998367126351	-5.47748638299505
H	-0.07613191844498	-3.70520592145065	-4.71917235546490
H	-1.66681506181808	-4.00442468321092	-3.97129747731790
H	-0.01605915176857	-0.23901570259739	1.88820354308849
H	1.31373106056420	1.10743260604118	-4.06754613776711
H	0.28735993450706	-0.21032581612396	-4.65009936773200
H	1.33632554179936	0.87932769214842	7.31735175896948
H	-0.53074106183811	3.25309278924684	4.26782921446763
H	1.54353187273246	-0.34828132449922	3.22261347772917
H	2.09902657720722	-0.72347366514008	5.59088859033139
H	0.02123587285331	2.87308912220398	6.65904222690361
H	-0.19037022179402	3.41332354333497	-3.13901304805920
H	-1.92072609795331	3.06327098818346	-3.12341746701682
H	-0.39596609484589	2.33738632589220	-5.30749755435746
H	-1.67017997367820	1.26281519392071	-4.71913633031632

1 imaginary frequency (-564.86 cm^{-1})
$E_{\mathrm{el}}^{\mathrm{sp}}$ (DLPNO-CCSD(T)) [Ha] = -1390.737579476659
$G - E_{\mathrm{el}}$ [Ha] = 0.32143327

P
48

C	0.26097754009034	0.55257525580669	-2.94149820659466
C	0.51289822145445	-0.63498344544264	-0.86041652843062
C	0.12887824102688	1.75485618233473	-0.85045487807576
C	0.34966106209640	0.56287285826669	-0.15746730634521
C	0.09439982979031	1.73953711272225	-2.22817513975315
C	0.45636280424953	-0.63419478216191	-2.26155346466780
O	3.49379391533602	1.10953897065563	-1.46229135633542
C	3.50686914180707	0.57039581574464	-0.43727270175790
O	3.53125161454722	0.02506740351333	0.58607173589024
C	0.58356162003812	-1.90220895870701	-3.04481577917963
C	0.88040513857847	-1.91099772981817	-0.17257977305659
O	1.78920451893371	-2.62027767702492	-0.52480202093318
O	0.09646678628777	-2.18579179191531	0.87998150605848
C	0.41421466218020	-3.39700997356033	1.59027004155340
O	1.30411420970655	-2.03856048882938	-4.00163276846074
O	-0.23839767270540	-2.85253542638684	-2.58537780013500
C	-0.10949043791048	-4.13807931570306	-3.21223262961618
N	0.43214319323989	0.57326233566640	1.25218728204000
C	0.18319913569937	0.81225355982104	-4.42579948472239

C	1.13047618245741	1.49302389651586	1.98148369849944
O	1.64790418785733	2.48483941785750	1.49535016127051
C	1.21516022765705	1.20340016760974	3.45152473402864
C	1.42391298072462	0.78352247783072	6.19479476293286
C	1.28947440587913	2.28777611625547	4.32181989007388
C	1.26262876980552	-0.09284147249772	3.95918102605530
C	1.37134614589395	-0.30115282729042	5.32808743004625
C	1.38320496119485	2.07890831220467	5.68980327738964
C	-0.09685425092993	2.89761025709173	-3.17487321566496
C	-0.47987804848420	2.20283705775725	-4.49614871905671
H	0.00038085515904	2.67449565553548	-0.29687726298331
H	1.43718423575362	-3.35563634393936	1.96605986603166
H	0.30194885423379	-4.25588599076504	0.92842640040203
H	-0.29806273040490	-3.44874843112473	2.41087193192818
H	-0.84051459723296	-4.77785094905593	-2.72247754359180
H	-0.31918572084819	-4.06269575121190	-4.27965443637045
H	0.90042199551149	-4.52289987158733	-3.06283987854592
H	0.08131969291057	-0.23987256151618	1.72873112301675
H	1.19559949111511	0.83203351823716	-4.84216625709792
H	-0.37048004399873	0.04473089194740	-4.96899138366639
H	1.50461842886822	0.61964838021606	7.26402006482277
H	1.27172024870791	3.29122173383691	3.91289925064694
H	1.25228186960111	-0.94925393326039	3.29381911338167
H	1.42239714292840	-1.31220585330584	5.71707751502977
H	1.42941469629914	2.92712062868985	6.36399868163792
H	0.85048830029772	3.43972558370616	-3.27459624598155
H	-0.84718003258768	3.61094536172093	-2.82818226827260
H	-0.17384978330424	2.77012956242588	-5.37646254519423
H	-1.56641198951172	2.08210506113426	-4.53719989824623

0 imaginary frequencies
E_{el}^{sp} (DLPNO-CCSD(T)) [Ha] = -1390.847688057941
$G - E_{el}$ [Ha] = 0.31769408

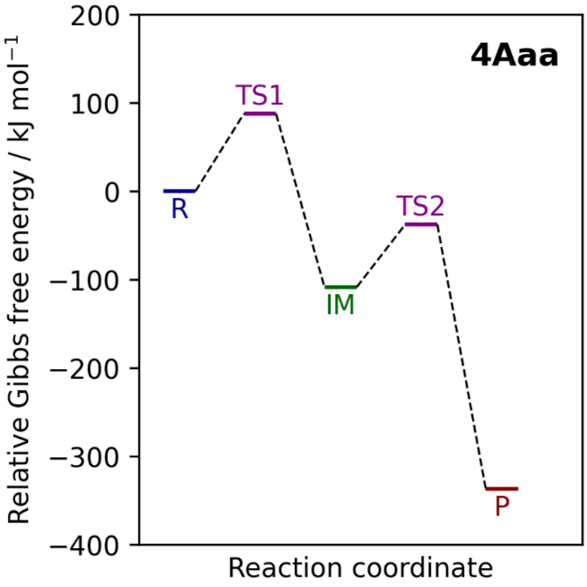

CASE 1b

R

51

C	1.26905602586151	1.07806259057109	-1.52925204582090
C	0.03604416638854	-2.06450906340364	-1.58412262355875
C	0.25677688400124	1.32606815259569	0.63710309994840
C	1.19920385813316	0.52474304151904	1.18669924604335
C	0.29717356636321	1.60349593991287	-0.76419998548382
C	-0.59545104092991	-1.38948296574274	-2.34447244652716
O	2.22320291729793	0.28065579997584	-0.96815673842233
C	2.25052979240248	-0.03761421473732	0.34778146800802
O	3.13190770836255	-0.76692732841824	0.75227743718014
C	-1.37242465395799	-0.52227625445088	-3.22792728887614
C	0.84704736679181	-2.89658423437134	-0.69774722348699
O	1.70382732307860	-3.64234073905602	-1.09364419525498
O	0.50833527976006	-2.70494366763648	0.57271138628778
C	1.25938901930819	-3.47251675417821	1.53494513813937
O	-2.11663928792167	0.33366587941995	-2.82315125318193
O	-1.14443262587761	-0.81106256740130	-4.50232286899046
C	-1.88129828200047	-0.02088813789334	-5.45592284397632
N	1.32205968968183	0.12339360168364	2.51521573456890
C	0.48112490568525	0.42219532913040	3.54673499603207
O	-0.50240845761234	1.13218024723661	3.41212253517406
C	0.84790447449586	-0.17232557835977	4.87622694261772
C	1.41967814409076	-1.18321859370611	7.40889000835234
C	0.28371499516564	0.40948610886967	6.00931779471153
C	1.69446492389967	-1.27056899191051	5.01910566450301
C	1.97658486330488	-1.77480034107265	6.28170993977534
C	0.57221209348106	-0.08949983755307	7.27071109440736
C	1.42106469552227	1.20789895845881	-2.98800986711560
C	1.63820190288222	1.40533214510089	-5.76300387124268
C	2.15850677461086	0.26587272110641	-3.70637511625324
C	0.79941008123788	2.25443123720757	-3.67211967599138
C	0.90854609599616	2.35111981799025	-5.05045667417212
C	2.26290920348135	0.36556106492031	-5.08652137589591
H	-0.53013072861400	1.74076390017754	1.24841716534584
H	-0.48224505649632	2.19977994653777	-1.21450734732432
H	2.30953309107473	-3.18389276170730	1.49357467047714
H	1.15316237924245	-4.53727253828361	1.32614495745987
H	0.82377962681125	-3.22384623016482	2.49966720638267
H	-1.53549496867399	-0.35242419296747	-6.43181984660145
H	-2.95021492796745	-0.20393649355500	-5.33966606061588
H	-1.66566428267254	1.03646128120201	-5.30886842605386
H	2.15462785812304	-0.41661422080267	2.69522194427138
H	1.64335579351463	-1.57696422244044	8.39455082925851
H	-0.38086291896695	1.25596027837774	5.88520204914206
H	2.12828154787572	-1.76307816625420	4.15608293481289
H	2.63028780256027	-2.63402612246152	6.38353537312605
H	0.13434143744436	0.37419099602157	8.14781970811073
H	1.72309070114316	1.48297707226214	-6.84156826414576
H	2.63812323521932	-0.55324530497317	-3.18547886951791
H	0.24040593140701	3.00502146328505	-3.12583409224121
H	0.42673291312356	3.17223443014733	-5.57025357124956
H	2.83264816286641	-0.37655248020838	-5.63489675213580

0 imaginary frequencies

$E_{\mathrm{el}}^{\mathrm{sp}}$ (DLPNO-CCSD(T)) [Ha] = -1504.821880308475

$G - E_{\mathrm{el}}$ [Ha] = 0.33174786

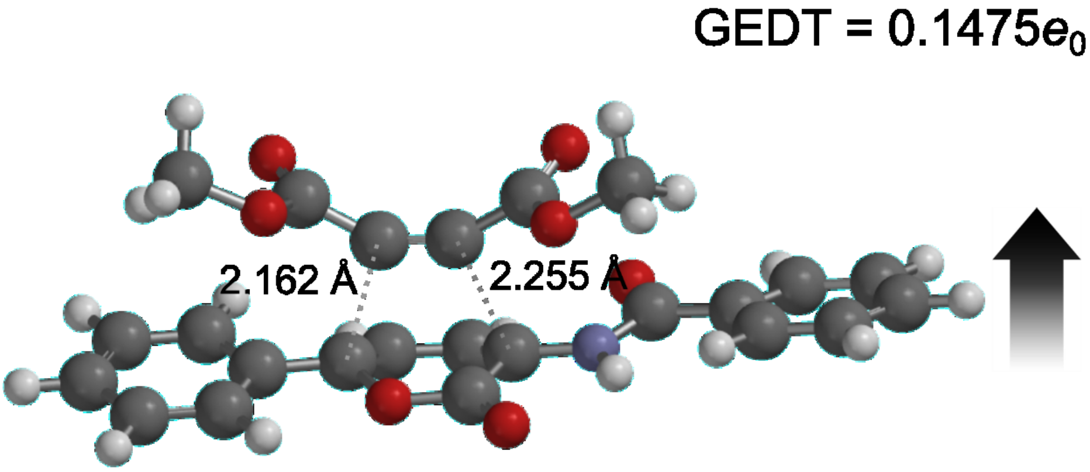

TS1

51

C	0.43755536514435	1.48118937052857	-1.73761312994127
C	-0.73124829764697	-0.61723612741111	-0.31305479800282
C	-0.16710623834524	1.98819748937156	0.51092491889817
C	0.53554815664725	0.82968643709898	0.86367317772097
C	-0.23475991453908	2.29964502437019	-0.82288575603316
C	-0.88397675903095	-0.20818350606281	-1.46988715942610
O	1.55130122387788	0.80471974270426	-1.29445918514574
C	1.62330580438636	0.37913332087220	-0.01912963323042
O	2.55305300952790	-0.30359216988383	0.34136260400436
C	-1.55102876868034	-0.32300484186985	-2.76493132151571
C	-1.00843558732549	-1.57552997655014	0.75324692810881
O	-1.99297976781418	-1.54201903706856	1.44706732394831
O	-0.02553523980205	-2.47013256770317	0.86760997447150
C	-0.22430113405684	-3.47465511686556	1.88002432233632
O	-2.59957263938741	0.21247268960443	-3.02568640517992
O	-0.83589190963231	-1.05976333473121	-3.61185477064506
C	-1.36032690983513	-1.14854605158872	-4.94958807804660
N	0.67891397050998	0.33208287200036	2.15025131546027
C	-0.16642287133840	0.54784734267266	3.21102915527870
O	-1.12126477651325	1.29763243264876	3.13927963477838
C	0.17019095612665	-0.19162387235225	4.47309695596069
C	0.66245693397062	-1.47781011573558	6.89615579283778
C	-0.53579573625859	0.16982050897155	5.61875998953218
C	1.12287597847204	-1.20728468128815	4.54885679226197
C	1.36599331521933	-1.84753394891518	5.75638441105765
C	-0.28984363149447	-0.46735912701361	6.82537698298098
C	0.49229437245543	1.74857309288077	-3.19407737965499
C	0.56809340148093	2.24010853779507	-5.93948634747633
C	1.55233423895009	1.27212711786897	-3.96540509963704
C	-0.53646289981955	2.45964424171849	-3.81212741502110
C	-0.49515852771506	2.70689723207889	-5.17622824350410
C	1.58881328752223	1.52065390942176	-5.32968572599083
H	-0.73578585958650	2.52373996129765	1.25470554688722

H	-0.88301195425987	3.08603873220842	-1.18176839404596
H	-1.09056909662198	-4.08815612855660	1.62977198907266
H	-0.37583331302476	-3.00753502362777	2.85298614255247
H	0.68387980212956	-4.07304634874670	1.87514050719462
H	-0.64755515308243	-1.75941420996988	-5.49857288711488
H	-2.34376444101074	-1.61933224471696	-4.93842605346968
H	-1.42997794321638	-0.15206012280856	-5.38645378788284
H	1.48076660973477	-0.26956522100722	2.26155932592533
H	0.85533017422537	-1.97882199353826	7.83869484645060
H	-1.27798248397649	0.95530137376522	5.54641069098273
H	1.67988857870208	-1.53321952563623	3.67769397975574
H	2.10588194205986	-2.63874493796873	5.80476824807508
H	-0.84270044065451	-0.17665485704327	7.71178592675503
H	0.59862166409465	2.43268674770614	-7.00644852740181
H	2.34910772000433	0.70848197081415	-3.49816467336206
H	-1.38733514346093	2.80463659757744	-3.23819454010411
H	-1.30184807913588	3.26040708166803	-5.64391826422976
H	2.42026901202412	1.14910126101540	-5.91856990722616

1 imaginary frequency (-456.51 cm^{-1})
$E_{\mathrm{el}}^{\mathrm{sp}}$ (DLPNO-CCSD(T)) [Ha] = -1504.787820600946
$G - E_{\mathrm{el}}$ [Ha] = 0.33446549

IM
51

C	0.70095300990842	1.15941185517969	-1.60588295765819
C	0.24713110967205	-0.81126049648559	-0.31044961692766
C	-0.16378511841592	1.46190782222888	0.57357662647622
C	0.69507129123866	0.21386102403588	0.74460788007553
C	-0.17813266520975	1.94778320095924	-0.65395291484147
C	0.21895932954645	-0.29279562953630	-1.53344424303172
O	2.04445475408417	1.13699543562923	-1.02298303358778
C	2.09879377461949	0.61636800973431	0.21196835943779
O	3.13004925311649	0.45554933823863	0.79577536292728
C	-0.23523137390657	-0.98028980381179	-2.78093390898044
C	-0.07137344168879	-2.25252844914288	-0.10956823049508
O	-0.69948850120718	-2.90050369152628	-0.90842535958920
O	0.43710912584497	-2.76156134917339	1.01685149692144
C	0.18795648311625	-4.16294326393679	1.24414781165663
O	-1.33114533182011	-0.81768912292556	-3.25007409061273
O	0.73695547814420	-1.70556581868862	-3.32053796970068
C	0.39814047029711	-2.37336089645934	-4.55081290660233
N	0.74354086859433	-0.26958646030592	2.09107806200033
C	1.13598955451138	0.54287152721217	3.11516491981610
O	1.32955211847002	1.73842758417766	2.97093652759112
C	1.29186180161876	-0.13532005768666	4.44360153403041
C	1.64291321862858	-1.29951316471815	6.94598584908334
C	2.13796375203440	0.45646026520164	5.37729122492178
C	0.61321498749686	-1.30758384063098	4.77066441441540
C	0.78652325510134	-1.88551881496106	6.02147472121848
C	2.31877775363822	-0.12784929764230	6.62243207719740
C	0.81276370460384	1.69840016469029	-3.00961358913998
C	0.94602130063447	2.58868856792328	-5.64597206666473
C	2.01378387399841	1.63312127785685	-3.70865006162083
C	-0.32302292566950	2.19902850787095	-3.63885950302109
C	-0.25676076231891	2.64245472294729	-4.95239830577072

C	2.07904348792790	2.08279837036573	-5.02140164788274
H	-0.65948412856451	1.88695614482280	1.43291111482957
H	-0.70945677125569	2.82979272914486	-0.98132750532218
H	0.62451067277334	-4.75169614049484	0.43770332886839
H	-0.88528424882149	-4.34550923969555	1.29738088140897
H	0.66840427073909	-4.39084024227715	2.19268723416933
H	1.29751929722979	-2.90715118135897	-4.84837972950259
H	-0.42564948931942	-3.06703817478195	-4.38012440168578
H	0.11545010780584	-1.63985259971701	-5.30657865976300
H	0.84602276417609	-1.26316242945967	2.20783702759279
H	1.77976711287322	-1.75352913519345	7.92162151151549
H	2.65121421247046	1.37313781431676	5.11189487610523
H	-0.07539580693537	-1.76166028429353	4.06601107254479
H	0.24701964736489	-2.79107120210669	6.27681579570390
H	2.98622324663089	0.33180258046204	7.34309152685329
H	0.99943942435473	2.93903962323211	-6.67112947596587
H	2.89866046654829	1.23740518586925	-3.22697327928633
H	-1.27056500971425	2.22749111403336	-3.11304777411496
H	-1.14808393241137	3.02967124846440	-5.43366043044899
H	3.02110152744540	2.03610467241312	-5.55686357514397

0 imaginary frequencies

$E_{\mathrm{el}}^{\mathrm{sp}}$ (DLPNO-CCSD(T)) [Ha] = -1504.862124935991

$G - E_{\mathrm{el}}$ [Ha] = 0.33854082

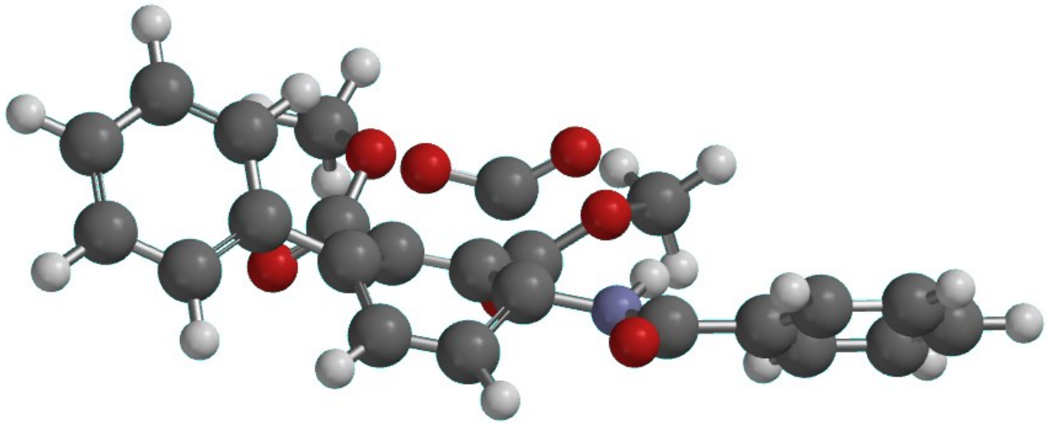

TS2

51

C	-0.49868183848562	1.50076763331989	-1.72953461741083
C	-0.64393927578062	-0.41641001993079	-0.32174354190731
C	-1.03434157820464	1.84447181380898	0.54025875913150
C	-0.32546508081406	0.57191983463937	0.73324729763075
C	-1.10827501885268	2.31729751090067	-0.71201336951657
C	-0.71127652403880	0.06504033642056	-1.57347468273859
O	1.32437197427019	1.47077744162603	-0.94957868477567
C	1.30308648024124	0.99257814608309	0.20082567722419
O	2.14643826626168	0.72779237809869	1.02152627608499
C	-0.97231437099302	-0.78497886973875	-2.77824643685285
C	-0.82225039360666	-1.88019176000931	-0.06662862492346
O	-1.72438809860760	-2.52214540920906	-0.53704813154451

O	0.12702119347467	-2.37599325108460	0.72867444203172
C	0.02970182335843	-3.78476668298210	1.02338632462337
O	-1.87395161363401	-0.57692690491619	-3.54774253652463
O	-0.08768869452663	-1.77466544155614	-2.88915128815604
C	-0.32270380312085	-2.69432244593905	-3.97054034820684
N	-0.26803417666921	0.06256291286589	2.06100132951641
C	0.09456463578559	0.83701913549595	3.13351364942529
O	0.16665900801467	2.05216352585841	3.09406027390186
C	0.37402231442830	0.06802572841160	4.39080011518826
C	0.94982928689875	-1.26234404853736	6.76567954992369
C	1.22179484667281	0.64922666725058	5.32975736457744
C	-0.19379383352059	-1.17789441810252	4.64972643244493
C	0.09135681910341	-1.83894110069859	5.83738100648441
C	1.51515246297319	-0.01745657640438	6.51027123687011
C	-0.19976275493334	2.03735201200517	-3.07140814506793
C	0.34937964483149	2.97221743141509	-5.63415213606336
C	0.80915138275328	1.44639483107885	-3.83375234914641
C	-0.92823584838222	3.10053766421801	-3.59916406131973
C	-0.65927894023992	3.55921662271916	-4.88138443121429
C	1.08661166494718	1.91712736956221	-5.10639195386508
H	-1.34231115442368	2.41081144319853	1.40498051195624
H	-1.46657672014984	3.31103177747926	-0.93942420275914
H	0.10532819119073	-4.35939738130774	0.10049463029196
H	-0.91928541349450	-3.99715450780527	1.51611037436380
H	0.86709405890862	-3.99908962165592	1.68274210243620
H	0.48550683742278	-3.41990986322912	-3.91608860708456
H	-1.28958234192521	-3.18110123113205	-3.83761892867747
H	-0.30340443157530	-2.16467179476303	-4.92343745270838
H	0.02145515141154	-0.90021843456214	2.12210724720508
H	1.17462267908147	-1.78108979811113	7.69148835220426
H	1.64863767998181	1.62254495489538	5.11867679590321
H	-0.88417034005484	-1.62696891049309	3.94364129948067
H	-0.36304548915392	-2.80249391184344	6.04079294467328
H	2.18429228421239	0.43481736131353	7.23402330889101
H	0.56237384429496	3.33678483802163	-6.63321186059496
H	1.39242054669513	0.63474449492340	-3.41477289993652
H	-1.72811674863353	3.55620311809364	-3.02881291358904
H	-1.24038516775938	4.37749190967715	-5.29152067816645
H	1.88038657436635	1.46221349063106	-5.68832441971404

1 imaginary frequency (-550.97 cm^{-1})
$E_{\mathrm{el}}^{\mathrm{sp}}$ (DLPNO-CCSD(T)) [Ha] = -1504.835950017181
$G - E_{\mathrm{el}}$ [Ha] = 0.33609774

P

51

C	0.42354777228888	1.33718326707979	-1.85284163843846
C	0.36038459830661	-0.47048936939024	-0.22887358506179
C	0.81522825148164	1.77947063352894	0.50030399349154
C	0.65787759769136	0.42797004977262	0.80819395770742
C	0.69679309698588	2.21374099256183	-0.80583380880330
C	0.24386920737454	-0.00688665203470	-1.54227502493915
O	3.61437570911671	0.26549643293404	-1.60348010806921
C	3.66010495484865	-0.17640979514214	-0.53351599345547
O	3.71595834982010	-0.62111325970353	0.53579752866817
C	-0.09354353172101	-0.97247870846896	-2.64321341549603

C	0.06807149353249	-1.91881106533450	0.01610475569505
O	-0.84390700335311	-2.51473241901750	-0.49806898533515
O	0.93693965852201	-2.49527323153921	0.86025639541347
C	0.70353859281995	-3.88778112428064	1.14446649362765
O	-1.08296090342253	-0.90947422533983	-3.32325039645792
O	0.86639726463582	-1.89400729545006	-2.78823296289877
C	0.60016435011013	-2.90660270493957	-3.77193506035823
N	0.79521499409796	-0.05144872594007	2.11958024547701
C	0.67196751880848	0.64893380786895	3.28753923757137
O	0.46876714606351	1.84890836231699	3.35081526263006
C	0.81656053452679	-0.18619985502786	4.52821064947727
C	1.09497384650220	-1.62079179333233	6.89779167004516
C	1.30365283460052	0.43962913987752	5.67292234474889
C	0.45448289989260	-1.53074935025971	4.57962329507241
C	0.59016595560566	-2.24428544917959	5.76346392785425
C	1.45111133166638	-0.27702883866114	6.85113494678775
C	0.38524367147071	1.84365417720290	-3.24909250023746
C	0.34144772308696	2.83409265520019	-5.85937282604678
C	1.19542587483865	1.27815463264906	-4.23222170205845
C	-0.43892428413105	2.91610185050455	-3.58307359625647
C	-0.46317764321248	3.40728112775331	-4.88168626768765
C	1.17233500788292	1.76955138632983	-5.53033888792558
H	1.03805124240948	2.47959364307463	1.28989972539170
H	0.85284130531193	3.26385452462968	-1.02736574999012
H	0.76874377206946	-4.46955456149276	0.22518176529779
H	-0.28148523931558	-4.01940983098726	1.59333282979851
H	1.48809341066079	-4.17671852357307	1.84014405674523
H	1.46943832570324	-3.56066523516896	-3.76306395693139
H	-0.30112383734806	-3.45848725354038	-3.50133783898394
H	0.47066629767668	-2.45198189389691	-4.75477165718810
H	1.02718470249139	-1.02766112345814	2.19677426971115
H	1.20396068958173	-2.18000952088028	7.82083157574872
H	1.56505653522317	1.49017562989043	5.62553961953175
H	0.03208844171962	-2.02532355331608	3.71154225881098
H	0.29364720016809	-3.28679760600727	5.80091594616412
H	1.84105628781029	0.21344905979880	7.73627476441815
H	0.32252494850858	3.21733599304900	-6.87394997694354
H	1.85993332990889	0.45978323350827	-3.97571681747165
H	-1.07703477396559	3.35641950711384	-2.82385399882830
H	-1.11619027643369	4.23663410551410	-5.13176778951240
H	1.81045076708156	1.32393875320349	-6.28604697051030

0 imaginary frequencies
E_{el}^{sp} (DLPNO-CCSD(T)) [Ha] = -1504.944508402895
$G - E_{el}$ [Ha] = 0.33267106

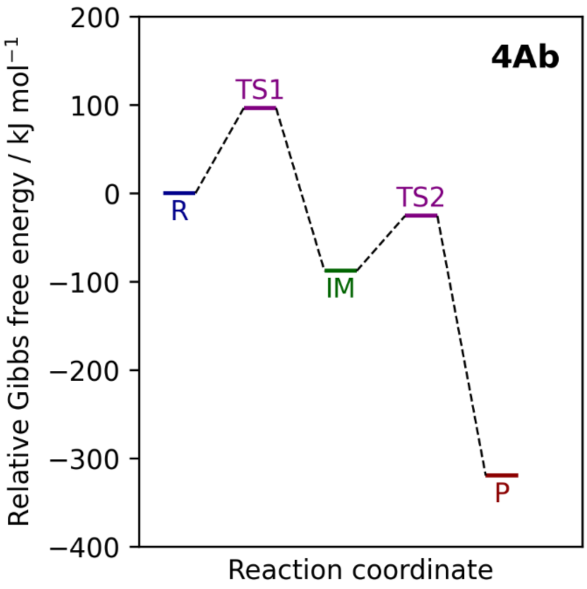

CASE 1d

R

55

C	1.22610098194822	0.96028897527873	-1.45919649267303
C	-0.48391605422935	-2.16522867787441	-0.02045684794927
C	0.29778940098511	1.01908960687774	0.75879665166480
C	1.38662077621357	0.37998067432181	1.24813836867585
C	0.22342894287102	1.31750797364631	-0.63653843233262
C	-0.25294324397598	-2.08391207389622	-1.19232864270224
O	2.32537435458142	0.32456695485346	-0.96047103826486
C	2.47825136983724	0.01200613755121	0.35013267509152
O	3.49483884534254	-0.54413268807164	0.70171216152931
C	-0.04800145705012	-1.99395937167334	-2.63581119745835
C	-0.74307426464585	-2.28550866947312	1.41278769645592
O	0.08267231671631	-2.66640911846522	2.20314408611514
O	-1.98958937408335	-1.93619830061097	1.69934220057794
C	-2.36738217228211	-2.04995100778977	3.08889291222139
O	-0.94984039993579	-1.83059537145163	-3.41759812130361
O	1.23471548876561	-2.11977308207620	-2.94696948977463
C	1.53935264733989	-2.05475283260880	-4.35494243951070
N	1.64036294109062	-0.00287550576887	2.56240162485903
C	1.31557789142502	1.19496927868062	-2.90720571469529
C	1.48479201785405	1.67589838121187	-5.65902626106011
C	2.55965673249909	1.25360462437726	-3.54591047837487
C	0.16706366485867	1.36773598634118	-3.67227206790799
C	0.23933872210504	1.60843029585477	-5.03828276242289
C	2.64537233619161	1.49391191200117	-4.90151960869731
O	1.66839858857774	1.90072915217978	-6.98292133336587
C	0.51455098716480	2.09552023080638	-7.79080802536995
C	0.77460476603745	0.04883954364251	3.61097948589696
O	-0.32084614273110	0.58884597145755	3.54066142649735
C	1.25753901041647	-0.59477904551528	4.87400332503962
C	2.05639213100405	-1.74003632693324	7.27918678045512
C	0.78159693285810	-0.08917585659460	6.08132421513314
C	2.12314490529933	-1.68644693740469	4.87327655610547

C	2.51819457257759	-2.25844844700738	6.07533010102698
C	1.18660187883212	-0.65458598714514	7.28133637973005
H	-0.50729795674775	1.30494298762067	1.41853714484008
H	-0.63193951926265	1.84769347994598	-1.02966546600825
H	-1.79071820921349	-1.34474384170826	3.68571430586644
H	-3.42594258396899	-1.80311465308565	3.11582699480736
H	-2.19869007599650	-3.06982334554907	3.43518535475950
H	1.17645332567896	-1.11722683164572	-4.77325935678881
H	2.62305847776169	-2.11052612423937	-4.41642154396143
H	1.07692316999185	-2.89776497624477	-4.86980663432384
H	2.53947108685075	-0.44297484324214	2.69069645790647
H	3.46681706652928	1.11326393314192	-2.97073813093381
H	-0.80989806822388	1.28779540173927	-3.21007777723784
H	-0.67624783058616	1.72532165208654	-5.60190456703842
H	3.60721148708485	1.54560308454464	-5.39911018409580
H	-0.12841989429123	1.21002093583548	-7.77916487443989
H	-0.05611989534401	2.96779725574114	-7.45752564592001
H	0.88190474896079	2.26521822445832	-8.80146752856378
H	2.36959821594076	-2.18639592365504	8.21696999432757
H	0.09669320907676	0.75059367867567	6.06612405002844
H	2.46512526595919	-2.12091869672789	3.94043888911837
H	3.18258660011597	-3.11548356102413	6.07010838336172
H	0.82268128522456	-0.25037423538934	8.21963244108400

0 imaginary frequencies

$E_{\mathrm{el}}^{\mathrm{sp}}$ (DLPNO-CCSD(T)) [Ha] = -1619.183426745904

$G - E_{\mathrm{el}}$ [Ha] = 0.35768037

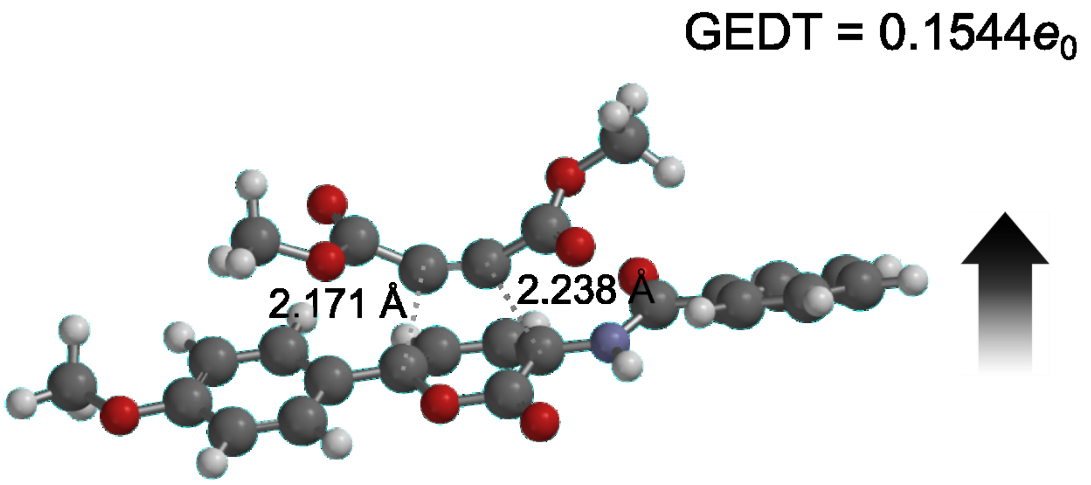

TS1

55

C	0.51877738565739	1.20119682772426	-1.21924558131510
C	-0.41134089724079	-0.96147329526752	0.30517974631031
C	-0.28625939742475	1.73517641632565	0.96053134652222
C	0.55968560907468	0.71759053530767	1.42139899196040
C	-0.32472976171905	1.95370233834423	-0.39190411405167
C	-0.54764240736213	-0.66025247722288	-0.88656511309212
O	1.69266897217945	0.72977316196629	-0.67858074850872
C	1.75565902127431	0.39626766351017	0.62576176544904

O	2.75764424028613	-0.10658277632702	1.07424209439600
C	-1.08350719322160	-0.96079867602649	-2.21005073830034
C	-0.54229492240762	-1.90399976119693	1.41228086445165
O	0.35240093694572	-2.61142067588653	1.80705719302824
O	-1.76319032021189	-1.85125253531974	1.94083660944722
C	-2.00852329688245	-2.74656223541514	3.04171356971224
O	-2.17431947100311	-0.60764570691621	-2.58619113828735
O	-0.20093515597430	-1.63223446368188	-2.94620737291470
C	-0.58902230115259	-1.87491821766409	-4.31053478895584
N	0.70729994973789	0.33114666632700	2.74727971193858
C	0.61436902873211	1.38050089289089	-2.68214810382923
C	0.76662978065357	1.68941419457774	-5.45945415553024
C	1.76542635187250	0.99813896685100	-3.38009231914672
C	-0.45607360014371	1.90039298928000	-3.40168969011774
C	-0.38985341389341	2.06243190124080	-4.77926804177851
C	1.84345638426779	1.15335259231424	-4.74779193224257
O	0.93513192636445	1.79940643634420	-6.79909431616448
C	-0.13527552943239	2.34224090740893	-7.56179936604302
C	-0.21716031267657	0.46619700483839	3.75047063176115
O	-1.26192883902713	1.07596498014950	3.60876319639411
C	0.15455350930669	-0.18655916478630	5.04689884306159
C	0.72834320518446	-1.34218522258724	7.51090367747040
C	-0.41918150349454	0.32332772074006	6.20953438413003
C	1.00758680753690	-1.28666114953641	5.12032643536264
C	1.28920667313583	-1.86387521058037	6.35164861104761
C	-0.12696115156039	-0.24744236609471	7.43886619879662
H	-0.96866348959452	2.22026462118785	1.64091076405803
H	-1.06233652652668	2.60821351760059	-0.83332527792229
H	-1.33192082953924	-2.52542835858255	3.86720745720934
H	-3.03987500661617	-2.56510140411542	3.33437621994577
H	-1.87335046907352	-3.77990460966722	2.72066154290154
H	-0.77177509438389	-0.92692707348052	-4.81719237688466
H	0.25275467266124	-2.39489944694831	-4.76175746609516
H	-1.48664792999072	-2.49320901390095	-4.34319528041585
H	1.57563338308138	-0.14784226503958	2.93819680712556
H	2.60591149338864	0.57643452024470	-2.84438656821156
H	-1.37707136662949	2.16825144293244	-2.89880143692861
H	-1.24490851735881	2.46782097922423	-5.30278671233271
H	2.73556710933980	0.86292503213423	-5.29070969863774
H	-1.03412144476457	1.72421681108347	-7.47376479118590
H	-0.36157689106247	3.36599456281843	-7.24835995750062
H	0.20494723170633	2.34489283756864	-8.59590264770445
H	0.95344348702494	-1.79258221472087	8.47171629147363
H	-1.09122510175704	1.17009690943605	6.13544864996711
H	1.42830963123375	-1.72845146712958	4.22356133889756
H	1.94427796940692	-2.72643259783611	6.40280897594824
H	-0.56801261792734	0.15930895555892	8.34221781533102

1 imaginary frequency (-459.07 cm^{-1})
$E_{\mathrm{el}}^{\mathrm{sp}}$ (DLPNO-CCSD(T)) [Ha] = -1619.147688177524
$G - E_{\mathrm{el}}$ [Ha] = 0.36486907

IM
55

C	0.96422735061668	0.68235984213071	-1.20746934074546
C	0.32630996498542	-1.00959782780143	0.37395278333121
C	0.13723501161486	1.40206792970531	0.88833515227288
C	0.87180758342857	0.11961686604609	1.26062723380110

C	0.16467147362806	1.68701620320250	-0.40085569966876
C	0.35182750161246	-0.69490931495087	-0.91672237326429
O	2.30114152774358	0.62573092153876	-0.61046573131466
C	2.30764985068214	0.30112827467366	0.69155417244469
O	3.31993568466577	0.14007680936458	1.30673770303489
C	-0.12916652622736	-1.50902299692631	-2.07401486574517
C	-0.08520788354168	-2.33585430829494	0.90438429609868
O	0.18039042177445	-2.74827942382702	2.01269742468253
O	-0.77278645478663	-3.03290865346851	0.00843436375877
C	-1.16947039801872	-4.35908147337754	0.40220065003693
O	-1.21168803280628	-1.35820069110080	-2.57742599024825
O	0.81465472455574	-2.33597640063498	-2.50881316583750
C	0.48476441468284	-3.08416725153317	-3.69406025758541
N	0.88154288888163	-0.17366925358446	2.66011996901347
C	1.12689364527251	0.98228596635390	-2.67407791043067
C	1.34458284525324	1.41129323818435	-5.42774471035391
C	2.29555030776208	0.63668143673522	-3.35435338892364
C	0.07371876074116	1.52747263066940	-3.39191451785758
C	0.16941699862439	1.74630088911871	-4.76218903539605
C	2.40743241594375	0.85306023429280	-4.71346772715024
O	1.54855796162026	1.58608567521502	-6.75802346129284
C	0.49614292084588	2.16160788012361	-7.51993539905374
C	1.36579460541933	0.71815883714441	3.56528989587220
O	1.63086394883526	1.87354142128983	3.26919376256509
C	1.51751938092761	0.20609146930857	4.96746371586303
C	1.82183458344829	-0.61995664121024	7.60792593631160
C	1.46163181460439	1.13533039852123	6.00310704610796
C	1.73962845712011	-1.13800675016008	5.25832292692598
C	1.89459677399341	-1.54793829927947	6.57635370092182
C	1.60522067131370	0.72342385803166	7.31966375292534
H	-0.30794561717542	2.00213834401864	1.66703087398663
H	-0.27869907806620	2.55502865833216	-0.86703693026847
H	-1.81086634740364	-4.31045966795372	1.28224561031584
H	-1.71373898587617	-4.76053583760161	-0.44913895259959
H	-0.28800635328432	-4.96303337361724	0.61886683408856
H	0.27956292422736	-2.40010527567940	-4.51822885258363
H	1.36104279206972	-3.69170127746872	-3.90701297055368
H	-0.38653830813472	-3.71312629052892	-3.50882222598517
H	0.72862071998182	-1.13573681367671	2.91913988247514
H	3.12625153497419	0.20084102581543	-2.81378239743339
H	-0.85667352052324	1.77624992883506	-2.89405317583332
H	-0.67365948261993	2.17319145443703	-5.28820326487975
H	3.31655001638638	0.59612455472388	-5.24517591121945
H	-0.40160353084183	1.53622163948753	-7.48831022175889
H	0.25482508833411	3.16719452153995	-7.16147187218919
H	0.86307980370171	2.21901318268061	-8.54341516723360
H	1.94047211193159	-0.94252699396431	8.63686704646667
H	1.30566354788977	2.17993746918927	5.76084006310008
H	1.82332546598269	-1.87327441072214	4.46541783706444
H	2.07864905930966	-2.59385821643117	6.79616536829935
H	1.55246296391955	1.45068288308392	8.12253151564145

0 imaginary frequencies

$E_{\mathrm{el}}^{\mathrm{sp}}$ (DLPNO-CCSD(T)) [Ha] = -1619.222498635955

$G - E_{\mathrm{el}}$ [Ha] = 0.36902135

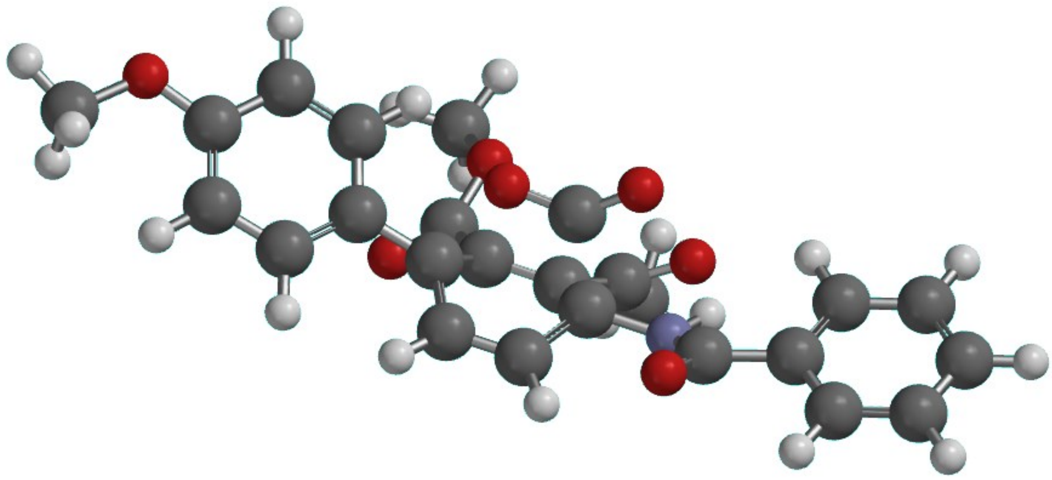

TS2

55

C	-0.39651372616089	0.98904209369331	-1.21202675457573
C	-0.61341740056803	-0.66114210554628	0.49972885671419
C	-0.92561225442340	1.72197313519403	0.96839367721846
C	-0.25675002004794	0.47726197518371	1.37713301311734
C	-0.98513472712125	1.97957935582000	-0.34565432460424
C	-0.65302847698651	-0.39596545717874	-0.81638848405821
O	1.42393501238686	1.02334149347077	-0.41743867864493
C	1.38165287208212	0.75882193287606	0.80007498082283
O	2.21461828814473	0.63213774136991	1.66371794242890
C	-0.91576650141932	-1.42441788591619	-1.87367895232568
C	-0.77990090324719	-2.04069728802313	1.04361175731300
O	-0.11699182995787	-2.49761167468507	1.94609248519705
O	-1.75347826973510	-2.69399509720625	0.42277599274886
C	-1.93690450758452	-4.06835304547186	0.81294027535263
O	-1.83168817823325	-1.35521072100767	-2.65115536375864
O	-0.00597973773103	-2.39864945410394	-1.85410580832603
C	-0.20679301916900	-3.44887663111280	-2.81630447614233
N	-0.20935349204362	0.18847327500334	2.76910190822565
C	-0.07572461329617	1.28867460672902	-2.61289142500026
C	0.51867377703710	1.78462235921054	-5.29772113570090
C	0.89029924131702	0.52598230702823	-3.28299554318097
C	-0.72506732447348	2.30167493571068	-3.30818657547456
C	-0.44620982899036	2.54910240839169	-4.64586317620851
C	1.19152367687022	0.77265019065647	-4.60242854735234
O	0.87337978743945	1.94649876055047	-6.59136766794732
C	0.22455644156973	2.96791533218778	-7.34037796505420
C	0.20198170897500	1.10142860424411	3.70110584173101
O	0.19540737780697	2.30645531440720	3.51077214183812
C	0.61591869495981	0.51763643436874	5.01931627141609
C	1.39384263163758	-0.44983748633710	7.50877841814233
C	0.38904167243377	1.27450500893151	6.16595827039134
C	1.24829546121150	-0.71915274916110	5.12081148926541
C	1.63953444362907	-1.19855426380432	6.36451340798184
C	0.76783540889880	0.78848160506994	7.40840989684447

H	-1.21061632457591	2.43345708036087	1.72736525180997
H	-1.31337663297637	2.93183979089554	-0.73661576964080
H	-2.18184508213772	-4.12556975658510	1.87348674223644
H	-2.76194987157520	-4.43225611411427	0.20561892645141
H	-1.02668855767387	-4.63434955667006	0.61194481802290
H	-0.18332643545806	-3.03928047786847	-3.82670494573792
H	0.61449368895643	-4.14506703203278	-2.66325215227934
H	-1.16638890944961	-3.93697445709702	-2.64043150847050
H	-0.03728696678064	-0.78033828188270	2.98850552649868
H	1.43124361910909	-0.24596696886576	-2.74808300741774
H	-1.48863557770336	2.89829044429965	-2.82442244654254
H	-0.98464993491406	3.33252136970198	-5.16115071595962
H	1.94981817247176	0.19795244907471	-5.12137799515642
H	-0.85267569078621	2.78613565776255	-7.39974723170310
H	0.40911182806071	3.95292426324379	-6.90148273484532
H	0.65695755583958	2.92643675278670	-8.33842451437563
H	1.69689295336478	-0.82773103036106	8.47947349462622
H	-0.08583752912906	2.24396314531809	6.06974626405379
H	1.47431706266562	-1.30135323402760	4.23374942071452
H	2.14444876789543	-2.15556933354738	6.43740817764790
H	0.57981217958585	1.37714027906525	8.29974265167245

1 imaginary frequency (-532.30 cm^{-1})
E_{el}^{sp} (DLPNO-CCSD(T)) [Ha] = -1619.196737364630
$G - E_{el}$ [Ha] = 0.36665488

P

55

C	0.69387914674612	0.74438887369184	-1.51900478024945
C	0.56253439056893	-0.78122464685400	0.36965002769660
C	0.99455131907061	1.55997525091708	0.74939953173229
C	0.83584500749961	0.27091419756696	1.26091377499722
C	0.91833900683239	1.77877957734349	-0.61168695658589
C	0.50354124279036	-0.53509551029286	-1.00729409244827
O	3.83995198236043	-0.41642944451135	-1.00141607877796
C	3.85749696794287	-0.62814568246585	0.13784208703695
O	3.88448551196178	-0.84230393447751	1.27671360003015
C	0.25527440545931	-1.66096511305764	-1.97131930133625
C	0.31023433486338	-2.15233336478869	0.90209210796081
O	0.87171075167249	-2.63386780125586	1.86477520127309
O	-0.63111541984578	-2.79432531442919	0.21734140572984
C	-0.88008362315379	-4.15655892872317	0.60247514514659
O	-0.69217646459030	-1.73594183033230	-2.70820101254651
O	1.25088991511942	-2.55611852470679	-1.93127093534073
C	1.09774122625835	-3.68361354401783	-2.80764641558117
N	0.95840581215632	-0.01060257756574	2.62586418412270
C	0.71752617253250	1.02424192719949	-2.97719380986199
C	0.77810377421418	1.58720174109792	-5.72030692255406
C	1.58426049104087	0.33479232395904	-3.82922503711905
C	-0.10203274825726	2.00251241517358	-3.52277958383762
C	-0.08391088794375	2.29020330550627	-4.88433742094228
C	1.61660792133752	0.60849478933866	-5.18241626763066
O	0.87726962124474	1.78420844655553	-7.06222488830777
C	0.03901253829124	2.76895707081285	-7.64842605545212
C	0.84282733550843	0.85711434278951	3.67569009105157
O	0.65134919119104	2.05536657188310	3.55378247555694
C	0.97702228540070	0.22252452982501	5.03162930039882
C	1.23217549831985	-0.81435387308044	7.60381645363727

C	1.36291746075898	1.04698481189464	6.08614128434760
C	0.70765976780136	-1.12336837365499	5.27413364390753
C	0.83263400143972	-1.63748255587396	6.55835960418789
C	1.49755664972567	0.52996463180723	7.36583683754214
H	1.19196808558127	2.37692372130747	1.42516898952593
H	1.07863354075499	2.78156952441940	-0.99271022042883
H	-1.22713898317625	-4.19710313756554	1.63510901629613
H	-1.65140833132379	-4.51195377089222	-0.07675119018201
H	0.03118630832172	-4.74655893037224	0.49667519149326
H	1.04699399035856	-3.34930934575276	-3.84464122778085
H	1.97752641882412	-4.30270184786190	-2.64618355975252
H	0.18852221493680	-4.23263636045057	-2.55774807380791
H	1.15553651749336	-0.97790168717251	2.83458654844543
H	2.25476628723850	-0.41522913292872	-3.42310410385704
H	-0.78585570481057	2.54603508488542	-2.87871168504600
H	-0.74551075646983	3.05277597175754	-5.27299457235863
H	2.29275280377166	0.07868151411396	-5.84402020669126
H	-1.01824398994323	2.52529710215354	-7.50270647286450
H	0.24535387628063	3.76112319016422	-7.23421023635177
H	0.26901263791804	2.76529016433463	-8.71281016454617
H	1.33218889686696	-1.21942441205720	8.60513676385591
H	1.55592709779730	2.09431656431560	5.88680538522392
H	0.37520221026901	-1.78193299380351	4.47912943826902
H	0.61257637488904	-2.68340721876619	6.74186045113558
H	1.80749591810332	1.17630221289760	8.17970273163813

0 imaginary frequencies
E_{el}^{sp} (DLPNO-CCSD(T)) [Ha] = -1619.305363980978
$G - E_{el}$ [Ha] = 0.36231322

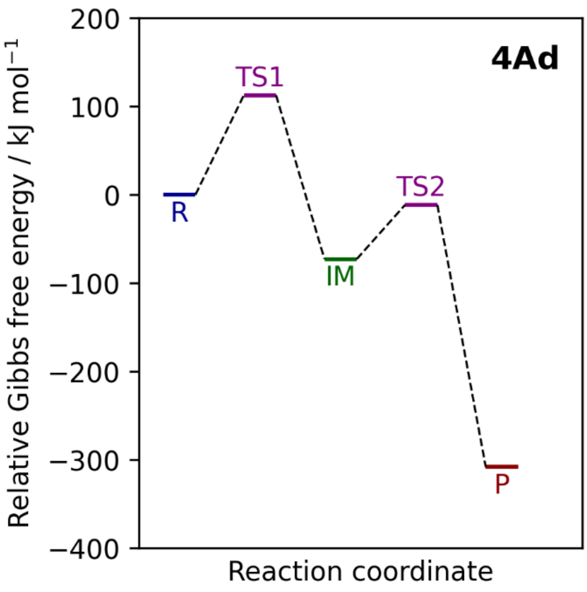

CASE 1l

R

58

C	-0.34149179003360	-0.39057812674851	-2.00910549454205
C	2.33106267591370	-0.39452725841559	0.15207829582431
C	1.96118241291494	1.95799848737940	-0.29270347269350
C	1.59513233236224	0.71999465518111	0.34341548383710
C	3.08978581799626	1.99819482251748	-1.02645181602886
C	-1.05152367105520	-0.86231202276826	-1.16872946783461
O	3.84401227931864	0.86904230553112	-1.20061511015925
C	3.51711073466470	-0.34232332451850	-0.69133747155747
O	4.21612238669324	-1.29704714347941	-0.95747751364649
C	0.51538745415796	0.18183070999519	-3.04169811706331
C	-1.89400382295909	-1.40168511535272	-0.10298309861198
O	-2.67221230446689	-0.73505153540279	0.52580935496726
O	-1.66764721114155	-2.70042401387043	0.05685885465309
C	-2.39095639371200	-3.32497233952289	1.13596434795282
O	0.17408259254745	1.07053643165073	-3.77855192755321
O	1.70131475166996	-0.41990245747965	-3.05136876991354
C	2.62408204573347	0.03022331260260	-4.06313298746313
N	2.07007002733269	-1.68020263743314	0.62804436547111
C	1.08949207797580	-2.04801980665736	1.49873541004632
O	0.32992446109048	-1.24955208378177	2.02717175810846
C	0.99472569775102	-3.51808651425170	1.77013503511050
C	0.67538328012590	-6.22147810353825	2.33481513161440
C	1.25582563982130	-4.47033598315725	0.78862825520706
C	0.56532059129270	-3.92165746854451	3.03135993404481
C	0.41565129381651	-5.27063126986388	3.31655524274026
C	1.09033684228508	-5.82019148462010	1.07064413370159
C	3.71687264278631	3.17042449901946	-1.70116882426501
C	1.07605681083481	3.13730011481797	-0.12140228914806
C	-0.63229611933481	5.33099321757264	0.22052071707639
C	0.53691702637719	3.79868872202369	-1.21652780904948
C	0.73076666795738	3.58237940867208	1.15799071836820
C	-0.10738349994685	4.66600416203391	1.33158194953256
C	-0.31033931199681	4.89214899514831	-1.05994667092406
O	-1.44765500343337	6.38479942938883	0.48879209444344
C	-2.02346339483933	7.07157625074874	-0.61294158748316
H	0.70231605564650	0.68638129494435	0.94937450076785
H	-2.03771229708978	-4.35309504686105	1.16122995357505
H	-3.46234219330040	-3.28411856936777	0.93671433016476
H	-2.16241376679488	-2.81581431497516	2.07154541854898
H	2.24049178627294	-0.23073872877016	-5.05002146466290
H	2.76148658444544	1.10844619938856	-3.99456439042207
H	3.55445161329810	-0.49231979378851	-3.85547905857591
H	2.73213465223866	-2.37455834523060	0.31347266485686
H	0.55051175311855	-7.27638744763335	2.55452797438107
H	1.55373075956098	-4.16476540628039	-0.20860775719928
H	0.34769546250525	-3.16836119813801	3.77977850030352
H	0.09119936201108	-5.58217004871304	4.30343829636851
H	1.28001396330966	-6.55851211841954	0.29934508328046
H	3.23674791307659	4.09477898855260	-1.38731427128371
H	4.77861468617206	3.20988458380200	-1.44593279560262
H	3.63847215115255	3.08625784653577	-2.78853803828458
H	0.76322492843447	3.44916098209698	-2.21831854636263
H	1.13108767212402	3.07409612871731	2.02956473668528
H	-0.37118066958640	5.01638768093657	2.32321138310559

H	-0.71533081312555	5.37649590733677	-1.93836866330087
H	-2.63843482224747	7.86248316229201	-0.18630264569376
H	-2.65131650165008	6.40268610742049	-1.21012652016951
H	-1.25110430007277	7.51366530127763	-1.25055734524270

0 imaginary frequencies

E_{el}^{sp} (DLPNO-CCSD(T)) [Ha] = -1658.426022861175

$G - E_{el}$ [Ha] = 0.39011383

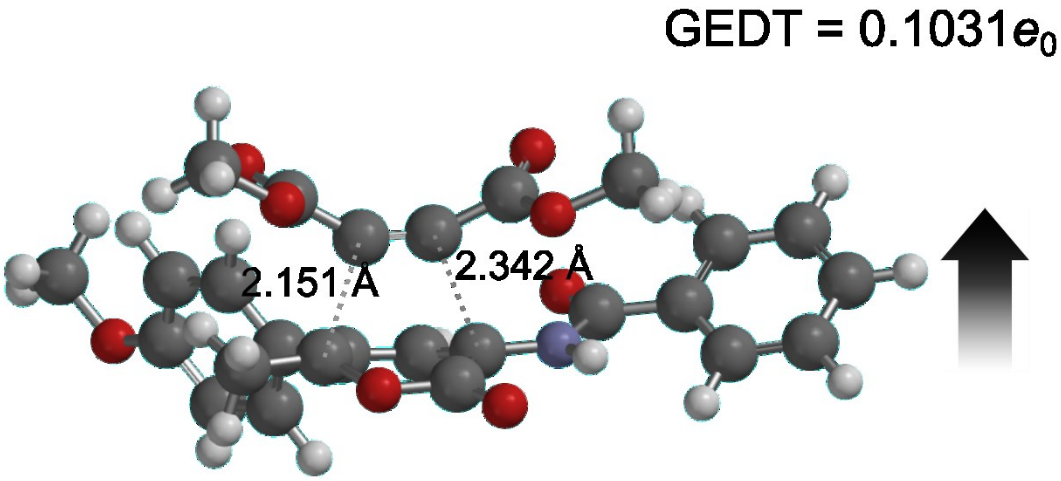

TS1

58

C	0.07419905232173	-0.07022182751997	-2.01704167064368
C	1.09024120686531	-0.81964570868898	0.67983082350890
C	0.91449127546047	1.46544154048026	0.03135326844131
C	0.66427521416302	0.47387599910180	0.96132937121381
C	1.63459728688680	1.10419410092259	-1.11519578688188
C	-0.13829118406797	-1.05235886699647	-1.30014066230821
O	2.50443029693898	0.03545612346404	-1.03000698793665
C	2.23062891976714	-1.00742709895176	-0.22604276422361
O	2.92373314513325	-1.99851785325466	-0.25286247612820
C	-0.21801384592658	0.61427340088645	-3.27744615472215
C	-0.76782886078989	-2.31202291643470	-0.93270916925062
O	-1.85615820394594	-2.39616513716008	-0.41952298500203
O	0.02652530566944	-3.34832807163403	-1.20553617935290
C	-0.50129511941315	-4.64012603268312	-0.84858824867059
O	-1.01378587380950	1.51298337771769	-3.40005688704227
O	0.52399311989678	0.12110960039801	-4.26851153668436
C	0.32786771813765	0.73999692953270	-5.55400015442260
N	0.84607307314990	-1.94328371105982	1.45227791552193
C	-0.19182592674549	-2.12256879557807	2.33632117678880
O	-0.97164862210568	-1.23765645262533	2.62833095793880
C	-0.30037973788874	-3.50822458335383	2.89512128943964
C	-0.63731813922241	-6.06932975839459	3.92377975333286
C	0.80152795951538	-4.34557791408066	3.05692139204654
C	-1.56857941009978	-3.95293432747029	3.25935412735461
C	-1.73759613096728	-5.23340370848503	3.76462269982820
C	0.63179991698790	-5.62228035404864	3.57620579434315

C	2.12243604772911	2.07809404058747	-2.14298732735843
C	0.35254917840468	2.82741638185570	0.22221502616612
C	-0.69090330657363	5.38376880874336	0.68766150290546
C	-0.58514101463703	3.37884603280540	-0.64037719739073
C	0.75462425228370	3.57205993709610	1.33453892386116
C	0.24418308408640	4.83473342550879	1.56710412736267
C	-1.10774577064009	4.64943938892745	-0.41866887166053
O	-1.13741502221922	6.63001978828293	0.99488262202831
C	-2.09794269813433	7.21957755811608	0.13034553609371
H	0.03270422364451	0.66135359910227	1.81621479542961
H	0.27969908031308	-5.35113678745228	-1.10757312810446
H	-1.41056684645171	-4.84215831629951	-1.41557823229875
H	-0.71926751395456	-4.67706326460755	0.21954363382565
H	-0.70529695527212	0.61268887899875	-5.87804020324943
H	0.56773891342941	1.80248220436271	-5.49809986447762
H	1.00891893775220	0.22607980181171	-6.22816957230268
H	1.41221563112987	-2.73967248952825	1.19753667219477
H	-0.76846342938177	-7.06878382227492	4.32442476063569
H	1.80151089202826	-4.00532076524899	2.80879211445491
H	-2.41412744370776	-3.28800095234643	3.13020498054586
H	-2.72849591734920	-5.58015338684676	4.03638767511149
H	1.49309471902794	-6.26699062803196	3.71186195571093
H	2.97401980409939	2.62984495139135	-1.73530069988414
H	2.45138644389750	1.54623770364393	-3.03550500025384
H	1.34641696069115	2.79310639322048	-2.40643268103945
H	-0.93554493124410	2.81246794823677	-1.49531269662846
H	1.48224443324666	3.15521149768631	2.02332663841856
H	0.55873215209571	5.41518446059495	2.42707168445861
H	-1.84117815031414	5.04295937834273	-1.10977280506803
H	-2.31821677363451	8.19995616095989	0.54989645803808
H	-3.01519921402212	6.62339824391895	0.09413492039857
H	-1.69863220223465	7.33709587435902	-0.88211265441244

1 imaginary frequency (-432.62 cm^{-1})
E_{el}^{sp} (DLPNO-CCSD(T)) [Ha] = -1658.389478707232
$G - E_{el}$ [Ha] = 0.39069816

IM
58

C	1.25180537615687	0.27823902219175	-1.96323765517643
C	1.60955989024505	-0.59055655140188	0.22821980814477
C	1.35580528799523	1.78377940431139	-0.02607996046322
C	1.13388426608279	0.75010038012775	0.77222578561877
C	2.09280940152212	1.36202071185335	-1.30114314711326
C	1.01569427216271	-0.76517085731864	-1.17937901465020
O	3.32135730145048	0.66858905750280	-0.84987032716581
C	3.10500128862251	-0.36316099028302	-0.04184818859404
O	3.98853537261410	-1.03503054808667	0.42040715583901
C	0.71195226829547	0.46657915113091	-3.34043084779669
C	0.22931112708374	-1.94689843368588	-1.63338756540394
O	-0.74919893773453	-1.85757043751019	-2.33135542585771
O	0.75302187635138	-3.09569601312649	-1.20955431960152
C	0.00930930850660	-4.27843301091356	-1.55039733060378
O	-0.00149723106652	1.39333119862345	-3.63634406894963
O	1.12828114363402	-0.47326632438142	-4.18179700152439
C	0.56235158109865	-0.41589631230300	-5.50399428668864
N	1.46645943509889	-1.67761597639962	1.14553657688547
C	0.23980369520635	-2.15529067097041	1.46803182588177

O	-0.77988370375151	-1.62739736665210	1.04359793851239
C	0.20654225253018	-3.36351098922344	2.35071477395864
C	0.03409831690812	-5.63445989923692	3.94748750667511
C	1.26046682967072	-3.70506114109765	3.19532536383924
C	-0.93716592781151	-4.15713216935737	2.31625588340044
C	-1.02063585901754	-5.29340189985073	3.10739402362771
C	1.17145336795009	-4.83703302257053	3.99453057705306
C	2.58368382928537	2.46639976342576	-2.20382745432649
C	0.98380391600280	3.18621020133903	0.25117535395216
C	0.23586404546319	5.82325278640660	0.82018029830386
C	0.13222424307309	3.88394516828260	-0.59822209162712
C	1.45578524563816	3.83023236177367	1.39714595538701
C	1.08974001054937	5.13258269779022	1.68152155767446
C	-0.24670097739523	5.19313273738735	-0.32463181479879
O	-0.06866398718736	7.09798412869440	1.17864249296118
C	-0.93917308487309	7.83383009570529	0.33092623327872
H	0.66934005559387	0.78595002428580	1.74798483849160
H	0.54968313589824	-5.10349859570976	-1.09193510352530
H	-0.03277798011200	-4.39779738435244	-2.63340075786406
H	-1.00155302879688	-4.20839616044278	-1.14709392317336
H	-0.52058311061030	-0.53016923751779	-5.44498708148574
H	0.80975322766534	0.53372967696387	-5.97913404003035
H	1.00831586430730	-1.24597727288392	-6.04676667632347
H	2.29859207748130	-2.22016182443528	1.31707940361413
H	-0.03276476242697	-6.51992940465893	4.57053132017021
H	2.14491318156762	-3.08048112422806	3.25766644901609
H	-1.75387298471744	-3.87022929672357	1.66439342832037
H	-1.91040639019486	-5.91247743575753	3.07233095855434
H	1.98970411106001	-5.09365392381555	4.65828813361637
H	3.21264438802302	3.15090543096020	-1.63398721487046
H	3.16855530075188	2.03786676171066	-3.02012020927488
H	1.74717215290215	3.02086553783593	-2.62277418718886
H	-0.26463999912563	3.39615132611342	-1.48271062707249
H	2.12465887014602	3.30154055188217	2.06791857463687
H	1.45760300804665	5.63632267594198	2.56829276607450
H	-0.91855735888443	5.70093911172524	-1.00354194544328
H	-1.05101345531704	8.81359038681532	0.79266359177062
H	-1.91894379696817	7.35151793132090	0.25666935345503
H	-0.51151174665048	7.94880199279327	-0.67013766212002

0 imaginary frequencies
E_{el}^{sp} (DLPNO-CCSD(T)) [Ha] = -1658.477140873292
$G - E_{el}$ [Ha] = 0.39627642

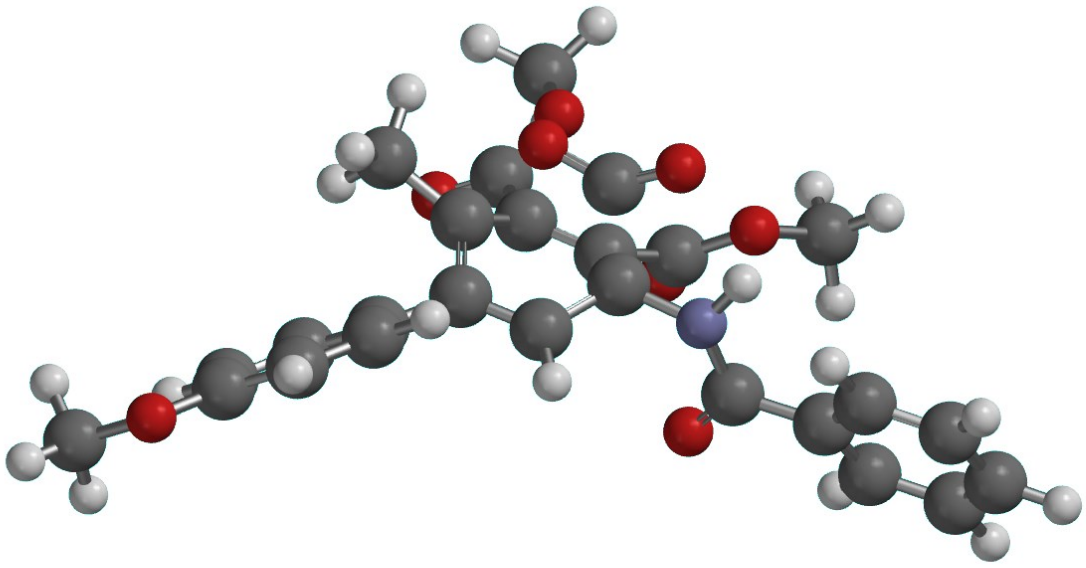

TS2

58

C	0.19684725353874	0.05673324574745	-1.91436119281777
C	0.60136260197915	-0.71339842904580	0.30830167049623
C	0.29063109045171	1.65382063022753	-0.03844038347715
C	0.22223353643476	0.61106721874665	0.80811326363159
C	0.71085429932400	1.30123539139005	-1.37745095485828
C	0.10969762199029	-0.97714444510706	-1.06097433916998
O	2.50951411006335	0.44884472881768	-0.79652581884115
C	2.29269188690061	-0.47730473361790	-0.00462023785578
O	2.99637026918551	-1.25810810401160	0.60485145394893
C	-0.10121614236196	-0.03575356342298	-3.37912887699520
C	-0.36986452631149	-2.31629584304422	-1.52679728399001
O	-1.33058645407317	-2.44351654096202	-2.24175538306213
O	0.38268080979575	-3.31216642021167	-1.07432399341678
C	-0.05664217000156	-4.63550710364728	-1.43177383837689
O	-0.84364171419582	0.72376354412031	-3.94935323131503
O	0.57153802887195	-1.02256746215595	-3.96376049858916
C	0.25829149742805	-1.25213437986357	-5.34922842042649
N	0.51222139219288	-1.76302517421465	1.26981100959887
C	-0.67603084701018	-2.35449268412866	1.54357800335921
O	-1.72214083834080	-1.94499206981669	1.05642269326307
C	-0.63375127372777	-3.54120870736112	2.45589463775047
C	-0.66377911071426	-5.78360214902895	4.10161475832162
C	0.41389400187421	-3.76843482162744	3.34559865179928
C	-1.69955109501956	-4.43558546985888	2.39965890159764
C	-1.71147837110229	-5.55758071125762	3.21522391069538
C	0.39556599999866	-4.88641137669715	4.16914999487835
C	1.25814020472794	2.33041327912605	-2.30218987139056
C	0.12396234021197	3.06453524042047	0.37818074483211
C	-0.22529675489891	5.71785520400650	1.19854864615910
C	-0.74077895455857	3.91298582033973	-0.30286897665663
C	0.81587852806026	3.56637748362155	1.48338677109147

C	0.64657322641272	4.87531795031224	1.89154135494936
C	-0.92370809194298	5.23198238044575	0.09529722410414
O	-0.32737088552132	6.98603506418811	1.67089609247510
C	-1.20381668686351	7.87751575939884	0.99519363457261
H	0.04232445120162	0.72212591935469	1.87067064346957
H	0.65416830208282	-5.31239658488331	-0.96393145946062
H	-0.04833865317765	-4.75306067756981	-2.51566299259006
H	-1.06309967101613	-4.80619679985274	-1.04813993110525
H	-0.79595723794650	-1.51312572255538	-5.44886255226961
H	0.47626172795635	-0.36042739392637	-5.93745614285482
H	0.89155776493078	-2.08094824715916	-5.65669337404097
H	1.39069162669025	-2.22132304395229	1.46817344974236
H	-0.67470958741162	-6.65806307843025	4.74345953710119
H	1.23713644000883	-3.06665933472187	3.42181700879584
H	-2.51239175263068	-4.23803361471193	1.71077000143811
H	-2.54041591346667	-6.25498761761375	3.16344969996132
H	1.20813841802967	-5.05431052604364	4.86740924857321
H	1.84456244327345	3.06403891594992	-1.75162164756929
H	1.87328232580394	1.85993608697438	-3.06959366829559
H	0.42931454759943	2.84282761483724	-2.79677080894251
H	-1.30250521161331	3.53960817623605	-1.15300224776914
H	1.50498080593599	2.92293750885681	2.02021070547898
H	1.18575084896635	5.26940767796391	2.74550595429993
H	-1.61193715324227	5.85988713345554	-0.45434452085599
H	-1.13373453419002	8.82617122676132	1.52485022502251
H	-2.23640740167841	7.51612851611684	1.02846029664226
H	-0.89796736890457	8.01721111308607	-0.04640754105699

1 imaginary frequency (-443.13 cm^{-1})
$E_{\mathrm{el}}^{\mathrm{sp}}$ (DLPNO-CCSD(T)) [Ha] = -1658.447885077129
$G - E_{\mathrm{el}}$ [Ha] = 0.39246922

P
58

C	0.63757870118538	0.25770915729102	-2.14631445623350
C	0.47984838797371	-0.51428382286726	0.11992404902526
C	0.65863993206278	1.86027053430858	-0.36782640878302
C	0.53834918941461	0.80939067672794	0.53283589698145
C	0.70852308063107	1.59082458232900	-1.74345051642339
C	0.54413845153997	-0.80142764979267	-1.24207129895134
O	3.97349051640296	1.34738261389472	-2.47726566201447
C	3.88549913940416	0.41570960176696	-1.79288967844362
O	3.81224138243411	-0.51059174598688	-1.10047144658034
C	0.72826922050353	-0.04108863512390	-3.61613886849966
C	0.52774439396044	-2.19396161201793	-1.79188008572170
O	-0.08943797979374	-2.51426598893196	-2.77741058244011
O	1.31864684202850	-3.02073379137809	-1.10686229382886
C	1.27720416517648	-4.39473356159298	-1.52153719898580
O	-0.09300792037993	0.27963262925998	-4.43467358050632
O	1.87759670136638	-0.66358542174142	-3.90551544451078
C	2.03297071634284	-1.07136121289588	-5.27419042843645
N	0.36515901291916	-1.50662934936927	1.11689969680614
C	-0.51027135351626	-2.55409903927676	1.08179007194709
O	-1.29365575761144	-2.73232210342708	0.16498406049826
C	-0.44014909953965	-3.48116049089952	2.25669054558311
C	-0.39760685159226	-5.26188046417125	4.39267265048668
C	0.74159688693360	-3.69810196021093	2.96156791744338
C	-1.59631870628503	-4.17015794183791	2.61457214418847

C	-1.57718351023106	-5.05108592050387	3.68548383501430
C	0.76222617937393	-4.59032936082732	4.02618282297919
C	0.89043793065717	2.68890276500027	-2.76227280893993
C	0.71522971326138	3.24279228262884	0.17646532083927
C	0.79385995802538	5.82285651028199	1.27192102056021
C	-0.19069084849169	4.21725576420782	-0.22297613500728
C	1.66374207781974	3.58243064342936	1.14475121406722
C	1.71001429741752	4.85366100770682	1.68478440134768
C	-0.16380337244747	5.50000241636690	0.31427436211036
O	0.91302726247650	7.04330303004269	1.85783968903686
C	0.00088426797796	8.05822554672229	1.46328329552184
H	0.46125278980253	1.02933738762192	1.59257055577786
H	1.94547903320939	-4.92399436519265	-0.84543947188290
H	1.61944947052155	-4.49056202557584	-2.55257620992891
H	0.25916693483805	-4.77636086110473	-1.43397494069612
H	1.23862311548462	-1.76933155812171	-5.54204731657128
H	1.99929604910845	-0.20385126414444	-5.93408415441521
H	3.00528575073346	-1.55638790372256	-5.32735166459829
H	0.80432838459821	-1.30107599574868	1.99889622649533
H	-0.38191559941240	-5.95597783924143	5.22613888438223
H	1.65775897435312	-3.19876468615690	2.66476476248953
H	-2.50375197679114	-4.00466161257093	2.04561348074495
H	-2.48179454157151	-5.57815609000304	3.96833628078012
H	1.68699945813719	-4.76568480911972	4.56488310885627
H	1.38195726798264	3.55228881224104	-2.31592984726520
H	1.49862838074467	2.35027315487715	-3.60150647689883
H	-0.07122528270157	3.01436497960116	-3.16647316902862
H	-0.94753742699413	3.97483027426998	-0.96113551129574
H	2.38411387045856	2.83895939803913	1.47085739157758
H	2.45216263031665	5.11914866764465	2.42933968266565
H	-0.89197134232301	6.22778388052676	-0.01805738660017
H	0.26758067602725	8.94274617044545	2.03959066702965
H	-1.03094739436865	7.77209069591574	1.69073655403493
H	0.09223777044521	8.27747590040733	0.39471245421703

0 imaginary frequencies
$E_{\mathrm{el}}^{\mathrm{sp}}$ (DLPNO-CCSD(T)) [Ha] = -1658.544643961818
$G - E_{\mathrm{el}}$ [Ha] = 0.38872765

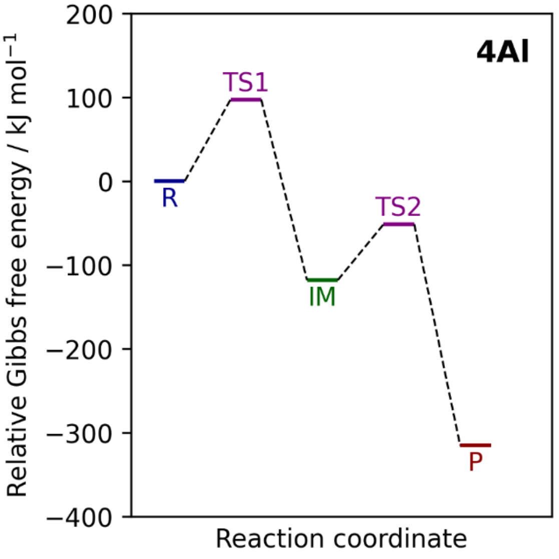

CASE 1p

R

49

C	2.46305076938533	1.32946541321887	-2.96100781250137
C	0.73167315558625	-2.56258687396342	-0.69342398406980
C	1.04541428380844	1.63431823473009	-1.04233487469951
C	1.73444196653176	0.70910226986816	-0.34582544454762
C	1.39428420690309	1.94549451696140	-2.40427100329964
C	0.08065377848962	-1.85575242078476	-1.40803492338372
O	3.17615191910345	0.42954342336044	-2.23416981376981
C	2.87213700546995	0.04641786509409	-0.96392191941265
O	3.55504504752347	-0.80026840363685	-0.43554523603886
C	-0.64132136804855	-0.94061739876982	-2.28600348610071
C	1.52734113030211	-3.44982728531919	0.14993494236218
O	1.95976887544265	-4.50708233259322	-0.22653722158811
O	1.68586913630275	-2.93223748680056	1.36396970014551
C	2.47070410078171	-3.72455780597983	2.27733922408701
O	-0.20420166781304	-0.56573774292468	-3.34537500089621
O	-1.79983843978790	-0.58518629292616	-1.75211748309191
C	-2.57050459310299	0.37295774853406	-2.50550588090649
N	1.49517035221572	0.26405200494891	0.95313263396937
C	3.05899934766610	1.45682383236552	-4.32523525374927
C	0.31460048667579	0.37397837975362	1.63550463088738
O	-0.65756497809030	0.96373113802321	1.19714388897195
C	0.30450966459968	-0.29906128196011	2.97414818435111
C	0.18053080651392	-1.55685914138921	5.45077605128854
C	-0.90246490021737	-0.81902671515503	3.43251734457815
C	1.44705415395112	-0.39781258959038	3.76388435555832
C	1.38205529455750	-1.02046405450254	5.00376815057505
C	-0.96184140762487	-1.45606205287981	4.66330060253327
C	0.47983325016239	2.90165843793435	-3.10218330909338
C	0.44073960280981	2.99638553901794	-4.60644962907451
O	-0.28463295656630	3.57226598593707	-2.43440729214023
H	0.19824501071687	2.14025373988731	-0.60628125017672
H	3.48840389796234	-3.82392854826506	1.89872272580290
H	2.02033540224481	-4.70994734010199	2.39724454642424
H	2.45568809577088	-3.17674487450741	3.21617485044454
H	-2.76768562212707	-0.00918676578549	-3.50721159971794
H	-3.49547279753829	0.49680386589648	-1.94822166552260
H	-2.03017048300522	1.31804305094100	-2.56219065522165
H	2.16521466512305	-0.41689766346632	1.28001715383576
H	3.04463075996368	2.48308821197459	-4.68245960845044
H	2.51482408106708	0.82885874859730	-5.03572477401284
H	4.09141456076952	1.11035637725995	-4.28723801175229
H	0.13186857790370	-2.04738231294050	6.41707354260863
H	-1.78605582142859	-0.72270750085980	2.81235188289465
H	2.38554087494242	0.03108851430254	3.42916748345459
H	2.27037693212459	-1.08230035470223	5.62302743078259
H	-1.90102503335845	-1.87131320577414	5.01211321070329
H	-0.49553056258417	3.47720742874965	-4.88701923611013
H	0.51556890233171	2.01563455833993	-5.07634227836242
H	1.26619053558971	3.61568915988204	-4.96558388856811

0 imaginary frequencies

$E_{\mathrm{el}}^{\mathrm{sp}}$ (DLPNO-CCSD(T)) [Ha] = -1465.850823106959

$G - E_{\mathrm{el}}$ [Ha] = 0.31409766

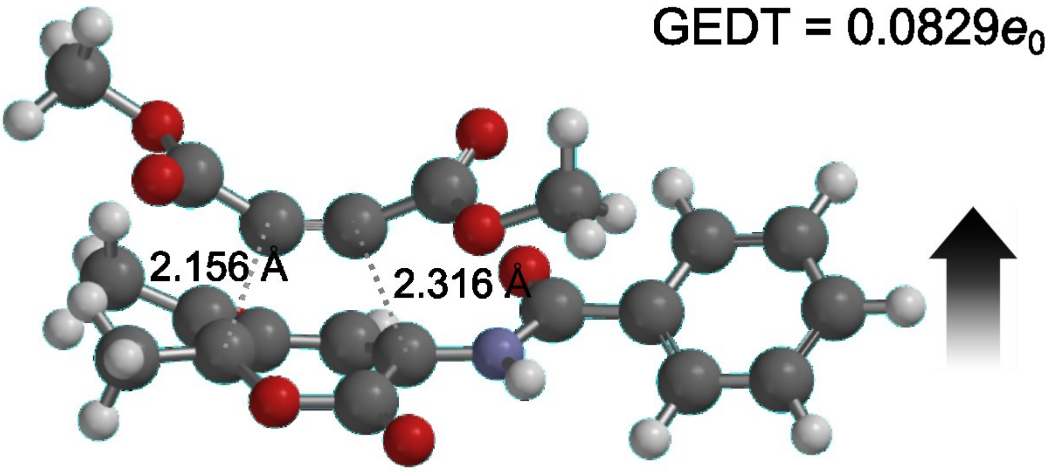

TS1

49

C	1.06076350640452	1.17044849331112	-2.34183102612869
C	-0.13455655322185	-0.89380741238398	-0.88563322421575
C	0.15319237930733	1.83555725314184	-0.24468438820799
C	0.88322407844390	0.79913073594106	0.32286814630017
C	0.21633725447563	2.02636762286309	-1.61394594160831
C	-0.13833039484131	-0.60252429729297	-2.08446596764908
O	2.15354250854731	0.65410109765659	-1.67995101438868
C	2.10242611313233	0.35861945337970	-0.36442625110601
O	3.02942766136642	-0.19961472369505	0.16971025694560
C	-0.51225288848506	-1.00941313626453	-3.43982952418752
C	-0.44391773718507	-1.74001871407191	0.26344630043793
O	-1.51681110181575	-1.75970999485095	0.81106925437697
O	0.61637732117531	-2.45869443999991	0.62814564798873
C	0.42190480872193	-3.31237960894355	1.77283251066495
O	0.14137014168799	-1.75702992017612	-4.12145785726504
O	-1.65463810022285	-0.43396895282036	-3.81927911393123
C	-2.11179185529379	-0.78118513865720	-5.14086406724508
N	0.86093653288342	0.43020880007288	1.65842703622393
C	1.38846451445601	1.23981281207472	-3.80401495223770
C	-0.17898289494458	0.62928658514812	2.53826977082948
O	-1.18018292333604	1.24979316040780	2.24194814585269
C	0.00967618564720	-0.00212902863201	3.88259039300909
C	0.22873305364314	-1.19593491173549	6.38355592828529
C	-1.13633488281189	-0.37296766518653	4.58141026850768
C	1.26626783823200	-0.22054069856428	4.44354638662129
C	1.37343739992699	-0.81242736230139	5.69513699721770
C	-1.02630745240223	-0.97588272580084	5.82545399755326
C	-0.70301273100418	3.07629852148870	-2.17985996900728
C	-1.14589192986045	3.05392599228306	-3.61827540233793
O	-1.08784531076298	3.95139145156647	-1.43312045887533
H	-0.54309911299275	2.40611807612561	0.34923340213484
H	-0.35936444728759	-4.04371246774865	1.56466213329463
H	0.14623208573508	-2.71601430259105	2.64348688872064
H	1.37883879451547	-3.80419602988227	1.92962560871409
H	-2.29078913189282	-1.85460388863036	-5.20433466615601

H	-3.03774201857901	-0.22823069641326	-5.28043618687289
H	-1.36925245505686	-0.48767460000991	-5.88387139279013
H	1.62716188065370	-0.16725848717496	1.93263528750985
H	1.97298065024163	2.14188009415777	-4.00267718729049
H	0.50013168636700	1.25880277587507	-4.42758488766163
H	1.98865197043872	0.37110978726987	-4.07099258172813
H	0.31451287373915	-1.66238805757546	7.35910108387573
H	-2.10604163833044	-0.19034586250293	4.13380799261683
H	2.16790664916693	0.09298775532464	3.92793819949624
H	2.35231932352561	-0.96923690672006	6.13448672643362
H	-1.92028303478067	-1.27336662127949	6.36233853382173
H	-1.95679899033378	3.77181143261848	-3.73228176481935
H	-1.48110266582914	2.05873542438827	-3.91272847666002
H	-0.31948696116361	3.34886932681062	-4.26918059506266

1 imaginary frequency (-456.03 cm^{-1})
E_{el}^{sp} (DLPNO-CCSD(T)) [Ha] = -1465.814229071406
$G - E_{el}$ [Ha] =0.31833224

IM
49

C	1.52682645048603	0.93534819313224	-2.38838854342169
C	0.75937428827680	-0.86895437060153	-1.00390236380838
C	0.47966714359528	1.48499289614533	-0.33980092979409
C	1.19420639613649	0.19385070894753	0.01887571694361
C	0.61671268519153	1.87535006958252	-1.60085518956973
C	0.91102044980293	-0.45553518326858	-2.25660068140751
O	2.79063813180289	0.87316713496300	-1.64046184216241
C	2.67639627335655	0.44198549485264	-0.37876467466639
O	3.61889691609670	0.24726978594802	0.32969672829287
C	0.60283938161642	-1.25334215428870	-3.48428135371815
C	0.24974073970876	-2.24137572630587	-0.72766044446889
O	-0.42765256193401	-2.85426452793049	-1.51362990726839
O	0.64420817690058	-2.72828567510936	0.45155666993601
C	0.20341247340327	-4.06623059866364	0.76068130001763
O	1.35897510419945	-2.05448026101176	-3.96687514004879
O	-0.57073968986680	-0.89524690897198	-3.99761071228850
C	-0.96232500615222	-1.59496062393925	-5.19506159780569
N	1.06288166094792	-0.18036512489493	1.39270165077772
C	1.93839398656772	1.31929465206827	-3.79112938749330
C	1.44277501865759	0.67204894328262	2.38708825628527
O	1.68380672918164	1.84962195143887	2.17875899335445
C	1.50424840530968	0.07595469903567	3.76097204986082
C	1.63396982083679	-0.91995592791292	6.35531293951113
C	1.26384825282405	0.91590667721721	4.84498030465831
C	1.82488251121814	-1.26160335577332	3.98068755354967
C	1.89301718475630	-1.75659551363253	5.27657327609249
C	1.31968720715146	0.41752542718996	6.13818350444876
C	-0.13904371302414	3.06540913706939	-2.10452654190804
C	-0.67331635362976	3.06271246797257	-3.51505813485666
O	-0.36863987370977	3.98555643812840	-1.34904732210985
H	-0.09315140129146	2.01225331766417	0.40795064822965
H	0.59525067330669	-4.76334546590368	0.02048813525208
H	-0.88568057902785	-4.10588423902719	0.76930496503528
H	0.60691495295340	-4.28173656332364	1.74712975048200
H	-1.04099036056378	-2.66242837814730	-4.98824809209069

H	-1.93067951144068	-1.18286711608593	-5.46850764729594
H	-0.23077471201359	-1.42383477281855	-5.98537135764303
H	0.95585852865861	-1.15861476947225	1.59558798112258
H	2.34698559023618	2.33020721730839	-3.79580119054280
H	1.09409201864539	1.27015455880545	-4.47637461598772
H	2.70816043480944	0.62713334811489	-4.13491365293865
H	1.68498155300856	-1.30823962276479	7.36689680820998
H	1.03416581096977	1.95871640057347	4.65995523998715
H	2.05975308375449	-1.91941745953881	3.15048291882993
H	2.15734298895342	-2.79502260150309	5.44384177495316
H	1.12277392472641	1.07345397018650	6.97907112812093
H	-1.50795768894004	3.76082657005219	-3.56936877831291
H	-0.99221212823045	2.06428382393159	-3.82100338198806
H	0.10645963177717	3.39923405727921	-4.20284281035520

0 imaginary frequencies
E_{el}^{sp} (DLPNO-CCSD(T)) [Ha] = -1465.893450408045
$G - E_{el}$ [Ha] = 0.32307387

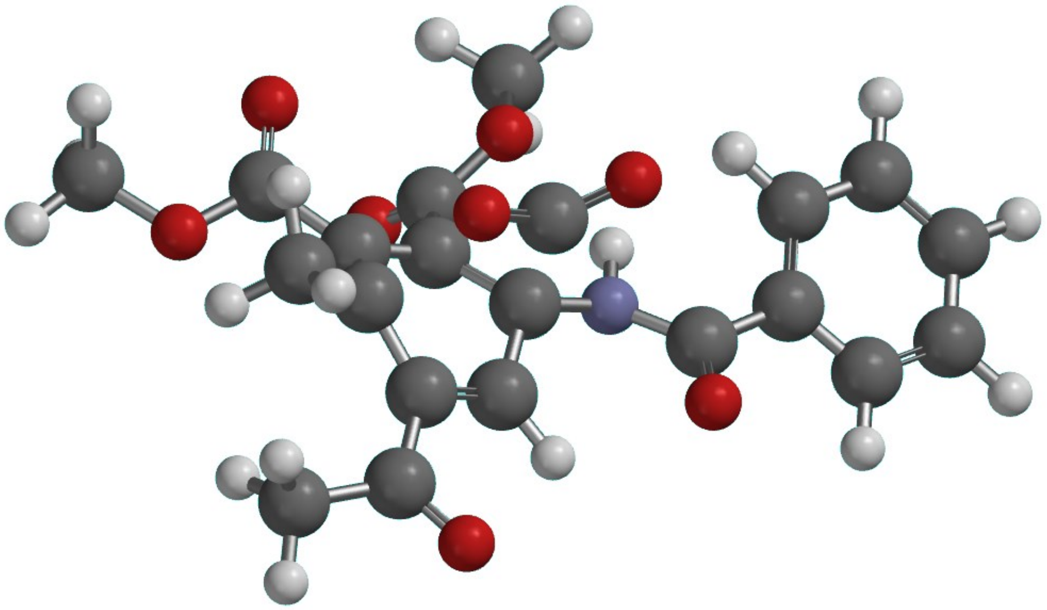

TS2
49

C	0.18050701116307	1.07378468802990	-2.31822301217020
C	-0.42445138833195	-0.57119991465044	-0.71114238671646
C	-0.45983974189311	1.80674889875438	-0.16655121978460
C	-0.03704882779937	0.47449423132765	0.24721239894194
C	-0.36028782044191	2.12257894984943	-1.47365391384579
C	-0.30897866913403	-0.26352247095932	-2.01541013484318
O	1.88647784308382	0.86734062291311	-1.37596962937793
C	1.71480883735774	0.56233543350682	-0.18455620761483
O	2.41804002640181	0.29367164218895	0.75140183600917
C	-0.47326828743585	-1.31130547887984	-3.06993884131566
C	-0.85871753907967	-1.93663357819626	-0.26833632695044

O	-1.92394863472172	-2.41013855462674	-0.55820579652040
O	0.06146042067112	-2.52163914589206	0.49695162027353
C	-0.26218062546579	-3.84999449882977	0.96060216132090
O	-0.06569295894035	-2.44038657276770	-2.96059643606880
O	-1.13138608945912	-0.84723957435680	-4.13051746639188
C	-1.32348316756273	-1.78841847360928	-5.20356120636612
N	-0.14047422422131	0.13260163680429	1.62086042467646
C	0.77269588982231	1.35675832403130	-3.66027430498832
C	0.29887785910721	0.95665519222284	2.62769642256554
O	0.42863636320579	2.16009108401585	2.49812714294330
C	0.56012687874105	0.26700546017642	3.93239560849433
C	1.06451175764706	-0.91010283912236	6.40021378150779
C	0.26280730362896	0.95334410743303	5.10660718592245
C	1.12763087008853	-1.00336495256153	3.99598340176863
C	1.38298428467489	-1.58795150553111	5.22986192495635
C	0.50474320022682	0.36191885180172	6.33765216291468
C	-0.72272931833388	3.51560648639153	-1.90577227953342
C	-1.15435579770678	3.77127773025532	-3.32858936022706
O	-0.73112165660865	4.40731286873320	-1.08493869705468
H	-0.73534664530870	2.53853031784924	0.57706325052082
H	-0.40376338991782	-4.51052435255944	0.10553791191092
H	-1.16867785781900	-3.82236949441720	1.56547616373480
H	0.59242057888527	-4.16325235800828	1.55504136526214
H	-1.90542443139007	-2.64018048768186	-4.85123246358650
H	-1.86427632082839	-1.24421994783757	-5.97419280284285
H	-0.35796653216370	-2.13129961907535	-5.57695634040666
H	-0.07177465899017	-0.85197673875371	1.81761845701340
H	1.37211198673741	2.26486792673038	-3.62162139807771
H	-0.01636797407946	1.47381281002016	-4.40292385730829
H	1.40860111244355	0.52554490965594	-3.96489889134614
H	1.26117327020552	-1.36938462163830	7.36306146493697
H	-0.15840630556084	1.94999608388790	5.04218858692809
H	1.41436255711212	-1.52565436886199	3.08905660294706
H	1.84017093959969	-2.57027395701667	5.27660984368563
H	0.26141616896414	0.89465946565273	7.25039760682732
H	-1.76066253021160	4.67615891391938	-3.34520129646224
H	-1.71820108864941	2.93075698321261	-3.73677838793601
H	-0.27573267771249	3.93317988646950	-3.95757466832604

1 imaginary frequency (-589.54 cm^{-1})

$E_{\mathrm{el}}^{\mathrm{sp}}$ (DLPNO-CCSD(T)) [Ha] = -1465.864192631434

$G - E_{\mathrm{el}}$ [Ha] = 0.31971943

P

49

C	0.63033450614739	1.05311509559035	-3.01105881714995
C	0.64890211734738	-0.60489853795254	-1.21565538538889
C	0.50549430878397	1.72240541751324	-0.67737815654812
C	0.56005255561575	0.40074649275691	-0.25160792885937
C	0.56000884516718	2.05018927901629	-2.02440864837214
C	0.67603983965347	-0.26838669975819	-2.57121190324300
O	3.87301568550187	0.51214667395171	-1.50560928566858
C	3.72516704258364	-0.05003344910382	-0.50441574060468
O	3.58187601732433	-0.61263018991658	0.50044358606817
C	0.78076009876089	-1.37767252101718	-3.57923094204544
C	0.82723323166611	-2.04446788266113	-0.84141574703663
O	1.67411232113862	-2.75546229725103	-1.31981957891900
O	-0.04944569202804	-2.45398184151677	0.08332871484918

C	0.08119593524067	-3.82706838369997	0.50074186844833
O	1.66892491361845	-1.48779176576311	-4.38333143917906
O	-0.25208629995864	-2.22199263455907	-3.47759626503459
C	-0.18492957627429	-3.38259577191146	-4.32311197639343
N	0.54782848939994	0.07700373853582	1.11741415678244
C	0.60725413019789	1.37054040543068	-4.48491315247767
C	1.16326146130505	0.78922767181590	2.11018397479692
O	1.65497490558721	1.89250409408684	1.94579081657928
C	1.18363283283927	0.10885799229962	3.44655284992583
C	1.27016799727768	-1.05141515691138	5.97322782800069
C	1.11869100733789	0.90785949737285	4.58509093232416
C	1.30989420536993	-1.27216312515626	3.57706714416107
C	1.35742024031820	-1.84965877707433	4.83940247740962
C	1.15122286500457	0.32861449694547	5.84488004810719
C	0.57872908953843	3.51436964861798	-2.37885957347462
C	-0.15067727260367	4.47551721759017	-1.46982218358630
O	1.18371482592399	3.91100007300182	-3.35135654647218
H	0.44195478614717	2.49739772463835	0.06940123131120
H	1.07603592078817	-4.00107446552294	0.91181358859007
H	-0.08630156163070	-4.48920094513638	-0.34867944645838
H	-0.68330470835638	-3.97342763573481	1.26026687957233
H	0.70318255176253	-3.96671040512320	-4.07753830662609
H	-1.08959495929047	-3.94946202696076	-4.11431746707167
H	-0.15042536898720	-3.08524555295099	-5.37151597540198
H	0.17084990860332	-0.82491024444649	1.35574954482366
H	-0.00298018874037	2.24898757402751	-4.68552457130577
H	0.21002613347521	0.53398921593115	-5.05865365348605
H	1.61419201416403	1.58097899009453	-4.84949342897990
H	1.30354497258652	-1.50393618122644	6.95846747810746
H	1.04138593736577	1.98275492246683	4.47018440906692
H	1.41123633616962	-1.90302412585958	2.70047773005500
H	1.47144520236561	-2.92371587010915	4.93693541789691
H	1.08877264882817	0.95378717737864	6.72879798076141
H	0.43464665051821	4.63661583105382	-0.55977311057369
H	-1.12570430567646	4.08330352936526	-1.17440400215962
H	-0.26176259787803	5.42866372784186	-1.98480542512099

0 imaginary frequencies
E_{el}^{sp} (DLPNO-CCSD(T)) [Ha] = -1465.972377454713
$G - E_{el}$ [Ha] = 0.31737784

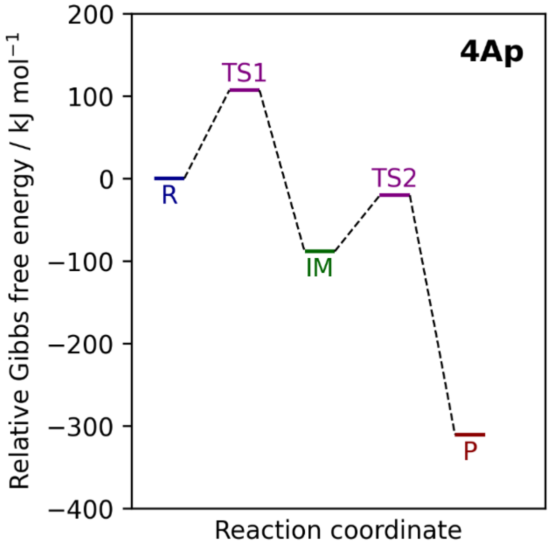

CASE 1u

R

50

C	0.58957988514548	0.82624009503736	-2.56380674221335
C	0.91359488867792	0.73472348954335	1.02738337562057
C	-0.58033763991304	2.62034974015384	0.70026028682070
C	-0.28613646379699	1.31737374056598	1.23725038563671
C	0.37286294133634	3.25142296505566	-0.00711691647534
C	-0.27836175548533	0.23739606167852	-1.98715859248343
O	1.60646977472995	2.67047898399080	-0.18342903516266
C	1.93836298140006	1.44760064440847	0.27504395504796
O	3.04280701048592	1.00474408150146	0.03190784906538
C	1.63702689078871	1.60860933485238	-3.21663106400966
C	-1.36638892182483	-0.42760250349106	-1.27237830717411
O	-2.45902453421235	0.06036981150978	-1.13816642576923
O	-0.98163158736118	-1.61039581439804	-0.81036890217500
C	-1.99719502161366	-2.36436959968148	-0.11273061074056
O	1.49382961510596	2.76055174623231	-3.53745699874987
O	2.73554245699343	0.88635390709688	-3.38525436486049
C	3.85586458340585	1.59047525562997	-3.95700489833413
N	1.32782067843918	-0.54203213435910	1.40492623182764
C	0.59024329715670	-1.48192288398502	2.05733820396964
O	-0.50635943954757	-1.24902546771973	2.54413790923128
C	1.21483706943098	-2.84161614156385	2.13902680949304
C	2.26958517785131	-5.40641103637484	2.35192764692461
C	2.07031030543066	-3.32861000068618	1.15302126801219
C	0.87770270565514	-3.64830952979671	3.22322479423974
C	1.41059154251675	-4.92370823573055	3.33410575815707
C	2.59358330677671	-4.61095642083735	1.25979176330661
C	0.33237467727089	4.59148556888698	-0.66808133860492
C	-1.95437640871295	3.18179695810894	0.94875793954539
C	1.11396285825646	5.64812389524764	0.12029340125550
H	-1.04853332889686	0.79511425238896	1.79730036935903
H	-1.50775780291616	-3.28529859809321	0.19447919690587
H	-2.82855837939833	-2.56950585237054	-0.78774729193342
H	-2.34024675509451	-1.80716479956619	0.75700721390715
H	4.65468161673405	0.85592885606398	-4.02029645742308
H	3.59509334255790	1.96765430323611	-4.94617996144039
H	4.14097508419912	2.41706129917797	-3.30579638327961
H	2.26567203973835	-0.76881089463702	1.10822480705125
H	2.68242913464115	-6.40605037878813	2.43586061884120
H	2.31149611056373	-2.72637726132771	0.28346678496228
H	0.19866287598446	-3.26266331711643	3.97462648420530
H	1.15517566952681	-5.54446734351299	4.18593235538385
H	3.25165070307704	-4.99005908528944	0.48565620537554
H	0.75693882401625	4.48029921150928	-1.66942338524458
H	-0.70447967630887	4.90182410992821	-0.79301769200918
H	-2.04480846186699	4.22154708603566	0.63969526021899
H	-2.70437715625268	2.59505903618171	0.41055850159682
H	-2.19327136268050	3.12454851630803	2.01397349504031
H	2.15596787900162	5.34626813989232	0.24860261997518
H	1.09965284737828	6.60429895588410	-0.40767951663610
H	0.67649592160935	5.79606725321885	1.11116339374226

0 imaginary frequencies

$E_{\mathrm{el}}^{\mathrm{sp}}$ (DLPNO-CCSD(T)) [Ha] = -1391.927684372138

$G - E_{\mathrm{el}}$ [Ha] = 0.33393417

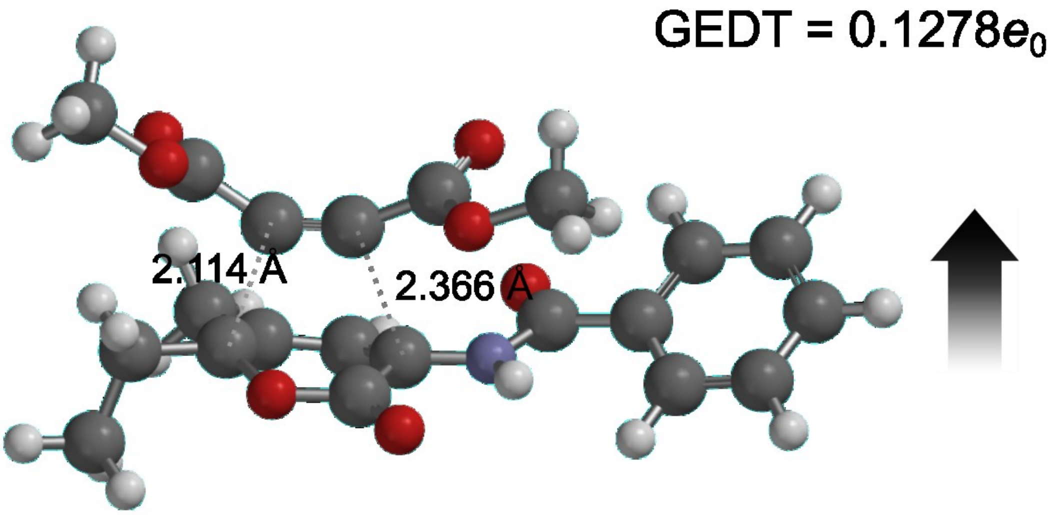

TS1

50

C	-0.35533063394819	1.18133424940058	-1.70575812328089
C	0.32400201196648	0.31359110392472	1.06281171513002
C	-0.60720886819489	2.45076152262459	0.58402481628678
C	-0.63454846173450	1.28619267954546	1.32653887073464
C	0.43526299122891	2.59231715285946	-0.34414326891218
C	-0.25127303092908	0.05094816832939	-1.21681165121169
O	1.61711797123490	1.91764999636845	-0.12115941256201
C	1.60931349788680	0.71422606613181	0.47888553545860
O	2.62488514411220	0.05913045117795	0.54981657780479
C	-0.83394016870341	1.99496534878978	-2.82638306040947
C	-0.37285989981813	-1.39508447683267	-1.17059635489264
O	-1.41484356368450	-1.97476891688525	-0.98088078559832
O	0.81116099420863	-1.99307380677042	-1.31737807235115
C	0.78772828868319	-3.43085300222415	-1.23702473214286
O	-1.88082640038807	2.59494229077976	-2.81936669542774
O	0.03796829422970	2.00118728222614	-3.83367225336206
C	-0.34199419850358	2.79351899801423	-4.97469022269016
N	0.37966713356682	-0.95066803514334	1.62393524423663
C	-0.67508750123213	-1.66908001817506	2.13716846180228
O	-1.78588055876465	-1.19963629619631	2.28603021803926
C	-0.34876750736424	-3.09182375871544	2.47416265352203
C	0.14095130612451	-5.77092348350784	3.03313330470377
C	0.92635995185745	-3.50313039263959	2.85662627597379
C	-1.38012284818101	-4.02303013525122	2.38483966402471
C	-1.13345987529563	-5.36070913051112	2.65564656007908
C	1.16766307318982	-4.84068867708539	3.14085606030272
C	0.70367669234093	3.85829941939747	-1.10659403199688
C	-1.74077807925683	3.43626206522646	0.62461581572749
C	1.45841627091688	4.88084077839710	-0.25078298405208
H	-1.46208238204565	1.06175946851474	1.98251258410182
H	1.82589233357851	-3.74193629731020	-1.32399477412866
H	0.19237331089086	-3.84178354064514	-2.05299690150136
H	0.37005396239844	-3.74969862667137	-0.28149773335346
H	0.47714958860122	2.69147817203336	-5.68258677441036
H	-1.27076563371663	2.41487136967878	-5.40216278270160
H	-0.47064560496205	3.83585898058159	-4.68053612040886

H	1.25583590957975	-1.42539258103508	1.45875985513436
H	0.33262637586051	-6.81649929284102	3.24919951768670
H	1.73406986790266	-2.78639823576040	2.96184678128388
H	-2.36721172741325	-3.68647174429656	2.09081166154532
H	-1.93590380905183	-6.08532931892818	2.57210086910569
H	2.15846677097631	-5.15516536553058	3.44955884880645
H	1.29490567638138	3.61180411218663	-1.99155005363084
H	-0.24639163371899	4.27346013983918	-1.44542369245200
H	-1.38585378752481	4.44801320204677	0.83357031648020
H	-2.26648310570457	3.45559333270203	-0.33328382232969
H	-2.45329912865572	3.15148949420643	1.39910738555679
H	2.42101877143141	4.47942850695455	0.07175389988621
H	1.64273242807359	5.78917533547892	-0.82833332691260
H	0.88625979157047	5.15304544553998	0.63929413730554

1 imaginary frequency (-390.93 cm^{-1})
$E_{\mathrm{el}}^{\mathrm{sp}}$ (DLPNO-CCSD(T)) [Ha] = -1391.897111465631
$G - E_{\mathrm{el}}$ [Ha] = 0.33794283

IM

50

C	0.70752893303898	1.75184051250254	-1.21902982733479
C	1.02048601257593	0.62432994478522	0.85787975734354
C	0.03823052876994	2.81807740451332	0.87396215375472
C	0.05825807696782	1.63493891936807	1.46864062954521
C	1.06137868668823	2.88960630388456	-0.26341970024022
C	0.71992484098783	0.55400199914566	-0.64999387375806
O	2.36757425930030	2.50691781605169	0.30731503587614
C	2.38266009369301	1.32596696379104	0.91457362125927
O	3.36300886955655	0.87103970366349	1.44246678742122
C	0.32529029177784	2.03477127034776	-2.63265143839927
C	0.43635067704288	-0.69561581490241	-1.40907143422104
O	-0.39980499770948	-0.76519592622716	-2.27471048373301
O	1.23070059955626	-1.70089072661374	-1.04447021001036
C	0.96911306043953	-2.96108652723319	-1.68547626876786
O	-0.61595449430654	2.73049080386821	-2.92262357635264
O	1.14911078406295	1.46363899436003	-3.50526509670386
C	0.78136134575508	1.60790337005257	-4.88915943833997
N	1.10612230913591	-0.61048164909653	1.57368949309215
C	0.06546423960533	-1.47821048364359	1.58596496573582
O	-0.99923615780717	-1.20392198156506	1.04738164782969
C	0.29737268826752	-2.78629859449231	2.27560605458900
C	0.63875411151078	-5.26653817532227	3.48798185664227
C	1.26518651455627	-2.95328028303885	3.26379467425915
C	-0.50530441031491	-3.86212356892518	1.90511304230076
C	-0.33064712009081	-5.10079807924112	2.50444221531773
C	1.43190645276352	-4.19124353819160	3.87047655613717
C	1.27638921140760	4.24036767757486	-0.92459073852802
C	-0.91916170768233	3.93015137433578	1.16301908522021
C	2.02305793436324	5.25163926731578	-0.05619224243507
H	-0.55138087894362	1.33352305554161	2.30952583204964
H	1.66940865771550	-3.66433165246573	-1.24050422568167
H	1.13412850154856	-2.87873690590966	-2.76016936227831
H	-0.05963296759252	-3.26765038697846	-1.49265627265101
H	1.55541787481069	1.09024532750355	-5.45083403064323
H	-0.19272764342469	1.14831396373986	-5.06013665656976
H	0.74601824013405	2.66291194510445	-5.16224706108503

H	2.03525126229888	-0.90941886684580	1.82573810299977
H	0.77233174914017	-6.23352426003323	3.96106053885853
H	1.87679832260049	-2.11801066734837	3.58734167154992
H	-1.26352868270109	-3.71277547857784	1.14544145087985
H	-0.95308974638587	-5.93780353309374	2.20752537157161
H	2.17928933209598	-4.31440379314883	4.64650867570022
H	1.83867457198368	4.08270726714285	-1.84970976785025
H	0.29722999711899	4.62333653722318	-1.21740662972002
H	-0.41221601027110	4.85399771121938	1.44564566403923
H	-1.52595286931521	4.14394091851984	0.27814798882964
H	-1.58970212353797	3.63960304402664	1.97276823390204
H	3.05608672539249	4.94160935481864	0.10379135297399
H	2.03267742406918	6.22394037677539	-0.55344847430339
H	1.55979662935130	5.38094406571867	0.92317734992835

0 imaginary frequencies
$E_{\mathrm{el}}^{\mathrm{sp}}$ (DLPNO-CCSD(T)) [Ha] = -1391.981042936751
$G - E_{\mathrm{el}}$ [Ha] = 0.34312894

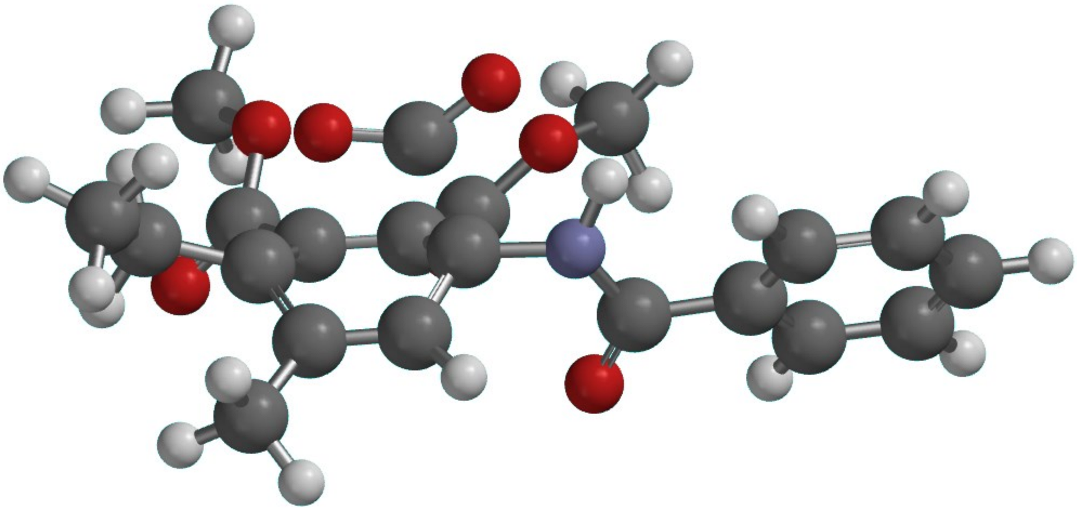

TS2
50

C	-0.27101620923029	1.50924553838282	-1.29646386382248
C	0.17786199770366	0.36408859945660	0.74592620516227
C	-0.66507536230506	2.62921185023463	0.85548616431734
C	-0.53275710483448	1.43171199687970	1.45287471196826
C	-0.09332145284887	2.69015288106742	-0.47073668905534
C	-0.15684783448931	0.31529546754621	-0.69193567440877
O	1.84241177493182	2.10554125730522	0.10506867513447
C	1.78805084115202	1.00566510070167	0.66674439974593
O	2.60976350824846	0.26875168839496	1.17714132769740
C	-0.47127691703894	1.67183645577903	-2.77338487099099
C	-0.26992775142201	-0.95271864878437	-1.47737335154729
O	-1.11278986012843	-1.11484793884090	-2.32267032486062
O	0.65534547230323	-1.84413468515005	-1.14228174639918
C	0.56531096072000	-3.11563921771094	-1.81059176887265
O	-1.37985764801653	2.30284625326196	-3.25103348685610

O	0.48472942915662	1.06683174720431	-3.47314112686741
C	0.31009710643051	1.07902565581150	-4.90134942281256
N	0.26870597396702	-0.86696489037391	1.46102135294694
C	-0.76761937984163	-1.73963796352168	1.47605938349370
O	-1.84601867100657	-1.46351279802782	0.96540652237770
C	-0.51440820632183	-3.06010606924072	2.13572683609273
C	-0.13576081175398	-5.56108433086378	3.29382834857928
C	0.49129640074365	-3.24667832157726	3.08165572436483
C	-1.33512206441170	-4.12728460332225	1.77973987005973
C	-1.14215086988128	-5.37618189229634	2.35178056202417
C	0.67656116149860	-4.49489450743841	3.66138511817000
C	0.23262685469517	3.97951097662873	-1.15729065429109
C	-1.27097481319530	3.82187089354994	1.54193264450616
C	0.97418711151938	5.03495584265270	-0.33612793089697
H	-0.81164340588454	1.25562603073029	2.48535222079470
H	1.37319406665514	-3.71622447441394	-1.39956416298794
H	0.68862366519633	-2.98376225681527	-2.88583858580669
H	-0.40191369120700	-3.57420027217402	-1.60206739425265
H	1.17082330086444	0.55013245194537	-5.30383232085809
H	-0.61619477881109	0.56579279762533	-5.16215335155442
H	0.28087232840759	2.10517955019151	-5.26857895598035
H	1.21456755398238	-1.16359870836700	1.65724468030078
H	0.01265157408709	-6.53615495905304	3.74546946859199
H	1.11955034372340	-2.41913067950571	3.39244922467656
H	-2.12136024500026	-3.96287456766446	1.05230317859812
H	-1.77943967113625	-6.20600542025707	2.06631920325902
H	1.45423216205296	-4.63340078580637	4.40440639376590
H	0.81698377688979	3.74190084767574	-2.04866108104649
H	-0.72432641005924	4.37476497605408	-1.52191655660006
H	-0.51020320236168	4.43099159555817	2.03398329822898
H	-1.81408666722760	4.45643696988400	0.83929001887322
H	-1.97198627842691	3.47660594731594	2.30327715445377
H	1.80429072422020	4.58918191575225	0.21183001946695
H	1.37946176259949	5.78685194757693	-1.01604439565475
H	0.32387945509184	5.54903075603830	0.36933500877196

1 imaginary frequency (-421.24 cm^{-1})
E_{el}^{sp} (DLPNO-CCSD(T)) [Ha] = -1391.949266079580
$G - E_{el}$ [Ha] = 0.33948220

P

50

C	0.18995057186636	1.93494297093408	-1.08981841871767
C	0.19488485803212	0.54694984604882	0.86996833672875
C	0.01292554798100	2.96147499624297	1.07123322280066
C	0.06223170969946	1.69792247209010	1.63882268550258
C	0.05581695969513	3.09096574836132	-0.32485746863154
C	0.28570924858914	0.66460485801802	-0.51468141920433
O	3.40420929357292	1.76978789754888	-0.00914100500613
C	3.35491219164751	0.93473757926354	0.79117256182312
O	3.32122928876795	0.10360681989597	1.60031437864836
C	0.29812723343476	2.05501227465844	-2.58550892935445
C	0.50407318376822	-0.50678873559441	-1.41979962160880
O	-0.08327600160490	-0.67877170785827	-2.45776490950805
O	1.47811155255771	-1.30510417657964	-0.97186661731512
C	1.69284589945848	-2.50188832502412	-1.73635274127934
O	-0.57837212275196	2.44942811232891	-3.31104604152112

O	1.51570410521785	1.69758265323466	-3.00738762839734
C	1.69032331231231	1.67003364970462	-4.43272745026389
N	0.26513259070246	-0.68662006528832	1.55091282945534
C	-0.38182942974071	-1.83174480342409	1.19156445875508
O	-1.14778345807557	-1.89247182172225	0.24455443447383
C	-0.08079207604028	-3.02385124174668	2.04789194308235
C	0.40788703339520	-5.29660422253723	3.57366165466121
C	1.15794107869064	-3.19968191821621	2.66072956153505
C	-1.06789628319585	-3.99614662168603	2.18773784734170
C	-0.82737493769166	-5.12501635226912	2.95655900464059
C	1.40149725393885	-4.33770419974527	3.41952683687284
C	0.04983604117898	4.45682831140682	-0.97134819843753
C	-0.10742480145858	4.16622632505759	1.96912716329138
C	1.47410113563377	4.96980064083755	-1.21709660578563
H	-0.01042340799780	1.59383379238290	2.71706748031629
H	2.47943069635119	-3.04422005929462	-1.21576155568231
H	2.00466339243837	-2.25212406579460	-2.75115239123346
H	0.77495722742418	-3.09019733180313	-1.76715111511347
H	2.71878849221571	1.35502519649647	-4.59566620437683
H	0.99328532612567	0.95714999993175	-4.87543706591590
H	1.52046797548861	2.66115413088550	-4.85482918604352
H	0.72353463891536	-0.67198094201342	2.44646848328573
H	0.59781073877215	-6.18284760956327	4.16967188149064
H	1.94841080152634	-2.46913801351283	2.52382704244346
H	-2.01984029053997	-3.85399674307082	1.68943994170249
H	-1.60191262390978	-5.87518628249589	3.07265250639677
H	2.37083215732767	-4.47782659491154	3.88528665912342
H	-0.49399143845507	4.41620926809056	-1.91492207398323
H	-0.48762943969674	5.16010521573903	-0.33354963331307
H	0.69202449386801	4.88632696672860	1.77976322031337
H	-1.05734218436899	4.68295696157328	1.80750394189734
H	-0.05801876769252	3.86890681677686	3.01739315513322
H	2.02119479889212	4.29926049934775	-1.88456837852884
H	1.45414543767377	5.96267969624027	-1.67335027247619
H	2.03690099606029	5.03475813432643	-0.28182630001783

0 imaginary frequencies
$E_{\mathrm{el}}^{\mathrm{sp}}$ (DLPNO-CCSD(T)) [Ha] = -1392.049772741333
$G - E_{\mathrm{el}}$ [Ha] = 0.33596795

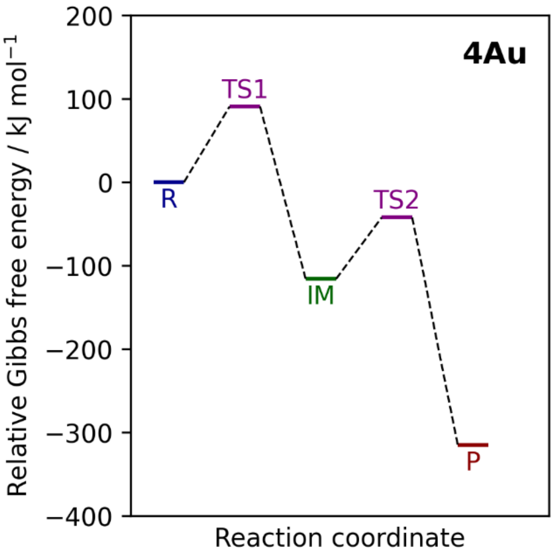

CASE 1x

R

64

C	0.15638057880029	-0.09246786036158	-1.72879688370606
C	2.75918796893121	-1.07280824455513	0.94763566832711
C	1.65799837528791	1.06247515311913	1.07051866409043
C	1.65966172175821	-0.35835869054413	1.26388772004958
C	2.78779510383558	1.68949810018264	0.68141155832224
C	-0.54598839745950	-0.88964354017281	-1.17576861967233
C	0.35565067938020	1.76526887294476	1.24851949246657
O	0.07355255430801	2.56577716267229	0.21945277031583
O	-0.37584890721054	1.58779245795808	2.19198608693829
C	-1.14044196130615	3.33992644507132	0.32437695169735
C	-1.20799589177837	4.23114608694527	-0.89511803623661
N	2.91965849064462	-2.45779300548105	0.99431459525625
C	1.92819531784515	-3.38752148898740	1.10838330529719
O	0.74843652539332	-3.09314802505283	1.21883493848895
O	3.91558206307246	0.96947926742356	0.45016182027078
C	1.08159645289938	0.86860426538615	-2.31314933809410
C	-1.39882549775428	-1.79162169970458	-0.40709420502345
O	-1.98422683721564	-1.43559546852072	0.58204790966680
O	-1.43617844841786	-3.00217068620842	-0.94601927796228
C	-2.19657352702331	-3.97560581076583	-0.20327199317815
O	0.44066668122669	1.80388878280069	-3.00191864093460
O	2.27526192352363	0.79959044795462	-2.16274269429653
C	1.27301786996222	2.84577237882337	-3.54937116363446
C	2.99908229098114	3.12782012247408	0.47351814784701
C	3.36683280128060	5.85929530504543	0.02655643714971
C	3.77259866034343	3.57136769126638	-0.60378317665389
C	2.43955879838323	4.06734185488697	1.33038429605886
C	2.61286454685740	5.42835521492296	1.11654466563748
C	3.95000402057939	4.92043607110865	-0.83010572473324
C	2.38692726771631	-4.81428137162828	1.07769770951532
C	3.11512249256192	-7.49863428566917	1.00462754386448
C	3.65208268550133	-5.20711231534929	1.50910900291070
C	1.48513104061821	-5.77202808720476	0.62027263171187
C	1.85053262102451	-7.10888586296834	0.57570758476441
C	4.01235462050048	-6.54802589304921	1.47544954067506
O	3.59607655947584	7.15998188523113	-0.27634634178080
C	3.02931675941531	8.15093852701060	0.57118657376777
C	3.96593140976607	-0.38853105326752	0.49830397570368
O	5.00057973840320	-0.94190424455852	0.20455454040726
H	0.75913143887649	-0.84179872266902	1.61162451588694
H	-1.10240439818122	3.91624390704035	1.25189618113053
H	-1.98690578776148	2.65231039455625	0.38160990964116
H	-2.10431258211274	4.85366244135195	-0.84259449325410
H	-1.25281757587474	3.63358120489941	-1.80739917002421
H	-0.33317283851367	4.88444685643312	-0.94360356536772
H	3.84857010715010	-2.76648964306924	0.74782563250981
H	-3.23608028944729	-3.65667251046195	-0.12247036488438
H	-1.76103418628674	-4.09481014010897	0.78856124684479
H	-2.12077817843792	-4.89730342839533	-0.77554361603981
H	1.76902370577840	3.38340388513375	-2.74080351244264
H	2.01540680811634	2.41797677692251	-4.22342843373541
H	0.59475807337817	3.50217179241806	-4.08914381112963
H	4.22101350131347	2.84774729212564	-1.27413461288835
H	1.86970779147785	3.74200214722346	2.19413491360374

H	2.17023634331173	6.13285601382426	1.80753746159518
H	4.54100538215660	5.27429633893331	-1.66709168470793
H	3.39961370706052	-8.54500132512275	0.97616201596887
H	4.35503949984062	-4.47961240812172	1.90059534444837
H	0.50089756838464	-5.45368914004674	0.29858687254695
H	1.14848753796917	-7.84967839842523	0.20885255965499
H	4.99416026734856	-6.85025862306144	1.82271733370699
H	3.32857140589940	9.11024242260797	0.15246529029925
H	1.93714395342851	8.08146571322482	0.58232348756574
H	3.41321959301285	8.06200869360885	1.59203246377632

0 imaginary frequencies
$E_{\mathrm{el}}^{\mathrm{sp}}$ (DLPNO-CCSD(T)) [Ha] = -1885.972878578885
$G - E_{\mathrm{el}}$ [Ha] = 0.42784579

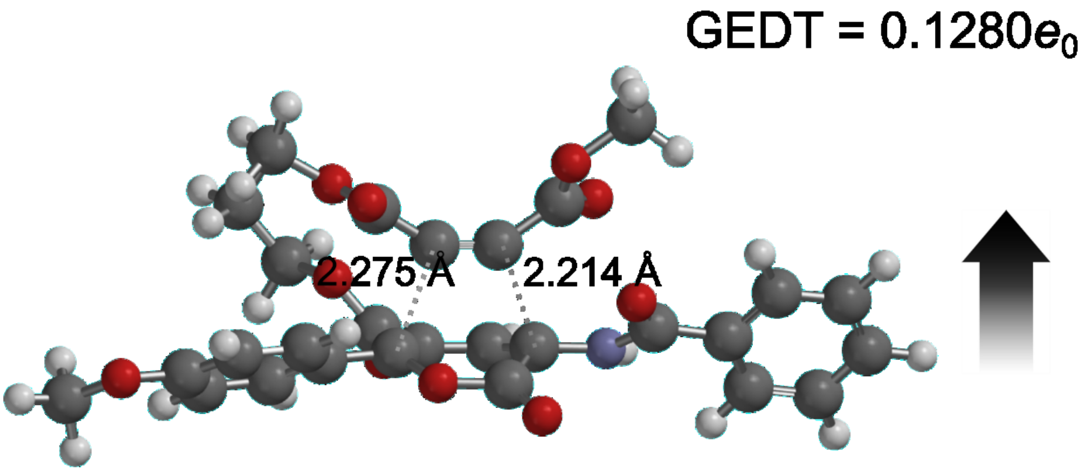

TS1
64

C	-0.36408245824921	0.00124622859396	-1.17142833476646
C	0.71682278601828	-1.62551802575497	0.98128057698628
C	0.09620948331062	0.63831620158189	1.43652566282162
C	0.02398473580229	-0.69841437990804	1.76099659450468
C	0.94996572300206	1.00825546758548	0.38918405780908
C	-0.44895577841698	-1.18934942966470	-0.84947896802170
C	-0.91698317817540	1.52244329083907	2.08412661747666
O	-1.60636011709524	2.24601452569109	1.21179251306041
O	-1.10819040949338	1.52362395012641	3.27770844854690
C	-2.56089856850032	3.17713531940507	1.76956003107475
C	-3.16637571236787	3.94496140792804	0.61760327632518
N	0.66417449080603	-2.97743654370983	1.32592785403416
C	0.93772439908748	-4.00848187622697	0.45585018222743
O	1.18063051248708	-3.82072649963722	-0.71936792893915
O	2.00107454781494	0.17756512770825	0.09889095810872
C	-0.61014254117635	1.01638516067649	-2.19085995107213
C	-1.17472136543374	-2.44792013686669	-1.07098082975683
O	-1.80652705649001	-3.01468407512136	-0.21268693565232
O	-1.10131423874124	-2.82577137939408	-2.34155529094927
C	-1.76005065301429	-4.06502505379666	-2.65272864178021

O	-1.57229386750394	1.86761563722572	-1.84372657149874
O	0.00773588085664	1.06582447950898	-3.22677113360498
C	-1.82503582157160	2.91679643922710	-2.79505986103500
C	1.25904238407445	2.38412098369199	-0.05481655548528
C	1.92676364529671	4.95863041383672	-0.92809332972282
C	1.88209440187380	2.58155519052290	-1.29278368270794
C	1.03042957067691	3.49296662350894	0.75272140383508
C	1.35245693673833	4.77451012358098	0.32828352891945
C	2.20169196779357	3.84861009558200	-1.73074652488683
C	0.88821533387791	-5.37511841803715	1.06088893537010
C	0.80294110545488	-7.96101691759067	2.07962132457919
C	1.14427424736825	-5.60222603900331	2.41115619963144
C	0.60392796878039	-6.44831934435976	0.22007655551513
C	0.55396220696416	-7.73691411420674	0.72928968980394
C	1.10384241230579	-6.89465899311457	2.91800305083813
O	2.26700946087800	6.16354367191845	-1.44207317451706
C	2.02212932629525	7.31948870582107	-0.65086900412401
C	1.98508095614279	-1.15150492313628	0.38923431294211
O	2.97464353038198	-1.79980199688202	0.20359379538886
H	-0.68512604608995	-1.04055146275731	2.50655075715668
H	-2.03389669210749	3.83277772399034	2.46714918551369
H	-3.31025188302687	2.61356429751139	2.32893277316653
H	-3.88761523089558	4.66906694685335	1.00385014462211
H	-3.68376394195381	3.27024046240895	-0.06721414070887
H	-2.39367551331161	4.48366871676443	0.06454466136910
H	0.12892321982651	-3.20608831165189	2.14832303301767
H	-2.82886499440230	-3.98710158782884	-2.45011820701885
H	-1.32857368465749	-4.87108234462894	-2.05847605105639
H	-1.57975024176655	-4.23022403956967	-3.71236385194943
H	-0.93458066768514	3.53717053824660	-2.90674123785457
H	-2.10266030194047	2.49444587363606	-3.76115572193651
H	-2.64607187780771	3.49505698189589	-2.37882787617961
H	2.11346353953645	1.73147183012444	-1.92090120462615
H	0.63239335966156	3.37966326781262	1.75260519691536
H	1.16874484192920	5.60958142098381	0.99044973893170
H	2.67283866679277	4.00163813796975	-2.69466756473354
H	0.76946175245544	-8.96977725562553	2.47705151301136
H	1.40742331623401	-4.77916833738933	3.06650316695855
H	0.42531238610277	-6.25855360402570	-0.83155636207450
H	0.32461425334139	-8.56936010748409	0.07317886292508
H	1.31487969944697	-7.06918824173122	3.96726764583509
H	2.35778259910120	8.16653614128485	-1.24656376378131
H	0.95544388746289	7.42639977671905	-0.43070576373147
H	2.58865330589482	7.28309227834143	0.28459621494969

1 imaginary frequency (-453.30 cm^{-1})
$E_{\mathrm{el}}^{\mathrm{sp}}$ (DLPNO-CCSD(T)) [Ha] = -1885.921049232456
$G - E_{\mathrm{el}}$ [Ha] = 0.43031405

IM
64

C	1.42401870495892	0.38895431379749	-0.94492369354574
C	1.90363128432638	-1.12706559501826	0.85885582140833
C	1.28297727676076	1.12329883567366	1.37599583639564
C	1.22111690980315	-0.13538306794805	1.78278810679008
C	2.10615475613069	1.29389500032495	0.09175006959957
C	1.35806740772917	-0.87948010331184	-0.55916102469379
C	0.51421946032389	2.18486735735857	2.08571477370436

O	-0.06933177860917	3.01961729110538	1.23483906697703
O	0.40880139065748	2.24149612266562	3.28778314493880
C	-0.77032107625228	4.14519518307955	1.80856399616838
C	-1.22222244568787	5.01764973728519	0.65991153486604
N	1.84285894638413	-2.46469813569516	1.35435664999547
C	2.35902174709168	-3.50740360606681	0.64909820218518
O	2.85144924183128	-3.34679430526629	-0.45662500308571
O	3.40944383314388	0.65711669382729	0.36919698125335
C	0.71216233855894	0.94948091116886	-2.13593568774460
C	0.60188216483666	-1.97386397721769	-1.23877769555491
O	-0.27364196357568	-2.58145660557344	-0.66847873512711
O	0.97302812318836	-2.16710106469946	-2.49109903639687
C	0.29110212643832	-3.22557165470235	-3.18407734348020
O	-0.18642782922830	1.84708572720921	-1.73002200765116
O	0.90772543333561	0.65122083350715	-3.28485617868659
C	-0.88530516130042	2.54696406875308	-2.77309807714547
C	2.49152829737408	2.69653545405564	-0.30614495662045
C	3.38557296268906	5.26873113493646	-0.97185152437216
C	2.73247363048751	3.04754932739411	-1.63334564738576
C	2.76790993413269	3.63635929733670	0.67749087179427
C	3.20285234583169	4.91672554106346	0.36349484237095
C	3.16119012858099	4.31803319076933	-1.96775026346720
C	2.26261740070353	-4.85036483969529	1.29900688802349
C	2.10854972883638	-7.39340372819741	2.41021684556282
C	2.28448485363342	-5.00894735779501	2.68237259004354
C	2.17654898793724	-5.96809949517031	0.47345403262373
C	2.09145741171722	-7.23632608394178	1.02807175690016
C	2.21113475338773	-6.28054944264420	3.23606034382200
O	3.79239963718729	6.49158187241765	-1.39298786139800
C	4.05682302886050	7.47933206183241	-0.40555080236267
C	3.35580319872077	-0.59885170476877	0.82190419044256
O	4.32210477336168	-1.19881943026803	1.18742057927291
H	0.71654076471504	-0.46738328231097	2.68132324378565
H	-0.08473318949548	4.67068627917366	2.47728481081415
H	-1.60860965782452	3.77052331769108	2.39963594699809
H	-1.74327076784287	5.89465788095352	1.05094879242693
H	-1.90590341907487	4.47320895589246	0.00497184175797
H	-0.36501514124568	5.35398784973792	0.07201097708080
H	1.25693374653412	-2.65025445903098	2.14954002180039
H	-0.78014542734404	-3.02482369332120	-3.22063330691125
H	0.47241518847882	-4.17456139949211	-2.67758690317039
H	0.71566810844997	-3.23592727930815	-4.18496993585902
H	-0.17320068621311	3.08999681334489	-3.39627103113912
H	-1.45063563304416	1.84459650490046	-3.38609852669625
H	-1.55265168063470	3.23758271879510	-2.26304289435053
H	2.61608796946350	2.32696518548232	-2.43310792319691
H	2.66638786665755	3.38105973336495	1.72674507900919
H	3.40159471502976	5.61748239685645	1.16304282013124
H	3.34253303485161	4.58833828761273	-3.00163955728923
H	2.04874116200865	-8.38589827999228	2.84390421278233
H	2.39249745085147	-4.14741243256555	3.33256343490851
H	2.17641468153803	-5.82866228275983	-0.60127864433471
H	2.01543744009569	-8.10475514697240	0.38297228816009
H	2.24184663643396	-6.40247049281231	4.31317929669510
H	4.36331032630439	8.37172809739278	-0.94863173310618
H	3.15983575700817	7.69888019147355	0.18203128229484
H	4.86405779001126	7.16266177831228	0.26208382098806

0 imaginary frequencies
$E_{\mathrm{el}}^{\mathrm{sp}}$ (DLPNO-CCSD(T)) [Ha] = -1886.004896448215
$G - E_{\mathrm{el}}$ [Ha] = 0.43408895

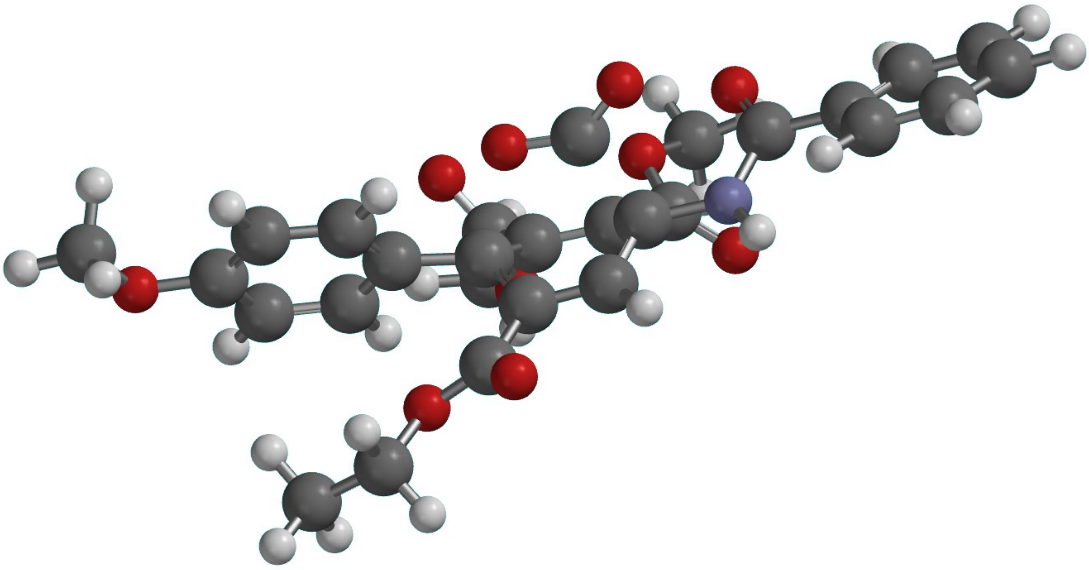

TS2
64

C	-0.35269204548961	0.01502779249884	-1.03485736265907
C	-0.08079262944406	-1.56714828291028	0.74504197388217
C	-0.47150536525107	0.72220487036678	1.31484276000391
C	-0.53814224408459	-0.56289910768068	1.69775533083600
C	0.08075131411168	0.96110821446020	-0.01631147791674
C	-0.46573809999838	-1.26734822220693	-0.64494681835951
C	-0.83710039167633	1.78411438942612	2.30052634959119
O	-1.30080331152605	2.88715626524294	1.72574923578791
O	-0.76229149041998	1.61871810606603	3.49567213888368
C	-1.62953736722514	3.98295268003386	2.60986865158203
C	-2.02285555964008	5.15683914355944	1.74310052043899
N	-0.17252775199954	-2.90391709856059	1.20685513030156
C	0.45382516989925	-3.94359402769622	0.58789409828100
O	0.91931771487323	-3.84043319994385	-0.53532681421346
O	1.79722169864550	0.08372482683522	0.31309383789531
C	-0.41378866080967	0.38622146320469	-2.48667188248319
C	-0.93674521025283	-2.34901951915908	-1.57126615593070
O	-1.86618779275712	-3.06430507969447	-1.29734838361512
O	-0.27827191145586	-2.34830307303103	-2.72249657549049
C	-0.68688792927725	-3.34891071044580	-3.66892772569056
O	-1.64568538667289	0.22945894783285	-2.97415226374718
O	0.53428242995347	0.76397570227566	-3.12059058935736
C	-1.77448502557209	0.43329114629118	-4.39257984432810
C	0.50807568480956	2.33400788807489	-0.36430670182879
C	1.19443220486427	4.99803004643794	-0.84178740185556
C	-0.20693285864438	3.09495729256069	-1.29079240614747
C	1.55003823885033	2.93200300490866	0.33159793310772
C	1.90952875898382	4.25139279727121	0.09159459210438
C	0.12490483979939	4.41238657043079	-1.52346505554507
C	0.49212931770837	-5.22933490987217	1.35160669644065
C	0.59829227993631	-7.67431644330532	2.67148461408452
C	0.58810949070265	-5.26893865776714	2.74040157370031

C	0.46462298042183	-6.41673649974760	0.62507916797169
C	0.50886188173532	-7.63628250869145	1.28378804131094
C	0.64462941329457	-6.49086382954315	3.39814523733934
O	1.45299346962212	6.29276503881502	-1.14528556968416
C	2.55640059319857	6.91989165590826	-0.50329940474079
C	1.63363562324801	-1.06367922009142	0.77201789799546
O	2.35486459546352	-1.89234687068529	1.25999857819096
H	-0.80136331868749	-0.84333838995691	2.71127740147583
H	-0.75369235333598	4.20209881437323	3.22428338637790
H	-2.44078192553664	3.66514445855173	3.26836328675923
H	-2.27792396015316	6.00738295594956	2.37969493833575
H	-2.89169128351091	4.91271991137376	1.12783337398369
H	-1.19978793209204	5.44621983367570	1.08681074973325
H	-0.54532275311397	-3.05019236168236	2.12879098817929
H	-1.73396155568171	-3.20589667676262	-3.93940158894694
H	-0.54710949575324	-4.34036575782982	-3.23719416827714
H	-0.04153160278837	-3.21172513444284	-4.53342592752294
H	-1.50019508919058	1.45632936790590	-4.65211956550474
H	-1.12940956754724	-0.26567268826230	-4.92656456062747
H	-2.82093346680702	0.24459229568055	-4.62053097795007
H	-1.06410595458442	2.67927682563635	-1.80691699555717
H	2.10419890332213	2.35817487863246	1.06406796335247
H	2.73999447713468	4.67927205966096	0.63648632045604
H	-0.43549681829328	5.01128846896036	-2.23187197903196
H	0.63960605843258	-8.62812795742061	3.18656086940153
H	0.65731722639970	-4.35158548335998	3.31553515054785
H	0.40628793686542	-6.36983178457038	-0.45596420659380
H	0.47733687605647	-8.55935242641822	0.71537492615001
H	0.73303231494452	-6.51788732478433	4.47862782869763
H	2.59889317623209	7.93186355420924	-0.90237606514274
H	2.41213078307978	6.96066408908937	0.58067224240414
H	3.49056265668387	6.39709789032241	-0.72971531683605

1 imaginary frequency (-566.79 cm^{-1})
E_{el}^{sp} (DLPNO-CCSD(T)) [Ha] = -1885.982578785906
$G - E_{el}$ [Ha] = 0.43197318

P

64

C	1.24859677074577	0.52456759645757	-0.91895965656053
C	1.15843290855755	-1.14697939971735	0.81141425359926
C	1.41851129559017	1.18263077876911	1.37209682898767
C	1.33275098012378	-0.14632076009464	1.75505054360724
C	1.39834229363835	1.54253764346465	0.02327919438657
C	1.09918826310085	-0.80782529424584	-0.54004952916863
C	1.56749659225938	2.19470282374678	2.46883224035361
O	0.73923147857614	3.22230371524239	2.32050035553398
O	2.33955409959151	2.06422395851232	3.38939302754879
C	0.91071204641670	4.33193456180545	3.22961127038722
C	0.06397950382310	5.47016599710685	2.70710098899219
N	1.06497648992549	-2.48289390919296	1.25608186672206
C	1.84131376660675	-3.49500055509345	0.76631865754961
O	2.66304823545529	-3.31718476212268	-0.11848216491090
O	4.41490125608283	-0.45869392373772	0.05137586605114
C	1.27030422023677	0.88388385130374	-2.37895517993551
C	0.85597099136852	-1.86913960811429	-1.57222632383800
O	-0.04062119338141	-2.67230314937362	-1.50875040173274
O	1.73246357920824	-1.79137775041340	-2.56945817625112

C	1.57443701533584	-2.76472225235656	-3.61305183439650
O	0.10061169166767	0.59998597601015	-2.96150005579540
O	2.21173984203842	1.37164046592430	-2.94553587665619
C	0.04520245780026	0.83876298848032	-4.37740360641396
C	1.55151723724753	2.95833860955018	-0.40110050888057
C	1.82495056204656	5.64002515996330	-1.14060042631569
C	0.54001871556511	3.60519659527400	-1.11047989915960
C	2.69750878169318	3.66882553572356	-0.07834727313423
C	2.84555312298620	5.00376990103608	-0.43969064338063
C	0.67011580705177	4.93066560589295	-1.47667549770905
C	1.62241425112694	-4.83311441482775	1.39537987954654
C	1.30422048933106	-7.35590945516894	2.51639009440323
C	0.37777568894536	-5.22810930887914	1.87969028990831
C	2.70361205636815	-5.70817884146441	1.46049237808025
C	2.54664047896923	-6.96356604536521	2.02840063841474
C	0.21959911891735	-6.49099368154616	2.43581836044005
O	1.86137137637366	6.94001087661408	-1.53736325936089
C	3.02143785179961	7.69509628818833	-1.21977919876218
C	4.33575710159878	-1.09540051599861	1.01551090566934
O	4.27056773002828	-1.71816289125956	1.99214667608406
H	1.40660630063282	-0.40669425630037	2.80501070059055
H	1.97104720233855	4.59163533022816	3.25734838073651
H	0.60713798831911	4.01358423747022	4.22955660043520
H	0.16686896110714	6.33409145472968	3.36811512639778
H	-0.99024262821889	5.18680575448731	2.67037324390485
H	0.38504717350998	5.75779519507208	1.70336740912415
H	0.58871910610906	-2.63668272184932	2.12964852769737
H	0.59096436744343	-2.66532800086023	-4.07478770717560
H	1.68951843347041	-3.76825589888754	-3.20226703513515
H	2.36179815635550	-2.55079227460011	-4.33224466634548
H	0.21794819633181	1.89488971182724	-4.58762008759405
H	0.79973235602760	0.23695868298769	-4.88603915952887
H	-0.95521378904202	0.54300809184968	-4.68534602090353
H	-0.36811776715146	3.06789956022977	-1.36455970379778
H	3.50049382605395	3.17622533304471	0.46110094935784
H	3.75578304251304	5.52530104438745	-0.17570147148183
H	-0.11672892207975	5.44083432639719	-2.02082964229389
H	1.17993635075267	-8.34046325045359	2.95444086717518
H	-0.47848580953188	-4.56766577976601	1.79405654164982
H	3.66213148515955	-5.39098449321348	1.06663974063653
H	3.39270634639555	-7.63932971288731	2.08821535319992
H	-0.75342142531521	-6.80201546475291	2.79983393558328
H	2.85027273685907	8.69283370862740	-1.62097274589406
H	3.16789824518133	7.75761205681536	-0.13662815674889
H	3.91340511196146	7.26309495532297	-1.68451578349341

0 imaginary frequencies
$E_{\mathrm{el}}^{\mathrm{sp}}$ (DLPNO-CCSD(T)) [Ha] = -1886.096134480758
$G - E_{\mathrm{el}}$ [Ha] = 0.42872374

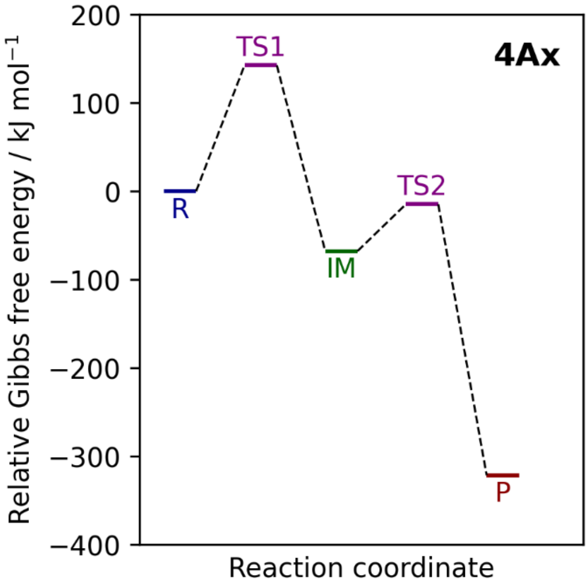
4Ax
TS1
TS2
R
IM
P
200
100
0
−100
−200
−300
−400
Relative Gibbs free energy / kJ mol−1
Reaction coordinate
